# Direct Metal-Free Transformation of Alkynes to Nitriles: Computational Evidence for the Precise Reaction Mechanism

**DOI:** 10.3390/ijms22063193

**Published:** 2021-03-21

**Authors:** Lucija Hok, Robert Vianello

**Affiliations:** Division of Organic Chemistry and Biochemistry, Ruđer Bošković Institute, Bijenička Cesta 54, 10000 Zagreb, Croatia; lucija.hok@irb.hr

**Keywords:** azides, DFT calculations, cyanides, *N*-iodosuccinimide, triple C≡C bond cleavage

## Abstract

Density functional theory calculations elucidated the precise reaction mechanism for the conversion of diphenylacetylenes into benzonitriles involving the cleavage of the triple C≡C bond, with *N*-iodosuccinimide (NIS) as an oxidant and trimethylsilyl azide (TMSN_3_) as a nitrogen donor. The reaction requires six steps with the activation barrier Δ*G*^‡^ = 33.5 kcal mol^−1^ and a highly exergonic reaction free-energy Δ*G*_R_ = −191.9 kcal mol^−1^ in MeCN. Reaction profiles agree with several experimental observations, offering evidence for the formation of molecular I_2_, interpreting the necessity to increase the temperature to finalize the reaction, and revealing thermodynamic aspects allowing higher yields for alkynes with para-electron-donating groups. In addition, the proposed mechanism indicates usefulness of this concept for both internal and terminal alkynes, eliminates the option to replace NIS by its Cl- or Br-analogues, and strongly promotes NaN_3_ as an alternative to TMSN_3_. Lastly, our results advise increasing the solvent polarity as another route to advance this metal-free strategy towards more efficient processes.

## 1. Introduction

**Scheme 1:**
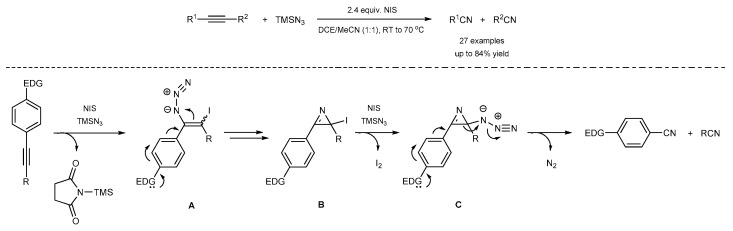
Triple C≡C bond cleavage reported by Yanada and co-workers [22] (**top**), and their proposed mechanism (**bottom**).

The transformation of alkynes is a fundamental method that has been widely used in organic synthesis [1,2,3,4,5] to afford ketones [6,7], diketones [8,9], acids and their derivatives [10,11,12], alkyl or alkenyl halides [13,14], cycloalkanes or cycloalkenes [15,16,17], and nitriles [18,19,20,21,22,23,24,25], which makes these processes highly appealing in academic research and industrial applications [5]. Amongst many reactions of the C≡C triple bond, its cleavage is deemed to be one of the most challenging targets and has stimulated new conceptual strategies in the organic community in recent years [5,10,11,12,15,16,17,18,19,20,21,22,23,24,25]. A triple bond having three shared electron pairs between two atoms is known as one of the strongest chemical bonds [4,5]. In spite of its large bond dissociation energy (>200 kcal mol^−1^), a complete cleavage of a C≡C triple bond is common in general organic chemistry [26]. Different from conventional organic synthesis, this methodology enables the reorganization of molecular skeletons and leads to the collection of valuable structural units in a straightforward and atom-economic fashion by retrosynthetic disconnections [10]. Most such approaches rely on oxidative cleavage with stoichiometric organometallic reagents and oxidants [27], such as Au [15,28], Ag [21], Pd [29,30], Ru [31,32], Rh [33], and Cu [34] species. These methods are promising since alkynes are excellent transition metal ligands and their complexes exhibit a variety of reactivities. However, these methods suffer from harsh reaction conditions, restricted substrate scope, and multiple limitations, such as environmental unfriendliness, the use of costly and/or toxic catalysts, and possible transition-metal impurities, which underlines the need to develop metal-free routes to the C≡C bond cleavage [23,24,25] that are currently rare. Recently, important progress was made by Yanada and co-workers [22], who reported the *N*-iodosuccinimide-mediated direct cleavage of internal alkynes to nitriles using trimethylsilyl azide (TMSN_3_) as a nitrogen source (Scheme 1).

Nitriles are versatile building blocks and precursors in organic synthesis, as they can be easily transformed into aldehydes, amines, amides, thioamides, acids, esters, heterocyclic compounds, etc. [35,36,37]. The cyano group is ubiquitous in useful natural products, pharmaceuticals, top-selling drugs, agricultural chemicals, functional materials, and dyes [38,39,40,41,42,43,44]. Conventional methods to access nitriles, such as the Sandmeyer [45] and Rosenmund von Braun reactions [46,47], or transition-metal-mediated cyanation of aryl halides with a cyanide source [48,49,50], were widely employed in the early years. Although mostly quite efficient, these methods are generally not favored due to the use of toxic metals and the emission of hazardous HCN gas [51]. As mentioned, nitrogenation of alkynes with TMSN_3_, achieved by Yanada’s group, does not employ cyanide salts and metal oxidants, which certainly promotes this sustainable route as a topic of considerable interest. Using 2.4 equivalents of NIS and TMSN_3_ in DCE:MeCN (1:1), starting at room temperature and rising to 70 °C, the authors explored the reactivity of an array of terminal and internal diaryl- and aryl-alkyl alkynes. In the case of symmetrical and unsymmetrical systems, the reaction proceeded quite satisfactorily, especially involving aryl moieties with an electron-donating group [22,51].

Inspired by these experimental advances, we used DFT calculations to clarify the precise mechanism of Yanada’s reactions. The mechanistic pathway postulated by authors (Scheme 1) [22] relies on the formation of an unstable iodo vinyl azide as the key-intermediate A, in which the electron-donating group (EDG) on the aryl ring can accelerate the formation of intermediate B, releasing N_2_ under thermal conditions, the latter proposed as the driving force of this process. Subsequent nucleophilic substitution of iodoazirine B with azide anion gives an intermediate C, and finally a ring-opening reaction affords the matching nitriles. Despite the proposed mechanism, the conversion of the key-vinyl iodide derivatives into nitriles deserves further studies, in order to elucidate transition via iodo azirine derivatives and provide deeper insights into alkyne cleavage reactions. Our calculations show different mechanistic routes to those proposed by Yanada [22] and reveal a very modest electronic effect of the *para*-EDG substituents on the reaction outcomes while offering guidelines to advance this reaction strategy and design new catalytic reaction systems towards even more efficient transformations.

## 2. Results and Discussion

**Figure 1:**
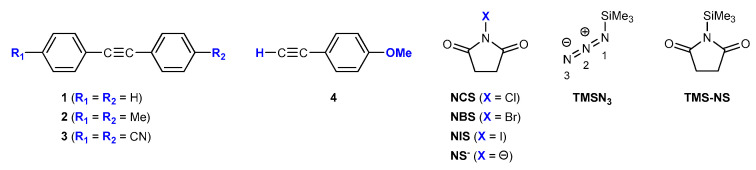
Chemical structures of investigated systems.

Chemical structures of studied systems with the relevant atom labeling used throughout the text are presented in Figure 1.

### 2.1. The Uncatalyzed Reaction with N_2_ as a Nitrogen Source in Acetonitrile

**Scheme 2:**
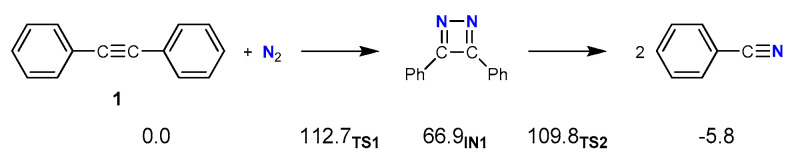
Direct conversion of **1** into benzonitriles with N_2_. Relative Gibbs free energies (in kcal mol^−1^) correspond to the MeCN solution. TS and IN denote transition states and intermediates.

As mentioned, one of the dominant reasons for the low reactivity of alkynes is the high intrinsic bond dissociation energies of the C≡C linkage, which often exceed 200 kcal mol^−1^ as opposed to those for the single C–C bonds that are typically found below 100 kcal mol^−1^ [52]. Our data for the parent diphenylacetylene **1** reveal the bond dissociation energy of 180.7 kcal mol^−1^ in MeCN, tying in with the preceding conclusion. Nevertheless, to further demonstrate the difficulty of cleaving the C≡C bond and converting alkynes into the matching nitriles, we studied a potential uncatalyzed direct transformation of **1** into two benzonitriles using molecular N_2_ as a nitrogen source, as depicted in Scheme 2.

Interestingly, the examined process is thermodynamically feasible with an exergonic reaction free energy Δ*G*_R_ = −5.8 kcal mol^−1^. However, the activation free energy is extremely high at Δ*G*^‡^ = 112.7 kcal mol^−1^ for the initial rate-limiting attachment of N_2_ onto the C≡C bond, making this process highly unlikely even under elevated temperatures. The first transition state TS1 is not symmetrical, rather one of the N-atoms from N_2_ attaches to alkyne carbon (d_C–N_ = 1.42 Å), while the other is further away from its vicinal partner (2.35 Å). All of this elongates the central C–C bond, from 1.21 Å in **1** to 1.39 Å in TS1, and then to 1.53 Å in the IN1 intermediate. This makes the subsequent carbon–carbon cleavage slightly less demanding, and occurring over the transition state TS2, being 109.8 kcal mol^–1^ higher than **1**. The obtained profile ties in with recently underlined ideas about the unsymmetrical character of various cycloaddition reactions [53], and strongly emphasizes the need for the catalytic environment in order for this reaction concept to occur under normal conditions.

### 2.2. Reaction on Diphenylacetylene with NIS and TMSN_3_ in Acetonitrile

Diphenylacetylene **1** is a symmetrical molecule, making both of its alkyne C-atoms likely candidates to undergo nucleophilic or electrophilic reactivity, as well as underlying the possibility that both reactants, NIS and TMSN_3_, attack the triple bond either in a concerted or a stepwise fashion. Our calculations show that a stepwise activation of the triple bond in **1** by either reactant offers no stable intermediates. In other words, the scan of the N–I∙∙∙C_alkyne_(**1**) coordinate with NIS, or either of the Si–N1∙∙∙C_alkyne_(**1**) or Si–N=N=N3∙∙∙C_alkyne_(**1**) coordinates with TMSN_3_, proceeds uphill in energy with no indications of any transition states or formed products. We also considered two additional possibilities for the initial TMSN_3_ approach, namely a simultaneous attack of (i) Si and N1 atoms, or (ii) N1 and N3 atoms onto the C≡C bond. The first attempt gave the four membered C≡C–N1–Si ring, yet with a very high activation barrier Δ*G*^‡^ = 50.1 kcal mol^–1^, while the second one, in line with a proposed such 1,3-cycloaddition reactivity of HN_3_ onto the alkene bond [21], produced a triazole through a one-step process [53], having the N1-attached SiMe_3_ group, and a much lower activation barrier Δ*G*^‡^ = 30.4 kcal mol^–1^. Nevertheless, the former intermediate is very stable (−71.1 kcal mol^–1^), with no tendency to easily lose the –SiMe_3_ group, thus hindering any further reaction progress, either (i) through a direct cleavage of the N–Si bond, being uphill in energy and raising the overall activation energy to Δ*G*^‡^ = 53.6 kcal mol^–1^, or (ii) by the approach of NIS (even its more nucleophilic I^–^ anion) towards it, which, in both cases, gave no stable products. All of these led us to rule out the possibility for a stepwise initiation of the C≡C bond cleavage.

**Figure 2:**
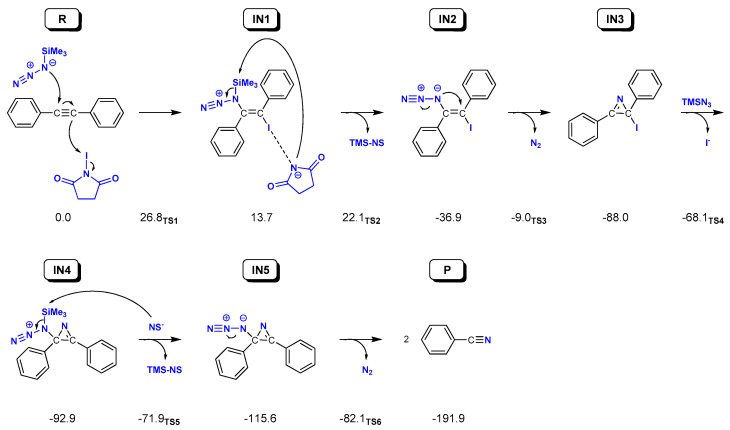
Reaction mechanism for the conversion of diphenylacetylene **1** to benzonitrile with *N*-iodosuccinimide (NIS) as an oxidant and trimethylsilyl azide (TMSN_3_) as a nitrogen source. Relative Gibbs free energies (in kcal mol^–1^) correspond to the MeCN solution. TS and IN denote transition states and intermediates, while R and P stand for reactants and products. Graphical representation of the reaction profile is given as Figure S1.

A one-step approach of NIS and TMSN_3_ onto the C≡C bond in **1** resulted as a likely mechanism to initiate the reaction. From the perspective of TMSN_3_, this can occur with either its N1 or terminal N3 atoms (Figure 1), with the latter being more demanding with a higher activation energy Δ*G*^‡^ = 36.1 kcal mol^−1^. This is reasonable given the lower N3-nucleophilicity, as seen in its atomic charge of −0.14 |e| in the isolated TMSN_3_, as opposed to a much higher charge of −0.74 |e| on N1, being in line with many reports on the reactivity of this system [54,55,56,57]. As such, a much more feasible route occurs if TMSN_3_ approaches **1** with its N1 atom (Figure 2), offering the first transition state TS1, being 26.8 kcal mol^–1^ higher than reactants R. There, TMSN_3_ acts as a nucleophile, given the mentioned charge on its N1 atom of −0.74 |e| and within the azide unit of −0.61 |e| in R, to assume −0.66 and −0.32 |e| in the transition state TS1, respectively. In contrast, NIS participates as an electrophile, seen in the charge on the iodine atom being changed from 0.42 |e| in reactants to 0.31 |e| in TS1. All of this reduces the bond order of the central C≡C linkage and elongates it from 1.21 to 1.29 Å, in the same order. The latter is accompanied by the bending of the C_phenyl_–C≡C angles, from linearity in R to values of 131.3 and 141.9° in TS1, where, accordingly, the charge on the alkyne C-atom to accommodate the nucleophilic azide is 0.16 |e|, while on its vicinal counterpart, to accept the electrophilic I^+^ cation, it is −0.24 |e|, thus firmly supporting previous conclusions.

The initial step is endergonic (Δ*G*_R_ = 13.7 kcal mol^−1^), giving a high-energy alkene intermediate IN1, most favorably as the *E*-isomer (Figure 2). An alternative approach of NIS and TMSN_3_ that would give the matching *Z*-isomer is sterically hindered and linked with a 4.9 kcal mol^−1^ higher barrier, thus is disfavored and not considered further. However, it is worth mentioning that such a kinetic difference still allows for the formation of the corresponding *Z*-isomer, but at significantly lower yields and around 3–4 orders of magnitude slower rates, thus placing our results in qualitative agreement with Yanada and co-workers [22], who reported the isolation of a mixture of *E*- and *Z*-stereoisomers in around 2:1 ratio. The relative instability of IN1 has a dominant contribution in the fact that the azide moiety still bears the attached –SiMe_3_ group, with the Si–N distance being not much changed from 1.78 Å in TMSN_3_ into 1.88 Å in IN1. Additionally, the formed succinimide anion NS^–^ is still partially bonded to iodine in IN1 (d_N–I_ is 2.06 Å in R, 2.20 Å in TS1, and 2.36 Å in IN1). While its exclusion from IN1 would be favorable (Δ*G*_R_ = −12.5 kcal mol^−1^), in the next step, NS^–^ approaches the Si-atom and cleaves the Si–N bond within TMSN_3_. This nucleophilic attack is supported by the positive charge on the Si-atom of 1.91 |e| in IN1, being further increased from 1.86 |e| in the isolated TMSN_3_, thus facilitating the reaction. The latter requires only 8.4 kcal mol^–1^ to reach the second transition state TS2 describing the cleavage of the mentioned Si–N bond in TMSN_3_ and the formation of a new Si–N bond with NS^–^ to offer TMS–NS as a byproduct (Figure 2). Departure of the latter is favored (−11.6 kcal mol^−1^), contributing to the total reaction free energy for the formation of intermediate IN2 of Δ*G*_R_ = −36.9 kcal mol^−1^, thus overcoming the potential earlier exclusion of NS^–^ from IN1 and allowing the reaction to proceed to this point. IN2 features azide and iodine attached as *E*-moieties to the rest of the 1,2-diphenylvinyl skeleton, although significantly twisted, with a torsional angle between phenyl rings of 47.5°, imposed to reduce steric interference among substituents. In addition, the central C–C linkage assumes a typical alkene double bond distance of 1.35 Å, with the attached azide and iodine at the matching C–N and C–I distances of 1.43 and 2.31 Å, respectively. Once IN2 is afforded, its azide undergoes an internal cyclization, where the N-atom directly bonded to carbon approaches the neighboring alkene C-atom to give an even more stable 2-iodo-2*H*-azirine intermediate IN3, gaining further 45.4 kcal mol^−1^ in the reaction free energy from IN2, and additional 5.7 kcal mol^−1^ for the exclusion of the formed N_2_, all despite featuring a highly strained three-membered azirine ring. This suggests this step is promoted by the cleavage of the azide N–N bond and the departure of the stable N_2_ (Figure 2), with the relevant azide N1–N2 bond already increased from 1.13 Å in IN2 to 1.72 Å in TS3. Still, the accompanying activation free energy is enhanced to Δ*G*^‡^ = 27.9 kcal mol^−1^, being the highest up until now, which, together with the high exergonicity of the IN3 formation, helps explain two different experimental observations [22]: (i) the fact that the authors isolated the intermediate IN2 in a 39% yield following the reaction at room temperature for 3 h, and (ii) an accelerated formation of IN3 accompanied by the release of N_2_ gas only after increasing the reaction temperature to 70 °C for 5 h, which are both strongly confirmed by the calculated reaction profile.

The next sequence converts the 2-iodo-2*H*-azirine intermediate IN3 into its 2-azide analogue IN5, which our calculations show is a necessary precondition that affords two nitriles (Figure 2). In other words, the iodine in IN3 should be replaced by the azide N_3_^–^ to complete the alkyne-to-nitrile conversion. However, IN3 is not prone to a simple substitution, which differs from the proposal by Yanada (Scheme 1), as all of our attempts to model such a process gave no suitable transition states or intermediates, typically resulting in the breaking of the strained azirine ring. In doing so, we considered both the SN1 reaction possibility, which did not provide a favorable cleavage of the C–I bond in IN3, and the SN2 reaction alternative with either TMSN_3_ or its even more nucleophilic azide N_3_^–^ anion, which all failed, as well as their 1,3-cycloaddition on the unsaturated N=C bond [21]. Another route to introduce azide in IN3 is the addition reaction of TMSN_3_ on the unsaturated azirine C-atom. This is justified by a notably positive charge on this C-atom in IN3 of 0.32 |e|, while, for example, the vicinal double-bonded N-atom is largely negative at –0.38 |e|. The addition of TMSN_3_ can occur either (i) on its own, or (ii) in a concerted fashion with additional NIS, thus mimicking a case during the initial R→IN1 conversion. The latter one-step approach requires 16.3 kcal mol^−1^ to form a reactive complex with IN3 and further 21.0 kcal mol^−1^ to reach the transition state describing a simultaneous cleavage of the C–I bond and the formation of a new C–N bond with TMSN_3_. This gives the cleaved I^–^ anion, which is captured by the nearby NIS to afford the molecular I_2_ bound to the anionic NS^–^. Although the reaction proceeds in a good direction and offers desired products, following the departure of I_2_, this reaction sequence is significantly endergonic (Δ*G*_R_ = 12.7 kcal mol^−1^), while, more importantly, the kinetic barrier is extensive at Δ*G*^‡^ = 37.3 kcal mol^–1^. A more feasible route (Figure 2) involves the sole approach of the N1 atom in TMSN_3_ on the mentioned azirine carbon to give the intermediate IN4, with the –SiMe_3_ moiety bonded to the rest of the azide, a reaction similar to the already described 1→IN1 conversion. The reaction proceeds through the transition state TS4, having a practically formed C–N bond (2.18 Å) and a significantly departed iodine anion I^–^ (d_C–I_ = 4.18 Å), being only 19.9 kcal mol^–1^ higher in energy than IN3, also containing a 9.9 kcal mol^–1^ contribution to bring TMSN_3_ into a reactive complex. Interestingly, TMSN_3_ addition on the unsaturated C-atom in IN3 is kinetically much more feasible than its analogous addition on the parent alkyne **1**, clearly being a result of a higher reactivity of the strained azirine. The formed IN4 with the adjacent I^–^ anion is less stable by 6.6 kcal mol^–1^ than the preceding IN3, but this process is facilitated by the positive solvation energy of the free I^–^ anion in MeCN, making its departure from the complex thermodynamically favorable (−11.5 kcal mol^−1^), thus leaving the isolated IN4 as the most stable stationary point hitherto on the profile (Δ*G*_R_ = −92.9 kcal mol^−1^).

**Scheme 3:**
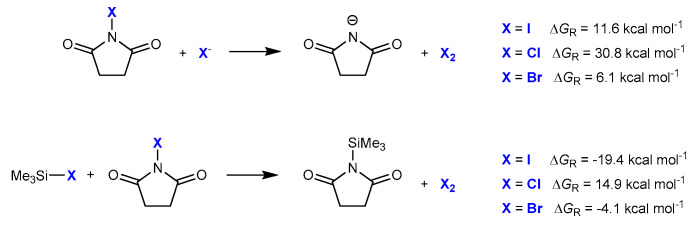
Two potential routes for the formation of molecular halogens X_2_ observed experimentally. The calculated reaction Gibbs free energies (in kcal mol^−1^) correspond to the MeCN solution.

As mentioned, the formation of IN4 offers a free I^–^ anion. Given that Yanada observed a change in the mixture color to purplish red [22], attributed to the formation of I_2_, the potential fate of the formed I^–^ can lead to two different routes, also knowing that the attached –SiMe_3_ group in IN4 must be cleaved to afford the 2-azide-2*H*-azirine analogue IN5. In one scenario, I^–^ could engage in a reaction with IN4 as a nucleophile to cleave the Si–N bond within azide. The activation energy for this is Δ*G*^‡^ = 24.6 kcal mol^−1^ and offers TMSI (Me_3_Si–I) as a byproduct, which departs the complex (−14.4 kcal mol^−1^) and leaves a highly stable IN5 as a product. However, TMSI was not reported among products under experimental conditions [22], and its likely clearance could occur through the subsequent exergonic reaction with NIS (Δ*G*_R_ = −19.4 kcal mol^−1^, Scheme 3), which produces TMS–NS adduct and I_2_, thus potentially agreeing with experiments. Overall, this reaction sequence IN4 + I^–^ + NIS → IN5 + TMS–NS + I_2_, apart from the kinetic barrier of 24.6 kcal mol^–1^, is associated with the thermodynamic change of –34.3 kcal mol^–1^, thus suggesting a viable process. Nevertheless, what turns out to be an even more feasible option starts with a favorable exclusion of the formed I^–^ from IN4 (−11.5 kcal mol^−1^) and undergoes a direct reaction with NIS, to give the expected I_2_ (Scheme 3) responsible for the observed change in the reaction mixture color [22]. While this reaction is endergonic (+11.6 kcal mol^−1^, Scheme 3), it produces a nucleophilic succinimide anion NS^–^, which cleaves the –SiMe_3_ moiety from IN4, in analogy with its role during the IN1→IN2 conversion. Bringing NS^–^ to IN4 requires 6.0 kcal mol^−1^ in free energy and an additional 3.4 kcal mol^−1^ to arrive at the transition state TS5 describing the simultaneous Si–N bond cleavage in azide (1.92 Å) and Si–N bond formation with NS^–^ (2.54 Å). The activation energy for the overall process is Δ*G*^‡^ = 21.0 kcal mol^−1^, being 3.6 kcal mol^−1^ more favorable than for the first described scenario. In addition, the latter conclusion is further promoted by the remarkably high activation energy of the TMSI + NIS → TMS–NS + I_2_ reaction, occurring in the first scenario, which exceeds 55 kcal mol^−1^, thus making it very unlikely. Lastly, following the departure of the produced TMS–NS adduct (Δ*G*_R_ = –7.4 kcal mol^−1^), the reaction gives the desired IN5 as a stable intermediate, being the most stable stationary point on the reaction profile up to that point (Δ*G*_R_ = –115.6 kcal mol^−1^).

**Figure 3:**
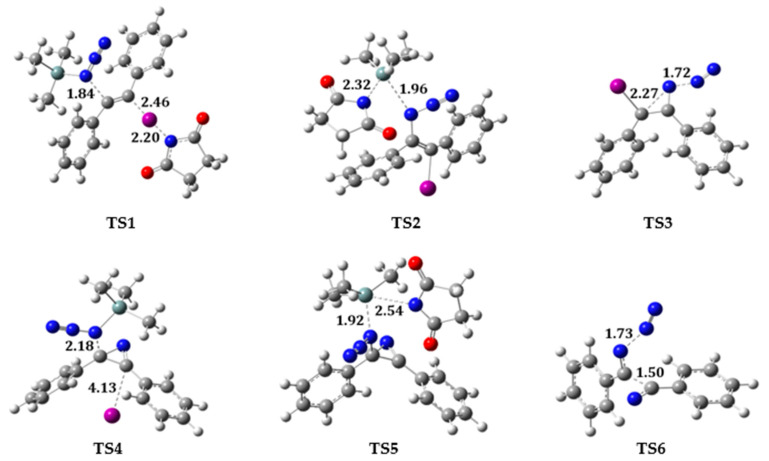
Transition state geometries during the conversion of **1** into two benzonitriles shown in Figure 2 with relevant bond distances obtained at the M06–2X/6–31+G(d) level of theory.

Once 2-azide-2*H*-azirine IN5 is formed, it undergoes intramolecular rearrangement to give two nitriles. In locating the transition state for this process, we have considered (i) the independent cleavage of any of the three bonds within azirine, (ii) the cleavage of the C–N(azide) bond, and (iii) various approaches of the attached azide onto the ring. Yet, the only feasible route was cleaving the azide N1–N2 bond, which liberates N_2_, while the reorganization of the electron density in the rest of the system affords two nitriles as final products (Figure 2). The barrier for this process is Δ*G*^‡^ = 33.5 kcal mol^−1^, being the highest on the reaction profile, thus making this reaction the rate-limiting step. The matching transition state TS6 features a practically departed N_2_ molecule (d_N1–N2_ = 1.73 Å, Figure 3), with its constituting N-atoms at a close-to-bonding distance of d_N2–N3_ = 1.11 Å, being 1.09 Å in the isolated N_2_. This directly gives two benzonitriles in a complex with N_2_, which following a very favorable exclusion of all three products (−12.9 kcal mol^−1^) gives an exceedingly exergonic total reaction free energy of Δ*G*_R_ = –191.9 kcal mol^−1^, thus clearly underlying the thermodynamic feasibility of the overall process.

In concluding this part, it is worth mentioning that, while studying several mechanistic possibilities, the obtained reaction profile (Figure 2) suggests that the conversion of **1** into two benzonitriles, oxidized by NIS and using TMSN_3_ as a nitrogen donor, is a feasible process (Δ*G*_R_ = –191.9 kcal mol^−1^) that involves six steps with the rate-limiting exclusion of N_2_ from the 2-azide-2*H*-azirine intermediate IN5 in the last step, linked with the activation barrier of Δ*G*^‡^ = 33.5 kcal mol^−1^. The identified pathway strongly agrees with several experimental observations [22] by (i) providing evidence for the formation of molecular I_2_ responsible for the change in color, and (ii) rationalizing the need to increase the reaction temperature following the first part of the process. Given the significance of the rate-limiting last step for the description of the entire process, we decided to validate the obtained activation barrier by two additional sets of calculations: (i) by correcting the total electronic energies through single-point calculations with a significantly larger and more flexible 6–311+G(2d,p) basis-set on all atoms, which gave Δ*G*^‡^ = 32.2 kcal mol^−1^, and (ii) by reoptimizing all geometries during the IN5→P conversion in the implicit solvent at the (SMD)/M06–2X/6–31+G(d) level with all parameters for pure MeCN, which gave Δ*G*^‡^ = 33.8 kcal mol^−1^. Such a close match in the obtained barriers lends some credence to the employed computational setup and led us to conclude that the presented reaction profile is likely associated with reliable kinetic and thermodynamic parameters.

### 2.3. Reaction on Diphenylacetylene Derivatives with NIS and TMSN_3_ in Acetonitrile

In order to further confirm the validity of the proposed mechanism, we considered symmetrical *p*-Me (**2**) and *p*-CN (**3**) derivatives of the parent **1**, as well as a terminal analogue **4** (Figure 1), with the idea of supporting the assumptions that electron-donating *para*-groups facilitate the reaction, as postulated by Yanada (Scheme 1) [22]. At first glance, such behavior would contradict the anticipated effect of these substituents on the stability of the C≡C bond, as, for example, the electron-donating *p*-Me group should additionally stabilize the central triple bond, thus hindering the conversion. Indeed, our data show that the bond energy in **1** is 180.7 kcal mol^−1^ in MeCN, being further increased to 181.6 kcal mol^−1^ in **2**. In contrast, the electron-withdrawing *p*-CN group in **3** reduces the strength of the triple bond to 178.8 kcal mol^−1^, leading us to conclude that the stability of the central C≡C linkage is not the only, or even the predominant effect governing the outcomes of the studied alkyne→nitrile conversion. In order to focus only on the electronic effects of the considered substituents, we have undertaken calculations while maintaining NIS and TMSN_3_ as reactants, and MeCN as the solvent (Table 1).

Table 1 reveals that the stability of all relevant stationary points in **2** and **3** is not significantly affected by the *para*-substitution with either groups, and that the matching conversion proceeds through the same mechanism. Since the rate-limiting step corresponds to the IN5→P transition and involves breaking of the azide N1–N2 bond, the latter appears independent from the electronic features of the distant *para*-moieties that are clearly too far to exert any notable electronic effect on the mentioned process, which contradicts the assumption proposed by Yanada (Scheme 1) [22]. This is also evident during the IN2→IN3 conversion, which is, surprisingly, most feasible for **3** (27.7 kcal mol^−1^ in the activation barrier), followed by **1** (27.9 kcal mol^−1^) and **2** (28.3 kcal mol^−1^). Thus, the overall activation barrier for all **1**–**3** is the same at Δ*G*^‡^ = 33.5 kcal mol^−1^, and not making any difference among systems. In contrast, thermodynamic aspects speak in favor of the *p*-Me derivative **2**, since its reaction free energy is more favorable by 0.1 kcal mol^−1^, while for **3** it is less favorable by 3.1 kcal mol^−1^ relative to **1**, in line with the observed reactivity trends [22]. Such small differences might appear confusing, yet these are not unexpected given very small variations in the reported reaction yields [22] that range from 44% for **1** to only 65% and 47% for **2** and the matching *p*-F derivative. Nevertheless, to further strengthen these arguments, let us note that all stationary points during the conversion of **2** are consistently more stable than those for **1** (Table 1), indicating a more optimal reaction for the former, whereas the same can be concluded for **3**, only in the opposite direction. Although Yanada did not consider a system symmetrically substituted with two classical and strong electron-withdrawing groups [22], our data point to a conclusion that such derivatives should be less favorable and would offer lower yields in MeCN, and their consideration will likely be associated with harsher reaction conditions.

### 2.4. Reaction on Terminal Alkynes with NIS and TMSN_3_ in Acetonitrile

According to experiments [22], terminal alkynes give nitriles in moderate yields, as, for example, **4** offered the matching nitrile in a 51% yield, being slightly reduced from 84% when its symmetrical *p*-OMe analogue was used. Our calculations show the initial approach of NIS and TMSN_3_ onto **4** is more feasible than with any of **1**–**3** (by 4.1 kcal mol^−1^ in the activation barrier) and it gives between a 9.5–11.2 kcal mol^−1^ more stable intermediate IN1. The negative charge on the terminal C(H)-atom of −0.24 |e| in **4**, as opposed to practically neutral charge of –0.06 |e| on its vicinal alkyne partner, directs the attack of the electrophilic NIS onto the former site, while the latter C-atom, bearing the *p*-methoxyphenyl unit, is approached by TMSN_3_ in a concerted way. An opposite case has a 1.7 kcal mol^−1^ higher activation barrier, thus is not considered further. Such a significant improvement in the stability of IN1 comes as a result of the reduced steric interferences between TMSN_3_ and the H-atom on the same carbon, the latter involving a much bulkier phenyl ring in **1**–**3**. The rest of the mechanism proceeds in analogy to internal alkynes, where we notice a 0.8 kcal mol^−1^ increase in the barrier for the IN2→IN3 conversion, and a 10.9 kcal mol^−1^ increase for IN3→IN4, both relative to **2**. Yet, for the rate-limiting IN5→P step, the kinetic barrier in **4** is increased by 0.1 kcal mol^−1^ relative to that for **2**, which goes in line with the mentioned tendency that terminal alkynes are less reactive [22], being further prompted by a 0.3 kcal mol^−1^ lower exergonicity for **4** (Table 1). Moreover, the analysis of geometries for the relevant TS6 offers some indication as to why the presence of an aromatic phenyl unit on both alkyne carbons facilitates this step, and why internal alkynes generally give somewhat higher yields than terminal analogues. Namely, the additional phenyl ring in **2** acts as an electron-acceptor and extracts the electron density from the azirine ring, thus making all three bonds within the ring in TS6 up to 0.1 Å longer in **2** than in **4**, which promotes this ring-opening and helps affording the final products. In conclusion, the obtained insight confirms that the studied reaction strategy can be successfully employed for both internal and terminal alkynes, with the former allowing for slightly more favorable outcomes.

### 2.5. Changing Oxidant to NCS and NBS in Acetonitrile

After revealing the precise mechanism for the alkyne→nitrile conversions, and tying the obtained results in agreement with experimental observations [22], we felt it worthwhile to study the impact of changing the oxidant from NIS into its Cl- and Br-analogues, NCS and NBS, and replacing TMSN_3_ as a nitrogen source with its sodium alternative NaN_3_ (Figure 1), with the idea of suggesting guidelines towards even more effective processes. In doing so, we maintained MeCN as a solvent and focused on the most reactive system **2** as an illustrative example (Table 1).

The approach of both NCS and NBS onto **2** is considerably more demanding than with NIS, as clearly seen in much higher activation barriers for the first step by 9.0 and 3.2 kcal mol^−1^, respectively, which already makes NCS a very poor alternative, since the initial step with NCS already has a 2.3 kcal mol^−1^ higher barrier than the whole NIS-catalyzed process. Such a trend is closely reflected in the computed N–halogen bond energies of the matching *N*-halogeno-succinimides, being 201.5, 176.6, and 94.1 kcal mol^−1^ for Cl-, Br-, and I-analogues, respectively. This shows that the N–halogen bond cleavage is most demanding for NCS, followed by NBS and NIS, which affects the feasibility of the R→IN1 reaction. Furthermore, let us recall that around 28 kcal mol^−1^ in the kinetic barrier for systems **1**–**2** (Table 1) required raising the temperature up to 70 °C to advance the reaction [22]. In contrast, the values calculated for NBS and NCS initially assume 30.0 and as much as 35.8 kcal mol^−1^, thus providing a likely reason which led Yanada and co-workers to conclude that “under the same reaction conditions, the use of NBS or NCS instead of NIS failed to give a significant amount of nitrile products” [22]. Moreover, as the reaction proceeds, the obtained insight offers additional reasons supporting such a claim. Specifically, once Cl^−^ is produced, following the IN3→IN4 conversion with NCS, its departure is exergonic at 13.8 kcal mol^−1^, being 2.3 kcal mol^−1^ more favorable than with I^−^. Yet, the subsequent reaction with NCS, to afford the nucleophilic NS^–^, is extensively endergonic (+30.8 kcal mol^−1^, Scheme 3), which, together with the approach of NS^−^ onto IN4 (+6.1 kcal mol^−1^) and further 2.4 kcal mol^−1^ to reach TS5, gives the total activation free energy of Δ*G*^‡^ = 39.3 kcal mol^−1^, being the highest on the entire reaction profile. The latter makes the IN4→IN5 conversion prevailing as the rate-limiting step of the NCS-catalyzed process, being about 6 kcal mol^−1^ higher than values calculated for NIS (Table 1). In addition, the overall reaction free energy is by 13.1 kcal mol^−1^ less exergonic with NCS than with NIS. All of this consistently makes NCS a significantly poorer option to facilitate the investigated alkyne→nitrile conversion. Alternatively, the stability of stationary points with NBS much closely reflects those with NIS (Table 1). This comes as a result of the fact that the relevant Br^–^ + NBS → NS^–^ + Br_2_ reaction is not so unfavorable as with chlorine, with the reaction free energy being even more favorable, by 5.5 kcal mol^−1^, than the analogous process for iodine (Scheme 3). This reduces the kinetic barrier for the IN4→IN5 conversion from 20.1 kcal mol^−1^ with NIS to 14.6 kcal mol^−1^ with NBS, yet the next conversion of IN5 into P does not depend on the halogenated oxidant, and thus is identical for both NIS and NBS at Δ*G*^‡^ = 33.5 kcal mol^−1^, indicating the rate-limiting step. With all this in mind, we can conclude that the practical usefulness of NBS is similar to NIS at best, likely providing a somewhat poorer choice. On the other hand, all data obtained for NCS are consistent in ruling out this alternative as a viable option.

### 2.6. Changing Nitrogen Source to NaN_3_ in Acetonitrile

The choice to consider sodium azide NaN_3_ as a potential replacement for TMSN_3_ originates in the fact that the azide unit is less strongly bonded to sodium than to the –SiMe_3_ moiety, which could overcome some difficulties linked with the cleavage of the –SiMe_3_ group from azide following the nucleophilic attack of TMSN_3_ during two steps (Figure 2). This is supported by the heterolytic Si–N bond energy of 59.6 kcal mol^−1^ calculated for TMSN_3_, being significantly reduced to 13.3 kcal mol^−1^ in NaN_3_. Nevertheless, despite such a weak bonding, in order to consistently evaluate reaction profiles with both nitrogen-donating reagents on the same footing, we have considered all processes with a full NaN_3_ molecule, although a consideration of only its significantly more nucleophilic and more reactive azide N_3_^−^ anion could also be warranted, which could likely lead to even more favorable profiles.

**Figure 4:**
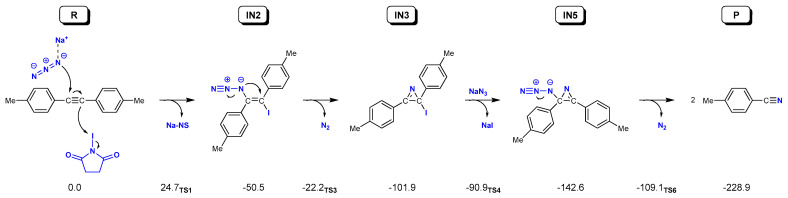
Reaction mechanism for the conversion of **2** with NIS and NaN_3_. Relative Gibbs free energies (in kcal mol^−1^) correspond to the MeCN solution. TS and IN denote transition states and intermediates, while R and P stand for reactants and products. Graphical representation of the reaction profile is given as Figure S2.

Indeed, the overall process with NaN_3_ requires two steps less than with TMSN_3_ (Figure 4), since each of the two nucleophilic additions of the former are, beside the formation of a new C(alkyne)–N1(azide) bond, linked with a significant increase in the Na–N1 bond, having practically dissociated Na^+^ cation in the produced intermediate, thus no special chemical reactions to detach the latter are needed. Specifically, the initial one-step approach of both NaN_3_ and NIS onto **2** gives a fully formed intermediate with Na^+^ and NS^–^ well separated from the rest of IN2 at d_Na–N_ = 2.37 Å and d_N–I_ = 2.53 Å, being notably elongated from d_Si–N_ = 1.87 Å and d_N–I_ = 2.37 Å when TMSN_3_ is used. Joining Na^+^ and NS^–^ to give Na–NS is favorable (–19.0 kcal mol^−1^), which, together with its positive exclusion (–32.4 kcal mol^−1^), leads to the first step of the NaN_3_-enabled reaction being extensively exergonic at Δ*G*_R_ = –50.5 kcal mol^−1^ (Figure 4). Additionally, all of this offers a 2.1 kcal mol^−1^ lower barrier to reach the first transition state TS1 with NaN_3_ than with TMSN_3_, already hinting at an improved practical usefulness of the former. The reason for that lies in the fact that the reaction with NaN_3_ proceeds through a significantly more polar transition state TS1, which is obviously favored in a solvent of such a polarity (MeCN). To support this claim, let us mention that in TS1 with TMSN_3_, the matching charges on the azide N_3_^–^ and –SiMe_3_ fragments are −0.31 and 0.69 |e|, respectively, being appreciably increased to −0.67 and 0.98 |e| on N_3_^–^ and Na^+^ with NaN_3_. Analogously, the charges on I^+^ cation and NS^–^ anion are 0.31 and −0.53 |e| with TMSN_3_, being increased to 0.33 and −0.58 |e| with NaN_3_. Therefore, a much higher nucleophilicity of NaN_3_ and its easier tendency to liberate the azide relative to TMSN_3_, together with a polar nature of TS1 facilitate the approach of NaN_3_ and NIS onto **2**. This also suggests it would be worth considering performing this reaction in even more polar solvents, provided it is not hindered by the stability of reagents or any other difficulty. This notion motivated us to inspect the effect of different solvents on the outcomes of the proposed reaction, which will be discussed later in the text.

Once IN2 is formed, it undergoes the same intramolecular rearrangement with the exclusion of N_2_ to afford IN3 (Figure 4). The next step involves a slight endergonic inclusion of another NaN_3_ (+0.9 kcal mol^−1^) and further 10.1 kcal mol^−1^ to reach the transition state TS4 for its addition onto azirine. The latter gives a free I^–^ anion, which joins with the liberated Na^+^ to give NaI through an exergonic reaction (−8.2 kcal mol^−1^). The exclusion of NaI is further exergonic (−34.9 kcal mol^−1^), offering the 2-azide-2*H*-azirine intermediate IN5 very low on the energy profile (Figure 4). The last step again sees the exclusion of N_2_, which furnishes desired nitriles. In analogy to the TMSN_3_-enabled reaction, this process is linked with the same activation barrier of Δ*G*^‡^ = 33.5 kcal mol^−1^, again being the rate-limiting step. Yet, in this case, the reaction is by as much as 36.9 kcal mol^−1^ more exergonic (Δ*G*_R_ = −228.9 kcal mol^−1^), thus indicating its thermodynamic dominance and a likely preference for the use of NaN_3_. With all this, we can conclude that the reaction with NaN_3_ proceeds through only four steps with the rate-limiting process identical to that when TMSN_3_ is used yet being significantly thermodynamically more favored. This justifies Yanada’s statement that “the use of sodium azide instead of TMSN_3_ was also found effective for the triple bond cleavage reaction” [22], knowing that the use of NaN_3_ offered comparable nitrile’s yields, but without the need to heat the mixture above room temperature to finish the reaction, as was the case with TMSN_3_. All of this firmly promotes the use of NaN_3_ in the proposed alkyne→nitrile transformation, which is recommended for future experimental evaluations.

### 2.7. Changing Solvent Polarity

Lastly, we examined the impact of the solvent polarity on the reaction outcomes. In doing so, we considered toluene and water as cases with lower and higher polarity than MeCN, taking **1** as an illustrative example, together with NIS and TMSN_3_ (Table 1). We note that the obtained insight should direct towards more optimal solvents, rather than exclusively promoting any of the employed two solvents as only alternatives.

The initial step R→IN1 already reveals notable differences among solvents. Due to the described polar nature of TS1, it is not surprising that the matching activation energy is lowest in the most polar water at Δ*G*^‡^ = 24.5 kcal mol^−1^, to be increased to 26.8 kcal mol^−1^ in MeCN, and further to 29.6 kcal mol^−1^ in toluene, in line with a decrease in the solvent polarity. This offers a very polar intermediate IN1 (Figure 2), which is again most stable in water. The next step sees the approach of anionic NS^–^ to the attached azide, which decreases the polarity of the system, thus being most feasible in toluene, requiring only 4.5 kcal mol^−1^ (8.4 kcal mol^−1^ in MeCN and 8.9 kcal mol^−1^ in H_2_O) to reach a highly stable intermediate IN2. The exclusion of the formed TMS–NS complex is more favorable by 1.6 kcal mol^−1^ in toluene than in H_2_O, thus showing equal stability of the matching IN2 in both solvents. The following rearrangement of IN2 is found within 1 kcal mol^−1^ in the kinetic barrier in all three solvents, and offers IN3, being most stable in water. The next step is most feasible in MeCN (Δ*G*^‡^ = 19.9 kcal mol^−1^, while 21.1 and 26.8 kcal mol^−1^ in H_2_O and toluene), because of the least demanding inclusion of TMSN_3_ into the reactive complex with IN3 in the former. This gives a polar intermediate IN4, again being most stable in water and least stable in toluene, which has a dominant contribution in the energy required to exclude the formed I^–^ anion from the system, being most exergonic in water (−12.9 kcal mol^−1^) and even highly endergonic in toluene (+19.2 kcal mol^−1^). In addition, the following reaction of I^–^ with NIS, which gives I_2_ and a nucleophilic NS^–^, together with the energy required to bring the latter into the reactive complex with IN4, is by far most favorable in toluene (−21.7 kcal mol^−1^), which makes the IN4→IN5 conversion an almost barrier-less process in that solvent (Δ*G*^‡^ = 2.5 kcal mol^−1^), whereas it costs 18.7 and 21.0 kcal mol^−1^ to analogously afford IN5 in H_2_O and MeCN, respectively. There, the exclusion of the formed TMS–NS is most favorable in H_2_O, thus the highest stability of IN5 in that solvent. The last conversion of the 2-azide-2*H*-azirine intermediate IN5 into the final nitriles represents the overall rate-limiting step in all three solvents. As described, the matching transition state TS6 involves the cleavage of the azide N1–N2 bond, followed by the liberation of the molecular N_2_, and the reorganization of the electron density within the azirine ring, which precedes the cleavage of several of its bonds to afford the final products P. As such, TS6 is highly polar in nature and is most favored in water, as seen in the calculated solvation free energies of TS6 being –14.0, –12.1, and –7.9 kcal mol^−1^ in H_2_O, MeCN, and toluene, respectively. As a result, the activation free energy for this step, and the whole process, is lowest in water at Δ*G*^‡^ = 32.9 kcal mol^−1^, to be increased to 33.5 kcal mol^−1^ in MeCN and further to 34.6 kcal mol^−1^ in the least polar toluene. Additionally, this goes in line with the calculated increase in the overall reaction exergonicity, which is more favorable by 1.6 kcal mol^−1^ in H_2_O than in MeCN. This is found in excellent agreement with what was observed during the Ag-catalyzed conversion of *p*-methoxy phenylacetylene with TMSN_3_ [21], which was more efficient in DMSO (81%) than in less polar DMF (58%). All of this strongly promotes solvents more polar than MeCN as better options to advance the entire alkyne→nitrile conversion, and are recommended for future experiments.

## 3. Calculation Methods

All structures were optimized by a very efficient density functional theory M06–2X approach together with the 6–31+G(d) basis set on all atoms except halogens, which were treated with a larger 6–311G(d,p) basis set downloaded from the Basis Set Exchange database [58]. Thermal corrections were extracted from the matching frequency calculations without the scaling factors, so that all reported values correspond to Gibbs free energies at room temperature. To account for the solvent effects, the obtained energies were corrected for the Gibbs solvation energies employing the SMD polarizable continuum model at the same level of theory. The choice of this computational setup was prompted by its success in reproducing kinetic and thermodynamic parameters of various organic [59,60,61], organometallic [62,63,64,65], and enzymatic reactions [66,67], being particularly accurate for relative trends among similar reactants, which is the focus here. All transition state structures were located using the scan procedure, employing both 1D and 2D scans, the latter specifically utilized to consider the possibility for concerted or one-step mechanisms. The validity of all transition states was confirmed through both the vibrational analysis and IRC calculations in both directions that identified the matching reactants and products. Atomic charges were obtained through the natural bond orbital (NBO) analysis [68]. All calculations were performed with the Gaussian 16 software [69].

## 4. Conclusions

DFT calculations considered several mechanistic possibilities and demonstrated that a conversion of diphenylacetylene **1** into two benzonitriles, oxidized by NIS and utilizing TMSN_3_ as a nitrogen donor, is a feasible process involving six reaction steps, with the rate-limiting exclusion of molecular nitrogen from the 2-azide-2*H*-azirine intermediate IN5 in the last step, associated with the activation barrier of Δ*G*^‡^ = 33.5 kcal mol^−1^ and the reaction free energy of Δ*G*_R_ = −191.9 kcal mol^−1^ in MeCN. The proposed reaction pathway strongly agrees with several experimental observations [22], by (i) providing evidence for the formation of the molecular I_2_, seen as a change in the reaction mixture color as the reaction proceeds to purplish red, and (ii) interpreting why the first part of the process occurs under room temperature, while the second part proceeds only after heating the mixture up to 70 °C. We also confirmed that *para*-electron-donating groups facilitate the reaction, but showed this is not a kinetic effect channelized through the substituent electronic contributions, but rather a thermodynamic effect seen in the highest Δ*G*_R_ = −192.0 kcal mol^−1^ for the *p*-Me derivative **2**, to be reduced to −191.9 and −188.8 kcal mol^−1^ in the unsubstituted **1** and its *p*-CN derivative **3**. The proposed mechanism also helped in suggesting that this reaction strategy can successfully be employed for both internal and terminal alkynes, and turned useful in rationalizing a slightly more favorable outcome with the former [22], as evidenced by a 0.1 kcal mol^−1^ lower activation barrier and a 0.3 kcal mol^−1^ higher exergonicity calculated for the internal alkyne **2** relative to its terminal counterpart **4**.

As an extension of Yanada’s work, we revealed that other halogen-derived succinimides, NCS and NBS, provide poor options to replace NIS as an oxidant, most dominantly because of notably higher N–halogen bond dissociation energies, but also because of unfavorable side reactions X^−^ + NXS → X_2_ + NS^–^ that precede the fifth reaction step, being the least favorable for Cl^–^ (Δ*G*_R_ = +30.8 kcal mol^−1^), yielding the enhanced activation free energy of Δ*G*^‡^ = 39.3 kcal mol^−1^. In contrast, replacing nitrogen-donating TMSN_3_ with NaN_3_ reduced the number of the reaction steps to four and increased the overall reaction exergonicity to Δ*G*_R_ = –228.9 kcal mol^−1^, promoting the latter as a viable alternative to facilitate the process, and explaining comparable product yields when the reaction is performed entirely at room temperature with that nitrogen-donating system.

Lastly, we showed that the rate-limiting rearrangement of the 2-azide-2*H*-azirine intermediate IN5 into the final products proceeds through a polar transition state, and is, as such, additionally stabilized when solvents of higher polarity than MeCN are used. Water, as an illustrative example, reduces the activation free energy by 0.6 kcal mol^−1^ and increases the reaction free energy by –1.6 kcal mol^−1^ relative to MeCN, thus consistently suggesting an increase in the solvent polarity as another useful route to advance the investigated reaction strategy towards even more efficient processes.

## Figures and Tables

**Table 1:** Relative Gibbs free energies of stationary points during the alkyne→nitrile conversion shown in Figure 2 (in kcal mol^−1^).

Solvent	Acetonitrile							Toluene	Water
Oxidant	NIS	NIS	NIS	NIS	NCS	NBS	NIS	NIS	NIS
Nitrogen Source	TMSN_3_	TMSN_3_	TMSN_3_	TMSN_3_	TMSN_3_	TMSN_3_	NaN_3_	TMSN_3_	TMSN_3_
Alkyne	1	2	3	4	2	2	2	1	1
Stationary Point									
R	0.0	0.0	0.0	0.0	0.0	0.0	0.0	0.0	0.0
TS1	26.8	26.8	26.8	22.7	35.8	30.0	24.7	29.6	24.5
IN1	13.7	12.9	14.6	3.4	–5.5	1.7		16.1	9.7
TS2	22.1	20.7	22.2	8.4	1.0	8.3		20.7	18.6
IN2	−36.9	–37.6	–37.2	–43.4	–57.3	–50.9	–50.5	–38.5	–38.5
TS3	−9.0	–9.3	–9.4	–14.3	–28.5	–23.3	–22.2	–10.6	–11.2
IN3	–88.0	–88.9	–87.2	–94.9	–110.8	–104.7	–101.9	–89.2	–93.9
TS4	–68.1	–71.4	–62.4	–66.4	–78.5	–73.8	–90.9	–62.4	–72.7
IN4	–92.9	–93.8	–92.9	–88.1	–103.4	–91.4		–77.4	–98.9
TS5	–71.9	–73.7	–72.5	–69.4	–64.0	–76.8		–74.9	–80.2
IN5	–115.6	–116.3	–116.8	–112.9	–106.6	–119.4	–142.6	–118.6	–124.0
TS6	–82.1	–82.8	–83.3	–79.2	–73.1	–85.9	–109.1	–84.0	–91.1
P	–191.9	–192.0	–188.8	–191.7	–178.9	–192.5	–228.9	–193.9	–193.5
Rate-limiting step	IN5→P	IN5→P	IN5→P	IN5→P	IN4→IN5	IN5→P	IN5→P	IN5→P	IN5→P
Activation barrier Δ*G*^‡^	33.5	33.5	33.5	33.6	39.3	33.5	33.5	34.6	32.9

## Data Availability

Data is contained within the article or Supplementary Material.

## Supplementary Materials

The following are available online at https://www.mdpi.com/1422-0067/22/6/3193/s1: Cartesian coordinates, total molecular energies, thermal corrections and the number of imaginary frequencies for all computed systems; Figure S1: Graphical representation of the reaction profile for the conversion of diphenylacetylene **1** to benzonitrile with NIS as an oxidant and TMSN_3_ as a nitrogen source in MeCN.; Figure S2: Graphical representation of the reaction profile for the conversion of **2** to *p*-Me-benzonitrile with NIS as an oxidant and NaN_3_ as a nitrogen source in MeCN.
