# Supplementary material for: Direct Metal-Free Transformation of Alkynes to Nitriles: Computational Evidence for the Precise Reaction Mechanism
Source: Int J Mol Sci. 2021 Mar 21;22(6):3193. doi: 10.3390/ijms22063193 (PMC8004279; doi:10.3390/ijms22063193)
Supplement: Supplementary file 1 [file ijms-22-03193-s001.pdf]

## Supporting Information

# Direct Metal-Free Transformation of Alkynes to Nitriles: Computational Evidence for the Precise Reaction Mechanism

**Lucija Hok [1] and Robert Vianello [1,*]**

[1] Division of Organic Chemistry and Biochemistry, Ruđer Bošković Institute, Bijenička cesta 54, Zagreb, Croatia; lucija.hok@irb.hr (LH), robert.vianello@irb.hr (RV)
* Correspondence: robert.vianello@irb.hr

## Table of Contents

| CONTENT | PAGES |
| --- | --- |
| **Figure S1.** Graphical representation of the reaction profile for the conversion of diphenylacetylene **1** to benzonitrile with **NIS** as an oxidant and **$TMSN_3$** as a nitrogen source in the MeCN solution. | S2 |
| **Figure S2.** Graphical representation of the reaction profile for the conversion of **2** to *para*-Me-benzonitrile with **NIS** as an oxidant and **$NaN_3$** as a nitrogen source in the MeCN solution. | S3 |
| Cartesian coordinates, total molecular energies, thermal corrections to Gibbs free and the number of imaginary frequencies for all computed systems. | S4–S124 |

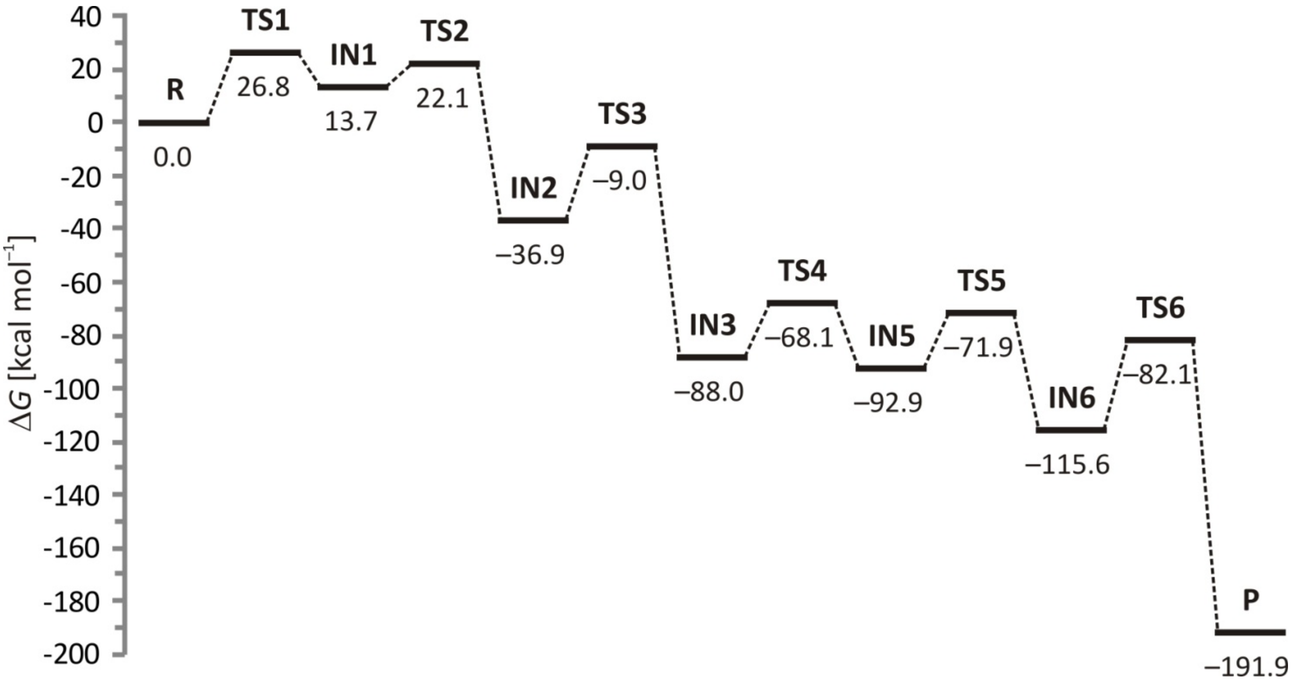


**Figure S1.** Graphical representation of the reaction profile for the conversion of diphenylacetylene **1** to benzonitrile with **NIS** as an oxidant and **$TMSN_3$** as a nitrogen source. Relative Gibbs free energies (in kcal $mol^{-1}$) correspond to the MeCN solution. **TS** and **IN** denote transition states and intermediates, while **R** and **P** stand for reactants and products. The matching chemical structures are given in Figure 2 in the main text.

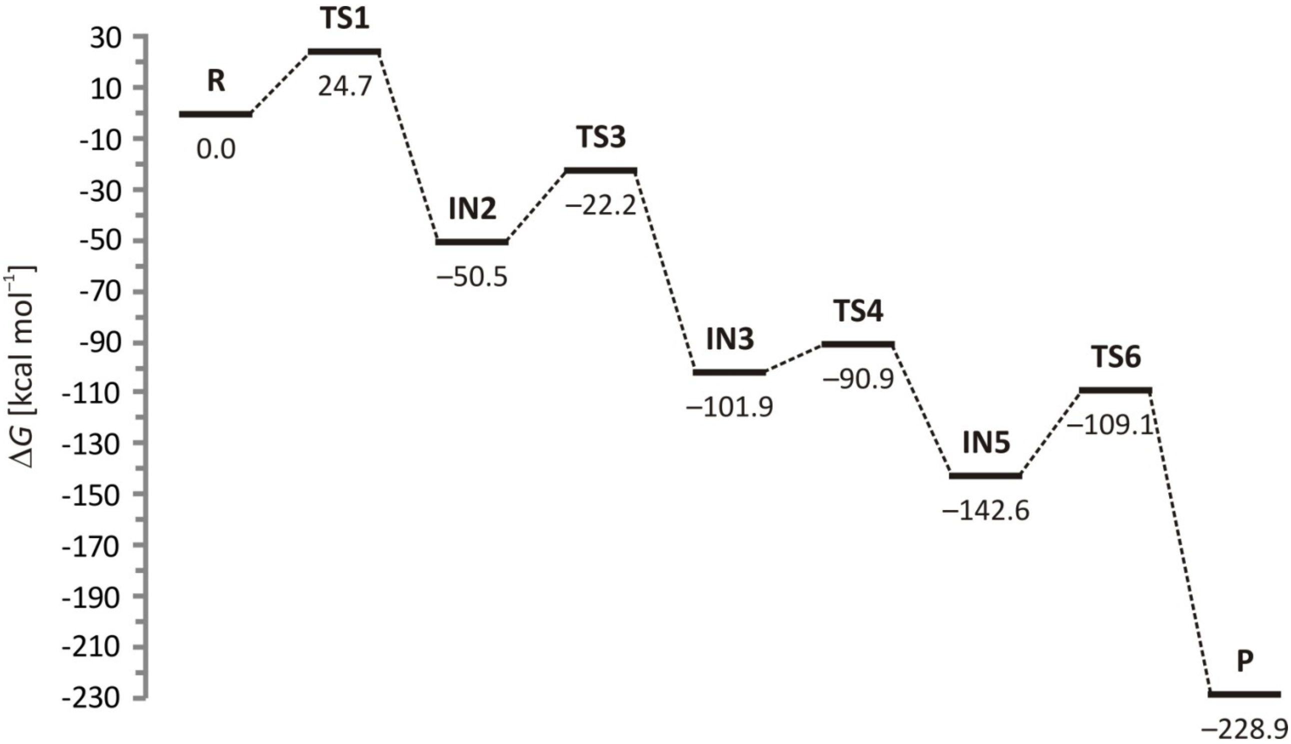


**Figure S2.** Graphical representation of the reaction profile for the conversion of **2** to *para*-Me-benzonitrile with **NIS** as an oxidant and $\mathbf{NaN_3}$ as a nitrogen source. Relative Gibbs free energies (in kcal $mol^{-1}$) correspond to the MeCN solution. **TS** and **IN** denote transition states and intermediates, while **R** and **P** stand for reactants and products. The matching chemical structures are given in Figure 4 in the main text.

| System: | isolated **1** |
|---|---|
| Oxidant: | |
| Nitrogen-donor: | |
| Stationary point: | |
| M06–2X/6–31+G(d) energy (in a.u.): | -539.239351949 |
| Thermal correction to Gibbs Free Energy (in a.u.): | 0.153991 |
| Number of imaginary frequencies: | 0 |

**CARTESIAN COORDINATES**

| Center Number | Atomic Number | Atomic Type | Coordinates (Angstroms) X | Y | Z |
|---|---|---|---|---|---|
| 1 | 6 | 0 | 0.606430 | -0.000338 | 0.000520 |
| 2 | 6 | 0 | -0.606432 | -0.000107 | 0.000140 |
| 3 | 6 | 0 | 2.039148 | -0.000144 | 0.000299 |
| 4 | 6 | 0 | 2.748731 | -1.210928 | -0.021133 |
| 5 | 6 | 0 | 2.748450 | 1.210823 | 0.021475 |
| 6 | 6 | 0 | 4.139608 | -1.206723 | -0.021429 |
| 7 | 1 | 0 | 2.198451 | -2.146620 | -0.037702 |
| 8 | 6 | 0 | 4.139336 | 1.206946 | 0.021108 |
| 9 | 1 | 0 | 2.197967 | 2.146387 | 0.038345 |
| 10 | 6 | 0 | 4.838720 | 0.000198 | -0.000342 |
| 11 | 1 | 0 | 4.679921 | -2.148594 | -0.038310 |
| 12 | 1 | 0 | 4.679434 | 2.148947 | 0.037749 |
| 13 | 1 | 0 | 5.924642 | 0.000319 | -0.000608 |
| 14 | 6 | 0 | -2.039151 | -0.000056 | -0.000027 |
| 15 | 6 | 0 | -2.748665 | -1.210896 | 0.021215 |
| 16 | 6 | 0 | -2.748519 | 1.210853 | -0.021377 |
| 17 | 6 | 0 | -4.139544 | -1.206764 | 0.021176 |
| 18 | 1 | 0 | -2.198349 | -2.146561 | 0.037920 |
| 19 | 6 | 0 | -4.139397 | 1.206904 | -0.021377 |
| 20 | 1 | 0 | -2.198083 | 2.146446 | -0.038078 |
| 21 | 6 | 0 | -4.838716 | 0.000111 | -0.000086 |
| 22 | 1 | 0 | -4.679808 | -2.148665 | 0.037944 |
| 23 | 1 | 0 | -4.679542 | 2.148872 | -0.038158 |
| 24 | 1 | 0 | -5.924636 | 0.000189 | -0.000062 |

| System: | isolated **2** |
|---|---|
| Oxidant: | |
| Nitrogen-donor: | |
| Stationary point: | |
| M06–2X/6–31+G(d) energy (in a.u.): | -617.833944709 |
| Thermal correction to Gibbs Free Energy (in a.u.): | 0.203067 |
| Number of imaginary frequencies: | 0 |

**CARTESIAN COORDINATES**

| Center Number | Atomic Number | Atomic Type | Coordinates (Angstroms) X | Y | Z |
|---|---|---|---|---|---|
| 1 | 6 | 0 | 4.143010 | -1.198778 | 0.009939 |
| 2 | 6 | 0 | 2.753706 | -1.206254 | 0.005632 |
| 3 | 6 | 0 | 2.038489 | 0.001155 | 0.002987 |
| 4 | 6 | 0 | 2.752696 | 1.207928 | 0.005857 |
| 5 | 6 | 0 | 4.143094 | 1.201056 | 0.010239 |
| 6 | 6 | 0 | 4.861601 | 0.001771 | 0.009497 |
| 7 | 1 | 0 | 4.682678 | -2.143318 | 0.015392 |
| 8 | 1 | 0 | 2.208240 | -2.145211 | 0.007861 |
| 9 | 1 | 0 | 2.207238 | 2.146876 | 0.008253 |
| 10 | 1 | 0 | 4.681890 | 2.145874 | 0.016030 |
| 11 | 6 | 0 | -2.038487 | -0.001079 | -0.002746 |
| 12 | 6 | 0 | -2.753770 | 1.206297 | -0.005773 |
| 13 | 6 | 0 | -2.752629 | -1.207885 | -0.005391 |
| 14 | 6 | 0 | -4.143069 | 1.198745 | -0.010267 |
| 15 | 1 | 0 | -2.208356 | 2.145283 | -0.008174 |
| 16 | 6 | 0 | -4.143032 | -1.201088 | -0.009964 |
| 17 | 1 | 0 | -2.207124 | -2.146806 | -0.007477 |
| 18 | 6 | 0 | -4.861602 | -0.001845 | -0.009640 |
| 19 | 1 | 0 | -4.682783 | 2.143259 | -0.016041 |
| 20 | 1 | 0 | -4.681771 | -2.145938 | -0.015599 |
| 21 | 6 | 0 | 0.606493 | 0.000503 | 0.001380 |
| 22 | 6 | 0 | -0.606492 | -0.000370 | -0.001045 |
| 23 | 6 | 0 | -6.368831 | 0.001854 | 0.016464 |
| 24 | 1 | 0 | -6.740439 | 0.124486 | 1.040517 |
| 25 | 1 | 0 | -6.773865 | 0.824183 | -0.581071 |
| 26 | 1 | 0 | -6.774822 | -0.935781 | -0.373485 |
| 27 | 6 | 0 | 6.368826 | -0.001964 | -0.016928 |
| 28 | 1 | 0 | 6.774949 | 0.935124 | 0.374208 |
| 29 | 1 | 0 | 6.773952 | -0.825118 | 0.579396 |
| 30 | 1 | 0 | 6.740185 | -0.123198 | -1.041242 |

| System: | isolated **3** |
|---|---|
| Oxidant: | |
| Nitrogen-donor: | |
| Stationary point: | |
| M06–2X/6–31+G(d) energy (in a.u.): | -723.671923818 |
| Thermal correction to Gibbs Free Energy (in a.u.): | 0.145910 |
| Number of imaginary frequencies: | 0 |

**CARTESIAN COORDINATES**

| Center Number | Atomic Number | Atomic Type | Coordinates (Angstroms) X | Y | Z |
|---|---|---|---|---|---|
| 1 | 6 | 0 | 4.133249 | 0.890725 | -0.825666 |
| 2 | 6 | 0 | 2.745640 | 0.889519 | -0.824301 |
| 3 | 6 | 0 | 2.040023 | 0.000011 | 0.000020 |
| 4 | 6 | 0 | 2.745653 | -0.889521 | 0.824307 |
| 5 | 6 | 0 | 4.133260 | -0.890724 | 0.825654 |
| 6 | 6 | 0 | 4.829112 | 0.000011 | -0.000001 |
| 7 | 1 | 0 | 4.684255 | 1.576295 | -1.461368 |
| 8 | 1 | 0 | 2.195904 | 1.575611 | -1.460471 |
| 9 | 1 | 0 | 2.195924 | -1.575626 | 1.460469 |
| 10 | 1 | 0 | 4.684279 | -1.576299 | 1.461340 |
| 11 | 6 | 0 | -2.040030 | -0.000010 | 0.000009 |
| 12 | 6 | 0 | -2.745652 | -0.889523 | -0.824303 |
| 13 | 6 | 0 | -2.745643 | 0.889514 | 0.824313 |
| 14 | 6 | 0 | -4.133259 | -0.890738 | -0.825639 |
| 15 | 1 | 0 | -2.195914 | -1.575629 | -1.460457 |
| 16 | 6 | 0 | -4.133252 | 0.890747 | 0.825648 |
| 17 | 1 | 0 | -2.195902 | 1.575617 | 1.460467 |
| 18 | 6 | 0 | -4.829111 | 0.000008 | 0.000007 |
| 19 | 1 | 0 | -4.684283 | -1.576312 | -1.461322 |
| 20 | 1 | 0 | -4.684267 | 1.576330 | 1.461329 |
| 21 | 6 | 0 | 0.605805 | -0.000012 | 0.000009 |
| 22 | 6 | 0 | -0.605795 | -0.000018 | 0.000004 |
| 23 | 6 | 0 | -6.269153 | 0.000018 | 0.000002 |
| 24 | 6 | 0 | 6.269153 | 0.000008 | -0.000013 |
| 25 | 7 | 0 | 7.426680 | -0.000004 | -0.000010 |
| 26 | 7 | 0 | -7.426679 | -0.000008 | -0.000031 |

| System: | isolated **4** |
|---|---|
| Oxidant: | |
| Nitrogen-donor: | |
| Stationary point: | |
| M06–2X/6–31+G(d) energy (in a.u.): | -422.747872085 |
| Thermal correction to Gibbs Free Energy (in a.u.): | 0.110335 |
| Number of imaginary frequencies: | 0 |

**CARTESIAN COORDINATES**

| Center Number | Atomic Number | Atomic Type | Coordinates (Angstroms) X | Y | Z |
|---|---|---|---|---|---|
| 1 | 6 | 0 | -0.698907 | -1.003802 | -0.000193 |
| 2 | 6 | 0 | 0.688224 | -1.139729 | -0.000159 |
| 3 | 6 | 0 | 1.523687 | -0.019382 | -0.000019 |
| 4 | 6 | 0 | 0.937645 | 1.258956 | -0.000081 |
| 5 | 6 | 0 | -0.438099 | 1.404343 | -0.000124 |
| 6 | 6 | 0 | -1.265539 | 0.273381 | -0.000098 |
| 7 | 1 | 0 | -1.318414 | -1.893041 | -0.000391 |
| 8 | 1 | 0 | 1.130537 | -2.131227 | -0.000196 |
| 9 | 1 | 0 | 1.577499 | 2.135923 | -0.000099 |
| 10 | 1 | 0 | -0.902837 | 2.385057 | -0.000135 |
| 11 | 6 | 0 | 2.950700 | -0.168460 | 0.000085 |
| 12 | 6 | 0 | 4.153068 | -0.290713 | 0.000204 |
| 13 | 8 | 0 | -2.600400 | 0.518332 | 0.000154 |
| 14 | 6 | 0 | -3.475756 | -0.590405 | 0.000176 |
| 15 | 1 | 0 | -4.483768 | -0.176761 | 0.000651 |
| 16 | 1 | 0 | -3.332976 | -1.206109 | -0.895929 |
| 17 | 1 | 0 | -3.332421 | -1.206699 | 0.895828 |
| 18 | 1 | 0 | 5.215440 | -0.398932 | 0.000289 |

| System: | isolated **NIS** |
|---|---|
| Oxidant: | |
| Nitrogen-donor: | |
| Stationary point: | |
| M06–2X/6–31+G(d) energy (in a.u.): | -7279.82166411 |
| Thermal correction to Gibbs Free Energy (in a.u.): | 0.047763 |
| Number of imaginary frequencies: | 0 |

**CARTESIAN COORDINATES**

| Center Number | Atomic Number | Atomic Type | Coordinates (Angstroms) X | Y | Z |
|---|---|---|---|---|---|
| 1 | 53 | 0 | -1.497460 | -0.000062 | -0.000059 |
| 2 | 6 | 0 | 1.304219 | 1.175696 | 0.000652 |
| 3 | 6 | 0 | 1.304558 | -1.175638 | 0.000755 |
| 4 | 6 | 0 | 2.770798 | 0.766613 | -0.001622 |
| 5 | 6 | 0 | 2.771026 | -0.766095 | 0.001107 |
| 6 | 1 | 0 | 3.244389 | 1.202009 | -0.885147 |
| 7 | 1 | 0 | 3.248722 | 1.205724 | 0.877658 |
| 8 | 1 | 0 | 3.248067 | -1.205124 | -0.878696 |
| 9 | 1 | 0 | 3.245735 | -1.201342 | 0.884106 |
| 10 | 7 | 0 | 0.545287 | -0.000101 | 0.000945 |
| 11 | 8 | 0 | 0.853965 | -2.291086 | -0.001027 |
| 12 | 8 | 0 | 0.853264 | 2.290995 | 0.000184 |

| System: | isolated **NCS** |
|---|---|
| Oxidant: | |
| Nitrogen-donor: | |
| Stationary point: | |
| M06–2X/6–31+G(d) energy (in a.u.): | -820.080119376 |
| Thermal correction to Gibbs Free Energy (in a.u.): | 0.050594 |
| Number of imaginary frequencies: | 0 |

**CARTESIAN COORDINATES**

| Center Number | Atomic Number | Atomic Type | Coordinates (Angstroms) X | Y | Z |
|---|---|---|---|---|---|
| 1 | 6 | 0 | 1.883423 | 0.767488 | -0.000156 |
| 2 | 6 | 0 | 1.883423 | -0.767488 | 0.000157 |
| 3 | 6 | 0 | 0.421488 | -1.185171 | 0.000048 |
| 4 | 6 | 0 | 0.421488 | 1.185171 | -0.000052 |
| 5 | 1 | 0 | 2.361557 | 1.202049 | 0.881148 |
| 6 | 7 | 0 | -0.327433 | 0.000000 | -0.000001 |
| 7 | 8 | 0 | -0.040100 | 2.293371 | -0.000033 |
| 8 | 8 | 0 | -0.040100 | -2.293371 | 0.000034 |
| 9 | 1 | 0 | 2.361249 | 1.201634 | -0.881837 |
| 10 | 1 | 0 | 2.361246 | -1.201634 | 0.881839 |
| 11 | 1 | 0 | 2.361560 | -1.202049 | -0.881145 |
| 12 | 17 | 0 | -2.010054 | 0.000000 | 0.000001 |

| System: | isolated **NBS** |
|---|---|
| Oxidant: | |
| Nitrogen-donor: | |
| Stationary point: | |
| M06–2X/6–31+G(d) energy (in a.u.): | -2934.06052282 |
| Thermal correction to Gibbs Free Energy (in a.u.): | 0.048897 |
| Number of imaginary frequencies: | 0 |

**CARTESIAN COORDINATES**

| Center Number | Atomic Number | Atomic Type | Coordinates (Angstroms) X | Y | Z |
|---|---|---|---|---|---|
| 1 | 6 | 0 | -2.367959 | -0.767100 | -0.000149 |
| 2 | 6 | 0 | -2.367959 | 0.767100 | 0.000152 |
| 3 | 6 | 0 | -0.904551 | 1.182489 | 0.000059 |
| 4 | 6 | 0 | -0.904551 | -1.182489 | -0.000055 |
| 5 | 1 | 0 | -2.845137 | -1.202794 | 0.881159 |
| 6 | 7 | 0 | -0.152535 | 0.000000 | 0.000001 |
| 7 | 8 | 0 | -0.448818 | -2.293855 | -0.000061 |
| 8 | 8 | 0 | -0.448818 | 2.293855 | 0.000061 |
| 9 | 1 | 0 | -2.844858 | -1.202399 | -0.881808 |
| 10 | 1 | 0 | -2.844858 | 1.202399 | 0.881811 |
| 11 | 1 | 0 | -2.845137 | 1.202794 | -0.881156 |
| 12 | 35 | 0 | 1.682827 | 0.000000 | -0.000002 |

| System: | isolated **$TMSN_3$** |
|---|---|
| Oxidant: | |
| Nitrogen-donor: | |
| Stationary point: | |
| M06–2X/6–31+G(d) energy (in a.u.): | -573.324585061 |
| Thermal correction to Gibbs Free Energy (in a.u.): | 0.088855 |
| Number of imaginary frequencies: | 0 |

**CARTESIAN COORDINATES**

| Center Number | Atomic Number | Atomic Type | Coordinates (Angstroms) X | Y | Z |
|---|---|---|---|---|---|
| 1 | 14 | 0 | 0.658261 | 0.000001 | 0.024662 |
| 2 | 6 | 0 | 1.597093 | 1.542665 | -0.462801 |
| 3 | 1 | 0 | 1.767887 | 1.563817 | -1.544247 |
| 4 | 1 | 0 | 1.038960 | 2.445080 | -0.192953 |
| 5 | 1 | 0 | 2.572408 | 1.583067 | 0.035385 |
| 6 | 6 | 0 | 1.596961 | -1.542792 | -0.462651 |
| 7 | 1 | 0 | 2.572258 | -1.583257 | 0.035567 |
| 8 | 1 | 0 | 1.038731 | -2.445135 | -0.192762 |
| 9 | 1 | 0 | 1.767786 | -1.564032 | -1.544091 |
| 10 | 6 | 0 | 0.211632 | 0.000104 | 1.847144 |
| 11 | 1 | 0 | -0.373006 | 0.886059 | 2.118969 |
| 12 | 1 | 0 | -0.373082 | -0.885782 | 2.119038 |
| 13 | 1 | 0 | 1.118787 | 0.000087 | 2.461858 |
| 14 | 7 | 0 | -0.852581 | 0.000024 | -0.917443 |
| 15 | 7 | 0 | -1.958951 | 0.000015 | -0.401400 |
| 16 | 7 | 0 | -3.014253 | -0.000006 | 0.008532 |

| System: | isolated **$NaN_3$** |
|---|---|
| Oxidant: | |
| Nitrogen-donor: | |
| Stationary point: | |
| M06–2X/6–31+G(d) energy (in a.u.): | -326.418631673 |
| Thermal correction to Gibbs Free Energy (in a.u.): | -0.009621 |
| Number of imaginary frequencies: | 0 |

**CARTESIAN COORDINATES**

| Center Number | Atomic Number | Atomic Type | Coordinates (Angstroms) X | Y | Z |
|---|---|---|---|---|---|
| 1 | 7 | 0 | -0.066664 | 0.003279 | 0.000000 |
| 2 | 7 | 0 | 1.128754 | 0.000466 | -0.000000 |
| 3 | 7 | 0 | 2.279816 | -0.001877 | 0.000000 |
| 4 | 11 | 0 | -2.126667 | -0.001188 | 0.000000 |

| System: | isolated **TMS–NS** |
|---|---|
| Oxidant: | |
| Nitrogen-donor: | |
| Stationary point: | |
| M06–2X/6–31+G(d) energy (in a.u.): | -769.133940997 |
| Thermal correction to Gibbs Free Energy (in a.u.): | 0.156109 |
| Number of imaginary frequencies: | 0 |

**CARTESIAN COORDINATES**

| Center Number | Atomic Number | Atomic Type | Coordinates (Angstroms) X | Y | Z |
|---|---|---|---|---|---|
| 1 | 14 | 0 | 1.412342 | 0.033506 | 0.000000 |
| 2 | 6 | 0 | 2.020245 | 1.798602 | -0.000028 |
| 3 | 1 | 0 | 1.677886 | 2.347919 | -0.881129 |
| 4 | 1 | 0 | 1.677919 | 2.347934 | 0.881078 |
| 5 | 1 | 0 | 3.117243 | 1.788587 | -0.000048 |
| 6 | 6 | 0 | 1.934082 | -0.870490 | -1.551987 |
| 7 | 1 | 0 | 3.027459 | -0.919614 | -1.612273 |
| 8 | 1 | 0 | 1.542828 | -1.891962 | -1.557022 |
| 9 | 1 | 0 | 1.573545 | -0.353707 | -2.448000 |
| 10 | 6 | 0 | 1.934069 | -0.870446 | 1.552018 |
| 11 | 1 | 0 | 1.573528 | -0.353635 | 2.448013 |
| 12 | 1 | 0 | 1.542807 | -1.891914 | 1.557078 |
| 13 | 1 | 0 | 3.027445 | -0.919578 | 1.612315 |
| 14 | 6 | 0 | -1.119188 | -1.152494 | -0.000002 |
| 15 | 6 | 0 | -1.268095 | 1.150236 | -0.000000 |
| 16 | 6 | 0 | -2.613827 | -0.864558 | -0.000002 |
| 17 | 6 | 0 | -2.712528 | 0.659096 | -0.000007 |
| 18 | 1 | 0 | -3.056890 | -1.336660 | -0.880834 |
| 19 | 1 | 0 | -3.056888 | -1.336653 | 0.880834 |
| 20 | 1 | 0 | -3.213827 | 1.069229 | -0.880570 |
| 21 | 1 | 0 | -3.213839 | 1.069236 | 0.880545 |
| 22 | 7 | 0 | -0.408923 | 0.047633 | -0.000004 |
| 23 | 8 | 0 | -0.920695 | 2.307783 | 0.000008 |
| 24 | 8 | 0 | -0.601568 | -2.247954 | 0.000003 |

| System: | isolated **Na–NS** |
|---|---|
| Oxidant: | |
| Nitrogen-donor: | |
| Stationary point: | |
| M06–2X/6–31+G(d) energy (in a.u.): | -522.244053329 |
| Thermal correction to Gibbs Free Energy (in a.u.): | 0.049515 |
| Number of imaginary frequencies: | 0 |

**CARTESIAN COORDINATES**

| Center Number | Atomic Number | Atomic Type | Coordinates (Angstroms) X | Y | Z |
|---|---|---|---|---|---|
| 1 | 6 | 0 | -0.505234 | 0.726916 | 0.000003 |
| 2 | 6 | 0 | 1.204575 | -0.709023 | 0.000002 |
| 3 | 6 | 0 | 0.703643 | 1.653926 | 0.000008 |
| 4 | 6 | 0 | 1.877352 | 0.675558 | -0.000014 |
| 5 | 1 | 0 | 0.658582 | 2.301115 | -0.880796 |
| 6 | 1 | 0 | 0.658596 | 2.301082 | 0.880837 |
| 7 | 1 | 0 | 2.519804 | 0.755929 | -0.881026 |
| 8 | 1 | 0 | 2.519847 | 0.755935 | 0.880965 |
| 9 | 7 | 0 | -0.171255 | -0.576844 | 0.000005 |
| 10 | 8 | 0 | 1.812615 | -1.760998 | 0.000006 |
| 11 | 8 | 0 | -1.692141 | 1.124098 | 0.000000 |
| 12 | 11 | 0 | -2.345804 | -1.005930 | -0.000005 |

| System: | isolated **NS anion** |
|---|---|
| Oxidant: | |
| Nitrogen-donor: | |
| Stationary point: | |
| M06–2X/6–31+G(d) energy (in a.u.): | -359.978917857 |
| Thermal correction to Gibbs Free Energy (in a.u.): | 0.049042 |
| Number of imaginary frequencies: | 0 |

**CARTESIAN COORDINATES**

| Center Number | Atomic Number | Atomic Type | Coordinates (Angstroms) X | Y | Z |
|---|---|---|---|---|---|
| 1 | 6 | 0 | 0.759869 | -1.224558 | -0.000173 |
| 2 | 6 | 0 | -0.759869 | -1.224558 | 0.000174 |
| 3 | 6 | 0 | -1.108470 | 0.286181 | -0.000040 |
| 4 | 6 | 0 | 1.108470 | 0.286181 | 0.000036 |
| 5 | 1 | 0 | 1.212266 | -1.691073 | 0.881789 |
| 6 | 7 | 0 | 0.000000 | 1.066819 | -0.000001 |
| 7 | 8 | 0 | 2.283508 | 0.659765 | 0.000031 |
| 8 | 8 | 0 | -2.283508 | 0.659765 | -0.000029 |
| 9 | 1 | 0 | 1.211769 | -1.690651 | -0.882620 |
| 10 | 1 | 0 | -1.211769 | -1.690648 | 0.882623 |
| 11 | 1 | 0 | -1.212266 | -1.691076 | -0.881787 |

| System: | isolated **1-product** (benzonitrile) |
|---|---|
| Oxidant: | |
| Nitrogen-donor: | |
| Stationary point: | |
| M06–2X/6–31+G(d) energy (in a.u.): | -324.362339233 |
| Thermal correction to Gibbs Free Energy (in a.u.): | 0.069865 |
| Number of imaginary frequencies: | 0 |

**CARTESIAN COORDINATES**

| Center Number | Atomic Number | Atomic Type | Coordinates (Angstroms) X | Y | Z |
|---|---|---|---|---|---|
| 1 | 6 | 0 | 2.044333 | -0.000166 | -0.000081 |
| 2 | 6 | 0 | 0.603327 | -0.000185 | 0.000099 |
| 3 | 6 | 0 | -0.090306 | 1.215467 | 0.000120 |
| 4 | 6 | 0 | -0.090445 | -1.215597 | 0.000107 |
| 5 | 6 | 0 | -1.481227 | 1.209585 | -0.000042 |
| 6 | 1 | 0 | 0.463768 | 2.148744 | 0.000173 |
| 7 | 6 | 0 | -1.481543 | -1.209345 | -0.000037 |
| 8 | 1 | 0 | 0.463151 | -2.149142 | 0.000195 |
| 9 | 6 | 0 | -2.175883 | 0.000105 | -0.000045 |
| 10 | 1 | 0 | -2.023749 | 2.149820 | -0.000153 |
| 11 | 1 | 0 | -2.024053 | -2.149600 | -0.000146 |
| 12 | 1 | 0 | -3.261808 | 0.000263 | -0.000188 |
| 13 | 7 | 0 | 3.201879 | 0.000105 | -0.000086 |

| System: | isolated **2-product** (*p*-tolunitrile) |
|---|---|
| Oxidant: | |
| Nitrogen-donor: | |
| Stationary point: | |
| M06–2X/6–31+G(d) energy (in a.u.): | -363.660332725 |
| Thermal correction to Gibbs Free Energy (in a.u.): | 0.094678 |
| Number of imaginary frequencies: | 0 |

**CARTESIAN COORDINATES**

| Center Number | Atomic Number | Atomic Type | Coordinates (Angstroms) X | Y | Z |
|---|---|---|---|---|---|
| 1 | 6 | 0 | 2.552748 | -0.000702 | 0.004487 |
| 2 | 6 | 0 | 1.112846 | 0.000319 | 0.000613 |
| 3 | 6 | 0 | 0.412532 | -1.211431 | -0.003159 |
| 4 | 6 | 0 | 0.414431 | 1.212042 | -0.003103 |
| 5 | 6 | 0 | -0.976596 | -1.201314 | -0.009570 |
| 6 | 1 | 0 | 0.960531 | -2.148448 | -0.004271 |
| 7 | 6 | 0 | -0.975739 | 1.203682 | -0.009564 |
| 8 | 1 | 0 | 0.963196 | 2.148604 | -0.004135 |
| 9 | 6 | 0 | -1.691215 | 0.002054 | -0.010080 |
| 10 | 1 | 0 | -1.518077 | -2.144107 | -0.015854 |
| 11 | 1 | 0 | -1.515569 | 2.147205 | -0.015926 |
| 12 | 7 | 0 | 3.710569 | -0.001719 | 0.008162 |
| 13 | 6 | 0 | -3.197803 | -0.001222 | 0.013987 |
| 14 | 1 | 0 | -3.566067 | -0.116945 | 1.039804 |
| 15 | 1 | 0 | -3.603609 | 0.933641 | -0.381634 |
| 16 | 1 | 0 | -3.601613 | -0.828490 | -0.576787 |

| System: | isolated **3-product** (*p*-dicyanobenzene) |
|---|---|
| Oxidant: | |
| Nitrogen-donor: | |
| Stationary point: | |
| M06–2X/6–31+G(d) energy (in a.u.): | -416.576126689 |
| Thermal correction to Gibbs Free Energy (in a.u.): | 0.065798 |
| Number of imaginary frequencies: | 0 |

**CARTESIAN COORDINATES**

| Center Number | Atomic Number | Atomic Type | Coordinates (Angstroms) X | Y | Z |
|---|---|---|---|---|---|
| 1 | 6 | 0 | -2.827218 | -0.000017 | 0.000137 |
| 2 | 6 | 0 | -1.386328 | 0.000012 | 0.000066 |
| 3 | 6 | 0 | -0.694130 | -1.216659 | 0.000031 |
| 4 | 6 | 0 | -0.694141 | 1.216669 | 0.000036 |
| 5 | 6 | 0 | 0.694057 | -1.216706 | -0.000036 |
| 6 | 1 | 0 | -1.246008 | -2.150555 | 0.000055 |
| 7 | 6 | 0 | 0.694061 | 1.216721 | -0.000031 |
| 8 | 1 | 0 | -1.246000 | 2.150576 | 0.000064 |
| 9 | 6 | 0 | 1.386339 | 0.000016 | -0.000066 |
| 10 | 1 | 0 | 1.245709 | -2.150678 | -0.000064 |
| 11 | 1 | 0 | 1.245691 | 2.150706 | -0.000055 |
| 12 | 7 | 0 | -3.984356 | -0.000015 | 0.000193 |
| 13 | 6 | 0 | 2.827307 | 0.000001 | -0.000137 |
| 14 | 7 | 0 | 3.984488 | -0.000025 | -0.000194 |

| System: | isolated **4-product** (*p*-methoxybenzonitrile) |
|---|---|
| Oxidant: | |
| Nitrogen-donor: | |
| Stationary point: | |
| M06–2X/6–31+G(d) energy (in a.u.): | -438.845103072 |
| Thermal correction to Gibbs Free Energy (in a.u.): | 0.100022 |
| Number of imaginary frequencies: | 0 |

**CARTESIAN COORDINATES**

| Center Number | Atomic Number | Atomic Type | Coordinates (Angstroms) X | Y | Z |
|---|---|---|---|---|---|
| 1 | 6 | 0 | 2.962802 | -0.180853 | -0.000005 |
| 2 | 6 | 0 | 1.533503 | -0.024822 | -0.000069 |
| 3 | 6 | 0 | 0.963476 | 1.258796 | -0.000202 |
| 4 | 6 | 0 | 0.705099 | -1.148179 | 0.000008 |
| 5 | 6 | 0 | -0.410818 | 1.406357 | -0.000035 |
| 6 | 1 | 0 | 1.607975 | 2.131966 | -0.000455 |
| 7 | 6 | 0 | -0.680040 | -1.006043 | 0.000132 |
| 8 | 1 | 0 | 1.146309 | -2.139852 | -0.000104 |
| 9 | 6 | 0 | -1.240679 | 0.275291 | 0.000313 |
| 10 | 1 | 0 | -0.874089 | 2.387412 | -0.000234 |
| 11 | 1 | 0 | -1.303556 | -1.892073 | -0.000157 |
| 12 | 7 | 0 | 4.114299 | -0.305263 | 0.000057 |
| 13 | 8 | 0 | -2.569604 | 0.523958 | 0.000371 |
| 14 | 6 | 0 | -3.454976 | -0.579847 | -0.000310 |
| 15 | 1 | 0 | -4.458670 | -0.156635 | -0.000645 |
| 16 | 1 | 0 | -3.315303 | -1.194713 | -0.896946 |
| 17 | 1 | 0 | -3.316125 | -1.195125 | 0.896186 |

| System: | **1** |
|---|---|
| Oxidant: | NIS |
| Nitrogen-donor: | $TMSN_3$ |
| Stationary point: | R |
| M06–2X/6–31+G(d) energy (in a.u.): | -8392.39413976 |
| Thermal correction to Gibbs Free Energy (in a.u.): | 0.326445 |
| Number of imaginary frequencies: | 0 |

**CARTESIAN COORDINATES**

---------------------------------------------------------------------

| Center Number | Atomic Number | Atomic Type | Coordinates (Angstroms) X | Y | Z |
|---|---|---|---|---|---|
| 1 | 6 | 0 | 0.997025 | 0.016615 | -0.040658 |
| 2 | 6 | 0 | 0.711167 | 1.196332 | 0.034633 |
| 3 | 6 | 0 | 1.297185 | -1.383450 | -0.111402 |
| 4 | 6 | 0 | 2.003312 | -1.907258 | -1.205284 |
| 5 | 6 | 0 | 0.871082 | -2.240805 | 0.914972 |
| 6 | 6 | 0 | 2.279243 | -3.269868 | -1.263176 |
| 7 | 1 | 0 | 2.338764 | -1.235394 | -1.988800 |
| 8 | 6 | 0 | 1.156708 | -3.600776 | 0.850681 |
| 9 | 1 | 0 | 0.316203 | -1.832065 | 1.754337 |
| 10 | 6 | 0 | 1.861987 | -4.118426 | -0.236376 |
| 11 | 1 | 0 | 2.821089 | -3.672759 | -2.114075 |
| 12 | 1 | 0 | 0.824856 | -4.257431 | 1.648948 |
| 13 | 1 | 0 | 2.081350 | -5.180727 | -0.286186 |
| 14 | 7 | 0 | 4.193377 | 0.417143 | -0.755269 |
| 15 | 7 | 0 | 3.941429 | 1.548839 | -0.365815 |
| 16 | 7 | 0 | 3.667754 | 2.606560 | -0.069411 |
| 17 | 6 | 0 | 0.360676 | 2.583684 | 0.122596 |
| 18 | 6 | 0 | 0.603969 | 3.443709 | -0.958232 |
| 19 | 6 | 0 | -0.236355 | 3.085348 | 1.288702 |
| 20 | 6 | 0 | 0.255398 | 4.786576 | -0.869422 |
| 21 | 1 | 0 | 1.069198 | 3.049125 | -1.856258 |
| 22 | 6 | 0 | -0.581434 | 4.430161 | 1.369294 |
| 23 | 1 | 0 | -0.427489 | 2.412871 | 2.119690 |
| 24 | 6 | 0 | -0.336236 | 5.282630 | 0.292622 |
| 25 | 1 | 0 | 0.447462 | 5.448437 | -1.708259 |
| 26 | 1 | 0 | -1.043329 | 4.813557 | 2.273918 |
| 27 | 1 | 0 | -0.606853 | 6.332119 | 0.358565 |
| 28 | 53 | 0 | -2.258933 | -0.107108 | -0.113620 |
| 29 | 14 | 0 | 5.312732 | -0.649763 | 0.131804 |
| 30 | 6 | 0 | 6.690252 | 0.423028 | 0.823028 |
| 31 | 1 | 0 | 7.424521 | -0.190228 | 1.357697 |
| 32 | 1 | 0 | 7.216338 | 0.959060 | 0.025816 |
| 33 | 1 | 0 | 6.304569 | 1.165429 | 1.531315 |
| 34 | 6 | 0 | 5.932985 | -1.852150 | -1.155861 |
| 35 | 1 | 0 | 5.093360 | -2.390916 | -1.607296 |
| 36 | 1 | 0 | 6.472998 | -1.331395 | -1.953103 |
| 37 | 1 | 0 | 6.606560 | -2.591645 | -0.708943 |
| 38 | 6 | 0 | 4.401363 | -1.521261 | 1.516877 |
| 39 | 1 | 0 | 3.793583 | -0.817083 | 2.096878 |
| 40 | 1 | 0 | 3.734290 | -2.298028 | 1.128424 |
| 41 | 1 | 0 | 5.114448 | -1.994859 | 2.202390 |
| 42 | 6 | 0 | -4.810646 | -1.304881 | 1.046672 |
| 43 | 6 | 0 | -5.149256 | -0.357548 | -1.067201 |
| 44 | 6 | 0 | -6.287316 | -1.519273 | 0.739188 |
| 45 | 6 | 0 | -6.508502 | -0.900952 | -0.644628 |
| 46 | 1 | 0 | -6.492528 | -2.592078 | 0.777697 |
| 47 | 1 | 0 | -6.873537 | -1.044086 | 1.529839 |
| 48 | 1 | 0 | -6.837933 | -1.620769 | -1.398201 |
| 49 | 1 | 0 | -7.222846 | -0.073801 | -0.645839 |
| 50 | 7 | 0 | -4.246198 | -0.633529 | -0.038172 |
| 51 | 8 | 0 | -4.879500 | 0.208863 | -2.095285 |
| 52 | 8 | 0 | -4.218011 | -1.644568 | 2.039853 |

---------------------------------------------------------------------

| System: | **1** |
|---|---|
| Oxidant: | NIS |
| Nitrogen-donor: | $TMSN_3$ |
| Stationary point: | TS1 |
| M06–2X/6–31+G(d) energy (in a.u.): | -8392.35128605 |
| Thermal correction to Gibbs Free Energy (in a.u.): | 0.334131 |
| Number of imaginary frequencies: | 1 (–355 $cm^{-1}$) |

**CARTESIAN COORDINATES**

| Center Number | Atomic Number | Atomic Type | Coordinates (Angstroms) X | Y | Z |
|---|---|---|---|---|---|
| 1 | 6 | 0 | 1.225120 | -0.367551 | -0.294685 |
| 2 | 6 | 0 | 0.515125 | 0.680635 | -0.063364 |
| 3 | 6 | 0 | 1.136286 | -1.824383 | -0.366765 |
| 4 | 6 | 0 | 1.797706 | -2.560653 | -1.358402 |
| 5 | 6 | 0 | 0.352613 | -2.497631 | 0.581969 |
| 6 | 6 | 0 | 1.677967 | -3.946468 | -1.400940 |
| 7 | 1 | 0 | 2.399815 | -2.042799 | -2.099467 |
| 8 | 6 | 0 | 0.226561 | -3.882304 | 0.527813 |
| 9 | 1 | 0 | -0.164955 | -1.930632 | 1.349067 |
| 10 | 6 | 0 | 0.892384 | -4.610143 | -0.458070 |
| 11 | 1 | 0 | 2.189482 | -4.507425 | -2.177512 |
| 12 | 1 | 0 | -0.396805 | -4.389034 | 1.257557 |
| 13 | 1 | 0 | 0.794525 | -5.690826 | -0.496097 |
| 14 | 7 | 0 | 3.013371 | -0.038203 | -0.581614 |
| 15 | 7 | 0 | 3.225823 | 0.912158 | -1.350664 |
| 16 | 7 | 0 | 3.342729 | 1.771656 | -2.066482 |
| 17 | 6 | 0 | 0.893892 | 2.080743 | 0.119893 |
| 18 | 6 | 0 | 0.289452 | 3.097515 | -0.632905 |
| 19 | 6 | 0 | 1.873554 | 2.426775 | 1.064055 |
| 20 | 6 | 0 | 0.695670 | 4.419694 | -0.479083 |
| 21 | 1 | 0 | -0.495284 | 2.841685 | -1.338823 |
| 22 | 6 | 0 | 2.264731 | 3.753494 | 1.226211 |
| 23 | 1 | 0 | 2.308762 | 1.646873 | 1.684615 |
| 24 | 6 | 0 | 1.685339 | 4.753541 | 0.446108 |
| 25 | 1 | 0 | 0.227139 | 5.195178 | -1.077361 |
| 26 | 1 | 0 | 3.016522 | 4.007745 | 1.968556 |
| 27 | 1 | 0 | 1.991126 | 5.787880 | 0.569651 |
| 28 | 53 | 0 | -1.908467 | 0.257660 | 0.002019 |
| 29 | 14 | 0 | 4.346780 | -0.661433 | 0.505754 |
| 30 | 6 | 0 | 5.411231 | 0.835015 | 0.864517 |
| 31 | 1 | 0 | 6.202893 | 0.570281 | 1.574535 |
| 32 | 1 | 0 | 5.899081 | 1.213816 | -0.040785 |
| 33 | 1 | 0 | 4.826488 | 1.652050 | 1.300589 |
| 34 | 6 | 0 | 5.278084 | -1.964821 | -0.453953 |
| 35 | 1 | 0 | 4.634297 | -2.824305 | -0.667158 |
| 36 | 1 | 0 | 5.655065 | -1.570839 | -1.404239 |
| 37 | 1 | 0 | 6.138940 | -2.320535 | 0.123975 |
| 38 | 6 | 0 | 3.471342 | -1.346923 | 2.000290 |
| 39 | 1 | 0 | 2.688092 | -0.672270 | 2.362035 |
| 40 | 1 | 0 | 3.005600 | -2.311975 | 1.776845 |
| 41 | 1 | 0 | 4.193577 | -1.495984 | 2.811262 |
| 42 | 6 | 0 | -4.667527 | -1.143359 | 0.673072 |
| 43 | 6 | 0 | -5.012142 | 0.818163 | -0.481074 |
| 44 | 6 | 0 | -6.187900 | -1.040285 | 0.532962 |
| 45 | 6 | 0 | -6.417117 | 0.264930 | -0.230290 |
| 46 | 1 | 0 | -6.547803 | -1.928247 | 0.006263 |
| 47 | 1 | 0 | -6.629392 | -1.050501 | 1.533119 |
| 48 | 1 | 0 | -6.914163 | 0.131285 | -1.194785 |
| 49 | 1 | 0 | -6.986430 | 1.010068 | 0.331835 |
| 50 | 7 | 0 | -4.087618 | -0.046441 | 0.070662 |
| 51 | 8 | 0 | -4.755573 | 1.847315 | -1.068964 |
| 52 | 8 | 0 | -4.071565 | -2.045754 | 1.225377 |

| System: | **1** |
|---|---|
| Oxidant: | NIS |
| Nitrogen-donor: | $TMSN_3$ |
| Stationary point: | IN1 |
| M06–2X/6–31+G(d) energy (in a.u.): | -8392.35918413 |
| Thermal correction to Gibbs Free Energy (in a.u.): | 0.336647 |
| Number of imaginary frequencies: | 0 |

**CARTESIAN COORDINATES**

| Center Number | Atomic Number | Atomic Type | Coordinates (Angstroms) X | Y | Z |
|---|---|---|---|---|---|
| 1 | 6 | 0 | 1.330752 | -0.369352 | -0.276432 |
| 2 | 6 | 0 | 0.507700 | 0.662466 | -0.052261 |
| 3 | 6 | 0 | 1.058719 | -1.821306 | -0.370874 |
| 4 | 6 | 0 | 1.661294 | -2.595546 | -1.371257 |
| 5 | 6 | 0 | 0.199712 | -2.443914 | 0.544912 |
| 6 | 6 | 0 | 1.403638 | -3.961110 | -1.464177 |
| 7 | 1 | 0 | 2.320708 | -2.124133 | -2.097258 |
| 8 | 6 | 0 | -0.064735 | -3.806192 | 0.444782 |
| 9 | 1 | 0 | -0.273638 | -1.857622 | 1.325265 |
| 10 | 6 | 0 | 0.537754 | -4.568936 | -0.555958 |
| 11 | 1 | 0 | 1.866554 | -4.545203 | -2.254168 |
| 12 | 1 | 0 | -0.753550 | -4.265258 | 1.146635 |
| 13 | 1 | 0 | 0.326137 | -5.631012 | -0.633297 |
| 14 | 7 | 0 | 2.806498 | -0.116470 | -0.490679 |
| 15 | 7 | 0 | 3.102033 | 0.790991 | -1.315894 |
| 16 | 7 | 0 | 3.369861 | 1.599640 | -2.040018 |
| 17 | 6 | 0 | 1.003284 | 2.045416 | 0.092587 |
| 18 | 6 | 0 | 0.521849 | 3.064624 | -0.742843 |
| 19 | 6 | 0 | 1.947462 | 2.375478 | 1.077628 |
| 20 | 6 | 0 | 1.009829 | 4.363280 | -0.625726 |
| 21 | 1 | 0 | -0.242807 | 2.829122 | -1.477880 |
| 22 | 6 | 0 | 2.421436 | 3.679691 | 1.204638 |
| 23 | 1 | 0 | 2.284334 | 1.604943 | 1.768462 |
| 24 | 6 | 0 | 1.963177 | 4.676360 | 0.343835 |
| 25 | 1 | 0 | 0.630109 | 5.138084 | -1.284787 |
| 26 | 1 | 0 | 3.137584 | 3.920793 | 1.985684 |
| 27 | 1 | 0 | 2.330424 | 5.693272 | 0.441358 |
| 28 | 53 | 0 | -1.735604 | 0.395257 | 0.021464 |
| 29 | 14 | 0 | 4.176382 | -0.830136 | 0.571620 |
| 30 | 6 | 0 | 5.314782 | 0.634135 | 0.796521 |
| 31 | 1 | 0 | 6.098595 | 0.377508 | 1.518405 |
| 32 | 1 | 0 | 5.818223 | 0.914409 | -0.135716 |
| 33 | 1 | 0 | 4.782295 | 1.512104 | 1.177697 |
| 34 | 6 | 0 | 4.979042 | -2.192368 | -0.413833 |
| 35 | 1 | 0 | 4.321076 | -3.062005 | -0.499745 |
| 36 | 1 | 0 | 5.243451 | -1.856527 | -1.422978 |
| 37 | 1 | 0 | 5.905309 | -2.507810 | 0.080810 |
| 38 | 6 | 0 | 3.295239 | -1.393366 | 2.106891 |
| 39 | 1 | 0 | 2.566640 | -0.655572 | 2.458102 |
| 40 | 1 | 0 | 2.763535 | -2.333291 | 1.930327 |
| 41 | 1 | 0 | 4.028102 | -1.555810 | 2.905767 |
| 42 | 6 | 0 | -4.606633 | -1.073746 | 0.632635 |
| 43 | 6 | 0 | -5.032117 | 0.906247 | -0.400640 |
| 44 | 6 | 0 | -6.137008 | -1.035175 | 0.518898 |
| 45 | 6 | 0 | -6.423341 | 0.297457 | -0.170506 |
| 46 | 1 | 0 | -6.467327 | -1.908793 | -0.050317 |
| 47 | 1 | 0 | -6.564801 | -1.117274 | 1.522108 |
| 48 | 1 | 0 | -6.926189 | 0.198818 | -1.136612 |
| 49 | 1 | 0 | -7.011877 | 0.992029 | 0.435552 |
| 50 | 7 | 0 | -4.068690 | 0.064018 | 0.093440 |
| 51 | 8 | 0 | -4.832792 | 1.978419 | -0.944558 |
| 52 | 8 | 0 | -3.977726 | -1.993368 | 1.133989 |

| System: | **1** |
|---|---|
| Oxidant: | NIS |
| Nitrogen-donor: | $TMSN_3$ |
| Stationary point: | TS2 |
| M06–2X/6–31+G(d) energy (in a.u.): | -8392.38637197 |
| Thermal correction to Gibbs Free Energy (in a.u.): | 0.346592 |
| Number of imaginary frequencies: | 1 (–33 $cm^{-1}$) |

**CARTESIAN COORDINATES**

| Center Number | Atomic Number | Atomic Type | Coordinates (Angstroms) X | Y | Z |
|---|---|---|---|---|---|
| 1 | 6 | 0 | 0.432760 | 0.375575 | -0.630881 |
| 2 | 6 | 0 | 1.610516 | 0.001871 | -0.106303 |
| 3 | 6 | 0 | -0.144339 | 1.730332 | -0.780312 |
| 4 | 6 | 0 | -0.492811 | 2.185413 | -2.056280 |
| 5 | 6 | 0 | -0.363899 | 2.547791 | 0.332774 |
| 6 | 6 | 0 | -1.048612 | 3.449845 | -2.224037 |
| 7 | 1 | 0 | -0.330901 | 1.544026 | -2.920473 |
| 8 | 6 | 0 | -0.931554 | 3.807864 | 0.162027 |
| 9 | 1 | 0 | -0.099452 | 2.178380 | 1.318596 |
| 10 | 6 | 0 | -1.273816 | 4.260731 | -1.112161 |
| 11 | 1 | 0 | -1.312156 | 3.798069 | -3.218012 |
| 12 | 1 | 0 | -1.109826 | 4.437448 | 1.028530 |
| 13 | 1 | 0 | -1.717042 | 5.243914 | -1.238950 |
| 14 | 7 | 0 | -0.437388 | -0.650842 | -1.209070 |
| 15 | 7 | 0 | 0.162701 | -1.459777 | -1.972603 |
| 16 | 7 | 0 | 0.659633 | -2.203638 | -2.641907 |
| 17 | 6 | 0 | 2.034501 | -1.405344 | 0.086862 |
| 18 | 6 | 0 | 3.268321 | -1.859865 | -0.395379 |
| 19 | 6 | 0 | 1.167804 | -2.308827 | 0.719444 |
| 20 | 6 | 0 | 3.622053 | -3.200339 | -0.268417 |
| 21 | 1 | 0 | 3.949689 | -1.160178 | -0.872223 |
| 22 | 6 | 0 | 1.529953 | -3.647521 | 0.847073 |
| 23 | 1 | 0 | 0.227869 | -1.941135 | 1.120589 |
| 24 | 6 | 0 | 2.752879 | -4.098207 | 0.350860 |
| 25 | 1 | 0 | 4.578536 | -3.542393 | -0.651686 |
| 26 | 1 | 0 | 0.854401 | -4.337784 | 1.343755 |
| 27 | 1 | 0 | 3.031129 | -5.142823 | 0.452572 |
| 28 | 53 | 0 | 2.985214 | 1.475591 | 0.520917 |
| 29 | 14 | 0 | -2.383470 | -0.984226 | -1.134745 |
| 30 | 6 | 0 | -2.581667 | -2.746062 | -0.495345 |
| 31 | 1 | 0 | -3.459700 | -3.186773 | -0.981331 |
| 32 | 1 | 0 | -1.709663 | -3.328149 | -0.828659 |
| 33 | 1 | 0 | -2.685316 | -2.846242 | 0.583136 |
| 34 | 6 | 0 | -2.435834 | -1.439552 | -3.017995 |
| 35 | 1 | 0 | -1.971751 | -0.686468 | -3.669942 |
| 36 | 1 | 0 | -2.035224 | -2.424263 | -3.287938 |
| 37 | 1 | 0 | -3.500558 | -1.461615 | -3.289966 |
| 38 | 6 | 0 | -3.315441 | 0.643282 | -1.288445 |
| 39 | 1 | 0 | -4.383571 | 0.402761 | -1.285277 |
| 40 | 1 | 0 | -3.116146 | 1.350455 | -0.481407 |
| 41 | 1 | 0 | -3.068661 | 1.106209 | -2.248434 |
| 42 | 6 | 0 | -3.559336 | -0.562691 | 1.537883 |
| 43 | 6 | 0 | -1.468778 | -0.081358 | 2.180920 |
| 44 | 6 | 0 | -3.723301 | -0.200130 | 3.011526 |
| 45 | 6 | 0 | -2.308900 | 0.162855 | 3.438705 |
| 46 | 1 | 0 | -4.444747 | 0.617266 | 3.095455 |
| 47 | 1 | 0 | -4.141404 | -1.060894 | 3.541103 |
| 48 | 1 | 0 | -2.191141 | 1.211171 | 3.730247 |

| | | | | | |
|---|---|---|---|---|---|
| 49 | 1 | 0 | -1.910763 | -0.447887 | 4.253190 |
| 50 | 7 | 0 | -2.253805 | -0.439891 | 1.115421 |
| 51 | 8 | 0 | -0.249032 | 0.038655 | 2.176411 |
| 52 | 8 | 0 | -4.481262 | -0.934301 | 0.821956 |

---------------------------------------------------------------------

| System: | **1** |
|---|---|
| Oxidant: | NIS |
| Nitrogen-donor: | $TMSN_3$ |
| Stationary point: | IN2 |
| M06–2X/6–31+G(d) energy (in a.u.): | -7623.31808876 |
| Thermal correction to Gibbs Free Energy (in a.u.): | 0.164029 |
| Number of imaginary frequencies: | 0 |

**CARTESIAN COORDINATES**

| Center Number | Atomic Number | Atomic Type | Coordinates (Angstroms) X | Y | Z |
|---|---|---|---|---|---|
| 1 | 6 | 0 | 0.468578 | 0.997297 | 0.047850 |
| 2 | 6 | 0 | -0.396310 | -0.040975 | 0.031556 |
| 3 | 6 | 0 | 1.949783 | 0.914451 | 0.132629 |
| 4 | 6 | 0 | 2.733728 | 1.606816 | -0.794656 |
| 5 | 6 | 0 | 2.572983 | 0.195340 | 1.156449 |
| 6 | 6 | 0 | 4.122866 | 1.550894 | -0.722363 |
| 7 | 1 | 0 | 2.248297 | 2.187259 | -1.574205 |
| 8 | 6 | 0 | 3.961902 | 0.150400 | 1.236525 |
| 9 | 1 | 0 | 1.966765 | -0.321955 | 1.894761 |
| 10 | 6 | 0 | 4.739308 | 0.821115 | 0.293270 |
| 11 | 1 | 0 | 4.723109 | 2.081857 | -1.455097 |
| 12 | 1 | 0 | 4.437691 | -0.405060 | 2.039100 |
| 13 | 1 | 0 | 5.822903 | 0.782074 | 0.354854 |
| 14 | 7 | 0 | 0.049451 | 2.360559 | 0.007615 |
| 15 | 7 | 0 | -1.003774 | 2.635286 | -0.589668 |
| 16 | 6 | 0 | -1.864671 | 0.087317 | 0.180769 |
| 17 | 6 | 0 | -2.753517 | -0.431439 | -0.768980 |
| 18 | 6 | 0 | -2.378386 | 0.749976 | 1.303095 |
| 19 | 6 | 0 | -4.125990 | -0.270257 | -0.609691 |
| 20 | 1 | 0 | -2.363076 | -0.957957 | -1.636126 |
| 21 | 6 | 0 | -3.753372 | 0.905709 | 1.462919 |
| 22 | 1 | 0 | -1.690344 | 1.142975 | 2.047612 |
| 23 | 6 | 0 | -4.630334 | 0.398142 | 0.506183 |
| 24 | 1 | 0 | -4.804755 | -0.668593 | -1.357768 |
| 25 | 1 | 0 | -4.138095 | 1.419751 | 2.338769 |
| 26 | 1 | 0 | -5.702286 | 0.518848 | 0.630697 |
| 27 | 53 | 0 | 0.297538 | -2.032941 | -0.229865 |
| 28 | 7 | 0 | -1.924129 | 3.026720 | -1.108391 |

| System: | **1** |
|---|---|
| Oxidant: | NIS |
| Nitrogen-donor: | $TMSN_3$ |
| Stationary point: | TS3 |
| M06–2X/6–31+G(d) energy (in a.u.): | -7623.27067785 |
| Thermal correction to Gibbs Free Energy (in a.u.): | 0.160160 |
| Number of imaginary frequencies: | 1 (–572 $cm^{-1}$) |

**CARTESIAN COORDINATES**

| Center Number | Atomic Number | Atomic Type | Coordinates (Angstroms) X | Y | Z |
|---|---|---|---|---|---|
| 1 | 6 | 0 | 0.550190 | -0.799826 | 0.214186 |
| 2 | 6 | 0 | -0.620023 | -0.066506 | 0.059966 |
| 3 | 6 | 0 | 1.902292 | -0.254226 | -0.084185 |
| 4 | 6 | 0 | 2.994010 | -0.610353 | 0.714715 |
| 5 | 6 | 0 | 2.102744 | 0.560672 | -1.203280 |
| 6 | 6 | 0 | 4.270313 | -0.147458 | 0.403796 |
| 7 | 1 | 0 | 2.828440 | -1.233404 | 1.590207 |
| 8 | 6 | 0 | 3.378587 | 1.019826 | -1.513997 |
| 9 | 1 | 0 | 1.253010 | 0.827354 | -1.826744 |
| 10 | 6 | 0 | 4.464463 | 0.667772 | -0.710360 |
| 11 | 1 | 0 | 5.112769 | -0.420714 | 1.032199 |
| 12 | 1 | 0 | 3.528562 | 1.649767 | -2.385810 |
| 13 | 1 | 0 | 5.459852 | 1.026828 | -0.954699 |
| 14 | 6 | 0 | -0.672453 | 1.395839 | 0.225861 |
| 15 | 6 | 0 | -1.527951 | 2.191797 | -0.553214 |
| 16 | 6 | 0 | 0.169174 | 2.026322 | 1.156814 |
| 17 | 6 | 0 | -1.508047 | 3.576731 | -0.433289 |
| 18 | 1 | 0 | -2.202429 | 1.720625 | -1.262023 |
| 19 | 6 | 0 | 0.177518 | 3.411911 | 1.281954 |
| 20 | 1 | 0 | 0.811152 | 1.421152 | 1.790109 |
| 21 | 6 | 0 | -0.656103 | 4.193475 | 0.483518 |
| 22 | 1 | 0 | -2.165585 | 4.177620 | -1.054426 |
| 23 | 1 | 0 | 0.832515 | 3.879427 | 2.011138 |
| 24 | 1 | 0 | -0.650995 | 5.274802 | 0.582676 |
| 25 | 7 | 0 | 0.354191 | -1.965747 | 0.826426 |
| 26 | 7 | 0 | 1.437020 | -3.141259 | 0.179188 |
| 27 | 53 | 0 | -2.469803 | -1.070219 | -0.176477 |
| 28 | 7 | 0 | 1.915070 | -4.121235 | 0.362348 |

| System: | **1** |
|---|---|
| Oxidant: | NIS |
| Nitrogen-donor: | $TMSN_3$ |
| Stationary point: | IN3 |
| M06–2X/6–31+G(d) energy (in a.u.): | -7513.88727644 |
| Thermal correction to Gibbs Free Energy (in a.u.): | 0.156399 |
| Number of imaginary frequencies: | 0 |

**CARTESIAN COORDINATES**

| Center Number | Atomic Number | Atomic Type | Coordinates (Angstroms) X | Y | Z |
|---|---|---|---|---|---|
| 1 | 6 | 0 | -0.937844 | 0.146406 | 0.768542 |
| 2 | 6 | 0 | 0.459856 | -0.089291 | 0.545980 |
| 3 | 7 | 0 | -0.269841 | 0.002941 | 1.835322 |
| 4 | 6 | 0 | -2.267585 | 0.382929 | 0.250476 |
| 5 | 6 | 0 | -2.461371 | 0.417072 | -1.133560 |
| 6 | 6 | 0 | -3.340307 | 0.576723 | 1.129390 |
| 7 | 6 | 0 | -3.736438 | 0.646703 | -1.641573 |
| 8 | 1 | 0 | -1.612726 | 0.259323 | -1.794199 |
| 9 | 6 | 0 | -4.609788 | 0.809579 | 0.614655 |
| 10 | 1 | 0 | -3.165037 | 0.544123 | 2.201049 |
| 11 | 6 | 0 | -4.806095 | 0.843761 | -0.768438 |
| 12 | 1 | 0 | -3.897000 | 0.672106 | -2.714659 |
| 13 | 1 | 0 | -5.447704 | 0.963887 | 1.287342 |
| 14 | 1 | 0 | -5.800205 | 1.023738 | -1.167034 |
| 15 | 6 | 0 | 1.437143 | 0.987776 | 0.182813 |
| 16 | 6 | 0 | 2.095218 | 1.014195 | -1.049346 |
| 17 | 6 | 0 | 1.656973 | 2.020697 | 1.098450 |
| 18 | 6 | 0 | 2.954961 | 2.063472 | -1.363145 |
| 19 | 1 | 0 | 1.943409 | 0.207040 | -1.760111 |
| 20 | 6 | 0 | 2.525190 | 3.064706 | 0.785489 |
| 21 | 1 | 0 | 1.146867 | 1.999335 | 2.057321 |
| 22 | 6 | 0 | 3.174252 | 3.091635 | -0.446781 |
| 23 | 1 | 0 | 3.459538 | 2.073436 | -2.324846 |
| 24 | 1 | 0 | 2.694190 | 3.856421 | 1.509393 |
| 25 | 1 | 0 | 3.849944 | 3.905740 | -0.691486 |
| 26 | 53 | 0 | 1.053823 | -2.101583 | -0.062033 |

| System: | **1** |
|---|---|
| Oxidant: | NIS |
| Nitrogen-donor: | $TMSN_3$ |
| Stationary point: | TS4 |
| M06–2X/6–31+G(d) energy (in a.u.): | -8087.18465593 |
| Thermal correction to Gibbs Free Energy (in a.u.): | 0.269473 |
| Number of imaginary frequencies: | 1 (–227 $cm^{-1}$) |

**CARTESIAN COORDINATES**

| Center Number | Atomic Number | Atomic Type | Coordinates (Angstroms) X | Y | Z |
|---|---|---|---|---|---|
| 1 | 6 | 0 | -0.547799 | 1.148998 | 1.150299 |
| 2 | 6 | 0 | 0.830894 | 1.269837 | 1.190292 |
| 3 | 7 | 0 | 0.154082 | 1.232152 | 2.317501 |
| 4 | 6 | 0 | -1.817304 | 1.627521 | 0.659451 |
| 5 | 6 | 0 | -2.012270 | 1.818206 | -0.714023 |
| 6 | 6 | 0 | -2.841911 | 1.876349 | 1.585931 |
| 7 | 6 | 0 | -3.244641 | 2.294882 | -1.155531 |
| 8 | 1 | 0 | -1.225733 | 1.545368 | -1.414965 |
| 9 | 6 | 0 | -4.063192 | 2.350978 | 1.130547 |
| 10 | 1 | 0 | -2.664572 | 1.700058 | 2.643375 |
| 11 | 6 | 0 | -4.259888 | 2.561559 | -0.239250 |
| 12 | 1 | 0 | -3.412676 | 2.436662 | -2.217874 |
| 13 | 1 | 0 | -4.863962 | 2.554440 | 1.834023 |
| 14 | 1 | 0 | -5.220161 | 2.926493 | -0.591598 |
| 15 | 6 | 0 | 2.175726 | 1.452470 | 0.738528 |
| 16 | 6 | 0 | 2.469027 | 1.527232 | -0.629658 |
| 17 | 6 | 0 | 3.185243 | 1.560634 | 1.712515 |
| 18 | 6 | 0 | 3.792877 | 1.705494 | -1.020458 |
| 19 | 1 | 0 | 1.678919 | 1.391658 | -1.362955 |
| 20 | 6 | 0 | 4.497335 | 1.741388 | 1.307851 |
| 21 | 1 | 0 | 2.924890 | 1.498513 | 2.765891 |
| 22 | 6 | 0 | 4.796666 | 1.810746 | -0.059143 |
| 23 | 1 | 0 | 4.036594 | 1.745159 | -2.076774 |
| 24 | 1 | 0 | 5.289459 | 1.824041 | 2.044694 |
| 25 | 1 | 0 | 5.828001 | 1.944090 | -0.372791 |
| 26 | 53 | 0 | 0.225786 | -0.812572 | -2.328976 |
| 27 | 7 | 0 | -1.055085 | -0.971001 | 1.080484 |
| 28 | 7 | 0 | -2.049511 | -1.142120 | 0.369940 |
| 29 | 7 | 0 | -2.974520 | -1.193143 | -0.269413 |
| 30 | 14 | 0 | 0.014563 | -2.399974 | 1.447768 |
| 31 | 6 | 0 | -0.331262 | -2.783627 | 3.251059 |
| 32 | 1 | 0 | -1.374515 | -3.078960 | 3.405578 |
| 33 | 1 | 0 | 0.307407 | -3.601060 | 3.605547 |
| 34 | 1 | 0 | -0.132680 | -1.905403 | 3.875923 |
| 35 | 6 | 0 | 1.792145 | -1.876758 | 1.233019 |
| 36 | 1 | 0 | 2.424547 | -2.769273 | 1.318281 |
| 37 | 1 | 0 | 1.954446 | -1.440054 | 0.241546 |
| 38 | 1 | 0 | 2.116094 | -1.174777 | 2.009304 |
| 39 | 6 | 0 | -0.510135 | -3.784074 | 0.313312 |
| 40 | 1 | 0 | -0.381712 | -3.483972 | -0.732740 |
| 41 | 1 | 0 | 0.107451 | -4.670282 | 0.500217 |
| 42 | 1 | 0 | -1.556177 | -4.068968 | 0.474294 |

| System: | **1** |
|---|---|
| Oxidant: | NIS |
| Nitrogen-donor: | $TMSN_3$ |
| Stationary point: | IN4 |
| M06–2X/6–31+G(d) energy (in a.u.): | -1167.06440988 |
| Thermal correction to Gibbs Free Energy (in a.u.): | 0.272801 |
| Number of imaginary frequencies: | 0 |

**CARTESIAN COORDINATES**

| Center Number | Atomic Number | Atomic Type | Coordinates (Angstroms) X | Y | Z |
|---|---|---|---|---|---|
| 1 | 6 | 0 | 0.186504 | 0.167090 | -0.241858 |
| 2 | 6 | 0 | -1.165307 | -0.210105 | -0.589958 |
| 3 | 7 | 0 | -0.319521 | -0.385671 | -1.517018 |
| 4 | 6 | 0 | 0.709098 | 1.564678 | -0.154214 |
| 5 | 6 | 0 | 1.819821 | 1.903157 | 0.623451 |
| 6 | 6 | 0 | 0.049111 | 2.561930 | -0.881685 |
| 7 | 6 | 0 | 2.266372 | 3.222790 | 0.672628 |
| 8 | 1 | 0 | 2.359882 | 1.147752 | 1.189688 |
| 9 | 6 | 0 | 0.500224 | 3.876836 | -0.831823 |
| 10 | 1 | 0 | -0.812608 | 2.310736 | -1.494170 |
| 11 | 6 | 0 | 1.608614 | 4.211522 | -0.054169 |
| 12 | 1 | 0 | 3.130974 | 3.474203 | 1.278710 |
| 13 | 1 | 0 | -0.016267 | 4.641688 | -1.402917 |
| 14 | 1 | 0 | 1.957419 | 5.238384 | -0.015711 |
| 15 | 6 | 0 | -2.563529 | -0.287033 | -0.244953 |
| 16 | 6 | 0 | -2.997778 | 0.217018 | 0.987793 |
| 17 | 6 | 0 | -3.469140 | -0.856519 | -1.152102 |
| 18 | 6 | 0 | -4.347055 | 0.150170 | 1.315152 |
| 19 | 1 | 0 | -2.282380 | 0.672373 | 1.668781 |
| 20 | 6 | 0 | -4.814181 | -0.921865 | -0.814101 |
| 21 | 1 | 0 | -3.111353 | -1.236877 | -2.104735 |
| 22 | 6 | 0 | -5.249526 | -0.420627 | 0.416071 |
| 23 | 1 | 0 | -4.696939 | 0.542651 | 2.264147 |
| 24 | 1 | 0 | -5.526305 | -1.360486 | -1.505289 |
| 25 | 1 | 0 | -6.302807 | -0.473122 | 0.673980 |
| 26 | 7 | 0 | 1.013761 | -0.842361 | 0.494904 |
| 27 | 7 | 0 | 0.813182 | -0.906074 | 1.739530 |
| 28 | 7 | 0 | 0.647008 | -0.966733 | 2.842105 |
| 29 | 14 | 0 | 2.353993 | -1.897768 | -0.361156 |
| 30 | 6 | 0 | 1.421821 | -3.294424 | -1.151735 |
| 31 | 1 | 0 | 2.127755 | -3.997402 | -1.609384 |
| 32 | 1 | 0 | 0.826577 | -3.851836 | -0.421199 |
| 33 | 1 | 0 | 0.757371 | -2.923270 | -1.937605 |
| 34 | 6 | 0 | 3.403293 | -2.387293 | 1.097896 |
| 35 | 1 | 0 | 4.272722 | -2.950418 | 0.739203 |
| 36 | 1 | 0 | 3.789843 | -1.518533 | 1.642630 |
| 37 | 1 | 0 | 2.874897 | -3.041125 | 1.800617 |
| 38 | 6 | 0 | 3.108903 | -0.679259 | -1.537093 |
| 39 | 1 | 0 | 3.620075 | 0.136481 | -1.017586 |
| 40 | 1 | 0 | 3.842943 | -1.194377 | -2.167768 |
| 41 | 1 | 0 | 2.347822 | -0.250598 | -2.197663 |

| System: | **1** |
|---|---|
| Oxidant: | NIS |
| Nitrogen-donor: | $TMSN_3$ |
| Stationary point: | TS5 |
| M06–2X/6–31+G(d) energy (in a.u.): | -1527.20016280 |
| Thermal correction to Gibbs Free Energy (in a.u.): | 0.349979 |
| Number of imaginary frequencies: | 1 (–58 $cm^{-1}$) |

**CARTESIAN COORDINATES**

| Center Number | Atomic Number | Atomic Type | Coordinates (Angstroms) X | Y | Z |
|---|---|---|---|---|---|
| 1 | 6 | 0 | -1.114524 | -0.582066 | 0.289432 |
| 2 | 6 | 0 | -1.249248 | 0.800173 | 0.705077 |
| 3 | 7 | 0 | -1.046566 | -0.008734 | 1.655116 |
| 4 | 6 | 0 | -2.272352 | -1.448802 | -0.096239 |
| 5 | 6 | 0 | -2.109301 | -2.626771 | -0.830261 |
| 6 | 6 | 0 | -3.560467 | -1.060085 | 0.290298 |
| 7 | 6 | 0 | -3.215570 | -3.403286 | -1.171213 |
| 8 | 1 | 0 | -1.117324 | -2.957717 | -1.125357 |
| 9 | 6 | 0 | -4.662213 | -1.838638 | -0.048752 |
| 10 | 1 | 0 | -3.701187 | -0.146746 | 0.862355 |
| 11 | 6 | 0 | -4.494229 | -3.012780 | -0.782493 |
| 12 | 1 | 0 | -3.072634 | -4.318328 | -1.737997 |
| 13 | 1 | 0 | -5.654967 | -1.525895 | 0.260472 |
| 14 | 1 | 0 | -5.354942 | -3.618994 | -1.047711 |
| 15 | 6 | 0 | -1.641164 | 2.168714 | 0.436086 |
| 16 | 6 | 0 | -1.763394 | 2.620941 | -0.879483 |
| 17 | 6 | 0 | -1.925763 | 3.013009 | 1.514784 |
| 18 | 6 | 0 | -2.178574 | 3.925998 | -1.116716 |
| 19 | 1 | 0 | -1.504320 | 1.956219 | -1.697921 |
| 20 | 6 | 0 | -2.337119 | 4.318351 | 1.270261 |
| 21 | 1 | 0 | -1.822869 | 2.637996 | 2.529451 |
| 22 | 6 | 0 | -2.464383 | 4.771851 | -0.043955 |
| 23 | 1 | 0 | -2.269720 | 4.288711 | -2.135579 |
| 24 | 1 | 0 | -2.558591 | 4.982096 | 2.100198 |
| 25 | 1 | 0 | -2.785323 | 5.792118 | -0.232906 |
| 26 | 7 | 0 | 0.195131 | -1.052093 | -0.207219 |
| 27 | 7 | 0 | 0.411995 | -0.810225 | -1.424414 |
| 28 | 7 | 0 | 0.606946 | -0.666259 | -2.512294 |
| 29 | 14 | 0 | 1.588666 | -1.791708 | 0.886798 |
| 30 | 6 | 0 | 2.595812 | -2.869753 | -0.261276 |
| 31 | 1 | 0 | 2.891871 | -2.358958 | -1.179550 |
| 32 | 1 | 0 | 3.529432 | -3.140263 | 0.243576 |
| 33 | 1 | 0 | 2.041854 | -3.786598 | -0.493718 |
| 34 | 6 | 0 | 0.413465 | -2.983955 | 1.778759 |
| 35 | 1 | 0 | 1.017676 | -3.610556 | 2.449003 |
| 36 | 1 | 0 | -0.321675 | -2.460181 | 2.399584 |
| 37 | 1 | 0 | -0.123909 | -3.655115 | 1.099838 |
| 38 | 6 | 0 | 2.246045 | -0.698494 | 2.255419 |
| 39 | 1 | 0 | 1.785593 | 0.292247 | 2.194345 |
| 40 | 1 | 0 | 2.000713 | -1.135445 | 3.229234 |
| 41 | 1 | 0 | 3.329275 | -0.584135 | 2.160300 |
| 42 | 7 | 0 | 2.717117 | 0.109223 | -0.371355 |
| 43 | 6 | 0 | 4.089533 | 0.146499 | -0.296234 |
| 44 | 6 | 0 | 4.627341 | 1.549867 | -0.606568 |
| 45 | 6 | 0 | 3.361921 | 2.385107 | -0.769284 |
| 46 | 6 | 0 | 2.239187 | 1.357375 | -0.603509 |
| 47 | 8 | 0 | 1.040440 | 1.656120 | -0.680799 |
| 48 | 1 | 0 | 3.243186 | 3.161385 | -0.007256 |

| | | | | | |
|---|---|---|---|---|---|
| 49 | 1 | 0 | 5.277114 | 1.871103 | 0.211836 |
| 50 | 8 | 0 | 4.800172 | -0.802374 | 0.002658 |
| 51 | 1 | 0 | 3.261110 | 2.868820 | -1.745033 |
| 52 | 1 | 0 | 5.241025 | 1.499277 | -1.510838 |

---------------------------------------------------------------------

| System: | **1** |
| --- | --- |
| Oxidant: | NIS |
| Nitrogen-donor: | $TMSN_3$ |
| Stationary point: | IN5 |
| M06–2X/6–31+G(d) energy (in a.u.): | -758.109104162 |
| Thermal correction to Gibbs Free Energy (in a.u.): | 0.169376 |
| Number of imaginary frequencies: | 0 |

**CARTESIAN COORDINATES**

| Center Number | Atomic Number | Atomic Type | Coordinates (Angstroms) X | Y | Z |
| --- | --- | --- | --- | --- | --- |
| 1 | 6 | 0 | -0.722235 | 0.864736 | -0.275842 |
| 2 | 6 | 0 | 0.606900 | 0.374467 | -0.566237 |
| 3 | 7 | 0 | 0.021419 | 0.861043 | -1.578665 |
| 4 | 6 | 0 | -1.900337 | -0.041094 | -0.119353 |
| 5 | 6 | 0 | -2.993727 | 0.316651 | 0.673310 |
| 6 | 6 | 0 | -1.907566 | -1.275644 | -0.778001 |
| 7 | 6 | 0 | -4.076253 | -0.551332 | 0.806162 |
| 8 | 1 | 0 | -2.994710 | 1.275245 | 1.180258 |
| 9 | 6 | 0 | -2.991632 | -2.138069 | -0.645209 |
| 10 | 1 | 0 | -1.066752 | -1.559725 | -1.404845 |
| 11 | 6 | 0 | -4.080197 | -1.779707 | 0.149249 |
| 12 | 1 | 0 | -4.921170 | -0.261571 | 1.424097 |
| 13 | 1 | 0 | -2.985688 | -3.091554 | -1.165122 |
| 14 | 1 | 0 | -4.925641 | -2.453263 | 0.253461 |
| 15 | 6 | 0 | 1.850093 | -0.258694 | -0.163492 |
| 16 | 6 | 0 | 2.000781 | -0.708938 | 1.151238 |
| 17 | 6 | 0 | 2.882658 | -0.416626 | -1.096079 |
| 18 | 6 | 0 | 3.190434 | -1.321385 | 1.534985 |
| 19 | 1 | 0 | 1.184057 | -0.577868 | 1.856271 |
| 20 | 6 | 0 | 4.068043 | -1.027573 | -0.705565 |
| 21 | 1 | 0 | 2.742700 | -0.056441 | -2.111606 |
| 22 | 6 | 0 | 4.220627 | -1.478786 | 0.608083 |
| 23 | 1 | 0 | 3.314882 | -1.674692 | 2.553746 |
| 24 | 1 | 0 | 4.875102 | -1.152950 | -1.420715 |
| 25 | 1 | 0 | 5.148764 | -1.955526 | 0.909352 |
| 26 | 7 | 0 | -0.938884 | 2.147146 | 0.384797 |
| 27 | 7 | 0 | -0.026413 | 2.963177 | 0.242978 |
| 28 | 7 | 0 | 0.764295 | 3.765823 | 0.163120 |

| System: | **1** |
| --- | --- |
| Oxidant: | NIS |
| Nitrogen-donor: | $TMSN_3$ |
| Stationary point: | TS6 |
| M06–2X/6–31+G(d) energy (in a.u.): | -758.048676779 |
| Thermal correction to Gibbs Free Energy (in a.u.): | 0.163760 |
| Number of imaginary frequencies: | 1 (–619 $cm^{-1}$) |

**CARTESIAN COORDINATES**

| Center Number | Atomic Number | Atomic Type | Coordinates (Angstroms) X | Y | Z |
| --- | --- | --- | --- | --- | --- |
| 1 | 6 | 0 | 0.582102 | 0.122194 | 1.140537 |
| 2 | 6 | 0 | -0.711497 | 0.829772 | 0.878712 |
| 3 | 7 | 0 | -0.010662 | 0.229844 | 2.216307 |
| 4 | 6 | 0 | 1.728998 | -0.362707 | 0.393641 |
| 5 | 6 | 0 | 1.672900 | -0.363249 | -1.002925 |
| 6 | 6 | 0 | 2.867466 | -0.818352 | 1.067826 |
| 7 | 6 | 0 | 2.763877 | -0.828633 | -1.730605 |
| 8 | 1 | 0 | 0.772097 | -0.011910 | -1.501038 |
| 9 | 6 | 0 | 3.953653 | -1.277814 | 0.332301 |
| 10 | 1 | 0 | 2.889118 | -0.805438 | 2.153744 |
| 11 | 6 | 0 | 3.901363 | -1.282560 | -1.063334 |
| 12 | 1 | 0 | 2.727621 | -0.837108 | -2.815408 |
| 13 | 1 | 0 | 4.842642 | -1.632422 | 0.844821 |
| 14 | 1 | 0 | 4.753635 | -1.641469 | -1.632889 |
| 15 | 6 | 0 | -1.837054 | -0.002360 | 0.335283 |
| 16 | 6 | 0 | -2.763860 | 0.589482 | -0.529523 |
| 17 | 6 | 0 | -1.974376 | -1.358563 | 0.652930 |
| 18 | 6 | 0 | -3.804846 | -0.162063 | -1.068599 |
| 19 | 1 | 0 | -2.662703 | 1.645613 | -0.760233 |
| 20 | 6 | 0 | -3.019887 | -2.106199 | 0.115463 |
| 21 | 1 | 0 | -1.271875 | -1.830108 | 1.334113 |
| 22 | 6 | 0 | -3.937459 | -1.512839 | -0.749535 |
| 23 | 1 | 0 | -4.518795 | 0.311369 | -1.736941 |
| 24 | 1 | 0 | -3.116238 | -3.156656 | 0.375229 |
| 25 | 1 | 0 | -4.751222 | -2.097142 | -1.169193 |
| 26 | 7 | 0 | -0.714841 | 2.179594 | 0.800351 |
| 27 | 7 | 0 | 0.397882 | 2.728314 | -0.399888 |
| 28 | 7 | 0 | 0.871542 | 3.613479 | -0.863234 |

| System: | **2** |
|---|---|
| Oxidant: | NIS |
| Nitrogen-donor: | $TMSN_3$ |
| Stationary point: | R |
| M06–2X/6–31+G(d) energy (in a.u.): | -8470.98861381 |
| Thermal correction to Gibbs Free Energy (in a.u.): | 0.378218 |
| Number of imaginary frequencies: | 0 |

**CARTESIAN COORDINATES**

---------------------------------------------------------------------

| Center Number | Atomic Number | Atomic Type | Coordinates (Angstroms) X | Y | Z |
|---|---|---|---|---|---|
| 1 | 6 | 0 | -0.267728 | 1.654868 | -1.085051 |
| 2 | 6 | 0 | -0.937843 | 0.656620 | -1.269265 |
| 3 | 6 | 0 | 0.484005 | 2.856844 | -0.874193 |
| 4 | 6 | 0 | 0.511604 | 3.852867 | -1.862041 |
| 5 | 6 | 0 | 1.193955 | 3.064166 | 0.318476 |
| 6 | 6 | 0 | 1.232718 | 5.022784 | -1.656543 |
| 7 | 1 | 0 | -0.039034 | 3.700190 | -2.785054 |
| 8 | 6 | 0 | 1.911680 | 4.239023 | 0.509060 |
| 9 | 1 | 0 | 1.178852 | 2.299956 | 1.090251 |
| 10 | 6 | 0 | 1.946718 | 5.235151 | -0.472339 |
| 11 | 1 | 0 | 1.242084 | 5.786817 | -2.430121 |
| 12 | 1 | 0 | 2.456694 | 4.385633 | 1.438427 |
| 13 | 7 | 0 | -3.853152 | 1.028531 | 0.598618 |
| 14 | 7 | 0 | -4.574441 | 0.204981 | 0.059191 |
| 15 | 7 | 0 | -5.245306 | -0.514886 | -0.501157 |
| 16 | 6 | 0 | -1.746045 | -0.504305 | -1.495984 |
| 17 | 6 | 0 | -2.463475 | -0.640519 | -2.693372 |
| 18 | 6 | 0 | -1.834564 | -1.520944 | -0.531931 |
| 19 | 6 | 0 | -3.252025 | -1.764007 | -2.913207 |
| 20 | 1 | 0 | -2.396599 | 0.141220 | -3.443759 |
| 21 | 6 | 0 | -2.630666 | -2.635729 | -0.764198 |
| 22 | 1 | 0 | -1.265364 | -1.439183 | 0.390284 |
| 23 | 6 | 0 | -3.356652 | -2.773741 | -1.952476 |
| 24 | 1 | 0 | -3.808685 | -1.851889 | -3.842956 |
| 25 | 1 | 0 | -2.691029 | -3.414391 | -0.007153 |
| 26 | 53 | 0 | 1.853609 | -0.690639 | -0.327581 |
| 27 | 14 | 0 | -3.263797 | 0.860199 | 2.266564 |
| 28 | 6 | 0 | -3.593018 | -0.896706 | 2.839334 |
| 29 | 1 | 0 | -3.225668 | -1.041404 | 3.861644 |
| 30 | 1 | 0 | -4.665455 | -1.123231 | 2.836838 |
| 31 | 1 | 0 | -3.093495 | -1.630051 | 2.197191 |
| 32 | 6 | 0 | -4.205459 | 2.094713 | 3.316587 |
| 33 | 1 | 0 | -4.062616 | 3.111760 | 2.936548 |
| 34 | 1 | 0 | -5.279628 | 1.881500 | 3.311816 |
| 35 | 1 | 0 | -3.859069 | 2.069339 | 4.356235 |
| 36 | 6 | 0 | -1.446136 | 1.292873 | 2.236146 |
| 37 | 1 | 0 | -0.851573 | 0.536423 | 1.712927 |
| 38 | 1 | 0 | -1.303641 | 2.245933 | 1.713902 |
| 39 | 1 | 0 | -1.053444 | 1.395128 | 3.254570 |
| 40 | 6 | 0 | 2.933475 | -2.969528 | 1.375686 |
| 41 | 6 | 0 | 4.583998 | -2.029618 | 0.007457 |
| 42 | 6 | 0 | 4.201737 | -3.759689 | 1.671849 |
| 43 | 6 | 0 | 5.281882 | -3.144876 | 0.776182 |
| 44 | 1 | 0 | 4.417293 | -3.673933 | 2.739901 |
| 45 | 1 | 0 | 4.005107 | -4.813770 | 1.460102 |
| 46 | 1 | 0 | 6.115229 | -2.707890 | 1.332110 |
| 47 | 1 | 0 | 5.702450 | -3.847459 | 0.052314 |
| 48 | 7 | 0 | 3.248910 | -2.007445 | 0.416608 |
| 49 | 8 | 0 | 5.072700 | -1.290191 | -0.807948 |
| 50 | 8 | 0 | 1.844721 | -3.128440 | 1.869317 |
| 51 | 6 | 0 | -4.272402 | -3.949523 | -2.164680 |
| 52 | 1 | 0 | -5.274693 | -3.717700 | -1.784800 |
| 53 | 1 | 0 | -4.367634 | -4.195118 | -3.226216 |
| 54 | 1 | 0 | -3.912424 | -4.837009 | -1.636404 |
| 55 | 6 | 0 | 2.756450 | 6.490201 | -0.271690 |
| 56 | 1 | 0 | 3.782351 | 6.350617 | -0.631768 |

| | | | | | |
|---|---|---|---|---|---|
| 57 | 1 | 0 | 2.325377 | 7.332740 | -0.819860 |
| 58 | 1 | 0 | 2.811789 | 6.762062 | 0.786294 |

---------------------------------------------------------------------

| System: | **2** |
|---|---|
| Oxidant: | NIS |
| Nitrogen-donor: | $TMSN_3$ |
| Stationary point: | TS1 |
| M06–2X/6–31+G(d) energy (in a.u.): | -8470.94572985 |
| Thermal correction to Gibbs Free Energy (in a.u.): | 0.385349 |
| Number of imaginary frequencies: | 1 (–354 $cm^{-1}$) |

**CARTESIAN COORDINATES**

| Center Number | Atomic Number | Atomic Type | Coordinates (Angstroms) X | Y | Z |
|---|---|---|---|---|---|
| 1 | 6 | 0 | -1.104602 | 0.462675 | -0.285578 |
| 2 | 6 | 0 | -0.475047 | -0.643667 | -0.090200 |
| 3 | 6 | 0 | -0.913047 | 1.909529 | -0.299209 |
| 4 | 6 | 0 | -1.506483 | 2.733172 | -1.261946 |
| 5 | 6 | 0 | -0.095833 | 2.493665 | 0.681352 |
| 6 | 6 | 0 | -1.285954 | 4.107450 | -1.246261 |
| 7 | 1 | 0 | -2.139038 | 2.294892 | -2.028899 |
| 8 | 6 | 0 | 0.127050 | 3.864071 | 0.680081 |
| 9 | 1 | 0 | 0.372559 | 1.865644 | 1.432525 |
| 10 | 6 | 0 | -0.464146 | 4.694698 | -0.279898 |
| 11 | 1 | 0 | -1.750577 | 4.732142 | -2.005314 |
| 12 | 1 | 0 | 0.777302 | 4.295446 | 1.437017 |
| 13 | 7 | 0 | -2.910867 | 0.270794 | -0.593638 |
| 14 | 7 | 0 | -3.184072 | -0.629914 | -1.401776 |
| 15 | 7 | 0 | -3.354088 | -1.451035 | -2.151362 |
| 16 | 6 | 0 | -0.958125 | -2.016801 | 0.046627 |
| 17 | 6 | 0 | -0.420365 | -3.056559 | -0.726628 |
| 18 | 6 | 0 | -1.973913 | -2.323705 | 0.962071 |
| 19 | 6 | 0 | -0.924154 | -4.346448 | -0.616506 |
| 20 | 1 | 0 | 0.391128 | -2.842699 | -1.416247 |
| 21 | 6 | 0 | -2.460466 | -3.624417 | 1.076335 |
| 22 | 1 | 0 | -2.366933 | -1.536861 | 1.601990 |
| 23 | 6 | 0 | -1.952032 | -4.655367 | 0.283257 |
| 24 | 1 | 0 | -0.502144 | -5.135981 | -1.234207 |
| 25 | 1 | 0 | -3.242432 | -3.842612 | 1.800780 |
| 26 | 53 | 0 | 1.958213 | -0.396974 | -0.009505 |
| 27 | 14 | 0 | -4.205295 | 0.954042 | 0.502771 |
| 28 | 6 | 0 | -5.380393 | -0.471048 | 0.803662 |
| 29 | 1 | 0 | -6.159021 | -0.171852 | 1.514412 |
| 30 | 1 | 0 | -5.883721 | -0.784374 | -0.118096 |
| 31 | 1 | 0 | -4.861564 | -1.342000 | 1.218212 |
| 32 | 6 | 0 | -5.030787 | 2.352343 | -0.420170 |
| 33 | 1 | 0 | -4.324677 | 3.169489 | -0.600061 |
| 34 | 1 | 0 | -5.425536 | 2.017484 | -1.385836 |
| 35 | 1 | 0 | -5.869748 | 2.750253 | 0.162487 |
| 36 | 6 | 0 | -3.298929 | 1.524828 | 2.026647 |
| 37 | 1 | 0 | -2.570497 | 0.783330 | 2.371836 |
| 38 | 1 | 0 | -2.761812 | 2.459845 | 1.838550 |
| 39 | 1 | 0 | -4.017106 | 1.699318 | 2.836172 |
| 40 | 6 | 0 | 4.822350 | 0.783054 | 0.691890 |
| 41 | 6 | 0 | 5.022026 | -1.180875 | -0.487908 |
| 42 | 6 | 0 | 6.331730 | 0.566049 | 0.558284 |
| 43 | 6 | 0 | 6.464606 | -0.741043 | -0.223918 |
| 44 | 1 | 0 | 6.762315 | 1.431671 | 0.047646 |
| 45 | 1 | 0 | 6.766105 | 0.527033 | 1.560880 |
| 46 | 1 | 0 | 6.974654 | -0.631096 | -1.184659 |
| 47 | 1 | 0 | 6.972997 | -1.535892 | 0.328445 |
| 48 | 7 | 0 | 4.163222 | -0.256110 | 0.070666 |
| 49 | 8 | 0 | 4.691727 | -2.180177 | -1.091205 |
| 50 | 8 | 0 | 4.295357 | 1.721240 | 1.255575 |
| 51 | 6 | 0 | -2.463955 | -6.067782 | 0.406961 |
| 52 | 1 | 0 | -2.736824 | -6.475799 | -0.571630 |
| 53 | 1 | 0 | -1.698412 | -6.724928 | 0.833992 |
| 54 | 1 | 0 | -3.345131 | -6.115840 | 1.052739 |
| 55 | 6 | 0 | -0.195316 | 6.177055 | -0.276767 |
| 56 | 1 | 0 | 0.842182 | 6.380197 | -0.563772 |

| | | | | | |
|---|---|---|---|---|---|
| 57 | 1 | 0 | -0.848330 | 6.702417 | -0.978720 |
| 58 | 1 | 0 | -0.348191 | 6.603916 | 0.719460 |

---------------------------------------------------------------------

| System: | **2** |
|---|---|
| Oxidant: | NIS |
| Nitrogen-donor: | $TMSN_3$ |
| Stationary point: | IN1 |
| M06–2X/6–31+G(d) energy (in a.u.): | -8470.95343535 |
| Thermal correction to Gibbs Free Energy (in a.u.): | 0.386187 |
| Number of imaginary frequencies: | 0 |

**CARTESIAN COORDINATES**

| Center Number | Atomic Number | Atomic Type | Coordinates (Angstroms) X | Y | Z |
|---|---|---|---|---|---|
| 1 | 6 | 0 | -1.190978 | 0.536212 | -0.267568 |
| 2 | 6 | 0 | -0.514127 | -0.604662 | -0.082705 |
| 3 | 6 | 0 | -0.730217 | 1.941203 | -0.302994 |
| 4 | 6 | 0 | -1.214463 | 2.831349 | -1.267484 |
| 5 | 6 | 0 | 0.197171 | 2.413089 | 0.637839 |
| 6 | 6 | 0 | -0.773668 | 4.152794 | -1.303270 |
| 7 | 1 | 0 | -1.928017 | 2.487593 | -2.013993 |
| 8 | 6 | 0 | 0.638385 | 3.728422 | 0.590398 |
| 9 | 1 | 0 | 0.585610 | 1.742413 | 1.396987 |
| 10 | 6 | 0 | 0.163784 | 4.620699 | -0.379768 |
| 11 | 1 | 0 | -1.151552 | 4.823326 | -2.071412 |
| 12 | 1 | 0 | 1.378087 | 4.062324 | 1.313657 |
| 13 | 7 | 0 | -2.685689 | 0.488519 | -0.489762 |
| 14 | 7 | 0 | -3.096626 | -0.342642 | -1.344961 |
| 15 | 7 | 0 | -3.466369 | -1.086444 | -2.093450 |
| 16 | 6 | 0 | -1.189261 | -1.913591 | 0.016687 |
| 17 | 6 | 0 | -0.841583 | -2.965788 | -0.844822 |
| 18 | 6 | 0 | -2.178696 | -2.150726 | 0.980537 |
| 19 | 6 | 0 | -1.496891 | -4.189066 | -0.767787 |
| 20 | 1 | 0 | -0.047509 | -2.815523 | -1.571189 |
| 21 | 6 | 0 | -2.819132 | -3.386351 | 1.062597 |
| 22 | 1 | 0 | -2.425570 | -1.366609 | 1.694046 |
| 23 | 6 | 0 | -2.496583 | -4.423575 | 0.184698 |
| 24 | 1 | 0 | -1.214420 | -4.986908 | -1.450872 |
| 25 | 1 | 0 | -3.570634 | -3.551084 | 1.832130 |
| 26 | 53 | 0 | 1.735606 | -0.632164 | 0.003742 |
| 27 | 14 | 0 | -3.949158 | 1.344147 | 0.598288 |
| 28 | 6 | 0 | -5.278741 | 0.041832 | 0.765061 |
| 29 | 1 | 0 | -6.023666 | 0.373434 | 1.497479 |
| 30 | 1 | 0 | -5.811311 | -0.129211 | -0.177375 |
| 31 | 1 | 0 | -4.872673 | -0.915367 | 1.109570 |
| 32 | 6 | 0 | -4.551982 | 2.841640 | -0.331836 |
| 33 | 1 | 0 | -3.779337 | 3.614415 | -0.382147 |
| 34 | 1 | 0 | -4.853513 | 2.585875 | -1.353973 |
| 35 | 1 | 0 | -5.429050 | 3.261379 | 0.174823 |
| 36 | 6 | 0 | -3.007780 | 1.720683 | 2.155033 |
| 37 | 1 | 0 | -2.380063 | 0.880653 | 2.469554 |
| 38 | 1 | 0 | -2.359179 | 2.591261 | 2.017672 |
| 39 | 1 | 0 | -3.715426 | 1.939237 | 2.963194 |
| 40 | 6 | 0 | 4.771287 | 0.450178 | 0.671850 |
| 41 | 6 | 0 | 4.964449 | -1.544656 | -0.397409 |
| 42 | 6 | 0 | 6.287271 | 0.224568 | 0.579104 |
| 43 | 6 | 0 | 6.417367 | -1.117302 | -0.138467 |
| 44 | 1 | 0 | 6.733437 | 1.063080 | 0.036685 |
| 45 | 1 | 0 | 6.704352 | 0.229775 | 1.590217 |
| 46 | 1 | 0 | 6.940652 | -1.059131 | -1.097044 |
| 47 | 1 | 0 | 6.908788 | -1.892493 | 0.456256 |
| 48 | 7 | 0 | 4.104770 | -0.599827 | 0.100985 |
| 49 | 8 | 0 | 4.643727 | -2.574463 | -0.965439 |
| 50 | 8 | 0 | 4.254431 | 1.431944 | 1.184745 |
| 51 | 6 | 0 | -3.167341 | -5.770112 | 0.278560 |
| 52 | 1 | 0 | -3.513084 | -6.109231 | -0.703120 |
| 53 | 1 | 0 | -2.471261 | -6.525484 | 0.659460 |
| 54 | 1 | 0 | -4.028819 | -5.739516 | 0.951392 |
| 55 | 6 | 0 | 0.677271 | 6.036115 | -0.430757 |
| 56 | 1 | 0 | 1.735665 | 6.050374 | -0.712240 |

| | | | | | |
|---|---|---|---|---|---|
| 57 | 1 | 0 | 0.125158 | 6.635289 | -1.159857 |
| 58 | 1 | 0 | 0.593312 | 6.521959 | 0.546595 |

---------------------------------------------------------------------

| System: | **2** |
|---|---|
| Oxidant: | NIS |
| Nitrogen-donor: | $TMSN_3$ |
| Stationary point: | TS2 |
| M06–2X/6–31+G(d) energy (in a.u.): | -8470.98093101 |
| Thermal correction to Gibbs Free Energy (in a.u.): | 0.395126 |
| Number of imaginary frequencies: | 1 (–32 $cm^{-1}$) |

**CARTESIAN COORDINATES**

| Center Number | Atomic Number | Atomic Type | Coordinates (Angstroms) X | Y | Z |
|---|---|---|---|---|---|
| 1 | 6 | 0 | -0.002147 | 0.583481 | -0.579161 |
| 2 | 6 | 0 | 1.153092 | 1.078467 | -0.106558 |
| 3 | 6 | 0 | -1.350751 | 1.188794 | -0.600064 |
| 4 | 6 | 0 | -2.014575 | 1.345052 | -1.818915 |
| 5 | 6 | 0 | -1.987841 | 1.584118 | 0.581916 |
| 6 | 6 | 0 | -3.291964 | 1.896082 | -1.861449 |
| 7 | 1 | 0 | -1.531483 | 1.030089 | -2.742000 |
| 8 | 6 | 0 | -3.268219 | 2.121528 | 0.529827 |
| 9 | 1 | 0 | -1.474199 | 1.449269 | 1.528695 |
| 10 | 6 | 0 | -3.940688 | 2.286878 | -0.687838 |
| 11 | 1 | 0 | -3.794203 | 2.015636 | -2.818035 |
| 12 | 1 | 0 | -3.760459 | 2.417131 | 1.453685 |
| 13 | 7 | 0 | 0.012169 | -0.728792 | -1.231141 |
| 14 | 7 | 0 | 0.947663 | -0.874177 | -2.068091 |
| 15 | 7 | 0 | 1.770757 | -1.046529 | -2.803781 |
| 16 | 6 | 0 | 2.424382 | 0.322969 | -0.032628 |
| 17 | 6 | 0 | 3.612836 | 0.848138 | -0.551069 |
| 18 | 6 | 0 | 2.438901 | -0.967204 | 0.519572 |
| 19 | 6 | 0 | 4.783032 | 0.094518 | -0.537673 |
| 20 | 1 | 0 | 3.619767 | 1.850541 | -0.970896 |
| 21 | 6 | 0 | 3.615113 | -1.708472 | 0.529845 |
| 22 | 1 | 0 | 1.524916 | -1.365384 | 0.950660 |
| 23 | 6 | 0 | 4.805273 | -1.194447 | 0.002207 |
| 24 | 1 | 0 | 5.694589 | 0.516995 | -0.952992 |
| 25 | 1 | 0 | 3.609417 | -2.705652 | 0.964620 |
| 26 | 53 | 0 | 1.211286 | 3.064671 | 0.608732 |
| 27 | 14 | 0 | -1.191276 | -2.292306 | -1.167576 |
| 28 | 6 | 0 | -0.100577 | -3.758400 | -0.704037 |
| 29 | 1 | 0 | -0.492899 | -4.647121 | -1.211591 |
| 30 | 1 | 0 | 0.904562 | -3.577644 | -1.113048 |
| 31 | 1 | 0 | -0.019576 | -3.965263 | 0.361138 |
| 32 | 6 | 0 | -1.068134 | -2.540229 | -3.086033 |
| 33 | 1 | 0 | -1.285275 | -1.631244 | -3.664091 |
| 34 | 1 | 0 | -0.130489 | -2.970569 | -3.458437 |
| 35 | 1 | 0 | -1.856242 | -3.261036 | -3.345838 |
| 36 | 6 | 0 | -2.987042 | -1.727514 | -1.144306 |
| 37 | 1 | 0 | -3.607466 | -2.629173 | -1.115152 |
| 38 | 1 | 0 | -3.244170 | -1.100247 | -0.288742 |
| 39 | 1 | 0 | -3.203604 | -1.186554 | -2.070128 |
| 40 | 6 | 0 | -2.133459 | -2.960562 | 1.550859 |
| 41 | 6 | 0 | -0.893673 | -1.212735 | 2.203442 |
| 42 | 6 | 0 | -2.390139 | -2.893310 | 3.054285 |
| 43 | 6 | 0 | -1.577965 | -1.683248 | 3.491260 |
| 44 | 1 | 0 | -3.466339 | -2.800985 | 3.224435 |
| 45 | 1 | 0 | -2.066901 | -3.834219 | 3.508648 |
| 46 | 1 | 0 | -2.186247 | -0.858903 | 3.876246 |
| 47 | 1 | 0 | -0.810980 | -1.898779 | 4.239721 |
| 48 | 7 | 0 | -1.296418 | -1.954419 | 1.122399 |

| | | | | | |
|---|---|---|---|---|---|
| 49 | 8 | 0 | -0.090381 | -0.287545 | 2.190525 |
| 50 | 8 | 0 | -2.607817 | -3.820130 | 0.818014 |
| 51 | 6 | 0 | -5.328629 | 2.873249 | -0.718563 |
| 52 | 1 | 0 | -6.019677 | 2.272913 | -0.117577 |
| 53 | 1 | 0 | -5.333185 | 3.888465 | -0.307723 |
| 54 | 1 | 0 | -5.718827 | 2.918816 | -1.738624 |
| 55 | 6 | 0 | 6.077631 | -2.000524 | 0.046941 |
| 56 | 1 | 0 | 6.820985 | -1.609360 | -0.652919 |
| 57 | 1 | 0 | 6.517122 | -1.974678 | 1.050546 |
| 58 | 1 | 0 | 5.890195 | -3.049273 | -0.202470 |

---------------------------------------------------------------------

| System: | **2** |
|---|---|
| Oxidant: | NIS |
| Nitrogen-donor: | $TMSN_3$ |
| Stationary point: | IN2 |
| M06–2X/6–31+G(d) energy (in a.u.): | -7701.91227979 |
| Thermal correction to Gibbs Free Energy (in a.u.): | 0.213655 |
| Number of imaginary frequencies: | 0 |

**CARTESIAN COORDINATES**

| Center Number | Atomic Number | Atomic Type | Coordinates (Angstroms) X | Y | Z |
|---|---|---|---|---|---|
| 1 | 6 | 0 | -0.471283 | 0.929932 | 0.012851 |
| 2 | 6 | 0 | 0.403493 | -0.100727 | 0.016084 |
| 3 | 6 | 0 | -1.949338 | 0.839579 | -0.089053 |
| 4 | 6 | 0 | -2.753056 | 1.518459 | 0.831979 |
| 5 | 6 | 0 | -2.562229 | 0.126384 | -1.121376 |
| 6 | 6 | 0 | -4.138579 | 1.452306 | 0.741644 |
| 7 | 1 | 0 | -2.284655 | 2.095570 | 1.624511 |
| 8 | 6 | 0 | -3.949978 | 0.074825 | -1.214055 |
| 9 | 1 | 0 | -1.950233 | -0.385542 | -1.858785 |
| 10 | 6 | 0 | -4.759861 | 0.730361 | -0.283368 |
| 11 | 1 | 0 | -4.749667 | 1.972931 | 1.475527 |
| 12 | 1 | 0 | -4.411522 | -0.481948 | -2.026010 |
| 13 | 7 | 0 | -0.059922 | 2.295484 | 0.085638 |
| 14 | 7 | 0 | 0.982498 | 2.563276 | 0.703727 |
| 15 | 7 | 0 | 1.894583 | 2.948366 | 1.241913 |
| 16 | 6 | 0 | 1.870220 | 0.042599 | -0.122337 |
| 17 | 6 | 0 | 2.763589 | -0.491719 | 0.815573 |
| 18 | 6 | 0 | 2.389922 | 0.737340 | -1.220021 |
| 19 | 6 | 0 | 4.132481 | -0.311460 | 0.667101 |
| 20 | 1 | 0 | 2.378478 | -1.047628 | 1.666759 |
| 21 | 6 | 0 | 3.764915 | 0.909906 | -1.363435 |
| 22 | 1 | 0 | 1.707747 | 1.144342 | -1.962609 |
| 23 | 6 | 0 | 4.658047 | 0.393217 | -0.423025 |
| 24 | 1 | 0 | 4.810140 | -0.726422 | 1.409926 |
| 25 | 1 | 0 | 4.147564 | 1.451119 | -2.225471 |
| 26 | 53 | 0 | -0.277203 | -2.102885 | 0.244715 |
| 27 | 6 | 0 | 6.145670 | 0.589201 | -0.563255 |
| 28 | 1 | 0 | 6.522439 | 1.273439 | 0.205185 |
| 29 | 1 | 0 | 6.681135 | -0.358989 | -0.450638 |
| 30 | 1 | 0 | 6.399962 | 1.009492 | -1.540116 |
| 31 | 6 | 0 | -6.262850 | 0.692128 | -0.392533 |
| 32 | 1 | 0 | -6.727055 | 0.565306 | 0.590287 |
| 33 | 1 | 0 | -6.645917 | 1.626122 | -0.819379 |
| 34 | 1 | 0 | -6.593748 | -0.128728 | -1.034665 |

| System: | **2** |
|---|---|
| Oxidant: | NIS |
| Nitrogen-donor: | $TMSN_3$ |
| Stationary point: | TS3 |
| M06–2X/6–31+G(d) energy (in a.u.): | -7701.86496209 |
| Thermal correction to Gibbs Free Energy (in a.u.): | 0.210699 |
| Number of imaginary frequencies: | 1 (–567 $cm^{-1}$) |

**CARTESIAN COORDINATES**

---------------------------------------------------------------------

| Center Number | Atomic Number | Atomic Type | Coordinates (Angstroms) X | Y | Z |
|---|---|---|---|---|---|
| 1 | 6 | 0 | 0.167006 | -1.165495 | 0.259616 |
| 2 | 6 | 0 | -0.898575 | -0.302766 | 0.036020 |
| 3 | 6 | 0 | 1.581579 | -0.768342 | 0.024592 |
| 4 | 6 | 0 | 2.591971 | -1.186153 | 0.896137 |
| 5 | 6 | 0 | 1.925243 | -0.011201 | -1.099633 |
| 6 | 6 | 0 | 3.917045 | -0.836051 | 0.654357 |
| 7 | 1 | 0 | 2.326194 | -1.763063 | 1.778629 |
| 8 | 6 | 0 | 3.251897 | 0.329772 | -1.337328 |
| 9 | 1 | 0 | 1.145116 | 0.312218 | -1.784438 |
| 10 | 6 | 0 | 4.268682 | -0.074523 | -0.465332 |
| 11 | 1 | 0 | 4.691575 | -1.153962 | 1.348826 |
| 12 | 1 | 0 | 3.505121 | 0.920733 | -2.214260 |
| 13 | 6 | 0 | -0.777065 | 1.158285 | 0.164488 |
| 14 | 6 | 0 | -1.450607 | 2.029571 | -0.706221 |
| 15 | 6 | 0 | 0.054412 | 1.714585 | 1.146891 |
| 16 | 6 | 0 | -1.262382 | 3.401871 | -0.617853 |
| 17 | 1 | 0 | -2.112069 | 1.621958 | -1.465419 |
| 18 | 6 | 0 | 0.225051 | 3.092459 | 1.236762 |
| 19 | 1 | 0 | 0.564399 | 1.060059 | 1.848086 |
| 20 | 6 | 0 | -0.425403 | 3.959871 | 0.356801 |
| 21 | 1 | 0 | -1.780762 | 4.056986 | -1.314674 |
| 22 | 1 | 0 | 0.872912 | 3.499345 | 2.009489 |
| 23 | 7 | 0 | -0.182271 | -2.291807 | 0.878645 |
| 24 | 7 | 0 | 0.812644 | -3.583065 | 0.299035 |
| 25 | 53 | 0 | -2.854957 | -1.063779 | -0.243282 |
| 26 | 7 | 0 | 1.207610 | -4.589563 | 0.530637 |
| 27 | 6 | 0 | -0.266133 | 5.454077 | 0.466796 |
| 28 | 1 | 0 | -0.147579 | 5.912563 | -0.519949 |
| 29 | 1 | 0 | -1.148306 | 5.905991 | 0.934849 |
| 30 | 1 | 0 | 0.604942 | 5.717635 | 1.072773 |
| 31 | 6 | 0 | 5.708662 | 0.278205 | -0.737296 |
| 32 | 1 | 0 | 5.786327 | 1.179948 | -1.351280 |
| 33 | 1 | 0 | 6.257142 | 0.449651 | 0.193676 |
| 34 | 1 | 0 | 6.213521 | -0.533721 | -1.273345 |

---------------------------------------------------------------------

| System: | **2** |
|---|---|
| Oxidant: | NIS |
| Nitrogen-donor: | $TMSN_3$ |
| Stationary point: | IN3 |
| M06–2X/6–31+G(d) energy (in a.u.): | -7592.48200169 |
| Thermal correction to Gibbs Free Energy (in a.u.): | 0.206497 |
| Number of imaginary frequencies: | 0 |

**CARTESIAN COORDINATES**

| Center Number | Atomic Number | Atomic Type | X | Y | Z |
|---|---|---|---|---|---|
| 1 | 6 | 0 | 0.823446 | 0.049971 | 0.895099 |
| 2 | 6 | 0 | -0.520675 | 0.448978 | 0.595613 |
| 3 | 7 | 0 | 0.149227 | 0.387348 | 1.914797 |
| 4 | 6 | 0 | 2.126623 | -0.395038 | 0.460141 |
| 5 | 6 | 0 | 2.352352 | -0.639513 | -0.896664 |
| 6 | 6 | 0 | 3.158392 | -0.576203 | 1.390561 |
| 7 | 6 | 0 | 3.607347 | -1.065207 | -1.319930 |
| 8 | 1 | 0 | 1.543194 | -0.492602 | -1.607433 |
| 9 | 6 | 0 | 4.404650 | -1.001315 | 0.955153 |
| 10 | 1 | 0 | 2.969009 | -0.381191 | 2.442465 |
| 11 | 6 | 0 | 4.647871 | -1.249449 | -0.404315 |
| 12 | 1 | 0 | 3.783834 | -1.256947 | -2.374962 |
| 13 | 1 | 0 | 5.206787 | -1.144704 | 1.674917 |
| 14 | 6 | 0 | -1.640039 | -0.497068 | 0.292348 |
| 15 | 6 | 0 | -2.178135 | -0.624414 | -0.992031 |
| 16 | 6 | 0 | -2.137096 | -1.302695 | 1.316398 |
| 17 | 6 | 0 | -3.187615 | -1.546091 | -1.240496 |
| 18 | 1 | 0 | -1.813204 | 0.009752 | -1.795911 |
| 19 | 6 | 0 | -3.156665 | -2.218921 | 1.059713 |
| 20 | 1 | 0 | -1.722383 | -1.208989 | 2.316283 |
| 21 | 6 | 0 | -3.697457 | -2.357063 | -0.218911 |
| 22 | 1 | 0 | -3.594890 | -1.635292 | -2.245359 |
| 23 | 1 | 0 | -3.537211 | -2.834310 | 1.871537 |
| 24 | 53 | 0 | -0.803884 | 2.459915 | -0.229305 |
| 25 | 6 | 0 | 6.015619 | -1.683338 | -0.863068 |
| 26 | 1 | 0 | 6.703978 | -0.830576 | -0.878290 |
| 27 | 1 | 0 | 6.437761 | -2.434771 | -0.189144 |
| 28 | 1 | 0 | 5.982467 | -2.104843 | -1.871065 |
| 29 | 6 | 0 | -4.804308 | -3.340327 | -0.504033 |
| 30 | 1 | 0 | -5.050436 | -3.929208 | 0.383689 |
| 31 | 1 | 0 | -5.714231 | -2.823394 | -0.828057 |
| 32 | 1 | 0 | -4.519281 | -4.033696 | -1.302531 |

| System: | **2** |
|---|---|
| Oxidant: | NIS |
| Nitrogen-donor: | $TMSN_3$ |
| Stationary point: | TS4 |
| M06–2X/6–31+G(d) energy (in a.u.): | -8165.78206794 |
| Thermal correction to Gibbs Free Energy (in a.u.): | 0.318230 |
| Number of imaginary frequencies: | 1 (–231 $cm^{-1}$) |

**CARTESIAN COORDINATES**

| Center Number | Atomic Number | Atomic Type | Coordinates (Angstroms) X | Y | Z |
|---|---|---|---|---|---|
| 1 | 6 | 0 | -0.611780 | -0.808434 | -1.313181 |
| 2 | 6 | 0 | 0.758316 | -1.014722 | -1.361173 |
| 3 | 7 | 0 | 0.097063 | -0.832331 | -2.482505 |
| 4 | 6 | 0 | -1.906669 | -1.268480 | -0.877548 |
| 5 | 6 | 0 | -2.129990 | -1.587646 | 0.466324 |
| 6 | 6 | 0 | -2.941194 | -1.368235 | -1.822185 |
| 7 | 6 | 0 | -3.389987 | -2.037310 | 0.851627 |
| 8 | 1 | 0 | -1.339425 | -1.436193 | 1.198634 |
| 9 | 6 | 0 | -4.186787 | -1.816703 | -1.417887 |
| 10 | 1 | 0 | -2.750843 | -1.099797 | -2.857924 |
| 11 | 6 | 0 | -4.427957 | -2.157819 | -0.075345 |
| 12 | 1 | 0 | -3.569886 | -2.277214 | 1.895533 |
| 13 | 1 | 0 | -4.991536 | -1.904271 | -2.143397 |
| 14 | 6 | 0 | 2.079312 | -1.314492 | -0.911932 |
| 15 | 6 | 0 | 2.351290 | -1.503576 | 0.448659 |
| 16 | 6 | 0 | 3.104132 | -1.407778 | -1.873225 |
| 17 | 6 | 0 | 3.656770 | -1.778843 | 0.841121 |
| 18 | 1 | 0 | 1.560868 | -1.379914 | 1.184416 |
| 19 | 6 | 0 | 4.393216 | -1.687148 | -1.462500 |
| 20 | 1 | 0 | 2.871207 | -1.256193 | -2.924060 |
| 21 | 6 | 0 | 4.687393 | -1.872779 | -0.097965 |
| 22 | 1 | 0 | 3.875740 | -1.905310 | 1.897286 |
| 23 | 1 | 0 | 5.192910 | -1.759849 | -2.195033 |
| 24 | 53 | 0 | 0.192215 | 0.762779 | 2.396921 |
| 25 | 7 | 0 | -0.964710 | 1.292907 | -1.025706 |
| 26 | 7 | 0 | -1.965805 | 1.461456 | -0.322972 |
| 27 | 7 | 0 | -2.903294 | 1.512533 | 0.297366 |
| 28 | 14 | 0 | 0.199182 | 2.682154 | -1.230224 |
| 29 | 6 | 0 | -0.052242 | 3.229461 | -3.006704 |
| 30 | 1 | 0 | -1.069078 | 3.601021 | -3.172330 |
| 31 | 1 | 0 | 0.648171 | 4.029971 | -3.271820 |
| 32 | 1 | 0 | 0.117181 | 2.391885 | -3.693046 |
| 33 | 6 | 0 | 1.939508 | 2.055787 | -0.991199 |
| 34 | 1 | 0 | 2.610391 | 2.923846 | -0.965876 |
| 35 | 1 | 0 | 2.036614 | 1.523818 | -0.038535 |
| 36 | 1 | 0 | 2.270588 | 1.412896 | -1.813925 |
| 37 | 6 | 0 | -0.301915 | 3.992226 | -0.000735 |
| 38 | 1 | 0 | -0.231923 | 3.596895 | 1.019029 |
| 39 | 1 | 0 | 0.368477 | 4.855363 | -0.086150 |
| 40 | 1 | 0 | -1.324085 | 4.347591 | -0.174106 |
| 41 | 6 | 0 | -5.796636 | -2.620956 | 0.346876 |
| 42 | 1 | 0 | -6.520878 | -1.803470 | 0.260950 |
| 43 | 1 | 0 | -6.147428 | -3.439107 | -0.290246 |
| 44 | 1 | 0 | -5.798001 | -2.966098 | 1.383283 |
| 45 | 6 | 0 | 6.101576 | -2.161261 | 0.327176 |
| 46 | 1 | 0 | 6.478115 | -3.063862 | -0.165679 |
| 47 | 1 | 0 | 6.763795 | -1.335007 | 0.047204 |
| 48 | 1 | 0 | 6.172153 | -2.304164 | 1.407632 |

---------------------------------------------------------------------

| System: | **2** |
|---|---|
| Oxidant: | NIS |
| Nitrogen-donor: | $TMSN_3$ |
| Stationary point: | IN4 |
| M06–2X/6–31+G(d) energy (in a.u.): | -1245.66316522 |
| Thermal correction to Gibbs Free Energy (in a.u.): | 0.323932 |
| Number of imaginary frequencies: | 0 |

**CARTESIAN COORDINATES**

---------------------------------------------------------------------

| Center Number | Atomic Number | Atomic Type | Coordinates (Angstroms) X | Y | Z |
|---|---|---|---|---|---|
| 1 | 6 | 0 | 0.384923 | 0.156415 | 0.269807 |
| 2 | 6 | 0 | -1.003736 | 0.238686 | 0.662063 |
| 3 | 7 | 0 | -0.183199 | 0.602627 | 1.558750 |
| 4 | 6 | 0 | 1.195298 | -1.093320 | 0.162518 |
| 5 | 6 | 0 | 0.775731 | -2.219888 | 0.881573 |
| 6 | 6 | 0 | 2.347533 | -1.183192 | -0.619507 |
| 7 | 6 | 0 | 1.501455 | -3.401208 | 0.816270 |
| 8 | 1 | 0 | -0.116286 | -2.170504 | 1.500672 |
| 9 | 6 | 0 | 3.067320 | -2.375992 | -0.680429 |
| 10 | 1 | 0 | 2.713000 | -0.327771 | -1.183596 |
| 11 | 6 | 0 | 2.659871 | -3.503123 | 0.034102 |
| 12 | 1 | 0 | 1.163788 | -4.264576 | 1.384179 |
| 13 | 1 | 0 | 3.962811 | -2.425527 | -1.293985 |
| 14 | 6 | 0 | -2.392621 | 0.010653 | 0.362937 |
| 15 | 6 | 0 | -2.751876 | -0.579022 | -0.854605 |
| 16 | 6 | 0 | -3.378624 | 0.371206 | 1.295593 |
| 17 | 6 | 0 | -4.092746 | -0.806017 | -1.138454 |
| 18 | 1 | 0 | -1.980141 | -0.872444 | -1.562840 |
| 19 | 6 | 0 | -4.710791 | 0.141145 | 0.996677 |
| 20 | 1 | 0 | -3.088467 | 0.824279 | 2.239497 |
| 21 | 6 | 0 | -5.088803 | -0.449187 | -0.221546 |
| 22 | 1 | 0 | -4.373672 | -1.268473 | -2.080147 |
| 23 | 1 | 0 | -5.479206 | 0.418766 | 1.713403 |
| 24 | 7 | 0 | 0.948942 | 1.314775 | -0.500655 |
| 25 | 7 | 0 | 0.726540 | 1.300406 | -1.742597 |
| 26 | 7 | 0 | 0.537662 | 1.292012 | -2.843281 |
| 27 | 14 | 0 | 2.035779 | 2.655570 | 0.308213 |
| 28 | 6 | 0 | 3.073584 | 1.661290 | 1.479698 |
| 29 | 1 | 0 | 3.748065 | 0.976581 | 0.957282 |
| 30 | 1 | 0 | 3.681969 | 2.340834 | 2.087999 |
| 31 | 1 | 0 | 2.444076 | 1.079503 | 2.161178 |
| 32 | 6 | 0 | 0.834488 | 3.824777 | 1.105237 |
| 33 | 1 | 0 | 1.376343 | 4.675534 | 1.534759 |
| 34 | 1 | 0 | 0.113495 | 4.221611 | 0.383185 |
| 35 | 1 | 0 | 0.287396 | 3.329412 | 1.912706 |
| 36 | 6 | 0 | 2.917354 | 3.342992 | -1.182224 |
| 37 | 1 | 0 | 2.240211 | 3.847343 | -1.880549 |
| 38 | 1 | 0 | 3.644610 | 4.093982 | -0.852534 |
| 39 | 1 | 0 | 3.478235 | 2.573540 | -1.724760 |
| 40 | 6 | 0 | 3.430793 | -4.795234 | -0.025832 |
| 41 | 1 | 0 | 4.309340 | -4.705923 | -0.668901 |
| 42 | 1 | 0 | 3.768118 | -5.093685 | 0.971817 |
| 43 | 1 | 0 | 2.804755 | -5.603986 | -0.416184 |
| 44 | 6 | 0 | -6.542665 | -0.695015 | -0.518037 |
| 45 | 1 | 0 | -6.970107 | -1.392866 | 0.209571 |
| 46 | 1 | 0 | -7.114170 | 0.236233 | -0.451941 |
| 47 | 1 | 0 | -6.683619 | -1.114374 | -1.516367 |

----------------------------------------------------------------------

| System: | **2** |
|---|---|
| Oxidant: | NIS |
| Nitrogen-donor: | $TMSN_3$ |
| Stationary point: | TS5 |
| M06–2X/6–31+G(d) energy (in a.u.): | -1605.79574355 |
| Thermal correction to Gibbs Free Energy (in a.u.): | 0.399005 |
| Number of imaginary frequencies: | 1 (–58 $cm^{-1}$) |

**CARTESIAN COORDINATES**

| Center Number | Atomic Number | Atomic Type | Coordinates (Angstroms) X | Y | Z |
|---|---|---|---|---|---|
| 1 | 6 | 0 | -0.888639 | -0.535968 | 0.394353 |
| 2 | 6 | 0 | -0.803976 | 0.855049 | 0.792733 |
| 3 | 7 | 0 | -0.690106 | 0.033195 | 1.747856 |
| 4 | 6 | 0 | -2.171132 | -1.227277 | 0.054915 |
| 5 | 6 | 0 | -2.209843 | -2.443246 | -0.628153 |
| 6 | 6 | 0 | -3.380830 | -0.632760 | 0.436096 |
| 7 | 6 | 0 | -3.430358 | -3.051340 | -0.920853 |
| 8 | 1 | 0 | -1.288803 | -2.938983 | -0.923273 |
| 9 | 6 | 0 | -4.590996 | -1.246865 | 0.142962 |
| 10 | 1 | 0 | -3.373999 | 0.314831 | 0.968860 |
| 11 | 6 | 0 | -4.638760 | -2.467346 | -0.542149 |
| 12 | 1 | 0 | -3.436900 | -4.001231 | -1.449587 |
| 13 | 1 | 0 | -5.519232 | -0.770033 | 0.449950 |
| 14 | 6 | 0 | -0.994472 | 2.262638 | 0.518427 |
| 15 | 6 | 0 | -1.081584 | 2.725558 | -0.797140 |
| 16 | 6 | 0 | -1.126155 | 3.151365 | 1.589535 |
| 17 | 6 | 0 | -1.305253 | 4.074103 | -1.034167 |
| 18 | 1 | 0 | -0.945291 | 2.028148 | -1.618086 |
| 19 | 6 | 0 | -1.346782 | 4.500309 | 1.338433 |
| 20 | 1 | 0 | -1.059459 | 2.777334 | 2.607713 |
| 21 | 6 | 0 | -1.438041 | 4.979770 | 0.026707 |
| 22 | 1 | 0 | -1.368449 | 4.437716 | -2.056727 |
| 23 | 1 | 0 | -1.451316 | 5.193042 | 2.169462 |
| 24 | 7 | 0 | 0.320860 | -1.198868 | -0.138981 |
| 25 | 7 | 0 | 0.510816 | -1.026166 | -1.371759 |
| 26 | 7 | 0 | 0.671494 | -0.942281 | -2.471714 |
| 27 | 14 | 0 | 1.647923 | -2.099992 | 0.916230 |
| 28 | 6 | 0 | 2.430734 | -3.350002 | -0.234101 |
| 29 | 1 | 0 | 2.736016 | -2.920539 | -1.190437 |
| 30 | 1 | 0 | 3.348934 | -3.724930 | 0.230329 |
| 31 | 1 | 0 | 1.745392 | -4.190380 | -0.393527 |
| 32 | 6 | 0 | 0.358842 | -3.091069 | 1.895806 |
| 33 | 1 | 0 | 0.901693 | -3.782033 | 2.555501 |
| 34 | 1 | 0 | -0.264210 | -2.454898 | 2.533760 |
| 35 | 1 | 0 | -0.300007 | -3.694926 | 1.262085 |
| 36 | 6 | 0 | 2.512062 | -1.082055 | 2.228525 |
| 37 | 1 | 0 | 2.198083 | -0.036362 | 2.153649 |
| 38 | 1 | 0 | 2.244528 | -1.453710 | 3.223308 |
| 39 | 1 | 0 | 3.595987 | -1.129167 | 2.092127 |
| 40 | 7 | 0 | 2.975648 | -0.437330 | -0.437159 |
| 41 | 6 | 0 | 4.340569 | -0.603959 | -0.421911 |
| 42 | 6 | 0 | 5.065210 | 0.692480 | -0.807443 |
| 43 | 6 | 0 | 3.932174 | 1.703575 | -0.943820 |
| 44 | 6 | 0 | 2.677628 | 0.861733 | -0.694772 |
| 45 | 8 | 0 | 1.535353 | 1.335015 | -0.733159 |
| 46 | 1 | 0 | 3.966792 | 2.508671 | -0.203527 |
| 47 | 1 | 0 | 5.796021 | 0.935783 | -0.031639 |
| 48 | 8 | 0 | 4.916984 | -1.638427 | -0.118349 |

| | | | | | |
|---|---|---|---|---|---|
| 49 | 1 | 0 | 3.858143 | 2.170766 | -1.929938 |
| 50 | 1 | 0 | 5.618134 | 0.523064 | -1.736152 |
| 51 | 6 | 0 | -5.961389 | -3.119268 | -0.854633 |
| 52 | 1 | 0 | -6.525273 | -3.321017 | 0.062136 |
| 53 | 1 | 0 | -6.579557 | -2.470752 | -1.484487 |
| 54 | 1 | 0 | -5.821644 | -4.067173 | -1.380804 |
| 55 | 6 | 0 | -1.648444 | 6.446125 | -0.248108 |
| 56 | 1 | 0 | -2.368558 | 6.597065 | -1.057858 |
| 57 | 1 | 0 | -2.013684 | 6.969654 | 0.639214 |
| 58 | 1 | 0 | -0.707314 | 6.918075 | -0.552335 |

---------------------------------------------------------------------

| System: | **2** |
|---|---|
| Oxidant: | NIS |
| Nitrogen-donor: | $TMSN_3$ |
| Stationary point: | IN5 |
| M06–2X/6–31+G(d) energy (in a.u.): | -836.704529787 |
| Thermal correction to Gibbs Free Energy (in a.u.): | 0.220562 |
| Number of imaginary frequencies: | 0 |

**CARTESIAN COORDINATES**

| Center Number | Atomic Number | Atomic Type | Coordinates (Angstroms) X | Y | Z |
|---|---|---|---|---|---|
| 1 | 6 | 0 | 0.591456 | 0.739856 | 0.615241 |
| 2 | 6 | 0 | -0.720805 | 1.223178 | 0.248608 |
| 3 | 7 | 0 | -0.013062 | 1.319482 | 1.566590 |
| 4 | 6 | 0 | 1.821256 | 0.040525 | 0.297489 |
| 5 | 6 | 0 | 1.992909 | -0.511254 | -0.973713 |
| 6 | 6 | 0 | 2.828439 | -0.090073 | 1.262959 |
| 7 | 6 | 0 | 3.167832 | -1.192367 | -1.277649 |
| 8 | 1 | 0 | 1.203167 | -0.403986 | -1.712917 |
| 9 | 6 | 0 | 3.994575 | -0.770803 | 0.947147 |
| 10 | 1 | 0 | 2.683680 | 0.346619 | 2.247518 |
| 11 | 6 | 0 | 4.181211 | -1.332498 | -0.325393 |
| 12 | 1 | 0 | 3.300976 | -1.621193 | -2.267410 |
| 13 | 1 | 0 | 4.778555 | -0.873087 | 1.693893 |
| 14 | 6 | 0 | -1.903571 | 0.318578 | 0.131195 |
| 15 | 6 | 0 | -2.956828 | 0.594438 | -0.745440 |
| 16 | 6 | 0 | -1.961176 | -0.840504 | 0.908687 |
| 17 | 6 | 0 | -4.036063 | -0.278715 | -0.840827 |
| 18 | 1 | 0 | -2.928948 | 1.495440 | -1.348840 |
| 19 | 6 | 0 | -3.047339 | -1.705621 | 0.808325 |
| 20 | 1 | 0 | -1.157894 | -1.066780 | 1.604863 |
| 21 | 6 | 0 | -4.101557 | -1.442674 | -0.068820 |
| 22 | 1 | 0 | -4.847700 | -0.048264 | -1.528171 |
| 23 | 1 | 0 | -3.074004 | -2.600831 | 1.425177 |
| 24 | 7 | 0 | -0.900576 | 2.446902 | -0.526061 |
| 25 | 7 | 0 | 0.011092 | 3.268045 | -0.414913 |
| 26 | 7 | 0 | 0.804825 | 4.070745 | -0.368979 |
| 27 | 6 | 0 | 5.449470 | -2.081433 | -0.642659 |
| 28 | 1 | 0 | 6.331240 | -1.494016 | -0.368085 |
| 29 | 1 | 0 | 5.493714 | -3.021873 | -0.082215 |
| 30 | 1 | 0 | 5.515237 | -2.320199 | -1.707171 |
| 31 | 6 | 0 | -5.276335 | -2.380162 | -0.190988 |
| 32 | 1 | 0 | -6.219329 | -1.857678 | 0.001846 |
| 33 | 1 | 0 | -5.337567 | -2.805163 | -1.198900 |
| 34 | 1 | 0 | -5.197935 | -3.208033 | 0.518980 |

| System: | **2** |
|---|---|
| Oxidant: | NIS |
| Nitrogen-donor: | $TMSN_3$ |
| Stationary point: | TS6 |
| M06–2X/6–31+G(d) energy (in a.u.): | -836.643853191 |
| Thermal correction to Gibbs Free Energy (in a.u.): | 0.214732 |
| Number of imaginary frequencies: | 1 (–625 $cm^{-1}$) |

**CARTESIAN COORDINATES**

| Center Number | Atomic Number | Atomic Type | Coordinates (Angstroms) X | Y | Z |
|---|---|---|---|---|---|
| 1 | 6 | 0 | 0.560162 | 0.660352 | 1.236226 |
| 2 | 6 | 0 | -0.696997 | 1.318698 | 0.759147 |
| 3 | 7 | 0 | -0.053445 | 1.067169 | 2.226088 |
| 4 | 6 | 0 | 1.707788 | -0.018999 | 0.667126 |
| 5 | 6 | 0 | 1.670596 | -0.402820 | -0.675818 |
| 6 | 6 | 0 | 2.842085 | -0.276472 | 1.445279 |
| 7 | 6 | 0 | 2.767403 | -1.048747 | -1.235628 |
| 8 | 1 | 0 | 0.777231 | -0.204086 | -1.263658 |
| 9 | 6 | 0 | 3.931117 | -0.917070 | 0.870520 |
| 10 | 1 | 0 | 2.859111 | 0.028534 | 2.487749 |
| 11 | 6 | 0 | 3.911899 | -1.309567 | -0.475123 |
| 12 | 1 | 0 | 2.736335 | -1.355273 | -2.278029 |
| 13 | 1 | 0 | 4.813936 | -1.120317 | 1.471801 |
| 14 | 6 | 0 | -1.843983 | 0.416227 | 0.408215 |
| 15 | 6 | 0 | -2.667374 | 0.755247 | -0.670637 |
| 16 | 6 | 0 | -2.109344 | -0.765141 | 1.106530 |
| 17 | 6 | 0 | -3.725182 | -0.069003 | -1.039521 |
| 18 | 1 | 0 | -2.473525 | 1.679567 | -1.206726 |
| 19 | 6 | 0 | -3.177020 | -1.580140 | 0.734529 |
| 20 | 1 | 0 | -1.490579 | -1.047024 | 1.953758 |
| 21 | 6 | 0 | -4.002480 | -1.249257 | -0.341985 |
| 22 | 1 | 0 | -4.352371 | 0.212783 | -1.883031 |
| 23 | 1 | 0 | -3.370428 | -2.492475 | 1.294705 |
| 24 | 7 | 0 | -0.635544 | 2.597154 | 0.321652 |
| 25 | 7 | 0 | 0.518805 | 2.758178 | -0.946534 |
| 26 | 7 | 0 | 1.028195 | 3.466430 | -1.626367 |
| 27 | 6 | 0 | 5.114472 | -1.974531 | -1.092516 |
| 28 | 1 | 0 | 4.836304 | -2.563124 | -1.970662 |
| 29 | 1 | 0 | 5.846080 | -1.223438 | -1.412101 |
| 30 | 1 | 0 | 5.611677 | -2.636384 | -0.377601 |
| 31 | 6 | 0 | -5.174288 | -2.117957 | -0.724795 |
| 32 | 1 | 0 | -6.112321 | -1.699293 | -0.342303 |
| 33 | 1 | 0 | -5.270340 | -2.200145 | -1.812034 |
| 34 | 1 | 0 | -5.070320 | -3.126751 | -0.315025 |

| System: | **3** |
|---|---|
| Oxidant: | NIS |
| Nitrogen-donor: | $TMSN_3$ |
| Stationary point: | R |
| M06–2X/6–31+G(d) energy (in a.u.): | -8576.82618702 |
| Thermal correction to Gibbs Free Energy (in a.u.): | 0.318466 |
| Number of imaginary frequencies: | 0 |

**CARTESIAN COORDINATES**

| Center Number | Atomic Number | Atomic Type | Coordinates (Angstroms) X | Y | Z |
|---|---|---|---|---|---|
| 1 | 6 | 0 | 0.678915 | 0.728732 | 0.861505 |
| 2 | 6 | 0 | 1.404122 | -0.243322 | 0.803343 |
| 3 | 6 | 0 | -0.136731 | 1.902486 | 0.928452 |
| 4 | 6 | 0 | 0.157053 | 2.892192 | 1.880867 |
| 5 | 6 | 0 | -1.214823 | 2.075300 | 0.046606 |
| 6 | 6 | 0 | -0.616604 | 4.040680 | 1.950588 |
| 7 | 1 | 0 | 0.994725 | 2.750297 | 2.557312 |
| 8 | 6 | 0 | -1.995876 | 3.220165 | 0.122320 |
| 9 | 1 | 0 | -1.454579 | 1.311757 | -0.685680 |
| 10 | 6 | 0 | -1.695891 | 4.204314 | 1.071758 |
| 11 | 1 | 0 | -0.394241 | 4.812459 | 2.679866 |
| 12 | 1 | 0 | -2.838967 | 3.347554 | -0.548707 |
| 13 | 7 | 0 | 2.876638 | 2.917395 | -0.266791 |
| 14 | 7 | 0 | 3.316348 | 2.428658 | 0.756924 |
| 15 | 7 | 0 | 3.690205 | 2.033028 | 1.752156 |
| 16 | 6 | 0 | 2.306308 | -1.349652 | 0.710686 |
| 17 | 6 | 0 | 3.383908 | -1.447172 | 1.606656 |
| 18 | 6 | 0 | 2.146016 | -2.315396 | -0.296841 |
| 19 | 6 | 0 | 4.287642 | -2.494021 | 1.495328 |
| 20 | 1 | 0 | 3.507906 | -0.688730 | 2.372747 |
| 21 | 6 | 0 | 3.048308 | -3.363891 | -0.407187 |
| 22 | 1 | 0 | 1.316566 | -2.231075 | -0.992857 |
| 23 | 6 | 0 | 4.121846 | -3.453136 | 0.487826 |
| 24 | 1 | 0 | 5.125019 | -2.573534 | 2.180554 |
| 25 | 1 | 0 | 2.931183 | -4.113017 | -1.183178 |
| 26 | 53 | 0 | -1.844235 | -1.611940 | 0.415749 |
| 27 | 14 | 0 | 3.067566 | 2.178551 | -1.880173 |
| 28 | 6 | 0 | 4.280593 | 0.756278 | -1.725977 |
| 29 | 1 | 0 | 4.407921 | 0.256444 | -2.693069 |
| 30 | 1 | 0 | 5.266424 | 1.107543 | -1.401059 |
| 31 | 1 | 0 | 3.937474 | 0.002465 | -1.007998 |
| 32 | 6 | 0 | 3.722648 | 3.544596 | -2.975428 |
| 33 | 1 | 0 | 3.037747 | 4.399141 | -2.974761 |
| 34 | 1 | 0 | 4.698937 | 3.896478 | -2.626923 |
| 35 | 1 | 0 | 3.832795 | 3.200035 | -4.009722 |
| 36 | 6 | 0 | 1.382876 | 1.617499 | -2.471723 |
| 37 | 1 | 0 | 1.020016 | 0.753934 | -1.904249 |
| 38 | 1 | 0 | 0.650691 | 2.425405 | -2.363759 |
| 39 | 1 | 0 | 1.424746 | 1.339245 | -3.531427 |
| 40 | 6 | 0 | -4.297523 | -0.669540 | -1.079326 |
| 41 | 6 | 0 | -4.549996 | -2.858119 | -0.267772 |
| 42 | 6 | 0 | -5.695652 | -1.098863 | -1.498674 |
| 43 | 6 | 0 | -5.857501 | -2.531415 | -0.976458 |
| 44 | 1 | 0 | -6.411864 | -0.394132 | -1.068310 |
| 45 | 1 | 0 | -5.768266 | -1.017713 | -2.586115 |
| 46 | 1 | 0 | -6.673357 | -2.645557 | -0.258416 |
| 47 | 1 | 0 | -6.010588 | -3.269445 | -1.767876 |
| 48 | 7 | 0 | -3.724425 | -1.732182 | -0.381386 |
| 49 | 8 | 0 | -4.249122 | -3.878860 | 0.291874 |
| 50 | 8 | 0 | -3.756969 | 0.387504 | -1.296002 |
| 51 | 6 | 0 | 5.062224 | -4.536730 | 0.368651 |
| 52 | 6 | 0 | -2.505383 | 5.392709 | 1.150366 |
| 53 | 7 | 0 | 5.818775 | -5.407242 | 0.270462 |
| 54 | 7 | 0 | -3.156386 | 6.347804 | 1.214491 |

| System: | **3** |
|---|---|
| Oxidant: | NIS |
| Nitrogen-donor: | $TMSN_3$ |
| Stationary point: | TS1 |
| M06–2X/6–31+G(d) energy (in a.u.): | -8576.78101296 |
| Thermal correction to Gibbs Free Energy (in a.u.): | 0.325839 |
| Number of imaginary frequencies: | 1 (–356 $cm^{-1}$) |

**CARTESIAN COORDINATES**

| Center Number | Atomic Number | Atomic Type | Coordinates (Angstroms) X | Y | Z |
|---|---|---|---|---|---|
| 1 | 6 | 0 | -1.019914 | 0.622112 | -0.276621 |
| 2 | 6 | 0 | -0.570031 | -0.567517 | -0.091893 |
| 3 | 6 | 0 | -0.569839 | 2.013674 | -0.283182 |
| 4 | 6 | 0 | -1.036483 | 2.940722 | -1.224886 |
| 5 | 6 | 0 | 0.370318 | 2.419253 | 0.676181 |
| 6 | 6 | 0 | -0.576359 | 4.250455 | -1.211463 |
| 7 | 1 | 0 | -1.756804 | 2.631162 | -1.975572 |
| 8 | 6 | 0 | 0.842745 | 3.724987 | 0.688025 |
| 9 | 1 | 0 | 0.736280 | 1.705911 | 1.406688 |
| 10 | 6 | 0 | 0.363991 | 4.644875 | -0.251828 |
| 11 | 1 | 0 | -0.931887 | 4.968257 | -1.943537 |
| 12 | 1 | 0 | 1.579978 | 4.031884 | 1.422316 |
| 13 | 7 | 0 | -2.816271 | 0.746990 | -0.594023 |
| 14 | 7 | 0 | -3.226908 | -0.091744 | -1.413084 |
| 15 | 7 | 0 | -3.520544 | -0.871489 | -2.168091 |
| 16 | 6 | 0 | -1.244536 | -1.853137 | 0.012545 |
| 17 | 6 | 0 | -0.876024 | -2.934952 | -0.803198 |
| 18 | 6 | 0 | -2.275874 | -2.028493 | 0.951438 |
| 19 | 6 | 0 | -1.556536 | -4.141481 | -0.720878 |
| 20 | 1 | 0 | -0.054918 | -2.819349 | -1.504009 |
| 21 | 6 | 0 | -2.948915 | -3.238831 | 1.049391 |
| 22 | 1 | 0 | -2.530784 | -1.209299 | 1.618639 |
| 23 | 6 | 0 | -2.599459 | -4.296679 | 0.201817 |
| 24 | 1 | 0 | -1.279070 | -4.971887 | -1.361764 |
| 25 | 1 | 0 | -3.740870 | -3.374559 | 1.779541 |
| 26 | 53 | 0 | 1.947316 | -0.729896 | 0.001275 |
| 27 | 14 | 0 | -3.998306 | 1.605672 | 0.519001 |
| 28 | 6 | 0 | -5.368661 | 0.366554 | 0.805429 |
| 29 | 1 | 0 | -6.094075 | 0.771000 | 1.520318 |
| 30 | 1 | 0 | -5.914551 | 0.140877 | -0.117604 |
| 31 | 1 | 0 | -4.987664 | -0.576078 | 1.212560 |
| 32 | 6 | 0 | -4.598690 | 3.122332 | -0.387891 |
| 33 | 1 | 0 | -3.780391 | 3.829799 | -0.556391 |
| 34 | 1 | 0 | -5.037668 | 2.863220 | -1.357653 |
| 35 | 1 | 0 | -5.370135 | 3.634190 | 0.198904 |
| 36 | 6 | 0 | -3.000238 | 2.007997 | 2.039528 |
| 37 | 1 | 0 | -2.404536 | 1.153886 | 2.378985 |
| 38 | 1 | 0 | -2.317539 | 2.844239 | 1.858046 |
| 39 | 1 | 0 | -3.675534 | 2.294561 | 2.853910 |
| 40 | 6 | 0 | 4.895960 | 0.047764 | 0.691427 |
| 41 | 6 | 0 | 4.822099 | -1.947327 | -0.473905 |
| 42 | 6 | 0 | 6.357039 | -0.378633 | 0.555950 |
| 43 | 6 | 0 | 6.308152 | -1.700880 | -0.213282 |
| 44 | 1 | 0 | 6.901316 | 0.413638 | 0.035170 |
| 45 | 1 | 0 | 6.785755 | -0.467294 | 1.557643 |
| 46 | 1 | 0 | 6.827191 | -1.670296 | -1.174820 |
| 47 | 1 | 0 | 6.705848 | -2.550858 | 0.347420 |
| 48 | 7 | 0 | 4.100541 | -0.904577 | 0.081566 |
| 49 | 8 | 0 | 4.349387 | -2.891644 | -1.066077 |
| 50 | 8 | 0 | 4.494809 | 1.051911 | 1.240382 |
| 51 | 6 | 0 | -3.308174 | -5.545779 | 0.290315 |
| 52 | 6 | 0 | 0.831449 | 6.006901 | -0.233537 |
| 53 | 7 | 0 | -3.887477 | -6.545614 | 0.362311 |
| 54 | 7 | 0 | 1.193931 | 7.105927 | -0.220639 |

| System: | **3** |
|---|---|
| Oxidant: | NIS |
| Nitrogen-donor: | $TMSN_3$ |
| Stationary point: | IN1 |
| M06–2X/6–31+G(d) energy (in a.u.): | -8576.78787820 |
| Thermal correction to Gibbs Free Energy (in a.u.): | 0.328163 |
| Number of imaginary frequencies: | 0 |

**CARTESIAN COORDINATES**

---------------------------------------------------------------------

| Center Number | Atomic Number | Atomic Type | Coordinates (Angstroms) X | Y | Z |
|---|---|---|---|---|---|
| 1 | 6 | 0 | -1.028234 | 0.768451 | -0.236776 |
| 2 | 6 | 0 | -0.598286 | -0.485224 | -0.061239 |
| 3 | 6 | 0 | -0.271864 | 2.040049 | -0.273686 |
| 4 | 6 | 0 | -0.582552 | 3.024459 | -1.222652 |
| 5 | 6 | 0 | 0.765166 | 2.278303 | 0.639502 |
| 6 | 6 | 0 | 0.127314 | 4.217114 | -1.270834 |
| 7 | 1 | 0 | -1.374569 | 2.851233 | -1.947013 |
| 8 | 6 | 0 | 1.488509 | 3.462992 | 0.592136 |
| 9 | 1 | 0 | 1.016526 | 1.529912 | 1.382551 |
| 10 | 6 | 0 | 1.166447 | 4.437712 | -0.359812 |
| 11 | 1 | 0 | -0.107913 | 4.972010 | -2.014117 |
| 12 | 1 | 0 | 2.304556 | 3.628955 | 1.287441 |
| 13 | 7 | 0 | -2.500627 | 1.042825 | -0.466435 |
| 14 | 7 | 0 | -3.063547 | 0.337436 | -1.348788 |
| 15 | 7 | 0 | -3.567344 | -0.296752 | -2.119495 |
| 16 | 6 | 0 | -1.518675 | -1.630115 | -0.000144 |
| 17 | 6 | 0 | -1.384963 | -2.696646 | -0.905011 |
| 18 | 6 | 0 | -2.527044 | -1.698171 | 0.976548 |
| 19 | 6 | 0 | -2.266056 | -3.768968 | -0.871696 |
| 20 | 1 | 0 | -0.578336 | -2.677532 | -1.632045 |
| 21 | 6 | 0 | -3.401417 | -2.776455 | 1.028064 |
| 22 | 1 | 0 | -2.602437 | -0.907482 | 1.719396 |
| 23 | 6 | 0 | -3.283172 | -3.809645 | 0.090989 |
| 24 | 1 | 0 | -2.164460 | -4.584498 | -1.580242 |
| 25 | 1 | 0 | -4.170461 | -2.830518 | 1.792685 |
| 26 | 53 | 0 | 1.646457 | -1.007615 | 0.030338 |
| 27 | 14 | 0 | -3.581065 | 2.114015 | 0.638668 |
| 28 | 6 | 0 | -5.138497 | 1.095676 | 0.785771 |
| 29 | 1 | 0 | -5.808604 | 1.560249 | 1.518155 |
| 30 | 1 | 0 | -5.689411 | 1.041339 | -0.160001 |
| 31 | 1 | 0 | -4.932796 | 0.074436 | 1.123726 |
| 32 | 6 | 0 | -3.866688 | 3.713010 | -0.272398 |
| 33 | 1 | 0 | -2.958577 | 4.321710 | -0.309200 |
| 34 | 1 | 0 | -4.210115 | 3.537899 | -1.298292 |
| 35 | 1 | 0 | -4.645101 | 4.291901 | 0.238490 |
| 36 | 6 | 0 | -2.575970 | 2.270633 | 2.192771 |
| 37 | 1 | 0 | -2.170084 | 1.309269 | 2.523463 |
| 38 | 1 | 0 | -1.738644 | 2.961228 | 2.052938 |
| 39 | 1 | 0 | -3.211600 | 2.664067 | 2.994552 |
| 40 | 6 | 0 | 4.767720 | -0.548645 | 0.673527 |
| 41 | 6 | 0 | 4.534932 | -2.558225 | -0.386795 |
| 42 | 6 | 0 | 6.198910 | -1.086491 | 0.569362 |
| 43 | 6 | 0 | 6.042741 | -2.434438 | -0.134823 |
| 44 | 1 | 0 | 6.803212 | -0.364335 | 0.013308 |
| 45 | 1 | 0 | 6.619372 | -1.158573 | 1.576222 |
| 46 | 1 | 0 | 6.563277 | -2.495950 | -1.094372 |
| 47 | 1 | 0 | 6.365711 | -3.287540 | 0.468193 |
| 48 | 7 | 0 | 3.895673 | -1.448436 | 0.114220 |

| | | | | | |
|---|---|---|---|---|---|
| 49 | 8 | 0 | 3.996277 | -3.494939 | -0.945209 |
| 50 | 8 | 0 | 4.459014 | 0.518372 | 1.178013 |
| 51 | 6 | 0 | -4.204448 | -4.914374 | 0.127954 |
| 52 | 6 | 0 | 1.894893 | 5.679219 | -0.403556 |
| 53 | 7 | 0 | -4.957489 | -5.793156 | 0.157365 |
| 54 | 7 | 0 | 2.463082 | 6.686770 | -0.441263 |

---------------------------------------------------------------------

| System: | **3** |
|---|---|
| Oxidant: | NIS |
| Nitrogen-donor: | $TMSN_3$ |
| Stationary point: | TS2 |
| M06–2X/6–31+G(d) energy (in a.u.): | -8576.81454842 |
| Thermal correction to Gibbs Free Energy (in a.u.): | 0.338389 |
| Number of imaginary frequencies: | 1 (–34 $cm^{-1}$) |

**CARTESIAN COORDINATES**

---------------------------------------------------------------------

| Center Number | Atomic Number | Atomic Type | Coordinates (Angstroms) X | Y | Z |
|---|---|---|---|---|---|
| 1 | 6 | 0 | 0.129310 | -0.546710 | -0.569342 |
| 2 | 6 | 0 | -0.901194 | -1.273815 | -0.108760 |
| 3 | 6 | 0 | 1.573234 | -0.874756 | -0.563391 |
| 4 | 6 | 0 | 2.269310 | -0.906239 | -1.776279 |
| 5 | 6 | 0 | 2.246095 | -1.132264 | 0.635650 |
| 6 | 6 | 0 | 3.625147 | -1.203430 | -1.802713 |
| 7 | 1 | 0 | 1.747080 | -0.694532 | -2.706372 |
| 8 | 6 | 0 | 3.605802 | -1.417046 | 0.618189 |
| 9 | 1 | 0 | 1.696319 | -1.089807 | 1.570506 |
| 10 | 6 | 0 | 4.294814 | -1.454273 | -0.600030 |
| 11 | 1 | 0 | 4.168707 | -1.236130 | -2.741021 |
| 12 | 1 | 0 | 4.139785 | -1.609191 | 1.542925 |
| 13 | 7 | 0 | -0.134586 | 0.729141 | -1.229767 |
| 14 | 7 | 0 | -1.071063 | 0.682529 | -2.078817 |
| 15 | 7 | 0 | -1.902399 | 0.690656 | -2.824931 |
| 16 | 6 | 0 | -2.301737 | -0.791365 | -0.056761 |
| 17 | 6 | 0 | -3.347666 | -1.552535 | -0.594635 |
| 18 | 6 | 0 | -2.578511 | 0.462318 | 0.509499 |
| 19 | 6 | 0 | -4.648085 | -1.065251 | -0.591059 |
| 20 | 1 | 0 | -3.140207 | -2.530273 | -1.019829 |
| 21 | 6 | 0 | -3.879038 | 0.951360 | 0.520374 |
| 22 | 1 | 0 | -1.766997 | 1.028012 | 0.957415 |
| 23 | 6 | 0 | -4.915005 | 0.191033 | -0.034403 |
| 24 | 1 | 0 | -5.457656 | -1.650454 | -1.014339 |
| 25 | 1 | 0 | -4.097213 | 1.917585 | 0.963788 |
| 26 | 53 | 0 | -0.574049 | -3.223609 | 0.623310 |
| 27 | 14 | 0 | 0.731571 | 2.518258 | -1.158996 |
| 28 | 6 | 0 | -0.633793 | 3.727982 | -0.689483 |
| 29 | 1 | 0 | -0.434599 | 4.677100 | -1.200145 |
| 30 | 1 | 0 | -1.582592 | 3.346568 | -1.095285 |
| 31 | 1 | 0 | -0.751404 | 3.917210 | 0.375491 |
| 32 | 6 | 0 | 0.556543 | 2.731747 | -3.075171 |
| 33 | 1 | 0 | 0.947187 | 1.885543 | -3.657368 |
| 34 | 1 | 0 | -0.447105 | 2.972295 | -3.445930 |
| 35 | 1 | 0 | 1.187444 | 3.593296 | -3.335463 |
| 36 | 6 | 0 | 2.602392 | 2.311103 | -1.141328 |
| 37 | 1 | 0 | 2.987534 | 1.776945 | -0.270917 |
| 38 | 1 | 0 | 2.916248 | 1.799767 | -2.056265 |
| 39 | 1 | 0 | 3.034478 | 3.317154 | -1.147043 |
| 40 | 6 | 0 | 1.515043 | 3.331235 | 1.557917 |
| 41 | 6 | 0 | 0.614901 | 1.386155 | 2.207325 |
| 42 | 6 | 0 | 1.758249 | 3.321296 | 3.063688 |
| 43 | 6 | 0 | 1.166143 | 1.989491 | 3.501924 |
| 44 | 1 | 0 | 2.831619 | 3.419179 | 3.247989 |
| 45 | 1 | 0 | 1.270069 | 4.194125 | 3.505947 |
| 46 | 1 | 0 | 1.900430 | 1.294674 | 3.920908 |
| 47 | 1 | 0 | 0.351197 | 2.074504 | 4.225617 |
| 48 | 7 | 0 | 0.891386 | 2.178420 | 1.125447 |

| | | | | | |
|---|---|---|---|---|---|
| 49 | 8 | 0 | 0.003460 | 0.321774 | 2.187529 |
| 50 | 8 | 0 | 1.822002 | 4.258292 | 0.820243 |
| 51 | 6 | 0 | -6.262214 | 0.699813 | -0.029281 |
| 52 | 6 | 0 | 5.703820 | -1.753605 | -0.617080 |
| 53 | 7 | 0 | -7.344896 | 1.108740 | -0.028994 |
| 54 | 7 | 0 | 6.835446 | -1.995471 | -0.631703 |

---------------------------------------------------------------------

| System: | **3** |
| --- | --- |
| Oxidant: | NIS |
| Nitrogen-donor: | $TMSN_3$ |
| Stationary point: | IN2 |
| M06–2X/6–31+G(d) energy (in a.u.): | -7807.74742823 |
| Thermal correction to Gibbs Free Energy (in a.u.): | 0.156014 |
| Number of imaginary frequencies: | 0 |

**CARTESIAN COORDINATES**

| Center Number | Atomic Number | Atomic Type | Coordinates (Angstroms) X | Y | Z |
| --- | --- | --- | --- | --- | --- |
| 1 | 6 | 0 | -0.461751 | -0.906512 | -0.051165 |
| 2 | 6 | 0 | 0.401569 | 0.134470 | -0.045406 |
| 3 | 6 | 0 | -1.941345 | -0.819659 | 0.059153 |
| 4 | 6 | 0 | -2.742546 | -1.498440 | -0.864278 |
| 5 | 6 | 0 | -2.541866 | -0.114286 | 1.106666 |
| 6 | 6 | 0 | -4.127264 | -1.443215 | -0.770083 |
| 7 | 1 | 0 | -2.275282 | -2.070323 | -1.659899 |
| 8 | 6 | 0 | -3.924999 | -0.063617 | 1.218487 |
| 9 | 1 | 0 | -1.921990 | 0.388456 | 1.842483 |
| 10 | 6 | 0 | -4.719268 | -0.722517 | 0.273075 |
| 11 | 1 | 0 | -4.752469 | -1.956943 | -1.492652 |
| 12 | 1 | 0 | -4.394229 | 0.477215 | 2.033473 |
| 13 | 7 | 0 | -0.053848 | -2.266730 | -0.145663 |
| 14 | 7 | 0 | 0.991237 | -2.521497 | -0.769565 |
| 15 | 6 | 0 | 1.869715 | -0.010152 | 0.085557 |
| 16 | 6 | 0 | 2.755831 | 0.555119 | -0.840617 |
| 17 | 6 | 0 | 2.385427 | -0.733631 | 1.170614 |
| 18 | 6 | 0 | 4.125919 | 0.380175 | -0.704415 |
| 19 | 1 | 0 | 2.365616 | 1.130052 | -1.675309 |
| 20 | 6 | 0 | 3.755761 | -0.908400 | 1.318227 |
| 21 | 1 | 0 | 1.702390 | -1.160481 | 1.900068 |
| 22 | 6 | 0 | 4.629136 | -0.353379 | 0.377112 |
| 23 | 1 | 0 | 4.810886 | 0.808126 | -1.428850 |
| 24 | 1 | 0 | 4.153077 | -1.467253 | 2.159098 |
| 25 | 53 | 0 | -0.281818 | 2.130348 | -0.250365 |
| 26 | 7 | 0 | 1.904698 | -2.891335 | -1.313516 |
| 27 | 6 | 0 | 6.050238 | -0.531838 | 0.524057 |
| 28 | 6 | 0 | -6.154549 | -0.667330 | 0.380807 |
| 29 | 7 | 0 | -7.307825 | -0.622273 | 0.466993 |
| 30 | 7 | 0 | 7.192639 | -0.675026 | 0.642346 |

| System: | **3** |
|---|---|
| Oxidant: | NIS |
| Nitrogen-donor: | $TMSN_3$ |
| Stationary point: | TS3 |
| M06–2X/6–31+G(d) energy (in a.u.): | -7807.70016448 |
| Thermal correction to Gibbs Free Energy (in a.u.): | 0.151959 |
| Number of imaginary frequencies: | 1 (–546 $cm^{-1}$) |

**CARTESIAN COORDINATES**

| Center Number | Atomic Number | Atomic Type | Coordinates (Angstroms) X | Y | Z |
|---|---|---|---|---|---|
| 1 | 6 | 0 | -0.057678 | -1.306048 | 0.284843 |
| 2 | 6 | 0 | -1.074623 | -0.393672 | 0.028429 |
| 3 | 6 | 0 | 1.382846 | -0.988703 | 0.079655 |
| 4 | 6 | 0 | 2.341389 | -1.469748 | 0.978332 |
| 5 | 6 | 0 | 1.789191 | -0.263092 | -1.045900 |
| 6 | 6 | 0 | 3.691220 | -1.215735 | 0.767363 |
| 7 | 1 | 0 | 2.018227 | -2.025224 | 1.854626 |
| 8 | 6 | 0 | 3.135407 | -0.006866 | -1.266859 |
| 9 | 1 | 0 | 1.043858 | 0.096162 | -1.750308 |
| 10 | 6 | 0 | 4.088464 | -0.482729 | -0.357039 |
| 11 | 1 | 0 | 4.438769 | -1.576900 | 1.466152 |
| 12 | 1 | 0 | 3.456449 | 0.554080 | -2.138429 |
| 13 | 6 | 0 | -0.870661 | 1.060956 | 0.141914 |
| 14 | 6 | 0 | -1.480128 | 1.952136 | -0.756645 |
| 15 | 6 | 0 | -0.028820 | 1.576995 | 1.140587 |
| 16 | 6 | 0 | -1.220339 | 3.312449 | -0.689151 |
| 17 | 1 | 0 | -2.151177 | 1.570809 | -1.520202 |
| 18 | 6 | 0 | 0.225674 | 2.939200 | 1.222453 |
| 19 | 1 | 0 | 0.416666 | 0.903008 | 1.865978 |
| 20 | 6 | 0 | -0.364328 | 3.811112 | 0.301467 |
| 21 | 1 | 0 | -1.678472 | 3.995778 | -1.396740 |
| 22 | 1 | 0 | 0.873781 | 3.332533 | 1.998821 |
| 23 | 7 | 0 | -0.480281 | -2.402537 | 0.907080 |
| 24 | 7 | 0 | 0.447829 | -3.770406 | 0.353234 |
| 25 | 53 | 0 | -3.058690 | -1.049258 | -0.277461 |
| 26 | 7 | 0 | 0.747264 | -4.810303 | 0.573929 |
| 27 | 6 | 0 | -0.102293 | 5.224156 | 0.378569 |
| 28 | 6 | 0 | 5.486474 | -0.219782 | -0.581990 |
| 29 | 7 | 0 | 6.610051 | -0.009217 | -0.762434 |
| 30 | 7 | 0 | 0.109669 | 6.360555 | 0.438098 |

| System: | **3** |
| --- | --- |
| Oxidant: | NIS |
| Nitrogen-donor: | $TMSN_3$ |
| Stationary point: | IN3 |
| M06–2X/6–31+G(d) energy (in a.u.): | -7698.31469140 |
| Thermal correction to Gibbs Free Energy (in a.u.): | 0.147838 |
| Number of imaginary frequencies: | 0 |

**CARTESIAN COORDINATES**

| Center Number | Atomic Number | Atomic Type | Coordinates (Angstroms) X | Y | Z |
| --- | --- | --- | --- | --- | --- |
| 1 | 6 | 0 | 0.778589 | 0.218272 | 0.931384 |
| 2 | 6 | 0 | -0.546705 | 0.658472 | 0.594771 |
| 3 | 7 | 0 | 0.110821 | 0.617678 | 1.928812 |
| 4 | 6 | 0 | 2.074716 | -0.290890 | 0.534128 |
| 5 | 6 | 0 | 2.309633 | -0.581784 | -0.812852 |
| 6 | 6 | 0 | 3.074112 | -0.479255 | 1.496934 |
| 7 | 6 | 0 | 3.550535 | -1.067359 | -1.205214 |
| 8 | 1 | 0 | 1.519897 | -0.421697 | -1.541342 |
| 9 | 6 | 0 | 4.313867 | -0.964884 | 1.109303 |
| 10 | 1 | 0 | 2.869270 | -0.241999 | 2.536529 |
| 11 | 6 | 0 | 4.548352 | -1.258038 | -0.242029 |
| 12 | 1 | 0 | 3.754363 | -1.298501 | -2.245095 |
| 13 | 1 | 0 | 5.103233 | -1.119011 | 1.837132 |
| 14 | 6 | 0 | -1.684124 | -0.277662 | 0.323246 |
| 15 | 6 | 0 | -2.246726 | -0.403063 | -0.950568 |
| 16 | 6 | 0 | -2.167130 | -1.066548 | 1.371516 |
| 17 | 6 | 0 | -3.270811 | -1.312033 | -1.180032 |
| 18 | 1 | 0 | -1.889394 | 0.221115 | -1.764156 |
| 19 | 6 | 0 | -3.200316 | -1.970724 | 1.153315 |
| 20 | 1 | 0 | -1.732281 | -0.964934 | 2.361246 |
| 21 | 6 | 0 | -3.751717 | -2.097627 | -0.125439 |
| 22 | 1 | 0 | -3.705602 | -1.414330 | -2.168797 |
| 23 | 1 | 0 | -3.583285 | -2.576927 | 1.967707 |
| 24 | 53 | 0 | -0.760427 | 2.629713 | -0.298915 |
| 25 | 6 | 0 | 5.836559 | -1.762224 | -0.644937 |
| 26 | 6 | 0 | -4.817834 | -3.037058 | -0.358836 |
| 27 | 7 | 0 | 6.870988 | -2.167430 | -0.968172 |
| 28 | 7 | 0 | -5.673179 | -3.793691 | -0.547622 |

| System: | **3** |
|---|---|
| Oxidant: | NIS |
| Nitrogen-donor: | $TMSN_3$ |
| Stationary point: | TS4 |
| M06–2X/6–31+G(d) energy (in a.u.): | -8271.60554183 |
| Thermal correction to Gibbs Free Energy (in a.u.): | 0.260702 |
| Number of imaginary frequencies: | 1 (–224 $cm^{-1}$) |

**CARTESIAN COORDINATES**

| Center Number | Atomic Number | Atomic Type | Coordinates (Angstroms) X | Y | Z |
|---|---|---|---|---|---|
| 1 | 6 | 0 | -0.597706 | -0.519159 | -1.312158 |
| 2 | 6 | 0 | 0.774479 | -0.670275 | -1.376143 |
| 3 | 7 | 0 | 0.112287 | -0.303253 | -2.453052 |
| 4 | 6 | 0 | -1.896855 | -1.048970 | -0.968608 |
| 5 | 6 | 0 | -2.124309 | -1.594264 | 0.299997 |
| 6 | 6 | 0 | -2.913678 | -0.983970 | -1.934093 |
| 7 | 6 | 0 | -3.382250 | -2.112338 | 0.593184 |
| 8 | 1 | 0 | -1.338991 | -1.561463 | 1.052522 |
| 9 | 6 | 0 | -4.163503 | -1.500110 | -1.638351 |
| 10 | 1 | 0 | -2.711073 | -0.534325 | -2.901861 |
| 11 | 6 | 0 | -4.390969 | -2.067523 | -0.373445 |
| 12 | 1 | 0 | -3.586192 | -2.533631 | 1.571474 |
| 13 | 1 | 0 | -4.965865 | -1.470043 | -2.367364 |
| 14 | 6 | 0 | 2.119150 | -1.017969 | -1.016951 |
| 15 | 6 | 0 | 2.420557 | -1.550355 | 0.243089 |
| 16 | 6 | 0 | 3.114503 | -0.829185 | -1.991916 |
| 17 | 6 | 0 | 3.737059 | -1.891899 | 0.530364 |
| 18 | 1 | 0 | 1.642783 | -1.646653 | 0.993752 |
| 19 | 6 | 0 | 4.423628 | -1.168185 | -1.702219 |
| 20 | 1 | 0 | 2.849470 | -0.418412 | -2.962072 |
| 21 | 6 | 0 | 4.728399 | -1.698531 | -0.437082 |
| 22 | 1 | 0 | 3.998374 | -2.292582 | 1.503499 |
| 23 | 1 | 0 | 5.212240 | -1.029719 | -2.433454 |
| 24 | 53 | 0 | 0.301363 | 0.423192 | 2.339230 |
| 25 | 7 | 0 | -1.112251 | 1.571324 | -0.774415 |
| 26 | 7 | 0 | -2.080921 | 1.574394 | -0.010802 |
| 27 | 7 | 0 | -2.984500 | 1.474672 | 0.653987 |
| 28 | 14 | 0 | -0.008162 | 3.023384 | -0.801470 |
| 29 | 6 | 0 | -0.443444 | 3.909494 | -2.394819 |
| 30 | 1 | 0 | -1.481186 | 4.259412 | -2.388710 |
| 31 | 1 | 0 | 0.204372 | 4.780937 | -2.544866 |
| 32 | 1 | 0 | -0.316016 | 3.242938 | -3.255255 |
| 33 | 6 | 0 | 1.748147 | 2.395255 | -0.873034 |
| 34 | 1 | 0 | 2.425849 | 3.253771 | -0.789677 |
| 35 | 1 | 0 | 1.957352 | 1.717934 | -0.037757 |
| 36 | 1 | 0 | 1.970560 | 1.901191 | -1.825661 |
| 37 | 6 | 0 | -0.391804 | 4.044824 | 0.709552 |
| 38 | 1 | 0 | -0.209737 | 3.463012 | 1.619634 |
| 39 | 1 | 0 | 0.250735 | 4.932774 | 0.728732 |
| 40 | 1 | 0 | -1.432016 | 4.390033 | 0.713561 |
| 41 | 6 | 0 | 6.093259 | -2.052487 | -0.138593 |
| 42 | 6 | 0 | -5.693156 | -2.605904 | -0.070956 |
| 43 | 7 | 0 | -6.739082 | -3.041910 | 0.161896 |
| 44 | 7 | 0 | 7.190512 | -2.338431 | 0.090673 |

| System: | **3** |
| --- | --- |
| Oxidant: | NIS |
| Nitrogen-donor: | $TMSN_3$ |
| Stationary point: | IN4 |
| M06–2X/6–31+G(d) energy (in a.u.): | -1351.47778194 |
| Thermal correction to Gibbs Free Energy (in a.u.): | 0.264771 |
| Number of imaginary frequencies: | 0 |

**CARTESIAN COORDINATES**

---------------------------------------------------------------------

| Center Number | Atomic Number | Atomic Type | Coordinates (Angstroms) X | Y | Z |
| --- | --- | --- | --- | --- | --- |
| 1 | 6 | 0 | 0.424183 | 0.350359 | 0.265669 |
| 2 | 6 | 0 | -0.959318 | 0.306461 | 0.689992 |
| 3 | 7 | 0 | -0.158354 | 0.719439 | 1.577751 |
| 4 | 6 | 0 | 1.332123 | -0.831428 | 0.145214 |
| 5 | 6 | 0 | 1.208768 | -1.863018 | 1.082496 |
| 6 | 6 | 0 | 2.283315 | -0.932377 | -0.874214 |
| 7 | 6 | 0 | 2.028888 | -2.981278 | 1.003692 |
| 8 | 1 | 0 | 0.479225 | -1.790423 | 1.883611 |
| 9 | 6 | 0 | 3.103923 | -2.051204 | -0.961104 |
| 10 | 1 | 0 | 2.405079 | -0.139242 | -1.607356 |
| 11 | 6 | 0 | 2.977638 | -3.077950 | -0.020324 |
| 12 | 1 | 0 | 1.940196 | -3.779858 | 1.732772 |
| 13 | 1 | 0 | 3.842429 | -2.132835 | -1.751763 |
| 14 | 6 | 0 | -2.336964 | -0.036532 | 0.420506 |
| 15 | 6 | 0 | -2.669795 | -0.657198 | -0.789775 |
| 16 | 6 | 0 | -3.316492 | 0.247005 | 1.383021 |
| 17 | 6 | 0 | -3.990794 | -0.997380 | -1.044822 |
| 18 | 1 | 0 | -1.894266 | -0.887685 | -1.515904 |
| 19 | 6 | 0 | -4.637039 | -0.088629 | 1.127651 |
| 20 | 1 | 0 | -3.036963 | 0.723957 | 2.317818 |
| 21 | 6 | 0 | -4.970335 | -0.708229 | -0.085985 |
| 22 | 1 | 0 | -4.271698 | -1.484306 | -1.972415 |
| 23 | 1 | 0 | -5.413867 | 0.121133 | 1.855126 |
| 24 | 7 | 0 | 0.891157 | 1.548900 | -0.493515 |
| 25 | 7 | 0 | 0.395836 | 1.697509 | -1.646687 |
| 26 | 7 | 0 | -0.022645 | 1.844519 | -2.671362 |
| 27 | 14 | 0 | 2.045883 | 2.875322 | 0.273831 |
| 28 | 6 | 0 | 3.159894 | 1.825026 | 1.318769 |
| 29 | 1 | 0 | 3.769817 | 1.141961 | 0.720187 |
| 30 | 1 | 0 | 3.836911 | 2.477304 | 1.883028 |
| 31 | 1 | 0 | 2.586601 | 1.240153 | 2.046037 |
| 32 | 6 | 0 | 0.899059 | 3.996952 | 1.206996 |
| 33 | 1 | 0 | 1.467764 | 4.826472 | 1.643526 |
| 34 | 1 | 0 | 0.132575 | 4.431675 | 0.556830 |
| 35 | 1 | 0 | 0.405862 | 3.460935 | 2.023491 |
| 36 | 6 | 0 | 2.801407 | 3.617543 | -1.255811 |
| 37 | 1 | 0 | 2.072104 | 4.151766 | -1.874870 |
| 38 | 1 | 0 | 3.558024 | 4.353780 | -0.960146 |
| 39 | 1 | 0 | 3.309397 | 2.866899 | -1.871264 |
| 40 | 6 | 0 | -6.342998 | -1.054582 | -0.350938 |
| 41 | 6 | 0 | 3.828612 | -4.236388 | -0.107697 |
| 42 | 7 | 0 | 4.514116 | -5.165048 | -0.180814 |
| 43 | 7 | 0 | -7.445253 | -1.330906 | -0.565709 |

---------------------------------------------------------------------

| System: | **3** |
|---|---|
| Oxidant: | NIS |
| Nitrogen-donor: | $TMSN_3$ |
| Stationary point: | TS5 |
| M06–2X/6–31+G(d) energy (in a.u.): | -1711.62988089 |
| Thermal correction to Gibbs Free Energy (in a.u.): | 0.341799 |
| Number of imaginary frequencies: | 1 (–62 $cm^{-1}$) |

**CARTESIAN COORDINATES**

| Center Number | Atomic Number | Atomic Type | Coordinates (Angstroms) X | Y | Z |
|---|---|---|---|---|---|
| 1 | 6 | 0 | -0.853657 | -0.403662 | 0.393087 |
| 2 | 6 | 0 | -0.370601 | 0.906286 | 0.788439 |
| 3 | 7 | 0 | -0.470823 | 0.085235 | 1.742022 |
| 4 | 6 | 0 | -2.292876 | -0.696412 | 0.106939 |
| 5 | 6 | 0 | -2.689784 | -1.821038 | -0.622564 |
| 6 | 6 | 0 | -3.268041 | 0.187245 | 0.586434 |
| 7 | 6 | 0 | -4.036157 | -2.062262 | -0.871729 |
| 8 | 1 | 0 | -1.952042 | -2.529639 | -0.986687 |
| 9 | 6 | 0 | -4.614022 | -0.047436 | 0.344575 |
| 10 | 1 | 0 | -2.974815 | 1.063013 | 1.158249 |
| 11 | 6 | 0 | -5.001810 | -1.174895 | -0.388837 |
| 12 | 1 | 0 | -4.342323 | -2.938040 | -1.434382 |
| 13 | 1 | 0 | -5.368192 | 0.637062 | 0.718686 |
| 14 | 6 | 0 | -0.173530 | 2.320073 | 0.526082 |
| 15 | 6 | 0 | -0.149124 | 2.800726 | -0.784714 |
| 16 | 6 | 0 | -0.044150 | 3.187685 | 1.615435 |
| 17 | 6 | 0 | -0.001259 | 4.161189 | -1.011487 |
| 18 | 1 | 0 | -0.213086 | 2.102624 | -1.612793 |
| 19 | 6 | 0 | 0.108507 | 4.549048 | 1.393098 |
| 20 | 1 | 0 | -0.064851 | 2.789526 | 2.625840 |
| 21 | 6 | 0 | 0.126829 | 5.031849 | 0.078399 |
| 22 | 1 | 0 | 0.026631 | 4.555868 | -2.021385 |
| 23 | 1 | 0 | 0.211899 | 5.240350 | 2.222672 |
| 24 | 7 | 0 | 0.095957 | -1.382291 | -0.163003 |
| 25 | 7 | 0 | 0.345856 | -1.234640 | -1.391244 |
| 26 | 7 | 0 | 0.540229 | -1.177494 | -2.486637 |
| 27 | 14 | 0 | 1.116465 | -2.638823 | 0.880839 |
| 28 | 6 | 0 | 1.570799 | -4.003997 | -0.309556 |
| 29 | 1 | 0 | 1.951484 | -3.629226 | -1.262157 |
| 30 | 1 | 0 | 2.390521 | -4.585937 | 0.124884 |
| 31 | 1 | 0 | 0.712176 | -4.664523 | -0.474109 |
| 32 | 6 | 0 | -0.404651 | -3.256800 | 1.826967 |
| 33 | 1 | 0 | -0.080836 | -4.069559 | 2.490899 |
| 34 | 1 | 0 | -0.848093 | -2.480996 | 2.461274 |
| 35 | 1 | 0 | -1.186838 | -3.661669 | 1.175399 |
| 36 | 6 | 0 | 2.223989 | -1.908793 | 2.198035 |
| 37 | 1 | 0 | 2.204224 | -0.816281 | 2.146327 |
| 38 | 1 | 0 | 1.872540 | -2.216065 | 3.188757 |
| 39 | 1 | 0 | 3.254211 | -2.243297 | 2.048040 |
| 40 | 7 | 0 | 2.866104 | -1.308915 | -0.459876 |
| 41 | 6 | 0 | 4.145783 | -1.818164 | -0.445136 |
| 42 | 6 | 0 | 5.178958 | -0.737187 | -0.787323 |
| 43 | 6 | 0 | 4.338843 | 0.530825 | -0.900621 |
| 44 | 6 | 0 | 2.912145 | 0.025302 | -0.678285 |
| 45 | 8 | 0 | 1.922912 | 0.774996 | -0.703622 |
| 46 | 1 | 0 | 4.569202 | 1.283275 | -0.140238 |
| 47 | 1 | 0 | 5.936780 | -0.706168 | -0.000107 |
| 48 | 8 | 0 | 4.432331 | -2.973542 | -0.171069 |

| | | | | | |
|---|---|---|---|---|---|
| 49 | 1 | 0 | 4.395219 | 1.025280 | -1.874563 |
| 50 | 1 | 0 | 5.684515 | -1.014685 | -1.716865 |
| 51 | 6 | 0 | -6.397235 | -1.420354 | -0.646666 |
| 52 | 6 | 0 | 0.280071 | 6.445480 | -0.155916 |
| 53 | 7 | 0 | -7.518584 | -1.616825 | -0.854985 |
| 54 | 7 | 0 | 0.400557 | 7.581035 | -0.343406 |

---------------------------------------------------------------------

| | |
|---|---|
| System: | **3** |
| Oxidant: | NIS |
| Nitrogen-donor: | $TMSN_3$ |
| Stationary point: | IN5 |
| M06–2X/6–31+G(d) energy (in a.u.): | -942.540097599 |
| Thermal correction to Gibbs Free Energy (in a.u.): | 0.160989 |
| Number of imaginary frequencies: | 0 |

**CARTESIAN COORDINATES**

| Center Number | Atomic Number | Atomic Type | Coordinates (Angstroms) X | Y | Z |
|---|---|---|---|---|---|
| 1 | 6 | 0 | 0.578267 | 0.869861 | 0.615826 |
| 2 | 6 | 0 | -0.722164 | 1.369411 | 0.223443 |
| 3 | 7 | 0 | -0.025759 | 1.472857 | 1.550254 |
| 4 | 6 | 0 | 1.820276 | 0.168310 | 0.336068 |
| 5 | 6 | 0 | 2.020408 | -0.407844 | -0.921476 |
| 6 | 6 | 0 | 2.798697 | 0.075291 | 1.333610 |
| 7 | 6 | 0 | 3.204357 | -1.083892 | -1.188285 |
| 8 | 1 | 0 | 1.246580 | -0.324412 | -1.679383 |
| 9 | 6 | 0 | 3.982186 | -0.598173 | 1.071259 |
| 10 | 1 | 0 | 2.621148 | 0.533463 | 2.302268 |
| 11 | 6 | 0 | 4.181727 | -1.176562 | -0.190602 |
| 12 | 1 | 0 | 3.378941 | -1.538790 | -2.157447 |
| 13 | 1 | 0 | 4.754346 | -0.681946 | 1.828731 |
| 14 | 6 | 0 | -1.921729 | 0.485402 | 0.119560 |
| 15 | 6 | 0 | -2.959599 | 0.776544 | -0.770312 |
| 16 | 6 | 0 | -2.006991 | -0.653827 | 0.928448 |
| 17 | 6 | 0 | -4.064835 | -0.062137 | -0.857279 |
| 18 | 1 | 0 | -2.900257 | 1.663385 | -1.390720 |
| 19 | 6 | 0 | -3.109226 | -1.493629 | 0.849772 |
| 20 | 1 | 0 | -1.212948 | -0.881547 | 1.633294 |
| 21 | 6 | 0 | -4.142202 | -1.199676 | -0.047776 |
| 22 | 1 | 0 | -4.871467 | 0.162200 | -1.547660 |
| 23 | 1 | 0 | -3.177174 | -2.374680 | 1.479489 |
| 24 | 7 | 0 | -0.878155 | 2.577420 | -0.570519 |
| 25 | 7 | 0 | 0.053318 | 3.382444 | -0.476417 |
| 26 | 7 | 0 | 0.861718 | 4.168970 | -0.448201 |
| 27 | 6 | 0 | 5.411176 | -1.877063 | -0.463456 |
| 28 | 6 | 0 | -5.284995 | -2.071388 | -0.138289 |
| 29 | 7 | 0 | 6.398319 | -2.439934 | -0.681650 |
| 30 | 7 | 0 | -6.202482 | -2.773391 | -0.212271 |

| System: | **3** |
| --- | --- |
| Oxidant: | NIS |
| Nitrogen-donor: | $TMSN_3$ |
| Stationary point: | TS6 |
| M06–2X/6–31+G(d) energy (in a.u.): | -942.479838425 |
| Thermal correction to Gibbs Free Energy (in a.u.): | 0.155591 |
| Number of imaginary frequencies: | 1 (–602 $cm^{-1}$) |

**CARTESIAN COORDINATES**

| Center Number | Atomic Number | Atomic Type | Coordinates (Angstroms) X | Y | Z |
| --- | --- | --- | --- | --- | --- |
| 1 | 6 | 0 | 0.550405 | 0.956963 | 1.182248 |
| 2 | 6 | 0 | -0.704544 | 1.559961 | 0.630270 |
| 3 | 7 | 0 | -0.066221 | 1.454615 | 2.125817 |
| 4 | 6 | 0 | 1.706402 | 0.213442 | 0.710058 |
| 5 | 6 | 0 | 1.692433 | -0.305992 | -0.587572 |
| 6 | 6 | 0 | 2.810935 | 0.030598 | 1.549883 |
| 7 | 6 | 0 | 2.788692 | -1.021273 | -1.051968 |
| 8 | 1 | 0 | 0.819724 | -0.155279 | -1.217735 |
| 9 | 6 | 0 | 3.908765 | -0.679589 | 1.086201 |
| 10 | 1 | 0 | 2.800955 | 0.446688 | 2.552614 |
| 11 | 6 | 0 | 3.894825 | -1.203950 | -0.213776 |
| 12 | 1 | 0 | 2.796598 | -1.437350 | -2.053698 |
| 13 | 1 | 0 | 4.776833 | -0.833209 | 1.718460 |
| 14 | 6 | 0 | -1.851528 | 0.625002 | 0.382196 |
| 15 | 6 | 0 | -2.701735 | 0.874952 | -0.701327 |
| 16 | 6 | 0 | -2.087013 | -0.492058 | 1.193324 |
| 17 | 6 | 0 | -3.762739 | 0.022720 | -0.977944 |
| 18 | 1 | 0 | -2.526403 | 1.754015 | -1.312883 |
| 19 | 6 | 0 | -3.150402 | -1.345872 | 0.926528 |
| 20 | 1 | 0 | -1.448127 | -0.689920 | 2.048326 |
| 21 | 6 | 0 | -3.989432 | -1.093390 | -0.164095 |
| 22 | 1 | 0 | -4.422055 | 0.217971 | -1.817703 |
| 23 | 1 | 0 | -3.335363 | -2.208352 | 1.558806 |
| 24 | 7 | 0 | -0.657713 | 2.790978 | 0.075765 |
| 25 | 7 | 0 | 0.541536 | 2.852134 | -1.181532 |
| 26 | 7 | 0 | 1.083006 | 3.501967 | -1.892341 |
| 27 | 6 | 0 | -5.086562 | -1.981372 | -0.448278 |
| 28 | 6 | 0 | 5.036307 | -1.941081 | -0.693676 |
| 29 | 7 | 0 | -5.966869 | -2.697138 | -0.678812 |
| 30 | 7 | 0 | 5.953259 | -2.532403 | -1.078699 |

| System: | **4** |
|---|---|
| Oxidant: | NIS |
| Nitrogen-donor: | $TMSN_3$ |
| Stationary point: | R |
| M06–2X/6–31+G(d) energy (in a.u.): | -8275.90538929 |
| Thermal correction to Gibbs Free Energy (in a.u.): | 0.283132 |
| Number of imaginary frequencies: | 0 |

**CARTESIAN COORDINATES**

| Center Number | Atomic Number | Atomic Type | Coordinates (Angstroms) X | Y | Z |
|---|---|---|---|---|---|
| 1 | 6 | 0 | -0.888977 | -0.889248 | -0.763892 |
| 2 | 6 | 0 | -0.525656 | -2.036487 | -0.911153 |
| 3 | 6 | 0 | -1.313464 | 0.471562 | -0.600414 |
| 4 | 6 | 0 | -2.200596 | 1.043529 | -1.518091 |
| 5 | 6 | 0 | -0.845958 | 1.251937 | 0.472974 |
| 6 | 6 | 0 | -2.616713 | 2.365294 | -1.382913 |
| 7 | 1 | 0 | -2.579501 | 0.439013 | -2.335655 |
| 8 | 6 | 0 | -1.258191 | 2.565385 | 0.616071 |
| 9 | 1 | 0 | -0.149234 | 0.824557 | 1.188423 |
| 10 | 6 | 0 | -2.144462 | 3.130735 | -0.311684 |
| 11 | 1 | 0 | -3.308622 | 2.778208 | -2.107339 |
| 12 | 1 | 0 | -0.902393 | 3.180131 | 1.436207 |
| 13 | 7 | 0 | -4.017574 | -1.605728 | -0.732007 |
| 14 | 7 | 0 | -3.602035 | -2.695612 | -1.093595 |
| 15 | 7 | 0 | -3.206362 | -3.677594 | -1.496866 |
| 16 | 53 | 0 | 2.345626 | -0.750974 | -0.318197 |
| 17 | 14 | 0 | -4.352643 | -1.270306 | 0.989902 |
| 18 | 6 | 0 | -5.890345 | -2.239364 | 1.460159 |
| 19 | 1 | 0 | -6.149533 | -2.072657 | 2.512103 |
| 20 | 1 | 0 | -6.747051 | -1.938999 | 0.847988 |
| 21 | 1 | 0 | -5.738515 | -3.316033 | 1.322235 |
| 22 | 6 | 0 | -4.643303 | 0.569617 | 1.087756 |
| 23 | 1 | 0 | -3.708456 | 1.127286 | 0.969438 |
| 24 | 1 | 0 | -5.334750 | 0.893367 | 0.302218 |
| 25 | 1 | 0 | -5.079171 | 0.836543 | 2.057169 |
| 26 | 6 | 0 | -2.891768 | -1.834486 | 2.020517 |
| 27 | 1 | 0 | -2.624442 | -2.874720 | 1.799475 |
| 28 | 1 | 0 | -2.008503 | -1.216232 | 1.831731 |
| 29 | 1 | 0 | -3.131770 | -1.773116 | 3.088448 |
| 30 | 6 | 0 | 4.435935 | 0.875739 | 1.182336 |
| 31 | 6 | 0 | 5.398743 | -0.570208 | -0.386863 |
| 32 | 6 | 0 | 5.937918 | 1.106651 | 1.288613 |
| 33 | 6 | 0 | 6.568160 | 0.160023 | 0.261979 |
| 34 | 1 | 0 | 6.136484 | 2.162900 | 1.089883 |
| 35 | 1 | 0 | 6.243684 | 0.902871 | 2.317936 |
| 36 | 1 | 0 | 7.127512 | 0.675236 | -0.523010 |
| 37 | 1 | 0 | 7.233817 | -0.585321 | 0.704527 |
| 38 | 7 | 0 | 4.225873 | -0.094071 | 0.202079 |
| 39 | 8 | 0 | 5.451218 | -1.408518 | -1.250243 |
| 40 | 8 | 0 | 3.568195 | 1.419531 | 1.818797 |
| 41 | 1 | 0 | -0.255304 | -3.062545 | -1.044547 |
| 42 | 8 | 0 | -2.489756 | 4.420915 | -0.085763 |
| 43 | 6 | 0 | -3.386919 | 5.034886 | -0.988910 |
| 44 | 1 | 0 | -3.525605 | 6.053142 | -0.627100 |
| 45 | 1 | 0 | -2.970495 | 5.059619 | -2.002805 |
| 46 | 1 | 0 | -4.352295 | 4.514874 | -1.000005 |

| System: | **4** |
| --- | --- |
| Oxidant: | NIS |
| Nitrogen-donor: | $TMSN_3$ |
| Stationary point: | TS1 |
| M06–2X/6–31+G(d) energy (in a.u.): | -8275.86169668 |
| Thermal correction to Gibbs Free Energy (in a.u.): | 0.289234 |
| Number of imaginary frequencies: | 1 (–344 $cm^{-1}$) |

**CARTESIAN COORDINATES**

| Center Number | Atomic Number | Atomic Type | Coordinates (Angstroms) X | Y | Z |
| --- | --- | --- | --- | --- | --- |
| 1 | 6 | 0 | -1.262228 | -0.916555 | -0.487608 |
| 2 | 6 | 0 | -0.313851 | -1.787715 | -0.460428 |
| 3 | 6 | 0 | -1.468269 | 0.525545 | -0.401287 |
| 4 | 6 | 0 | -2.210986 | 1.211061 | -1.364770 |
| 5 | 6 | 0 | -0.892280 | 1.249749 | 0.657413 |
| 6 | 6 | 0 | -2.393043 | 2.588772 | -1.287321 |
| 7 | 1 | 0 | -2.656256 | 0.658930 | -2.188025 |
| 8 | 6 | 0 | -1.066094 | 2.619949 | 0.744857 |
| 9 | 1 | 0 | -0.280251 | 0.735177 | 1.392315 |
| 10 | 6 | 0 | -1.820119 | 3.297364 | -0.223989 |
| 11 | 1 | 0 | -2.969538 | 3.093796 | -2.053309 |
| 12 | 1 | 0 | -0.608596 | 3.193043 | 1.544257 |
| 13 | 7 | 0 | -2.955934 | -1.561712 | -0.573405 |
| 14 | 7 | 0 | -2.984296 | -2.670639 | -1.129406 |
| 15 | 7 | 0 | -2.883427 | -3.651217 | -1.671595 |
| 16 | 53 | 0 | 1.906776 | -0.954800 | -0.209574 |
| 17 | 14 | 0 | -4.035137 | -1.257794 | 0.897177 |
| 18 | 6 | 0 | -5.373833 | -2.556460 | 0.746993 |
| 19 | 1 | 0 | -6.086416 | -2.443475 | 1.571887 |
| 20 | 1 | 0 | -5.936124 | -2.456835 | -0.187872 |
| 21 | 1 | 0 | -4.973110 | -3.574648 | 0.802277 |
| 22 | 6 | 0 | -4.697597 | 0.475683 | 0.748537 |
| 23 | 1 | 0 | -3.921949 | 1.224700 | 0.938251 |
| 24 | 1 | 0 | -5.115129 | 0.660547 | -0.246428 |
| 25 | 1 | 0 | -5.498177 | 0.616505 | 1.484737 |
| 26 | 6 | 0 | -2.933520 | -1.519058 | 2.376917 |
| 27 | 1 | 0 | -2.471715 | -2.511979 | 2.359313 |
| 28 | 1 | 0 | -2.130997 | -0.775261 | 2.406425 |
| 29 | 1 | 0 | -3.513373 | -1.429448 | 3.302742 |
| 30 | 6 | 0 | 4.199511 | 0.965214 | 0.903158 |
| 31 | 6 | 0 | 5.130053 | -0.692319 | -0.380836 |
| 32 | 6 | 0 | 5.703278 | 1.253634 | 0.938974 |
| 33 | 6 | 0 | 6.323673 | 0.148453 | 0.084441 |
| 34 | 1 | 0 | 5.872699 | 2.261278 | 0.549593 |
| 35 | 1 | 0 | 6.033732 | 1.245073 | 1.981236 |
| 36 | 1 | 0 | 6.853255 | 0.516829 | -0.798299 |
| 37 | 1 | 0 | 7.011930 | -0.500379 | 0.632837 |
| 38 | 7 | 0 | 3.970119 | -0.150568 | 0.130952 |
| 39 | 8 | 0 | 5.202283 | -1.676950 | -1.086983 |
| 40 | 8 | 0 | 3.349021 | 1.623664 | 1.471686 |
| 41 | 1 | 0 | -0.388111 | -2.870240 | -0.489034 |
| 42 | 8 | 0 | -1.937266 | 4.633285 | -0.051197 |
| 43 | 6 | 0 | -2.638598 | 5.374251 | -1.029864 |
| 44 | 1 | 0 | -2.589113 | 6.414146 | -0.709276 |
| 45 | 1 | 0 | -2.166865 | 5.270816 | -2.013867 |
| 46 | 1 | 0 | -3.687187 | 5.058497 | -1.087801 |

| System: | **4** |
|---|---|
| Oxidant: | NIS |
| Nitrogen-donor: | $TMSN_3$ |
| Stationary point: | IN1 |
| M06–2X/6–31+G(d) energy (in a.u.): | -8275.91345176 |
| Thermal correction to Gibbs Free Energy (in a.u.): | 0.297460 |
| Number of imaginary frequencies: | 0 |

**CARTESIAN COORDINATES**

| Center Number | Atomic Number | Atomic Type | Coordinates (Angstroms) X | Y | Z |
|---|---|---|---|---|---|
| 1 | 6 | 0 | -0.780026 | -0.839574 | 0.182779 |
| 2 | 6 | 0 | -1.539151 | -1.931373 | 0.027663 |
| 3 | 6 | 0 | -1.102089 | 0.587825 | 0.047084 |
| 4 | 6 | 0 | -2.280849 | 1.128507 | 0.567386 |
| 5 | 6 | 0 | -0.178597 | 1.438450 | -0.585021 |
| 6 | 6 | 0 | -2.555786 | 2.487045 | 0.441496 |
| 7 | 1 | 0 | -2.984506 | 0.491474 | 1.095346 |
| 8 | 6 | 0 | -0.441850 | 2.788478 | -0.716733 |
| 9 | 1 | 0 | 0.775524 | 1.064760 | -0.951393 |
| 10 | 6 | 0 | -1.636736 | 3.320093 | -0.211963 |
| 11 | 1 | 0 | -3.473048 | 2.882383 | 0.861233 |
| 12 | 1 | 0 | 0.285265 | 3.441643 | -1.188695 |
| 13 | 7 | 0 | 0.618647 | -1.051084 | 0.554075 |
| 14 | 7 | 0 | 1.244309 | -1.906780 | -0.150115 |
| 15 | 7 | 0 | 1.788772 | -2.667372 | -0.754599 |
| 16 | 53 | 0 | -3.489458 | -1.969034 | -0.708710 |
| 17 | 14 | 0 | 1.426097 | -0.285088 | 2.094913 |
| 18 | 6 | 0 | -0.010235 | -0.524582 | 3.279406 |
| 19 | 1 | 0 | 0.296258 | -0.195996 | 4.280098 |
| 20 | 1 | 0 | -0.881912 | 0.073434 | 2.992244 |
| 21 | 1 | 0 | -0.315360 | -1.574259 | 3.350419 |
| 22 | 6 | 0 | 1.814665 | 1.520378 | 1.907034 |
| 23 | 1 | 0 | 2.159399 | 1.779951 | 0.902322 |
| 24 | 1 | 0 | 0.934528 | 2.126279 | 2.148575 |
| 25 | 1 | 0 | 2.610869 | 1.768554 | 2.620942 |
| 26 | 6 | 0 | 2.807428 | -1.464325 | 2.484295 |
| 27 | 1 | 0 | 2.409521 | -2.433994 | 2.805873 |
| 28 | 1 | 0 | 3.486625 | -1.624888 | 1.639764 |
| 29 | 1 | 0 | 3.388388 | -1.055427 | 3.319858 |
| 30 | 6 | 0 | 4.269567 | -0.915743 | -0.748408 |
| 31 | 6 | 0 | 3.504482 | 1.122443 | -1.187792 |
| 32 | 6 | 0 | 5.406757 | -0.288167 | -1.563328 |
| 33 | 6 | 0 | 4.881150 | 1.109288 | -1.866674 |
| 34 | 1 | 0 | 6.321999 | -0.301901 | -0.964304 |
| 35 | 1 | 0 | 5.589650 | -0.901520 | -2.450298 |
| 36 | 1 | 0 | 5.489361 | 1.917109 | -1.449506 |
| 37 | 1 | 0 | 4.752188 | 1.316721 | -2.932857 |
| 38 | 7 | 0 | 3.226932 | -0.059506 | -0.561654 |
| 39 | 8 | 0 | 2.758308 | 2.101672 | -1.218374 |
| 40 | 8 | 0 | 4.313825 | -2.073308 | -0.320853 |
| 41 | 1 | 0 | -1.147617 | -2.919601 | 0.243691 |
| 42 | 8 | 0 | -1.812981 | 4.649138 | -0.384029 |
| 43 | 6 | 0 | -3.000264 | 5.236650 | 0.108877 |
| 44 | 1 | 0 | -2.938155 | 6.294032 | -0.145685 |
| 45 | 1 | 0 | -3.884592 | 4.796215 | -0.366556 |
| 46 | 1 | 0 | -3.073079 | 5.125859 | 1.197280 |

| System: | **4** |
|---|---|
| Oxidant: | NIS |
| Nitrogen-donor: | $TMSN_3$ |
| Stationary point: | TS2 |
| M06–2X/6–31+G(d) energy (in a.u.): | -8275.91092942 |
| Thermal correction to Gibbs Free Energy (in a.u.): | 0.299068 |
| Number of imaginary frequencies: | 1 (–77 $cm^{-1}$) |

**CARTESIAN COORDINATES**

| Center Number | Atomic Number | Atomic Type | Coordinates (Angstroms) X | Y | Z |
|---|---|---|---|---|---|
| 1 | 6 | 0 | -0.682379 | -0.780795 | 0.123600 |
| 2 | 6 | 0 | -1.305408 | -1.966451 | 0.081344 |
| 3 | 6 | 0 | -1.210611 | 0.582830 | -0.031213 |
| 4 | 6 | 0 | -2.428148 | 0.960252 | 0.542103 |
| 5 | 6 | 0 | -0.459863 | 1.543904 | -0.729307 |
| 6 | 6 | 0 | -2.911538 | 2.257636 | 0.406062 |
| 7 | 1 | 0 | -2.996564 | 0.246070 | 1.129622 |
| 8 | 6 | 0 | -0.932971 | 2.836109 | -0.872079 |
| 9 | 1 | 0 | 0.517102 | 1.302956 | -1.143737 |
| 10 | 6 | 0 | -2.164358 | 3.200613 | -0.311875 |
| 11 | 1 | 0 | -3.853617 | 2.522527 | 0.870981 |
| 12 | 1 | 0 | -0.351839 | 3.581749 | -1.405102 |
| 13 | 7 | 0 | 0.763718 | -0.798007 | 0.339626 |
| 14 | 7 | 0 | 1.380628 | -1.685237 | -0.330218 |
| 15 | 7 | 0 | 1.916633 | -2.481768 | -0.896199 |
| 16 | 53 | 0 | -3.284217 | -2.311521 | -0.478801 |
| 17 | 14 | 0 | 1.700416 | 0.098873 | 1.785082 |
| 18 | 6 | 0 | 0.210489 | -0.006859 | 2.952124 |
| 19 | 1 | 0 | 0.559238 | 0.305285 | 3.945361 |
| 20 | 1 | 0 | -0.609575 | 0.662540 | 2.673200 |
| 21 | 1 | 0 | -0.188442 | -1.023515 | 3.049542 |
| 22 | 6 | 0 | 2.108018 | 1.910890 | 1.648028 |
| 23 | 1 | 0 | 1.706275 | 2.359753 | 0.737020 |
| 24 | 1 | 0 | 1.690776 | 2.429204 | 2.518793 |
| 25 | 1 | 0 | 3.192845 | 2.047977 | 1.621382 |
| 26 | 6 | 0 | 2.966594 | -1.081279 | 2.482115 |
| 27 | 1 | 0 | 2.885811 | -2.090808 | 2.067277 |
| 28 | 1 | 0 | 3.984157 | -0.749636 | 2.267868 |
| 29 | 1 | 0 | 2.818045 | -1.141065 | 3.566648 |
| 30 | 6 | 0 | 4.360241 | -0.781564 | -0.483637 |
| 31 | 6 | 0 | 3.436202 | 1.134666 | -1.160568 |
| 32 | 6 | 0 | 5.423510 | -0.188501 | -1.413811 |
| 33 | 6 | 0 | 4.788665 | 1.115072 | -1.882976 |
| 34 | 1 | 0 | 6.349498 | -0.053324 | -0.846892 |
| 35 | 1 | 0 | 5.633930 | -0.900595 | -2.216275 |
| 36 | 1 | 0 | 5.348207 | 2.011934 | -1.601044 |
| 37 | 1 | 0 | 4.616248 | 1.165164 | -2.961564 |
| 38 | 7 | 0 | 3.303386 | 0.072475 | -0.305599 |
| 39 | 8 | 0 | 2.587534 | 1.999282 | -1.362620 |
| 40 | 8 | 0 | 4.440759 | -1.905655 | 0.004205 |
| 41 | 1 | 0 | -0.770661 | -2.884573 | 0.298637 |
| 42 | 8 | 0 | -2.544269 | 4.484234 | -0.500324 |
| 43 | 6 | 0 | -3.772478 | 4.906675 | 0.058221 |
| 44 | 1 | 0 | -3.879837 | 5.955624 | -0.215410 |
| 45 | 1 | 0 | -4.610716 | 4.332544 | -0.353587 |
| 46 | 1 | 0 | -3.761712 | 4.812285 | 1.150439 |

| System: | **4** |
|---|---|
| Oxidant: | NIS |
| Nitrogen-donor: | $TMSN_3$ |
| Stationary point: | IN2 |
| M06–2X/6–31+G(d) energy (in a.u.): | -7506.83711345 |
| Thermal correction to Gibbs Free Energy (in a.u.): | 0.118923 |
| Number of imaginary frequencies: | 0 |

**CARTESIAN COORDINATES**

| Center Number | Atomic Number | Atomic Type | Coordinates (Angstroms) X | Y | Z |
|---|---|---|---|---|---|
| 1 | 6 | 0 | -0.741155 | 1.232548 | 0.176050 |
| 2 | 6 | 0 | -1.886261 | 0.532224 | 0.113331 |
| 3 | 6 | 0 | 0.640768 | 0.704014 | 0.186018 |
| 4 | 6 | 0 | 1.614197 | 1.291209 | -0.621721 |
| 5 | 6 | 0 | 1.014327 | -0.354000 | 1.027069 |
| 6 | 6 | 0 | 2.926702 | 0.821540 | -0.631105 |
| 7 | 1 | 0 | 1.344745 | 2.130153 | -1.256493 |
| 8 | 6 | 0 | 2.316264 | -0.824100 | 1.036496 |
| 9 | 1 | 0 | 0.281733 | -0.798885 | 1.692829 |
| 10 | 6 | 0 | 3.279401 | -0.244401 | 0.200687 |
| 11 | 1 | 0 | 3.654857 | 1.294249 | -1.279525 |
| 12 | 1 | 0 | 2.618398 | -1.636261 | 1.689457 |
| 13 | 7 | 0 | -0.740522 | 2.660871 | 0.230166 |
| 14 | 7 | 0 | -1.839158 | 3.219950 | 0.116853 |
| 15 | 53 | 0 | -2.061414 | -1.530166 | -0.175270 |
| 16 | 7 | 0 | -2.789937 | 3.820140 | 0.027736 |
| 17 | 8 | 0 | 4.524405 | -0.776462 | 0.276083 |
| 18 | 6 | 0 | 5.533197 | -0.216779 | -0.539341 |
| 19 | 1 | 0 | 6.439362 | -0.783079 | -0.326478 |
| 20 | 1 | 0 | 5.281353 | -0.314681 | -1.602054 |
| 21 | 1 | 0 | 5.696055 | 0.839989 | -0.296257 |
| 22 | 1 | 0 | -2.854134 | 1.018770 | 0.150966 |

| System: | **4** |
|---|---|
| Oxidant: | NIS |
| Nitrogen-donor: | $TMSN_3$ |
| Stationary point: | TS3 |
| M06–2X/6–31+G(d) energy (in a.u.): | -7506.78547981 |
| Thermal correction to Gibbs Free Energy (in a.u.): | 0.113953 |
| Number of imaginary frequencies: | 1 (–534 $cm^{-1}$) |

**CARTESIAN COORDINATES**

| Center Number | Atomic Number | Atomic Type | Coordinates (Angstroms) X | Y | Z |
|---|---|---|---|---|---|
| 1 | 6 | 0 | 0.371911 | 0.265781 | 0.165953 |
| 2 | 6 | 0 | 1.319550 | -0.729881 | 0.254092 |
| 3 | 6 | 0 | -1.071975 | -0.063124 | 0.077576 |
| 4 | 6 | 0 | -2.023609 | 0.712891 | 0.739566 |
| 5 | 6 | 0 | -1.503628 | -1.150748 | -0.694948 |
| 6 | 6 | 0 | -3.382687 | 0.416686 | 0.648690 |
| 7 | 1 | 0 | -1.693218 | 1.545300 | 1.355296 |
| 8 | 6 | 0 | -2.850042 | -1.457946 | -0.793594 |
| 9 | 1 | 0 | -0.770859 | -1.743094 | -1.237354 |
| 10 | 6 | 0 | -3.798226 | -0.674731 | -0.120898 |
| 11 | 1 | 0 | -4.097937 | 1.030917 | 1.183014 |
| 12 | 1 | 0 | -3.200393 | -2.291044 | -1.394173 |
| 13 | 7 | 0 | 0.854734 | 1.479123 | 0.431026 |
| 14 | 7 | 0 | -0.028638 | 2.658895 | -0.562983 |
| 15 | 53 | 0 | 3.376126 | -0.477763 | 0.036860 |
| 16 | 7 | 0 | -0.291679 | 3.723382 | -0.689253 |
| 17 | 8 | 0 | -5.090972 | -1.050144 | -0.278710 |
| 18 | 6 | 0 | -6.087070 | -0.288849 | 0.373535 |
| 19 | 1 | 0 | -7.037437 | -0.753962 | 0.113874 |
| 20 | 1 | 0 | -5.952716 | -0.312845 | 1.461428 |
| 21 | 1 | 0 | -6.080350 | 0.750657 | 0.024853 |
| 22 | 1 | 0 | 1.039768 | -1.713618 | 0.617780 |

| System: | **4** |
|---|---|
| Oxidant: | NIS |
| Nitrogen-donor: | $TMSN_3$ |
| Stationary point: | IN3 |
| M06–2X/6–31+G(d) energy (in a.u.): | -7397.40581569 |
| Thermal correction to Gibbs Free Energy (in a.u.): | 0.111904 |
| Number of imaginary frequencies: | 0 |

**CARTESIAN COORDINATES**

| Center Number | Atomic Number | Atomic Type | Coordinates (Angstroms) X | Y | Z |
|---|---|---|---|---|---|
| 1 | 6 | 0 | -0.357886 | 1.066072 | 0.471988 |
| 2 | 6 | 0 | -1.721166 | 0.887840 | 0.864703 |
| 3 | 7 | 0 | -1.109531 | 2.049334 | 0.189918 |
| 4 | 6 | 0 | 0.989259 | 0.571166 | 0.340801 |
| 5 | 6 | 0 | 1.321121 | -0.700487 | 0.832663 |
| 6 | 6 | 0 | 1.966872 | 1.351630 | -0.282503 |
| 7 | 6 | 0 | 2.611711 | -1.179050 | 0.704749 |
| 8 | 1 | 0 | 0.553499 | -1.305691 | 1.307005 |
| 9 | 6 | 0 | 3.267729 | 0.880494 | -0.414354 |
| 10 | 1 | 0 | 1.700180 | 2.332664 | -0.665518 |
| 11 | 6 | 0 | 3.591440 | -0.390333 | 0.081844 |
| 12 | 1 | 0 | 2.895391 | -2.159155 | 1.072879 |
| 13 | 1 | 0 | 4.013129 | 1.498677 | -0.899904 |
| 14 | 53 | 0 | -3.045054 | -0.412172 | -0.257174 |
| 15 | 8 | 0 | 4.821648 | -0.941239 | 0.004336 |
| 16 | 6 | 0 | 5.852601 | -0.197289 | -0.617735 |
| 17 | 1 | 0 | 6.035645 | 0.742362 | -0.084161 |
| 18 | 1 | 0 | 6.742258 | -0.823741 | -0.569299 |
| 19 | 1 | 0 | 5.607967 | 0.012704 | -1.665167 |
| 20 | 1 | 0 | -2.096781 | 0.991584 | 1.877309 |

| System: | **4** |
|---|---|
| Oxidant: | NIS |
| Nitrogen-donor: | $TMSN_3$ |
| Stationary point: | TS4 |
| M06–2X/6–31+G(d) energy (in a.u.): | -7970.69051297 |
| Thermal correction to Gibbs Free Energy (in a.u.): | 0.223591 |
| Number of imaginary frequencies: | 1 (–280 $cm^{-1}$) |

**CARTESIAN COORDINATES**

| Center Number | Atomic Number | Atomic Type | Coordinates (Angstroms) X | Y | Z |
|---|---|---|---|---|---|
| 1 | 6 | 0 | 0.246235 | -0.543751 | -1.489773 |
| 2 | 6 | 0 | -0.711530 | 0.085824 | -2.251969 |
| 3 | 7 | 0 | -0.516742 | -1.184625 | -2.451588 |
| 4 | 6 | 0 | 1.588774 | -0.492999 | -0.994617 |
| 5 | 6 | 0 | 2.059769 | 0.722004 | -0.479648 |
| 6 | 6 | 0 | 2.405863 | -1.639966 | -0.981848 |
| 7 | 6 | 0 | 3.357193 | 0.812019 | 0.012110 |
| 8 | 1 | 0 | 1.390249 | 1.581106 | -0.450647 |
| 9 | 6 | 0 | 3.691168 | -1.553673 | -0.495495 |
| 10 | 1 | 0 | 2.018483 | -2.581177 | -1.361681 |
| 11 | 6 | 0 | 4.173619 | -0.325613 | 0.003997 |
| 12 | 1 | 0 | 3.709131 | 1.755576 | 0.410306 |
| 13 | 1 | 0 | 4.355022 | -2.411293 | -0.478692 |
| 14 | 53 | 0 | -1.685347 | 2.199796 | 0.034062 |
| 15 | 7 | 0 | -0.697194 | -1.239030 | 0.276062 |
| 16 | 7 | 0 | -0.107006 | -0.769387 | 1.252564 |
| 17 | 7 | 0 | 0.517286 | -0.320308 | 2.074707 |
| 18 | 14 | 0 | -2.429938 | -1.792310 | 0.482314 |
| 19 | 6 | 0 | -2.307845 | -3.661678 | 0.543324 |
| 20 | 1 | 0 | -1.699089 | -3.998308 | 1.389174 |
| 21 | 1 | 0 | -3.302232 | -4.112083 | 0.642870 |
| 22 | 1 | 0 | -1.854960 | -4.047754 | -0.376699 |
| 23 | 6 | 0 | -3.426745 | -1.256640 | -1.000703 |
| 24 | 1 | 0 | -4.466177 | -1.562102 | -0.826251 |
| 25 | 1 | 0 | -3.411070 | -0.167658 | -1.111840 |
| 26 | 1 | 0 | -3.095720 | -1.735162 | -1.927507 |
| 27 | 6 | 0 | -3.031205 | -1.060109 | 2.089155 |
| 28 | 1 | 0 | -2.977600 | 0.033634 | 2.047388 |
| 29 | 1 | 0 | -4.074364 | -1.349585 | 2.261050 |
| 30 | 1 | 0 | -2.444886 | -1.411127 | 2.945952 |
| 31 | 8 | 0 | 5.437470 | -0.350323 | 0.459468 |
| 32 | 6 | 0 | 5.985138 | 0.842387 | 0.998867 |
| 33 | 1 | 0 | 6.998980 | 0.589527 | 1.304959 |
| 34 | 1 | 0 | 6.014316 | 1.634156 | 0.242609 |
| 35 | 1 | 0 | 5.408830 | 1.176922 | 1.867942 |
| 36 | 1 | 0 | -1.283202 | 0.937678 | -2.594962 |

| System: | **4** |
|---|---|
| Oxidant: | NIS |
| Nitrogen-donor: | $TMSN_3$ |
| Stationary point: | IN4 |
| M06–2X/6–31+G(d) energy (in a.u.): | -1050.56050129 |
| Thermal correction to Gibbs Free Energy (in a.u.): | 0.228955 |
| Number of imaginary frequencies: | 0 |

**CARTESIAN COORDINATES**

| Center Number | Atomic Number | Atomic Type | Coordinates (Angstroms) X | Y | Z |
|---|---|---|---|---|---|
| 1 | 6 | 0 | -0.502249 | 1.101387 | 0.183901 |
| 2 | 6 | 0 | -0.880328 | 2.476160 | 0.392734 |
| 3 | 7 | 0 | -1.124772 | 2.055375 | -0.768247 |
| 4 | 6 | 0 | 0.876297 | 0.570335 | 0.033772 |
| 5 | 6 | 0 | 1.114364 | -0.641328 | -0.635948 |
| 6 | 6 | 0 | 1.957257 | 1.288821 | 0.538941 |
| 7 | 6 | 0 | 2.402501 | -1.113652 | -0.793778 |
| 8 | 1 | 0 | 0.284522 | -1.211242 | -1.049151 |
| 9 | 6 | 0 | 3.262889 | 0.829306 | 0.381563 |
| 10 | 1 | 0 | 1.789266 | 2.227021 | 1.063399 |
| 11 | 6 | 0 | 3.490156 | -0.379170 | -0.287445 |
| 12 | 1 | 0 | 2.606987 | -2.043862 | -1.313316 |
| 13 | 1 | 0 | 4.084274 | 1.410833 | 0.782425 |
| 14 | 7 | 0 | -1.511232 | 0.051053 | 0.587380 |
| 15 | 7 | 0 | -1.144232 | -0.674194 | 1.557410 |
| 16 | 7 | 0 | -0.810858 | -1.319077 | 2.404243 |
| 17 | 14 | 0 | -3.090801 | -0.411202 | -0.394120 |
| 18 | 6 | 0 | -4.205542 | 1.065315 | -0.234371 |
| 19 | 1 | 0 | -5.210547 | 0.790245 | -0.576136 |
| 20 | 1 | 0 | -4.292801 | 1.396759 | 0.805618 |
| 21 | 1 | 0 | -3.862033 | 1.903079 | -0.847016 |
| 22 | 6 | 0 | -3.662004 | -1.905614 | 0.558870 |
| 23 | 1 | 0 | -4.575186 | -2.286650 | 0.086404 |
| 24 | 1 | 0 | -2.935205 | -2.725218 | 0.543455 |
| 25 | 1 | 0 | -3.918423 | -1.675474 | 1.598765 |
| 26 | 6 | 0 | -2.464061 | -0.760548 | -2.105630 |
| 27 | 1 | 0 | -1.892235 | -1.692462 | -2.151488 |
| 28 | 1 | 0 | -3.317937 | -0.863181 | -2.785938 |
| 29 | 1 | 0 | -1.843659 | 0.059858 | -2.480511 |
| 30 | 8 | 0 | 4.700400 | -0.918933 | -0.494212 |
| 31 | 6 | 0 | 5.846169 | -0.224341 | -0.020454 |
| 32 | 1 | 0 | 6.700010 | -0.836506 | -0.304423 |
| 33 | 1 | 0 | 5.925116 | 0.760640 | -0.492619 |
| 34 | 1 | 0 | 5.813591 | -0.119679 | 1.069270 |
| 35 | 1 | 0 | -0.962760 | 3.381998 | 0.984198 |

| System: | **4** |
|---|---|
| Oxidant: | NIS |
| Nitrogen-donor: | $TMSN_3$ |
| Stationary point: | TS5 |
| M06–2X/6–31+G(d) energy (in a.u.): | -1410.70496037 |
| Thermal correction to Gibbs Free Energy (in a.u.): | 0.305923 |
| Number of imaginary frequencies: | 1 (–56 $cm^{-1}$) |

**CARTESIAN COORDINATES**

| Center Number | Atomic Number | Atomic Type | X | Y | Z |
|---|---|---|---|---|---|
| 1 | 6 | 0 | 0.704240 | -0.814828 | 0.608173 |
| 2 | 6 | 0 | 0.144326 | -2.030362 | 1.150863 |
| 3 | 7 | 0 | 0.246548 | -1.103642 | 1.993469 |
| 4 | 6 | 0 | 2.153261 | -0.589497 | 0.325444 |
| 5 | 6 | 0 | 2.597124 | 0.409671 | -0.536939 |
| 6 | 6 | 0 | 3.109881 | -1.404232 | 0.950650 |
| 7 | 6 | 0 | 3.957700 | 0.602393 | -0.785867 |
| 8 | 1 | 0 | 1.884778 | 1.071697 | -1.021590 |
| 9 | 6 | 0 | 4.460825 | -1.222592 | 0.716057 |
| 10 | 1 | 0 | 2.792963 | -2.183116 | 1.638612 |
| 11 | 6 | 0 | 4.895929 | -0.216319 | -0.157086 |
| 12 | 1 | 0 | 4.263387 | 1.392440 | -1.461651 |
| 13 | 1 | 0 | 5.205691 | -1.846560 | 1.199246 |
| 14 | 7 | 0 | -0.213159 | 0.083175 | -0.125822 |
| 15 | 7 | 0 | -0.442546 | -0.270751 | -1.314090 |
| 16 | 7 | 0 | -0.627613 | -0.509746 | -2.386918 |
| 17 | 14 | 0 | -1.315872 | 1.445492 | 0.684733 |
| 18 | 6 | 0 | -1.709082 | 2.619641 | -0.717350 |
| 19 | 1 | 0 | -2.180175 | 2.132664 | -1.573009 |
| 20 | 1 | 0 | -2.428165 | 3.364191 | -0.359003 |
| 21 | 1 | 0 | -0.795557 | 3.140032 | -1.028315 |
| 22 | 6 | 0 | 0.185365 | 2.224286 | 1.553164 |
| 23 | 1 | 0 | -0.153142 | 3.133930 | 2.067495 |
| 24 | 1 | 0 | 0.612580 | 1.562950 | 2.315918 |
| 25 | 1 | 0 | 0.986168 | 2.514351 | 0.863952 |
| 26 | 6 | 0 | -2.431843 | 0.986394 | 2.118214 |
| 27 | 1 | 0 | -2.388146 | -0.092601 | 2.295118 |
| 28 | 1 | 0 | -2.098421 | 1.498175 | 3.027150 |
| 29 | 1 | 0 | -3.466546 | 1.260124 | 1.895081 |
| 30 | 7 | 0 | -2.963239 | -0.062889 | -0.384049 |
| 31 | 6 | 0 | -4.238323 | 0.440458 | -0.524693 |
| 32 | 6 | 0 | -5.256700 | -0.682314 | -0.755513 |
| 33 | 6 | 0 | -4.422250 | -1.950912 | -0.617454 |
| 34 | 6 | 0 | -3.005579 | -1.418570 | -0.393646 |
| 35 | 8 | 0 | -2.026617 | -2.163561 | -0.248149 |
| 36 | 1 | 0 | -4.702489 | -2.573177 | 0.237842 |
| 37 | 1 | 0 | -6.060953 | -0.589695 | -0.021024 |
| 38 | 8 | 0 | -4.532403 | 1.623496 | -0.453218 |
| 39 | 1 | 0 | -4.424996 | -2.593436 | -1.502430 |
| 40 | 1 | 0 | -5.701965 | -0.554333 | -1.746495 |
| 41 | 1 | 0 | -0.156911 | -3.064240 | 1.049166 |
| 42 | 8 | 0 | 6.237553 | -0.117349 | -0.323949 |
| 43 | 6 | 0 | 6.723042 | 0.890976 | -1.187654 |
| 44 | 1 | 0 | 7.808452 | 0.798471 | -1.175697 |
| 45 | 1 | 0 | 6.354027 | 0.745311 | -2.209849 |
| 46 | 1 | 0 | 6.435932 | 1.887061 | -0.830540 |

| System: | **4** |
|---|---|
| Oxidant: | NIS |
| Nitrogen-donor: | $TMSN_3$ |
| Stationary point: | IN5 |
| M06–2X/6–31+G(d) energy (in a.u.): | -641.611479186 |
| Thermal correction to Gibbs Free Energy (in a.u.): | 0.124360 |
| Number of imaginary frequencies: | 0 |

**CARTESIAN COORDINATES**

| Center Number | Atomic Number | Atomic Type | Coordinates (Angstroms) X | Y | Z |
|---|---|---|---|---|---|
| 1 | 6 | 0 | 2.316890 | 1.506988 | 0.642489 |
| 2 | 6 | 0 | 1.725795 | 0.328339 | 0.048199 |
| 3 | 7 | 0 | 2.239433 | 1.575153 | -0.613601 |
| 4 | 6 | 0 | 0.255248 | 0.072637 | 0.037429 |
| 5 | 6 | 0 | -0.261120 | -1.228274 | 0.105632 |
| 6 | 6 | 0 | -0.636273 | 1.140946 | -0.042721 |
| 7 | 6 | 0 | -1.629412 | -1.446371 | 0.100288 |
| 8 | 1 | 0 | 0.419309 | -2.071182 | 0.158016 |
| 9 | 6 | 0 | -2.015844 | 0.934204 | -0.053548 |
| 10 | 1 | 0 | -0.260703 | 2.157867 | -0.112563 |
| 11 | 6 | 0 | -2.516699 | -0.366976 | 0.020367 |
| 12 | 1 | 0 | -2.039034 | -2.450098 | 0.152196 |
| 13 | 1 | 0 | -2.678255 | 1.789191 | -0.123221 |
| 14 | 7 | 0 | 2.482511 | -0.900618 | -0.138340 |
| 15 | 7 | 0 | 3.707166 | -0.773202 | -0.082351 |
| 16 | 7 | 0 | 4.835556 | -0.743772 | -0.044188 |
| 17 | 1 | 0 | 2.652185 | 2.116710 | 1.477044 |
| 18 | 8 | 0 | -3.838041 | -0.682582 | 0.020138 |
| 19 | 6 | 0 | -4.767816 | 0.376370 | -0.071460 |
| 20 | 1 | 0 | -4.637818 | 0.936855 | -1.005094 |
| 21 | 1 | 0 | -5.753659 | -0.087727 | -0.060162 |
| 22 | 1 | 0 | -4.674967 | 1.058942 | 0.781987 |

| System: | **4** |
|---|---|
| Oxidant: | NIS |
| Nitrogen-donor: | $TMSN_3$ |
| Stationary point: | TS6 |
| M06–2X/6–31+G(d) energy (in a.u.): | -641.550012902 |
| Thermal correction to Gibbs Free Energy (in a.u.): | 0.118441 |
| Number of imaginary frequencies: | 1 (–618 $cm^{-1}$) |

**CARTESIAN COORDINATES**

| Center Number | Atomic Number | Atomic Type | Coordinates (Angstroms) X | Y | Z |
|---|---|---|---|---|---|
| 1 | 6 | 0 | 2.481027 | 1.568577 | 0.556191 |
| 2 | 6 | 0 | 1.804516 | 0.524165 | -0.272641 |
| 3 | 7 | 0 | 2.342235 | 2.031386 | -0.572241 |
| 4 | 6 | 0 | 0.313589 | 0.413269 | -0.150536 |
| 5 | 6 | 0 | -0.294745 | -0.824448 | -0.352551 |
| 6 | 6 | 0 | -0.500141 | 1.510828 | 0.168901 |
| 7 | 6 | 0 | -1.676074 | -0.982739 | -0.236631 |
| 8 | 1 | 0 | 0.325436 | -1.675238 | -0.617139 |
| 9 | 6 | 0 | -1.874497 | 1.368325 | 0.281886 |
| 10 | 1 | 0 | -0.061434 | 2.493811 | 0.312312 |
| 11 | 6 | 0 | -2.472220 | 0.118931 | 0.082959 |
| 12 | 1 | 0 | -2.111943 | -1.961199 | -0.403345 |
| 13 | 1 | 0 | -2.510668 | 2.214626 | 0.521154 |
| 14 | 7 | 0 | 2.552091 | -0.444514 | -0.849291 |
| 15 | 7 | 0 | 3.366165 | -1.391801 | 0.367153 |
| 16 | 7 | 0 | 4.056995 | -2.234948 | 0.548125 |
| 17 | 1 | 0 | 2.810999 | 1.776680 | 1.569303 |
| 18 | 8 | 0 | -3.824449 | 0.078367 | 0.219071 |
| 19 | 6 | 0 | -4.468622 | -1.160564 | 0.010424 |
| 20 | 1 | 0 | -5.532104 | -0.975325 | 0.159630 |
| 21 | 1 | 0 | -4.300434 | -1.526933 | -1.009511 |
| 22 | 1 | 0 | -4.123647 | -1.912289 | 0.730803 |

| System: | **2** |
|---|---|
| Oxidant: | NCS |
| Nitrogen-donor: | $TMSN_3$ |
| Stationary point: | R |
| M06–2X/6–31+G(d) energy (in a.u.): | -2011.24416299 |
| Thermal correction to Gibbs Free Energy (in a.u.): | 0.382148 |
| Number of imaginary frequencies: | 0 |

**CARTESIAN COORDINATES**

| Center Number | Atomic Number | Atomic Type | Coordinates (Angstroms) X | Y | Z |
|---|---|---|---|---|---|
| 1 | 6 | 0 | 0.057903 | 0.411363 | -0.845762 |
| 2 | 6 | 0 | -0.680575 | 1.362032 | -0.679850 |
| 3 | 6 | 0 | 1.011868 | -0.636955 | -1.069973 |
| 4 | 6 | 0 | 2.213289 | -0.331676 | -1.726217 |
| 5 | 6 | 0 | 0.798877 | -1.954067 | -0.628811 |
| 6 | 6 | 0 | 3.183879 | -1.310915 | -1.915461 |
| 7 | 1 | 0 | 2.377538 | 0.680053 | -2.085468 |
| 8 | 6 | 0 | 1.773278 | -2.923504 | -0.833719 |
| 9 | 1 | 0 | -0.122395 | -2.218613 | -0.120231 |
| 10 | 6 | 0 | 2.983522 | -2.620642 | -1.470998 |
| 11 | 1 | 0 | 4.112966 | -1.045875 | -2.415091 |
| 12 | 1 | 0 | 1.590905 | -3.937378 | -0.484178 |
| 13 | 7 | 0 | 4.609033 | 1.374861 | -0.142814 |
| 14 | 7 | 0 | 3.642347 | 2.117715 | -0.195838 |
| 15 | 7 | 0 | 2.765405 | 2.824496 | -0.320715 |
| 16 | 6 | 0 | -1.467322 | 2.546596 | -0.485954 |
| 17 | 6 | 0 | -2.870358 | 2.511759 | -0.440638 |
| 18 | 6 | 0 | -0.813155 | 3.779711 | -0.345887 |
| 19 | 6 | 0 | -3.590213 | 3.685881 | -0.258529 |
| 20 | 1 | 0 | -3.395821 | 1.568436 | -0.550060 |
| 21 | 6 | 0 | -1.549586 | 4.946123 | -0.164715 |
| 22 | 1 | 0 | 0.271557 | 3.809455 | -0.380652 |
| 23 | 6 | 0 | -2.945943 | 4.920630 | -0.117105 |
| 24 | 1 | 0 | -4.676570 | 3.642213 | -0.225616 |
| 25 | 1 | 0 | -1.027861 | 5.894136 | -0.058136 |
| 26 | 14 | 0 | 4.866995 | 0.255037 | 1.223520 |
| 27 | 6 | 0 | 5.662830 | 1.236694 | 2.611147 |
| 28 | 1 | 0 | 5.889029 | 0.589536 | 3.466548 |
| 29 | 1 | 0 | 6.597651 | 1.699767 | 2.278800 |
| 30 | 1 | 0 | 4.997849 | 2.033424 | 2.962863 |
| 31 | 6 | 0 | 6.032841 | -1.041970 | 0.554987 |
| 32 | 1 | 0 | 5.557915 | -1.602922 | -0.256549 |
| 33 | 1 | 0 | 6.945506 | -0.580291 | 0.163683 |
| 34 | 1 | 0 | 6.318828 | -1.751865 | 1.339050 |
| 35 | 6 | 0 | 3.220035 | -0.450354 | 1.763785 |
| 36 | 1 | 0 | 2.454736 | 0.329870 | 1.859559 |
| 37 | 1 | 0 | 2.851554 | -1.189433 | 1.044846 |
| 38 | 1 | 0 | 3.315513 | -0.940883 | 2.739630 |
| 39 | 6 | 0 | -3.427044 | -3.490684 | 0.641816 |
| 40 | 6 | 0 | -5.111956 | -1.848996 | 0.403397 |
| 41 | 6 | 0 | -4.738592 | -4.196463 | 0.948045 |
| 42 | 6 | 0 | -5.832755 | -3.129770 | 0.795063 |
| 43 | 1 | 0 | -4.847433 | -5.032349 | 0.252559 |
| 44 | 1 | 0 | -4.676058 | -4.612770 | 1.956498 |
| 45 | 1 | 0 | -6.561475 | -3.364175 | 0.015218 |
| 46 | 1 | 0 | -6.386823 | -2.941367 | 1.717954 |
| 47 | 7 | 0 | -3.749767 | -2.163499 | 0.346156 |
| 48 | 8 | 0 | -5.578470 | -0.764454 | 0.179058 |
| 49 | 8 | 0 | -2.313042 | -3.944389 | 0.642377 |
| 50 | 6 | 0 | -3.747618 | 6.181798 | 0.081370 |
| 51 | 1 | 0 | -3.097222 | 7.057044 | 0.161046 |
| 52 | 1 | 0 | -4.435408 | 6.346188 | -0.755043 |
| 53 | 1 | 0 | -4.350574 | 6.124202 | 0.994061 |
| 54 | 6 | 0 | 4.036720 | -3.682823 | -1.659208 |
| 55 | 1 | 0 | 4.437375 | -4.014403 | -0.694052 |
| 56 | 1 | 0 | 3.622567 | -4.563865 | -2.160422 |

| | | | | | |
|---|---|---|---|---|---|
| 57 | 1 | 0 | 4.871863 | -3.312419 | -2.260560 |
| 58 | 17 | 0 | -2.571189 | -1.024888 | -0.056191 |

---------------------------------------------------------------------

| System: | **2** |
|---|---|
| Oxidant: | NCS |
| Nitrogen-donor: | $TMSN_3$ |
| Stationary point: | TS1 |
| M06–2X/6–31+G(d) energy (in a.u.): | -2011.18322325 |
| Thermal correction to Gibbs Free Energy (in a.u.): | 0.385619 |
| Number of imaginary frequencies: | 1 (–424 $cm^{-1}$) |

**CARTESIAN COORDINATES**

| Center Number | Atomic Number | Atomic Type | Coordinates (Angstroms) X | Y | Z |
|---|---|---|---|---|---|
| 1 | 6 | 0 | -0.657328 | 0.502510 | -0.319839 |
| 2 | 6 | 0 | -0.220035 | -0.694001 | -0.130964 |
| 3 | 6 | 0 | -0.178871 | 1.883007 | -0.339850 |
| 4 | 6 | 0 | -0.699080 | 2.848479 | -1.209038 |
| 5 | 6 | 0 | 0.864859 | 2.246888 | 0.528290 |
| 6 | 6 | 0 | -0.192693 | 4.145917 | -1.208773 |
| 7 | 1 | 0 | -1.496924 | 2.582618 | -1.896661 |
| 8 | 6 | 0 | 1.372414 | 3.538909 | 0.505971 |
| 9 | 1 | 0 | 1.297515 | 1.512279 | 1.199006 |
| 10 | 6 | 0 | 0.851228 | 4.512297 | -0.355409 |
| 11 | 1 | 0 | -0.607730 | 4.880900 | -1.894521 |
| 12 | 1 | 0 | 2.197936 | 3.786869 | 1.168735 |
| 13 | 7 | 0 | -2.444896 | 0.631093 | -0.595207 |
| 14 | 7 | 0 | -2.883624 | -0.197893 | -1.407784 |
| 15 | 7 | 0 | -3.199294 | -0.968441 | -2.163946 |
| 16 | 6 | 0 | -0.866248 | -1.992134 | -0.005249 |
| 17 | 6 | 0 | -0.402692 | -3.095526 | -0.739492 |
| 18 | 6 | 0 | -1.951028 | -2.177002 | 0.863113 |
| 19 | 6 | 0 | -1.046074 | -4.322497 | -0.641757 |
| 20 | 1 | 0 | 0.466940 | -2.978969 | -1.380008 |
| 21 | 6 | 0 | -2.578967 | -3.416704 | 0.964653 |
| 22 | 1 | 0 | -2.285376 | -1.345897 | 1.479722 |
| 23 | 6 | 0 | -2.145716 | -4.506497 | 0.206594 |
| 24 | 1 | 0 | -0.678280 | -5.163113 | -1.225964 |
| 25 | 1 | 0 | -3.413352 | -3.540868 | 1.651892 |
| 26 | 14 | 0 | -3.563188 | 1.466733 | 0.589076 |
| 27 | 6 | 0 | -4.983356 | 0.271536 | 0.831530 |
| 28 | 1 | 0 | -5.664398 | 0.652467 | 1.600992 |
| 29 | 1 | 0 | -5.567840 | 0.145967 | -0.087034 |
| 30 | 1 | 0 | -4.636049 | -0.717218 | 1.149727 |
| 31 | 6 | 0 | -4.133342 | 3.058256 | -0.204842 |
| 32 | 1 | 0 | -3.299111 | 3.756666 | -0.324813 |
| 33 | 1 | 0 | -4.578103 | 2.877871 | -1.189793 |
| 34 | 1 | 0 | -4.893877 | 3.540218 | 0.420610 |
| 35 | 6 | 0 | -2.525171 | 1.746540 | 2.109145 |
| 36 | 1 | 0 | -2.005335 | 0.836482 | 2.425925 |
| 37 | 1 | 0 | -1.768911 | 2.517567 | 1.929116 |
| 38 | 1 | 0 | -3.164328 | 2.081093 | 2.934490 |
| 39 | 6 | 0 | 4.545782 | 0.094973 | 0.720436 |
| 40 | 6 | 0 | 4.599221 | -1.914780 | -0.418542 |
| 41 | 6 | 0 | 6.031512 | -0.264996 | 0.639649 |
| 42 | 6 | 0 | 6.066516 | -1.602079 | -0.106440 |
| 43 | 1 | 0 | 6.555378 | 0.541482 | 0.119444 |
| 44 | 1 | 0 | 6.432362 | -0.316470 | 1.655454 |
| 45 | 1 | 0 | 6.621088 | -1.566742 | -1.047794 |
| 46 | 1 | 0 | 6.475915 | -2.424377 | 0.486421 |
| 47 | 7 | 0 | 3.827450 | -0.897792 | 0.099478 |
| 48 | 8 | 0 | 4.191164 | -2.886812 | -1.015971 |
| 49 | 8 | 0 | 4.081595 | 1.089116 | 1.243600 |
| 50 | 6 | 0 | -2.816399 | -5.852664 | 0.309697 |
| 51 | 1 | 0 | -3.678291 | -5.816663 | 0.981794 |
| 52 | 1 | 0 | -3.165047 | -6.195259 | -0.670314 |
| 53 | 1 | 0 | -2.122050 | -6.608478 | 0.692424 |
| 54 | 6 | 0 | 1.420821 | 5.907341 | -0.365916 |
| 55 | 1 | 0 | 0.862215 | 6.561353 | -1.040995 |
| 56 | 1 | 0 | 1.397350 | 6.349770 | 0.635378 |

| | | | | | |
|---|---|---|---|---|---|
| 57 | 1 | 0 | 2.466135 | 5.896082 | -0.692768 |
| 58 | 17 | 0 | 1.925009 | -0.792572 | -0.027891 |

---------------------------------------------------------------------

| System: | **2** |
|---|---|
| Oxidant: | NCS |
| Nitrogen-donor: | $TMSN_3$ |
| Stationary point: | IN1 |
| M06–2X/6–31+G(d) energy (in a.u.): | -2011.26739891 |
| Thermal correction to Gibbs Free Energy (in a.u.): | 0.396900 |
| Number of imaginary frequencies: | 0 |

**CARTESIAN COORDINATES**

| Center Number | Atomic Number | Atomic Type | Coordinates (Angstroms) X | Y | Z |
|---|---|---|---|---|---|
| 1 | 6 | 0 | -0.259463 | -1.146754 | 0.160050 |
| 2 | 6 | 0 | 0.723769 | -1.568847 | -0.655153 |
| 3 | 6 | 0 | -1.714370 | -1.323740 | 0.011467 |
| 4 | 6 | 0 | -2.483656 | -1.802506 | 1.074324 |
| 5 | 6 | 0 | -2.342712 | -0.963842 | -1.187245 |
| 6 | 6 | 0 | -3.864436 | -1.926447 | 0.943680 |
| 7 | 1 | 0 | -2.002981 | -2.079264 | 2.011085 |
| 8 | 6 | 0 | -3.721392 | -1.086128 | -1.303583 |
| 9 | 1 | 0 | -1.735595 | -0.574923 | -2.001203 |
| 10 | 6 | 0 | -4.504062 | -1.566993 | -0.245179 |
| 11 | 1 | 0 | -4.452310 | -2.304646 | 1.776328 |
| 12 | 1 | 0 | -4.205706 | -0.794768 | -2.232877 |
| 13 | 7 | 0 | 0.102862 | -0.428343 | 1.382665 |
| 14 | 7 | 0 | 1.036672 | -0.956580 | 2.051528 |
| 15 | 7 | 0 | 1.869548 | -1.378126 | 2.666483 |
| 16 | 6 | 0 | 2.151772 | -1.214965 | -0.516479 |
| 17 | 6 | 0 | 3.143308 | -2.200190 | -0.514373 |
| 18 | 6 | 0 | 2.515302 | 0.127854 | -0.352507 |
| 19 | 6 | 0 | 4.477069 | -1.848306 | -0.324201 |
| 20 | 1 | 0 | 2.869611 | -3.241862 | -0.659857 |
| 21 | 6 | 0 | 3.850953 | 0.464906 | -0.163763 |
| 22 | 1 | 0 | 1.739218 | 0.889497 | -0.396842 |
| 23 | 6 | 0 | 4.852314 | -0.513628 | -0.144890 |
| 24 | 1 | 0 | 5.239066 | -2.623738 | -0.317768 |
| 25 | 1 | 0 | 4.123174 | 1.511376 | -0.042996 |
| 26 | 14 | 0 | -0.555653 | 1.224419 | 2.156093 |
| 27 | 6 | 0 | 0.927939 | 2.344597 | 2.082131 |
| 28 | 1 | 0 | 0.682197 | 3.278687 | 2.599364 |
| 29 | 1 | 0 | 1.775520 | 1.877000 | 2.600920 |
| 30 | 1 | 0 | 1.201870 | 2.595335 | 1.058831 |
| 31 | 6 | 0 | -0.692084 | 0.622655 | 3.940869 |
| 32 | 1 | 0 | -1.354431 | -0.246489 | 4.029562 |
| 33 | 1 | 0 | 0.267641 | 0.383333 | 4.412657 |
| 34 | 1 | 0 | -1.141019 | 1.427686 | 4.536387 |
| 35 | 6 | 0 | -2.280272 | 1.520214 | 1.543870 |
| 36 | 1 | 0 | -2.538963 | 2.551347 | 1.807124 |
| 37 | 1 | 0 | -2.354801 | 1.423325 | 0.460731 |
| 38 | 1 | 0 | -2.970476 | 0.825829 | 2.033330 |
| 39 | 6 | 0 | -0.664057 | 3.206819 | -0.730920 |
| 40 | 6 | 0 | -0.111702 | 1.593113 | -2.160052 |
| 41 | 6 | 0 | -0.759586 | 3.902270 | -2.095753 |
| 42 | 6 | 0 | -0.378329 | 2.799051 | -3.073928 |
| 43 | 1 | 0 | -1.775730 | 4.285817 | -2.226940 |
| 44 | 1 | 0 | -0.084120 | 4.762754 | -2.104192 |
| 45 | 1 | 0 | -1.168095 | 2.537741 | -3.784581 |
| 46 | 1 | 0 | 0.524566 | 3.007688 | -3.655298 |
| 47 | 7 | 0 | -0.289611 | 1.897509 | -0.840104 |
| 48 | 8 | 0 | 0.220417 | 0.492982 | -2.600122 |

| | | | | | |
|---|---|---|---|---|---|
| 49 | 8 | 0 | -0.905418 | 3.786960 | 0.328757 |
| 50 | 6 | 0 | -5.997754 | -1.695117 | -0.399153 |
| 51 | 1 | 0 | -6.443279 | -0.742767 | -0.704531 |
| 52 | 1 | 0 | -6.248284 | -2.435169 | -1.166901 |
| 53 | 1 | 0 | -6.469503 | -2.006655 | 0.536547 |
| 54 | 6 | 0 | 6.299426 | -0.128920 | 0.025707 |
| 55 | 1 | 0 | 6.717264 | 0.230257 | -0.921671 |
| 56 | 1 | 0 | 6.411934 | 0.674528 | 0.759780 |
| 57 | 1 | 0 | 6.901820 | -0.980474 | 0.353689 |
| 58 | 17 | 0 | 0.335671 | -2.654391 | -1.946387 |

---------------------------------------------------------------------

| System: | **2** |
|---|---|
| Oxidant: | NCS |
| Nitrogen-donor: | $TMSN_3$ |
| Stationary point: | TS2 |
| M06–2X/6–31+G(d) energy (in a.u.): | -2011.26349316 |
| Thermal correction to Gibbs Free Energy (in a.u.): | 0.398413 |
| Number of imaginary frequencies: | 1 (–29 $cm^{-1}$) |

**CARTESIAN COORDINATES**

| Center Number | Atomic Number | Atomic Type | Coordinates (Angstroms) X | Y | Z |
|---|---|---|---|---|---|
| 1 | 6 | 0 | 0.009035 | -1.109797 | 0.298170 |
| 2 | 6 | 0 | 1.052687 | -1.686866 | -0.320993 |
| 3 | 6 | 0 | -1.413305 | -1.501845 | 0.256425 |
| 4 | 6 | 0 | -2.109898 | -1.715141 | 1.447922 |
| 5 | 6 | 0 | -2.086137 | -1.646249 | -0.963553 |
| 6 | 6 | 0 | -3.455416 | -2.070585 | 1.426001 |
| 7 | 1 | 0 | -1.598867 | -1.596257 | 2.401509 |
| 8 | 6 | 0 | -3.431768 | -1.991363 | -0.974341 |
| 9 | 1 | 0 | -1.546234 | -1.470831 | -1.888892 |
| 10 | 6 | 0 | -4.138370 | -2.209023 | 0.215760 |
| 11 | 1 | 0 | -3.983077 | -2.234317 | 2.362153 |
| 12 | 1 | 0 | -3.949092 | -2.092194 | -1.925907 |
| 13 | 7 | 0 | 0.261108 | 0.035293 | 1.167148 |
| 14 | 7 | 0 | 1.213540 | -0.148887 | 1.978739 |
| 15 | 7 | 0 | 2.065542 | -0.271509 | 2.690837 |
| 16 | 6 | 0 | 2.438036 | -1.169124 | -0.337805 |
| 17 | 6 | 0 | 3.517818 | -2.014179 | -0.063103 |
| 18 | 6 | 0 | 2.684188 | 0.187129 | -0.598046 |
| 19 | 6 | 0 | 4.815176 | -1.509673 | -0.028152 |
| 20 | 1 | 0 | 3.340703 | -3.069347 | 0.126612 |
| 21 | 6 | 0 | 3.984071 | 0.677452 | -0.561971 |
| 22 | 1 | 0 | 1.851017 | 0.835474 | -0.853378 |
| 23 | 6 | 0 | 5.070251 | -0.158369 | -0.274994 |
| 24 | 1 | 0 | 5.642590 | -2.178765 | 0.194286 |
| 25 | 1 | 0 | 4.160387 | 1.730179 | -0.771885 |
| 26 | 14 | 0 | -0.638974 | 1.778690 | 1.420264 |
| 27 | 6 | 0 | 0.642488 | 3.126558 | 1.104487 |
| 28 | 1 | 0 | 0.482372 | 3.932302 | 1.829926 |
| 29 | 1 | 0 | 1.634618 | 2.701072 | 1.318510 |
| 30 | 1 | 0 | 0.645629 | 3.545937 | 0.100251 |
| 31 | 6 | 0 | -0.350959 | 1.692085 | 3.339814 |
| 32 | 1 | 0 | -0.686041 | 0.754332 | 3.804810 |
| 33 | 1 | 0 | 0.667932 | 1.904117 | 3.686816 |
| 34 | 1 | 0 | -0.984526 | 2.483907 | 3.764034 |
| 35 | 6 | 0 | -2.503739 | 1.521628 | 1.495751 |
| 36 | 1 | 0 | -2.956450 | 2.495650 | 1.707525 |
| 37 | 1 | 0 | -2.938890 | 1.128602 | 0.574847 |
| 38 | 1 | 0 | -2.737143 | 0.846079 | 2.324025 |
| 39 | 6 | 0 | -1.640057 | 2.968725 | -1.061982 |
| 40 | 6 | 0 | -0.644680 | 1.217629 | -2.044961 |
| 41 | 6 | 0 | -1.989082 | 3.150897 | -2.536342 |
| 42 | 6 | 0 | -1.360163 | 1.932653 | -3.195024 |
| 43 | 1 | 0 | -3.076278 | 3.210081 | -2.636579 |
| 44 | 1 | 0 | -1.576356 | 4.103795 | -2.879654 |
| 45 | 1 | 0 | -2.090786 | 1.238583 | -3.622695 |
| 46 | 1 | 0 | -0.632048 | 2.162810 | -3.976807 |
| 47 | 7 | 0 | -0.916848 | 1.816085 | -0.838932 |
| 48 | 8 | 0 | 0.072718 | 0.243069 | -2.229204 |

| | | | | | |
|---|---|---|---|---|---|
| 49 | 8 | 0 | -1.950612 | 3.765969 | -0.186322 |
| 50 | 6 | 0 | -5.597662 | -2.583538 | 0.178407 |
| 51 | 1 | 0 | -6.184740 | -1.817887 | -0.339583 |
| 52 | 1 | 0 | -5.745354 | -3.527846 | -0.356408 |
| 53 | 1 | 0 | -6.004792 | -2.697995 | 1.186365 |
| 54 | 6 | 0 | 6.474859 | 0.387156 | -0.263055 |
| 55 | 1 | 0 | 6.825875 | 0.575707 | -1.283844 |
| 56 | 1 | 0 | 6.525822 | 1.334946 | 0.281629 |
| 57 | 1 | 0 | 7.170043 | -0.314551 | 0.205479 |
| 58 | 17 | 0 | 0.796755 | -3.173611 | -1.182804 |

---------------------------------------------------------------------

| System: | **2** |
|---|---|
| Oxidant: | NCS |
| Nitrogen-donor: | $TMSN_3$ |
| Stationary point: | IN2 |
| M06–2X/6–31+G(d) energy (in a.u.): | -1242.19443514 |
| Thermal correction to Gibbs Free Energy (in a.u.): | 0.216578 |
| Number of imaginary frequencies: | 0 |

**CARTESIAN COORDINATES**

| Center Number | Atomic Number | Atomic Type | Coordinates (Angstroms) X | Y | Z |
|---|---|---|---|---|---|
| 1 | 6 | 0 | 0.564826 | 0.409380 | 0.009517 |
| 2 | 6 | 0 | -0.343285 | -0.573633 | -0.185401 |
| 3 | 6 | 0 | 2.035814 | 0.243612 | 0.073937 |
| 4 | 6 | 0 | 2.867434 | 1.162085 | -0.571822 |
| 5 | 6 | 0 | 2.623590 | -0.787180 | 0.817696 |
| 6 | 6 | 0 | 4.252394 | 1.033165 | -0.504698 |
| 7 | 1 | 0 | 2.424863 | 1.983799 | -1.127096 |
| 8 | 6 | 0 | 4.005756 | -0.902889 | 0.889722 |
| 9 | 1 | 0 | 1.993602 | -1.491296 | 1.352622 |
| 10 | 6 | 0 | 4.844207 | -0.001290 | 0.222878 |
| 11 | 1 | 0 | 4.882504 | 1.752713 | -1.021476 |
| 12 | 1 | 0 | 4.444965 | -1.704054 | 1.479801 |
| 13 | 7 | 0 | 0.183768 | 1.771608 | 0.202448 |
| 14 | 7 | 0 | -0.821231 | 2.177458 | -0.401003 |
| 15 | 7 | 0 | -1.706807 | 2.671358 | -0.892992 |
| 16 | 6 | 0 | -1.805685 | -0.438657 | -0.013094 |
| 17 | 6 | 0 | -2.701534 | -0.891299 | -0.990297 |
| 18 | 6 | 0 | -2.320186 | 0.137874 | 1.153188 |
| 19 | 6 | 0 | -4.071123 | -0.746597 | -0.810416 |
| 20 | 1 | 0 | -2.316857 | -1.356104 | -1.893752 |
| 21 | 6 | 0 | -3.695333 | 0.276228 | 1.326218 |
| 22 | 1 | 0 | -1.636289 | 0.476449 | 1.927723 |
| 23 | 6 | 0 | -4.592513 | -0.158671 | 0.348809 |
| 24 | 1 | 0 | -4.751742 | -1.096199 | -1.583443 |
| 25 | 1 | 0 | -4.075346 | 0.724894 | 2.240578 |
| 26 | 6 | 0 | -6.081366 | -0.003782 | 0.524180 |
| 27 | 1 | 0 | -6.495500 | 0.669860 | -0.233845 |
| 28 | 1 | 0 | -6.592312 | -0.966796 | 0.421832 |
| 29 | 1 | 0 | -6.324585 | 0.406211 | 1.507936 |
| 30 | 6 | 0 | 6.342054 | -0.153527 | 0.293951 |
| 31 | 1 | 0 | 6.850076 | 0.710166 | -0.143316 |
| 32 | 1 | 0 | 6.679160 | -0.258316 | 1.330246 |
| 33 | 1 | 0 | 6.669402 | -1.046903 | -0.249398 |
| 34 | 17 | 0 | 0.182803 | -2.163312 | -0.698458 |

| System: | **2** |
|---|---|
| Oxidant: | NCS |
| Nitrogen-donor: | $TMSN_3$ |
| Stationary point: | TS3 |
| M06–2X/6–31+G(d) energy (in a.u.): | -1242.14580123 |
| Thermal correction to Gibbs Free Energy (in a.u.): | 0.213242 |
| Number of imaginary frequencies: | 1 (–589 $cm^{-1}$) |

**CARTESIAN COORDINATES**

| Center Number | Atomic Number | Atomic Type | Coordinates (Angstroms) X | Y | Z |
|---|---|---|---|---|---|
| 1 | 6 | 0 | -0.823971 | -1.282123 | -0.151326 |
| 2 | 6 | 0 | 0.501284 | -1.612127 | 0.112190 |
| 3 | 6 | 0 | -1.363184 | 0.095072 | -0.004322 |
| 4 | 6 | 0 | -2.316311 | 0.579846 | -0.903221 |
| 5 | 6 | 0 | -0.975248 | 0.901017 | 1.073002 |
| 6 | 6 | 0 | -2.858097 | 1.852869 | -0.736767 |
| 7 | 1 | 0 | -2.612285 | -0.039170 | -1.746584 |
| 8 | 6 | 0 | -1.521676 | 2.167331 | 1.233986 |
| 9 | 1 | 0 | -0.241740 | 0.526031 | 1.782546 |
| 10 | 6 | 0 | -2.471144 | 2.665469 | 0.331588 |
| 11 | 1 | 0 | -3.590141 | 2.221938 | -1.451006 |
| 12 | 1 | 0 | -1.211979 | 2.782961 | 2.075526 |
| 13 | 6 | 0 | 1.626183 | -0.680008 | -0.046791 |
| 14 | 6 | 0 | 2.771915 | -0.778606 | 0.757589 |
| 15 | 6 | 0 | 1.570771 | 0.348731 | -1.000851 |
| 16 | 6 | 0 | 3.802710 | 0.145666 | 0.636282 |
| 17 | 1 | 0 | 2.845450 | -1.575313 | 1.491078 |
| 18 | 6 | 0 | 2.611364 | 1.261256 | -1.119923 |
| 19 | 1 | 0 | 0.710117 | 0.424738 | -1.658255 |
| 20 | 6 | 0 | 3.743708 | 1.180585 | -0.302830 |
| 21 | 1 | 0 | 4.673260 | 0.059488 | 1.282161 |
| 22 | 1 | 0 | 2.546703 | 2.047159 | -1.868681 |
| 23 | 7 | 0 | -1.507697 | -2.277584 | -0.714995 |
| 24 | 7 | 0 | -3.121938 | -2.265888 | -0.126199 |
| 25 | 7 | 0 | -4.161238 | -2.568577 | -0.356042 |
| 26 | 6 | 0 | 4.883719 | 2.153099 | -0.459712 |
| 27 | 1 | 0 | 5.433932 | 2.274086 | 0.477741 |
| 28 | 1 | 0 | 5.593389 | 1.799933 | -1.216963 |
| 29 | 1 | 0 | 4.525410 | 3.136758 | -0.776668 |
| 30 | 6 | 0 | -3.065988 | 4.036401 | 0.528574 |
| 31 | 1 | 0 | -2.284972 | 4.785228 | 0.694620 |
| 32 | 1 | 0 | -3.652737 | 4.343321 | -0.341333 |
| 33 | 1 | 0 | -3.727177 | 4.052825 | 1.402205 |
| 34 | 17 | 0 | 0.931334 | -3.250736 | 0.502909 |

| System: | **2** |
|---|---|
| Oxidant: | NCS |
| Nitrogen-donor: | $TMSN_3$ |
| Stationary point: | IN3 |
| M06–2X/6–31+G(d) energy (in a.u.): | -1132.76553543 |
| Thermal correction to Gibbs Free Energy (in a.u.): | 0.208350 |
| Number of imaginary frequencies: | 0 |

**CARTESIAN COORDINATES**

---------------------------------------------------------------------

| Center Number | Atomic Number | Atomic Type | Coordinates (Angstroms) X | Y | Z |
|---|---|---|---|---|---|
| 1 | 6 | 0 | 0.658699 | 0.884121 | 0.660857 |
| 2 | 6 | 0 | -0.655778 | 1.326999 | 0.292284 |
| 3 | 7 | 0 | 0.018590 | 1.433429 | 1.606821 |
| 4 | 6 | 0 | 1.914330 | 0.248364 | 0.326762 |
| 5 | 6 | 0 | 2.116551 | -0.244040 | -0.964049 |
| 6 | 6 | 0 | 2.920999 | 0.124953 | 1.293987 |
| 7 | 6 | 0 | 3.322504 | -0.860244 | -1.285648 |
| 8 | 1 | 0 | 1.328117 | -0.139095 | -1.704597 |
| 9 | 6 | 0 | 4.117641 | -0.491100 | 0.960517 |
| 10 | 1 | 0 | 2.751632 | 0.515300 | 2.293626 |
| 11 | 6 | 0 | 4.335974 | -0.992359 | -0.332239 |
| 12 | 1 | 0 | 3.480432 | -1.242741 | -2.290331 |
| 13 | 1 | 0 | 4.901299 | -0.588211 | 1.708050 |
| 14 | 6 | 0 | -1.832369 | 0.412323 | 0.145843 |
| 15 | 6 | 0 | -2.684971 | 0.450061 | -0.957680 |
| 16 | 6 | 0 | -2.066578 | -0.541154 | 1.144266 |
| 17 | 6 | 0 | -3.745544 | -0.449194 | -1.059531 |
| 18 | 1 | 0 | -2.529258 | 1.187212 | -1.738262 |
| 19 | 6 | 0 | -3.130570 | -1.428794 | 1.036379 |
| 20 | 1 | 0 | -1.417307 | -0.578287 | 2.014137 |
| 21 | 6 | 0 | -3.988848 | -1.400831 | -0.068401 |
| 22 | 1 | 0 | -4.398570 | -0.402559 | -1.927674 |
| 23 | 1 | 0 | -3.300438 | -2.156377 | 1.827094 |
| 24 | 6 | 0 | 5.643598 | -1.658886 | -0.672156 |
| 25 | 1 | 0 | 6.484613 | -0.980234 | -0.495710 |
| 26 | 1 | 0 | 5.802011 | -2.545762 | -0.049419 |
| 27 | 1 | 0 | 5.671764 | -1.970136 | -1.719304 |
| 28 | 6 | 0 | -5.142599 | -2.366162 | -0.169388 |
| 29 | 1 | 0 | -4.797273 | -3.402835 | -0.096939 |
| 30 | 1 | 0 | -5.862626 | -2.202981 | 0.639862 |
| 31 | 1 | 0 | -5.672654 | -2.251976 | -1.118848 |
| 32 | 17 | 0 | -0.780594 | 2.861196 | -0.629426 |

---------------------------------------------------------------------

| System: | **2** |
| --- | --- |
| Oxidant: | NCS |
| Nitrogen-donor: | $TMSN_3$ |
| Stationary point: | TS4 |
| M06–2X/6–31+G(d) energy (in a.u.): | -1706.05189513 |
| Thermal correction to Gibbs Free Energy (in a.u.): | 0.322073 |
| Number of imaginary frequencies: | 1 (–237 $cm^{-1}$) |

**CARTESIAN COORDINATES**

| Center Number | Atomic Number | Atomic Type | Coordinates (Angstroms) X | Y | Z |
| --- | --- | --- | --- | --- | --- |
| 1 | 6 | 0 | -0.542810 | -0.455703 | -0.910988 |
| 2 | 6 | 0 | 0.831195 | -0.631460 | -0.952221 |
| 3 | 7 | 0 | 0.196083 | -0.298168 | -2.053010 |
| 4 | 6 | 0 | -1.826135 | -1.035470 | -0.590875 |
| 5 | 6 | 0 | -2.072061 | -1.557666 | 0.683625 |
| 6 | 6 | 0 | -2.819871 | -1.057642 | -1.582582 |
| 7 | 6 | 0 | -3.313214 | -2.133738 | 0.944768 |
| 8 | 1 | 0 | -1.312596 | -1.456822 | 1.457178 |
| 9 | 6 | 0 | -4.047513 | -1.631900 | -1.300013 |
| 10 | 1 | 0 | -2.612037 | -0.631972 | -2.560713 |
| 11 | 6 | 0 | -4.309962 | -2.179919 | -0.032390 |
| 12 | 1 | 0 | -3.513311 | -2.535785 | 1.933860 |
| 13 | 1 | 0 | -4.820288 | -1.660291 | -2.064145 |
| 14 | 6 | 0 | 2.151827 | -1.006066 | -0.547173 |
| 15 | 6 | 0 | 2.431638 | -1.400952 | 0.766996 |
| 16 | 6 | 0 | 3.163266 | -0.985581 | -1.525273 |
| 17 | 6 | 0 | 3.731963 | -1.775331 | 1.090133 |
| 18 | 1 | 0 | 1.648498 | -1.349668 | 1.519789 |
| 19 | 6 | 0 | 4.448669 | -1.361371 | -1.181872 |
| 20 | 1 | 0 | 2.923306 | -0.675902 | -2.539233 |
| 21 | 6 | 0 | 4.750368 | -1.760501 | 0.132724 |
| 22 | 1 | 0 | 3.960266 | -2.070385 | 2.110214 |
| 23 | 1 | 0 | 5.236965 | -1.347368 | -1.930174 |
| 24 | 7 | 0 | -1.016987 | 1.564244 | -0.390071 |
| 25 | 7 | 0 | -2.003272 | 1.557492 | 0.353647 |
| 26 | 7 | 0 | -2.927726 | 1.461971 | 0.987901 |
| 27 | 14 | 0 | 0.153711 | 2.954098 | -0.189340 |
| 28 | 6 | 0 | -0.134628 | 3.996278 | -1.724087 |
| 29 | 1 | 0 | -1.161114 | 4.376273 | -1.763820 |
| 30 | 1 | 0 | 0.544899 | 4.856364 | -1.744643 |
| 31 | 1 | 0 | 0.041232 | 3.405341 | -2.630089 |
| 32 | 6 | 0 | 1.904132 | 2.312005 | -0.182114 |
| 33 | 1 | 0 | 2.570533 | 3.170075 | -0.025899 |
| 34 | 1 | 0 | 2.049383 | 1.605166 | 0.640708 |
| 35 | 1 | 0 | 2.190799 | 1.848994 | -1.132626 |
| 36 | 6 | 0 | -0.318302 | 3.855483 | 1.373553 |
| 37 | 1 | 0 | -0.242092 | 3.171587 | 2.226369 |
| 38 | 1 | 0 | 0.364110 | 4.698346 | 1.534184 |
| 39 | 1 | 0 | -1.336390 | 4.258520 | 1.324987 |
| 40 | 6 | 0 | -5.657385 | -2.785766 | 0.258576 |
| 41 | 1 | 0 | -5.908413 | -3.554387 | -0.479656 |
| 42 | 1 | 0 | -5.682721 | -3.241592 | 1.251112 |
| 43 | 1 | 0 | -6.440693 | -2.021193 | 0.215859 |
| 44 | 6 | 0 | 6.158172 | -2.155840 | 0.489359 |
| 45 | 1 | 0 | 6.231503 | -2.472326 | 1.532281 |
| 46 | 1 | 0 | 6.503234 | -2.978962 | -0.145225 |
| 47 | 1 | 0 | 6.844844 | -1.316014 | 0.338036 |
| 48 | 17 | 0 | 0.167961 | 0.416042 | 2.354839 |

---------------------------------------------------------------------

| System: | **2** |
|---|---|
| Oxidant: | NCS |
| Nitrogen-donor: | $TMSN_3$ |
| Stationary point: | IN4 |
| M06–2X/6–31+G(d) energy (in a.u.): | -1245.66316522 |
| Thermal correction to Gibbs Free Energy (in a.u.): | 0.323932 |
| Number of imaginary frequencies: | 0 |

**CARTESIAN COORDINATES**

---------------------------------------------------------------------

| Center Number | Atomic Number | Atomic Type | Coordinates (Angstroms) X | Y | Z |
|---|---|---|---|---|---|
| 1 | 6 | 0 | 0.384923 | 0.156415 | 0.269807 |
| 2 | 6 | 0 | -1.003736 | 0.238686 | 0.662063 |
| 3 | 7 | 0 | -0.183199 | 0.602627 | 1.558750 |
| 4 | 6 | 0 | 1.195298 | -1.093320 | 0.162518 |
| 5 | 6 | 0 | 0.775731 | -2.219888 | 0.881573 |
| 6 | 6 | 0 | 2.347533 | -1.183192 | -0.619507 |
| 7 | 6 | 0 | 1.501455 | -3.401208 | 0.816270 |
| 8 | 1 | 0 | -0.116286 | -2.170504 | 1.500672 |
| 9 | 6 | 0 | 3.067320 | -2.375992 | -0.680429 |
| 10 | 1 | 0 | 2.713000 | -0.327771 | -1.183596 |
| 11 | 6 | 0 | 2.659871 | -3.503123 | 0.034102 |
| 12 | 1 | 0 | 1.163788 | -4.264576 | 1.384179 |
| 13 | 1 | 0 | 3.962811 | -2.425527 | -1.293985 |
| 14 | 6 | 0 | -2.392621 | 0.010653 | 0.362937 |
| 15 | 6 | 0 | -2.751876 | -0.579022 | -0.854605 |
| 16 | 6 | 0 | -3.378624 | 0.371206 | 1.295593 |
| 17 | 6 | 0 | -4.092746 | -0.806017 | -1.138454 |
| 18 | 1 | 0 | -1.980141 | -0.872444 | -1.562840 |
| 19 | 6 | 0 | -4.710791 | 0.141145 | 0.996677 |
| 20 | 1 | 0 | -3.088467 | 0.824279 | 2.239497 |
| 21 | 6 | 0 | -5.088803 | -0.449187 | -0.221546 |
| 22 | 1 | 0 | -4.373672 | -1.268473 | -2.080147 |
| 23 | 1 | 0 | -5.479206 | 0.418766 | 1.713403 |
| 24 | 7 | 0 | 0.948942 | 1.314775 | -0.500655 |
| 25 | 7 | 0 | 0.726540 | 1.300406 | -1.742597 |
| 26 | 7 | 0 | 0.537662 | 1.292012 | -2.843281 |
| 27 | 14 | 0 | 2.035779 | 2.655570 | 0.308213 |
| 28 | 6 | 0 | 3.073584 | 1.661290 | 1.479698 |
| 29 | 1 | 0 | 3.748065 | 0.976581 | 0.957282 |
| 30 | 1 | 0 | 3.681969 | 2.340834 | 2.087999 |
| 31 | 1 | 0 | 2.444076 | 1.079503 | 2.161178 |
| 32 | 6 | 0 | 0.834488 | 3.824777 | 1.105237 |
| 33 | 1 | 0 | 1.376343 | 4.675534 | 1.534759 |
| 34 | 1 | 0 | 0.113495 | 4.221611 | 0.383185 |
| 35 | 1 | 0 | 0.287396 | 3.329412 | 1.912706 |
| 36 | 6 | 0 | 2.917354 | 3.342992 | -1.182224 |
| 37 | 1 | 0 | 2.240211 | 3.847343 | -1.880549 |
| 38 | 1 | 0 | 3.644610 | 4.093982 | -0.852534 |
| 39 | 1 | 0 | 3.478235 | 2.573540 | -1.724760 |
| 40 | 6 | 0 | 3.430793 | -4.795234 | -0.025832 |
| 41 | 1 | 0 | 4.309340 | -4.705923 | -0.668901 |
| 42 | 1 | 0 | 3.768118 | -5.093685 | 0.971817 |
| 43 | 1 | 0 | 2.804755 | -5.603986 | -0.416184 |
| 44 | 6 | 0 | -6.542665 | -0.695015 | -0.518037 |
| 45 | 1 | 0 | -6.970107 | -1.392866 | 0.209571 |
| 46 | 1 | 0 | -7.114170 | 0.236233 | -0.451941 |
| 47 | 1 | 0 | -6.683619 | -1.114374 | -1.516367 |

-----------------------------------------------------------------------

| System: | **2** |
|---|---|
| Oxidant: | NCS |
| Nitrogen-donor: | $TMSN_3$ |
| Stationary point: | TS5 |
| M06–2X/6–31+G(d) energy (in a.u.): | -1605.79574355 |
| Thermal correction to Gibbs Free Energy (in a.u.): | 0.399005 |
| Number of imaginary frequencies: | 1 (–58 $cm^{-1}$) |

**CARTESIAN COORDINATES**

| Center Number | Atomic Number | Atomic Type | Coordinates (Angstroms) X | Y | Z |
|---|---|---|---|---|---|
| 1 | 6 | 0 | -0.888639 | -0.535968 | 0.394353 |
| 2 | 6 | 0 | -0.803976 | 0.855049 | 0.792733 |
| 3 | 7 | 0 | -0.690106 | 0.033195 | 1.747856 |
| 4 | 6 | 0 | -2.171132 | -1.227277 | 0.054915 |
| 5 | 6 | 0 | -2.209843 | -2.443246 | -0.628153 |
| 6 | 6 | 0 | -3.380830 | -0.632760 | 0.436096 |
| 7 | 6 | 0 | -3.430358 | -3.051340 | -0.920853 |
| 8 | 1 | 0 | -1.288803 | -2.938983 | -0.923273 |
| 9 | 6 | 0 | -4.590996 | -1.246865 | 0.142962 |
| 10 | 1 | 0 | -3.373999 | 0.314831 | 0.968860 |
| 11 | 6 | 0 | -4.638760 | -2.467346 | -0.542149 |
| 12 | 1 | 0 | -3.436900 | -4.001231 | -1.449587 |
| 13 | 1 | 0 | -5.519232 | -0.770033 | 0.449950 |
| 14 | 6 | 0 | -0.994472 | 2.262638 | 0.518427 |
| 15 | 6 | 0 | -1.081584 | 2.725558 | -0.797140 |
| 16 | 6 | 0 | -1.126155 | 3.151365 | 1.589535 |
| 17 | 6 | 0 | -1.305253 | 4.074103 | -1.034167 |
| 18 | 1 | 0 | -0.945291 | 2.028148 | -1.618086 |
| 19 | 6 | 0 | -1.346782 | 4.500309 | 1.338433 |
| 20 | 1 | 0 | -1.059459 | 2.777334 | 2.607713 |
| 21 | 6 | 0 | -1.438041 | 4.979770 | 0.026707 |
| 22 | 1 | 0 | -1.368449 | 4.437716 | -2.056727 |
| 23 | 1 | 0 | -1.451316 | 5.193042 | 2.169462 |
| 24 | 7 | 0 | 0.320860 | -1.198868 | -0.138981 |
| 25 | 7 | 0 | 0.510816 | -1.026166 | -1.371759 |
| 26 | 7 | 0 | 0.671494 | -0.942281 | -2.471714 |
| 27 | 14 | 0 | 1.647923 | -2.099992 | 0.916230 |
| 28 | 6 | 0 | 2.430734 | -3.350002 | -0.234101 |
| 29 | 1 | 0 | 2.736016 | -2.920539 | -1.190437 |
| 30 | 1 | 0 | 3.348934 | -3.724930 | 0.230329 |
| 31 | 1 | 0 | 1.745392 | -4.190380 | -0.393527 |
| 32 | 6 | 0 | 0.358842 | -3.091069 | 1.895806 |
| 33 | 1 | 0 | 0.901693 | -3.782033 | 2.555501 |
| 34 | 1 | 0 | -0.264210 | -2.454898 | 2.533760 |
| 35 | 1 | 0 | -0.300007 | -3.694926 | 1.262085 |
| 36 | 6 | 0 | 2.512062 | -1.082055 | 2.228525 |
| 37 | 1 | 0 | 2.198083 | -0.036362 | 2.153649 |
| 38 | 1 | 0 | 2.244528 | -1.453710 | 3.223308 |
| 39 | 1 | 0 | 3.595987 | -1.129167 | 2.092127 |
| 40 | 7 | 0 | 2.975648 | -0.437330 | -0.437159 |
| 41 | 6 | 0 | 4.340569 | -0.603959 | -0.421911 |
| 42 | 6 | 0 | 5.065210 | 0.692480 | -0.807443 |
| 43 | 6 | 0 | 3.932174 | 1.703575 | -0.943820 |
| 44 | 6 | 0 | 2.677628 | 0.861733 | -0.694772 |
| 45 | 8 | 0 | 1.535353 | 1.335015 | -0.733159 |
| 46 | 1 | 0 | 3.966792 | 2.508671 | -0.203527 |
| 47 | 1 | 0 | 5.796021 | 0.935783 | -0.031639 |
| 48 | 8 | 0 | 4.916984 | -1.638427 | -0.118349 |

| | | | | | |
|---|---|---|---|---|---|
| 49 | 1 | 0 | 3.858143 | 2.170766 | -1.929938 |
| 50 | 1 | 0 | 5.618134 | 0.523064 | -1.736152 |
| 51 | 6 | 0 | -5.961389 | -3.119268 | -0.854633 |
| 52 | 1 | 0 | -6.525273 | -3.321017 | 0.062136 |
| 53 | 1 | 0 | -6.579557 | -2.470752 | -1.484487 |
| 54 | 1 | 0 | -5.821644 | -4.067173 | -1.380804 |
| 55 | 6 | 0 | -1.648444 | 6.446125 | -0.248108 |
| 56 | 1 | 0 | -2.368558 | 6.597065 | -1.057858 |
| 57 | 1 | 0 | -2.013684 | 6.969654 | 0.639214 |
| 58 | 1 | 0 | -0.707314 | 6.918075 | -0.552335 |

---------------------------------------------------------------------

| System: | **2** |
|---|---|
| Oxidant: | NCS |
| Nitrogen-donor: | $TMSN_3$ |
| Stationary point: | IN5 |
| M06–2X/6–31+G(d) energy (in a.u.): | -836.704529787 |
| Thermal correction to Gibbs Free Energy (in a.u.): | 0.220562 |
| Number of imaginary frequencies: | 0 |

**CARTESIAN COORDINATES**

| Center Number | Atomic Number | Atomic Type | Coordinates (Angstroms) X | Y | Z |
|---|---|---|---|---|---|
| 1 | 6 | 0 | 0.591456 | 0.739856 | 0.615241 |
| 2 | 6 | 0 | -0.720805 | 1.223178 | 0.248608 |
| 3 | 7 | 0 | -0.013062 | 1.319482 | 1.566590 |
| 4 | 6 | 0 | 1.821256 | 0.040525 | 0.297489 |
| 5 | 6 | 0 | 1.992909 | -0.511254 | -0.973713 |
| 6 | 6 | 0 | 2.828439 | -0.090073 | 1.262959 |
| 7 | 6 | 0 | 3.167832 | -1.192367 | -1.277649 |
| 8 | 1 | 0 | 1.203167 | -0.403986 | -1.712917 |
| 9 | 6 | 0 | 3.994575 | -0.770803 | 0.947147 |
| 10 | 1 | 0 | 2.683680 | 0.346619 | 2.247518 |
| 11 | 6 | 0 | 4.181211 | -1.332498 | -0.325393 |
| 12 | 1 | 0 | 3.300976 | -1.621193 | -2.267410 |
| 13 | 1 | 0 | 4.778555 | -0.873087 | 1.693893 |
| 14 | 6 | 0 | -1.903571 | 0.318578 | 0.131195 |
| 15 | 6 | 0 | -2.956828 | 0.594438 | -0.745440 |
| 16 | 6 | 0 | -1.961176 | -0.840504 | 0.908687 |
| 17 | 6 | 0 | -4.036063 | -0.278715 | -0.840827 |
| 18 | 1 | 0 | -2.928948 | 1.495440 | -1.348840 |
| 19 | 6 | 0 | -3.047339 | -1.705621 | 0.808325 |
| 20 | 1 | 0 | -1.157894 | -1.066780 | 1.604863 |
| 21 | 6 | 0 | -4.101557 | -1.442674 | -0.068820 |
| 22 | 1 | 0 | -4.847700 | -0.048264 | -1.528171 |
| 23 | 1 | 0 | -3.074004 | -2.600831 | 1.425177 |
| 24 | 7 | 0 | -0.900576 | 2.446902 | -0.526061 |
| 25 | 7 | 0 | 0.011092 | 3.268045 | -0.414913 |
| 26 | 7 | 0 | 0.804825 | 4.070745 | -0.368979 |
| 27 | 6 | 0 | 5.449470 | -2.081433 | -0.642659 |
| 28 | 1 | 0 | 6.331240 | -1.494016 | -0.368085 |
| 29 | 1 | 0 | 5.493714 | -3.021873 | -0.082215 |
| 30 | 1 | 0 | 5.515237 | -2.320199 | -1.707171 |
| 31 | 6 | 0 | -5.276335 | -2.380162 | -0.190988 |
| 32 | 1 | 0 | -6.219329 | -1.857678 | 0.001846 |
| 33 | 1 | 0 | -5.337567 | -2.805163 | -1.198900 |
| 34 | 1 | 0 | -5.197935 | -3.208033 | 0.518980 |

| System: | **2** |
|---|---|
| Oxidant: | NCS |
| Nitrogen-donor: | $TMSN_3$ |
| Stationary point: | TS6 |
| M06–2X/6–31+G(d) energy (in a.u.): | -836.643853191 |
| Thermal correction to Gibbs Free Energy (in a.u.): | 0.214732 |
| Number of imaginary frequencies: | 1 (–625 $cm^{-1}$) |

**CARTESIAN COORDINATES**

---------------------------------------------------------------------

| Center Number | Atomic Number | Atomic Type | Coordinates (Angstroms) X | Y | Z |
|---|---|---|---|---|---|
| 1 | 6 | 0 | 0.560162 | 0.660352 | 1.236226 |
| 2 | 6 | 0 | -0.696997 | 1.318698 | 0.759147 |
| 3 | 7 | 0 | -0.053445 | 1.067169 | 2.226088 |
| 4 | 6 | 0 | 1.707788 | -0.018999 | 0.667126 |
| 5 | 6 | 0 | 1.670596 | -0.402820 | -0.675818 |
| 6 | 6 | 0 | 2.842085 | -0.276472 | 1.445279 |
| 7 | 6 | 0 | 2.767403 | -1.048747 | -1.235628 |
| 8 | 1 | 0 | 0.777231 | -0.204086 | -1.263658 |
| 9 | 6 | 0 | 3.931117 | -0.917070 | 0.870520 |
| 10 | 1 | 0 | 2.859111 | 0.028534 | 2.487749 |
| 11 | 6 | 0 | 3.911899 | -1.309567 | -0.475123 |
| 12 | 1 | 0 | 2.736335 | -1.355273 | -2.278029 |
| 13 | 1 | 0 | 4.813936 | -1.120317 | 1.471801 |
| 14 | 6 | 0 | -1.843983 | 0.416227 | 0.408215 |
| 15 | 6 | 0 | -2.667374 | 0.755247 | -0.670637 |
| 16 | 6 | 0 | -2.109344 | -0.765141 | 1.106530 |
| 17 | 6 | 0 | -3.725182 | -0.069003 | -1.039521 |
| 18 | 1 | 0 | -2.473525 | 1.679567 | -1.206726 |
| 19 | 6 | 0 | -3.177020 | -1.580140 | 0.734529 |
| 20 | 1 | 0 | -1.490579 | -1.047024 | 1.953758 |
| 21 | 6 | 0 | -4.002480 | -1.249257 | -0.341985 |
| 22 | 1 | 0 | -4.352371 | 0.212783 | -1.883031 |
| 23 | 1 | 0 | -3.370428 | -2.492475 | 1.294705 |
| 24 | 7 | 0 | -0.635544 | 2.597154 | 0.321652 |
| 25 | 7 | 0 | 0.518805 | 2.758178 | -0.946534 |
| 26 | 7 | 0 | 1.028195 | 3.466430 | -1.626367 |
| 27 | 6 | 0 | 5.114472 | -1.974531 | -1.092516 |
| 28 | 1 | 0 | 4.836304 | -2.563124 | -1.970662 |
| 29 | 1 | 0 | 5.846080 | -1.223438 | -1.412101 |
| 30 | 1 | 0 | 5.611677 | -2.636384 | -0.377601 |
| 31 | 6 | 0 | -5.174288 | -2.117957 | -0.724795 |
| 32 | 1 | 0 | -6.112321 | -1.699293 | -0.342303 |
| 33 | 1 | 0 | -5.270340 | -2.200145 | -1.812034 |
| 34 | 1 | 0 | -5.070320 | -3.126751 | -0.315025 |

---------------------------------------------------------------------

| System: | **2** |
|---|---|
| Oxidant: | NBS |
| Nitrogen-donor: | $TMSN_3$ |
| Stationary point: | R |
| M06–2X/6–31+G(d) energy (in a.u.): | -4125.22778057 |
| Thermal correction to Gibbs Free Energy (in a.u.): | 0.381279 |
| Number of imaginary frequencies: | 0 |

**CARTESIAN COORDINATES**

| Center Number | Atomic Number | Atomic Type | Coordinates (Angstroms) X | Y | Z |
|---|---|---|---|---|---|
| 1 | 6 | 0 | 0.496090 | 1.192449 | -1.037352 |
| 2 | 6 | 0 | 0.866582 | 0.039277 | -1.133948 |
| 3 | 6 | 0 | 0.071085 | 2.556496 | -0.895098 |
| 4 | 6 | 0 | 0.576051 | 3.345976 | 0.146604 |
| 5 | 6 | 0 | -0.858713 | 3.111926 | -1.787986 |
| 6 | 6 | 0 | 0.157049 | 4.666815 | 0.282457 |
| 7 | 1 | 0 | 1.293062 | 2.914578 | 0.840276 |
| 8 | 6 | 0 | -1.268583 | 4.429598 | -1.636313 |
| 9 | 1 | 0 | -1.257927 | 2.499477 | -2.591137 |
| 10 | 6 | 0 | -0.768283 | 5.228788 | -0.600465 |
| 11 | 1 | 0 | 0.554515 | 5.270340 | 1.094797 |
| 12 | 1 | 0 | -1.994014 | 4.847521 | -2.330559 |
| 13 | 7 | 0 | 2.499303 | 1.025478 | 1.885233 |
| 14 | 7 | 0 | 1.554891 | 0.264803 | 2.032850 |
| 15 | 7 | 0 | 0.635574 | -0.372583 | 2.204591 |
| 16 | 6 | 0 | 1.374131 | -1.300084 | -1.238120 |
| 17 | 6 | 0 | 0.868657 | -2.334636 | -0.432680 |
| 18 | 6 | 0 | 2.416368 | -1.582033 | -2.133416 |
| 19 | 6 | 0 | 1.412490 | -3.610372 | -0.521029 |
| 20 | 1 | 0 | 0.055614 | -2.139053 | 0.259050 |
| 21 | 6 | 0 | 2.953250 | -2.863836 | -2.205363 |
| 22 | 1 | 0 | 2.802735 | -0.789661 | -2.767953 |
| 23 | 6 | 0 | 2.465453 | -3.896115 | -1.398712 |
| 24 | 1 | 0 | 1.007758 | -4.400243 | 0.107533 |
| 25 | 1 | 0 | 3.764305 | -3.065127 | -2.900926 |
| 26 | 14 | 0 | 4.073106 | 0.556326 | 1.196217 |
| 27 | 6 | 0 | 4.195793 | -1.313792 | 1.152511 |
| 28 | 1 | 0 | 5.150690 | -1.616878 | 0.706948 |
| 29 | 1 | 0 | 4.152053 | -1.731307 | 2.165001 |
| 30 | 1 | 0 | 3.395657 | -1.770664 | 0.560044 |
| 31 | 6 | 0 | 5.347881 | 1.293975 | 2.352657 |
| 32 | 1 | 0 | 5.197757 | 2.374370 | 2.451954 |
| 33 | 1 | 0 | 5.278497 | 0.851721 | 3.351817 |
| 34 | 1 | 0 | 6.363016 | 1.127332 | 1.974778 |
| 35 | 6 | 0 | 4.209574 | 1.347582 | -0.494386 |
| 36 | 1 | 0 | 3.405776 | 1.014479 | -1.157476 |
| 37 | 1 | 0 | 4.139111 | 2.437925 | -0.408805 |
| 38 | 1 | 0 | 5.171192 | 1.105505 | -0.962109 |
| 39 | 6 | 0 | -4.933196 | -0.664377 | 0.351973 |
| 40 | 6 | 0 | -3.545123 | -2.496789 | 0.869845 |
| 41 | 6 | 0 | -5.851919 | -1.723306 | 0.946462 |
| 42 | 6 | 0 | -4.947818 | -2.914782 | 1.286120 |
| 43 | 1 | 0 | -6.349655 | -1.291790 | 1.818447 |
| 44 | 1 | 0 | -6.623661 | -1.958373 | 0.209273 |
| 45 | 1 | 0 | -4.928834 | -3.157074 | 2.351671 |
| 46 | 1 | 0 | -5.207900 | -3.827991 | 0.745080 |
| 47 | 7 | 0 | -3.642020 | -1.201102 | 0.357231 |
| 48 | 8 | 0 | -2.528598 | -3.136478 | 0.952124 |
| 49 | 8 | 0 | -5.231152 | 0.427653 | -0.052971 |
| 50 | 6 | 0 | 3.054719 | -5.281965 | -1.459600 |
| 51 | 1 | 0 | 3.595782 | -5.516004 | -0.535934 |
| 52 | 1 | 0 | 2.271581 | -6.037249 | -1.580334 |
| 53 | 1 | 0 | 3.754876 | -5.380389 | -2.293547 |
| 54 | 6 | 0 | -1.229574 | 6.655871 | -0.449464 |
| 55 | 1 | 0 | -2.311537 | 6.701969 | -0.285587 |
| 56 | 1 | 0 | -0.738701 | 7.144164 | 0.396575 |

| | | | | | |
|---|---|---|---|---|---|
| 57 | 1 | 0 | -1.009582 | 7.237646 | -1.350976 |
| 58 | 35 | 0 | -2.159533 | -0.294267 | -0.255876 |

---------------------------------------------------------------------

| System: | **2** |
|---|---|
| Oxidant: | NBS |
| Nitrogen-donor: | $TMSN_3$ |
| Stationary point: | TS1 |
| M06–2X/6–31+G(d) energy (in a.u.): | -4125.17640824 |
| Thermal correction to Gibbs Free Energy (in a.u.): | 0.386047 |
| Number of imaginary frequencies: | 1 (–367 $cm^{-1}$) |

**CARTESIAN COORDINATES**

| Center Number | Atomic Number | Atomic Type | Coordinates (Angstroms) X | Y | Z |
|---|---|---|---|---|---|
| 1 | 6 | 0 | -0.849219 | 0.490583 | -0.316933 |
| 2 | 6 | 0 | -0.331217 | -0.673045 | -0.138451 |
| 3 | 6 | 0 | -0.512571 | 1.909961 | -0.336403 |
| 4 | 6 | 0 | -1.091443 | 2.805217 | -1.242986 |
| 5 | 6 | 0 | 0.447821 | 2.389667 | 0.570181 |
| 6 | 6 | 0 | -0.720931 | 4.147168 | -1.243187 |
| 7 | 1 | 0 | -1.829609 | 2.448613 | -1.955660 |
| 8 | 6 | 0 | 0.820187 | 3.726896 | 0.548980 |
| 9 | 1 | 0 | 0.919118 | 1.708672 | 1.271164 |
| 10 | 6 | 0 | 0.241373 | 4.629500 | -0.351870 |
| 11 | 1 | 0 | -1.178009 | 4.827309 | -1.957996 |
| 12 | 1 | 0 | 1.583740 | 4.070374 | 1.242540 |
| 13 | 7 | 0 | -2.681964 | 0.471065 | -0.584546 |
| 14 | 7 | 0 | -3.054646 | -0.395092 | -1.389394 |
| 15 | 7 | 0 | -3.312558 | -1.192194 | -2.140290 |
| 16 | 6 | 0 | -0.902392 | -2.008672 | -0.004352 |
| 17 | 6 | 0 | -0.408408 | -3.081266 | -0.762328 |
| 18 | 6 | 0 | -1.950383 | -2.251943 | 0.893427 |
| 19 | 6 | 0 | -0.987254 | -4.339335 | -0.656712 |
| 20 | 1 | 0 | 0.431627 | -2.916748 | -1.430981 |
| 21 | 6 | 0 | -2.513174 | -3.521907 | 1.002702 |
| 22 | 1 | 0 | -2.307621 | -1.442023 | 1.525097 |
| 23 | 6 | 0 | -2.049900 | -4.583560 | 0.222767 |
| 24 | 1 | 0 | -0.597536 | -5.155929 | -1.260350 |
| 25 | 1 | 0 | -3.319573 | -3.691799 | 1.713095 |
| 26 | 14 | 0 | -3.860901 | 1.233639 | 0.585521 |
| 27 | 6 | 0 | -5.175342 | -0.070057 | 0.862978 |
| 28 | 1 | 0 | -5.888268 | 0.272065 | 1.621675 |
| 29 | 1 | 0 | -5.744640 | -0.271667 | -0.051569 |
| 30 | 1 | 0 | -4.744178 | -1.015864 | 1.208221 |
| 31 | 6 | 0 | -4.560988 | 2.756053 | -0.239621 |
| 32 | 1 | 0 | -3.780885 | 3.508502 | -0.393800 |
| 33 | 1 | 0 | -5.007200 | 2.516401 | -1.211127 |
| 34 | 1 | 0 | -5.343674 | 3.200774 | 0.385986 |
| 35 | 6 | 0 | -2.848672 | 1.633657 | 2.096906 |
| 36 | 1 | 0 | -2.229376 | 0.787739 | 2.413071 |
| 37 | 1 | 0 | -2.183687 | 2.482948 | 1.909366 |
| 38 | 1 | 0 | -3.514767 | 1.898161 | 2.926334 |
| 39 | 6 | 0 | 4.607962 | 0.470867 | 0.715196 |
| 40 | 6 | 0 | 4.801854 | -1.530704 | -0.412955 |
| 41 | 6 | 0 | 6.115350 | 0.213424 | 0.642076 |
| 42 | 6 | 0 | 6.244072 | -1.117662 | -0.101985 |
| 43 | 1 | 0 | 6.585570 | 1.054097 | 0.124751 |
| 44 | 1 | 0 | 6.513170 | 0.189969 | 1.660184 |
| 45 | 1 | 0 | 6.794139 | -1.044912 | -1.043929 |
| 46 | 1 | 0 | 6.709959 | -1.909539 | 0.490597 |
| 47 | 7 | 0 | 3.954820 | -0.568890 | 0.095032 |
| 48 | 8 | 0 | 4.466777 | -2.533647 | -1.005760 |
| 49 | 8 | 0 | 4.079794 | 1.434312 | 1.234877 |
| 50 | 6 | 0 | -2.650010 | -5.961903 | 0.334190 |
| 51 | 1 | 0 | -3.009409 | -6.314650 | -0.638266 |
| 52 | 1 | 0 | -1.908127 | -6.684587 | 0.690707 |
| 53 | 1 | 0 | -3.492703 | -5.973196 | 1.030965 |
| 54 | 6 | 0 | 0.665505 | 6.075200 | -0.361913 |
| 55 | 1 | 0 | 1.724097 | 6.165374 | -0.627832 |
| 56 | 1 | 0 | 0.084396 | 6.656519 | -1.082776 |

| | | | | | |
|---|---|---|---|---|---|
| 57 | 1 | 0 | 0.537546 | 6.529733 | 0.625978 |
| 58 | 35 | 0 | 1.919516 | -0.606447 | -0.039987 |

---------------------------------------------------------------------

| System: | **2** |
|---|---|
| Oxidant: | NBS |
| Nitrogen-donor: | $TMSN_3$ |
| Stationary point: | IN1 |
| M06–2X/6–31+G(d) energy (in a.u.): | -4125.23869663 |
| Thermal correction to Gibbs Free Energy (in a.u.): | 0.395910 |
| Number of imaginary frequencies: | 0 |

**CARTESIAN COORDINATES**

| Center Number | Atomic Number | Atomic Type | Coordinates (Angstroms) X | Y | Z |
|---|---|---|---|---|---|
| 1 | 6 | 0 | 0.256181 | -0.807445 | -0.606260 |
| 2 | 6 | 0 | -0.748108 | -1.459867 | 0.004357 |
| 3 | 6 | 0 | 1.708312 | -1.048384 | -0.528568 |
| 4 | 6 | 0 | 2.466811 | -1.176528 | -1.694102 |
| 5 | 6 | 0 | 2.344386 | -1.099898 | 0.717673 |
| 6 | 6 | 0 | 3.845150 | -1.360763 | -1.619781 |
| 7 | 1 | 0 | 1.979554 | -1.132370 | -2.666576 |
| 8 | 6 | 0 | 3.720751 | -1.276388 | 0.779128 |
| 9 | 1 | 0 | 1.745460 | -0.981984 | 1.617442 |
| 10 | 6 | 0 | 4.492793 | -1.409018 | -0.382928 |
| 11 | 1 | 0 | 4.424507 | -1.465385 | -2.533712 |
| 12 | 1 | 0 | 4.211500 | -1.304575 | 1.749374 |
| 13 | 7 | 0 | -0.075451 | 0.296945 | -1.512889 |
| 14 | 7 | 0 | -1.018409 | 0.056747 | -2.319846 |
| 15 | 7 | 0 | -1.855180 | -0.105937 | -3.042947 |
| 16 | 6 | 0 | -2.164011 | -1.037369 | -0.011903 |
| 17 | 6 | 0 | -3.184029 | -1.917425 | -0.386125 |
| 18 | 6 | 0 | -2.485282 | 0.286150 | 0.317503 |
| 19 | 6 | 0 | -4.501375 | -1.472374 | -0.454477 |
| 20 | 1 | 0 | -2.944239 | -2.950012 | -0.625702 |
| 21 | 6 | 0 | -3.805713 | 0.716737 | 0.246054 |
| 22 | 1 | 0 | -1.688745 | 0.953402 | 0.640683 |
| 23 | 6 | 0 | -4.833954 | -0.150998 | -0.140731 |
| 24 | 1 | 0 | -5.284533 | -2.164649 | -0.753571 |
| 25 | 1 | 0 | -4.043922 | 1.745363 | 0.508716 |
| 26 | 14 | 0 | 0.630174 | 2.097254 | -1.679594 |
| 27 | 6 | 0 | -0.829317 | 3.158075 | -1.225952 |
| 28 | 1 | 0 | -0.559720 | 4.207439 | -1.388443 |
| 29 | 1 | 0 | -1.682397 | 2.917343 | -1.874396 |
| 30 | 1 | 0 | -1.106224 | 3.045718 | -0.179014 |
| 31 | 6 | 0 | 0.767621 | 2.133205 | -3.562533 |
| 32 | 1 | 0 | 1.405965 | 1.325801 | -3.940264 |
| 33 | 1 | 0 | -0.193421 | 2.097208 | -4.087792 |
| 34 | 1 | 0 | 1.245850 | 3.078323 | -3.849388 |
| 35 | 6 | 0 | 2.356941 | 2.126829 | -1.005888 |
| 36 | 1 | 0 | 2.646940 | 3.179836 | -0.923371 |
| 37 | 1 | 0 | 2.420445 | 1.684106 | -0.012142 |
| 38 | 1 | 0 | 3.031328 | 1.608166 | -1.694692 |
| 39 | 6 | 0 | 0.762606 | 2.987807 | 1.708726 |
| 40 | 6 | 0 | 0.163464 | 1.003155 | 2.517343 |
| 41 | 6 | 0 | 0.859157 | 3.186682 | 3.227579 |
| 42 | 6 | 0 | 0.447075 | 1.829133 | 3.781488 |
| 43 | 1 | 0 | 1.881279 | 3.482793 | 3.481459 |
| 44 | 1 | 0 | 0.200409 | 4.009405 | 3.520737 |
| 45 | 1 | 0 | 1.225555 | 1.328316 | 4.364784 |
| 46 | 1 | 0 | -0.455918 | 1.851893 | 4.398582 |
| 47 | 7 | 0 | 0.359813 | 1.725851 | 1.374779 |
| 48 | 8 | 0 | -0.195211 | -0.173075 | 2.564035 |

| | | | | | |
|---|---|---|---|---|---|
| 49 | 8 | 0 | 1.026362 | 3.881814 | 0.903212 |
| 50 | 6 | 0 | 5.984249 | -1.603518 | -0.288085 |
| 51 | 1 | 0 | 6.446282 | -1.631054 | -1.278475 |
| 52 | 1 | 0 | 6.449167 | -0.792484 | 0.281962 |
| 53 | 1 | 0 | 6.223280 | -2.542185 | 0.223256 |
| 54 | 6 | 0 | -6.264759 | 0.320649 | -0.179150 |
| 55 | 1 | 0 | -6.880210 | -0.325641 | -0.810975 |
| 56 | 1 | 0 | -6.699653 | 0.315665 | 0.826835 |
| 57 | 1 | 0 | -6.334778 | 1.343121 | -0.562083 |
| 58 | 35 | 0 | -0.376129 | -3.065742 | 0.930549 |

---------------------------------------------------------------------

| System: | **2** |
|---|---|
| Oxidant: | NBS |
| Nitrogen-donor: | $TMSN_3$ |
| Stationary point: | TS2 |
| M06–2X/6–31+G(d) energy (in a.u.): | -4125.23486588 |
| Thermal correction to Gibbs Free Energy (in a.u.): | 0.397889 |
| Number of imaginary frequencies: | 1 (–30 $cm^{-1}$) |

**CARTESIAN COORDINATES**

---------------------------------------------------------------------

| Center Number | Atomic Number | Atomic Type | Coordinates (Angstroms) X | Y | Z |
|---|---|---|---|---|---|
| 1 | 6 | 0 | 0.015023 | -0.852552 | -0.477062 |
| 2 | 6 | 0 | -1.073379 | -1.435484 | 0.052147 |
| 3 | 6 | 0 | 1.418995 | -1.310402 | -0.462779 |
| 4 | 6 | 0 | 2.115863 | -1.439447 | -1.666324 |
| 5 | 6 | 0 | 2.075264 | -1.593786 | 0.741316 |
| 6 | 6 | 0 | 3.445242 | -1.851613 | -1.671999 |
| 7 | 1 | 0 | 1.618421 | -1.209577 | -2.606689 |
| 8 | 6 | 0 | 3.405738 | -1.993673 | 0.725333 |
| 9 | 1 | 0 | 1.535856 | -1.479357 | 1.676361 |
| 10 | 6 | 0 | 4.112033 | -2.130125 | -0.476921 |
| 11 | 1 | 0 | 3.973441 | -1.949328 | -2.617043 |
| 12 | 1 | 0 | 3.911226 | -2.201965 | 1.665693 |
| 13 | 7 | 0 | -0.157529 | 0.394118 | -1.220927 |
| 14 | 7 | 0 | -1.104518 | 0.359472 | -2.057590 |
| 15 | 7 | 0 | -1.947032 | 0.372438 | -2.791283 |
| 16 | 6 | 0 | -2.425101 | -0.837394 | 0.104287 |
| 17 | 6 | 0 | -3.550365 | -1.560604 | -0.303608 |
| 18 | 6 | 0 | -2.588425 | 0.489949 | 0.530107 |
| 19 | 6 | 0 | -4.808460 | -0.963967 | -0.306247 |
| 20 | 1 | 0 | -3.439689 | -2.593536 | -0.622245 |
| 21 | 6 | 0 | -3.850078 | 1.072741 | 0.524647 |
| 22 | 1 | 0 | -1.721648 | 1.041814 | 0.882309 |
| 23 | 6 | 0 | -4.980181 | 0.359918 | 0.105961 |
| 24 | 1 | 0 | -5.671010 | -1.539007 | -0.633474 |
| 25 | 1 | 0 | -3.960929 | 2.100937 | 0.862265 |
| 26 | 14 | 0 | 0.842517 | 2.097953 | -1.279352 |
| 27 | 6 | 0 | -0.411783 | 3.454537 | -0.900435 |
| 28 | 1 | 0 | -0.151888 | 4.336405 | -1.497093 |
| 29 | 1 | 0 | -1.396446 | 3.112878 | -1.253192 |
| 30 | 1 | 0 | -0.489726 | 3.744026 | 0.145707 |
| 31 | 6 | 0 | 0.661135 | 2.195576 | -3.209064 |
| 32 | 1 | 0 | 0.977374 | 1.282488 | -3.732577 |
| 33 | 1 | 0 | -0.325946 | 2.486737 | -3.588719 |
| 34 | 1 | 0 | 1.353992 | 2.985601 | -3.531719 |
| 35 | 6 | 0 | 2.695392 | 1.758196 | -1.269958 |
| 36 | 1 | 0 | 3.201894 | 2.727550 | -1.319428 |
| 37 | 1 | 0 | 3.044946 | 1.227722 | -0.382079 |
| 38 | 1 | 0 | 2.958970 | 1.184210 | -2.163094 |
| 39 | 6 | 0 | 1.741650 | 3.050024 | 1.351688 |
| 40 | 6 | 0 | 0.713446 | 1.219605 | 2.136072 |
| 41 | 6 | 0 | 2.032199 | 3.101299 | 2.849177 |
| 42 | 6 | 0 | 1.374549 | 1.833878 | 3.374127 |
| 43 | 1 | 0 | 3.114718 | 3.144645 | 2.996989 |
| 44 | 1 | 0 | 1.609142 | 4.023505 | 3.257641 |
| 45 | 1 | 0 | 2.083875 | 1.104873 | 3.778280 |
| 46 | 1 | 0 | 0.609596 | 2.001556 | 4.136602 |
| 47 | 7 | 0 | 1.015857 | 1.932193 | 1.002549 |
| 48 | 8 | 0 | 0.007553 | 0.221033 | 2.199974 |

| | | | | | |
|---|---|---|---|---|---|
| 49 | 8 | 0 | 2.100614 | 3.912227 | 0.559239 |
| 50 | 6 | 0 | 5.553208 | -2.570654 | -0.468798 |
| 51 | 1 | 0 | 5.975342 | -2.568989 | -1.477105 |
| 52 | 1 | 0 | 6.161703 | -1.908084 | 0.155396 |
| 53 | 1 | 0 | 5.649427 | -3.583493 | -0.062969 |
| 54 | 6 | 0 | -6.343051 | 1.002335 | 0.128233 |
| 55 | 1 | 0 | -7.074506 | 0.402607 | -0.419861 |
| 56 | 1 | 0 | -6.702804 | 1.112661 | 1.157339 |
| 57 | 1 | 0 | -6.315824 | 2.001195 | -0.318255 |
| 58 | 35 | 0 | -0.908886 | -3.166376 | 0.810273 |

---------------------------------------------------------------------

| System: | **2** |
|---|---|
| Oxidant: | NBS |
| Nitrogen-donor: | $TMSN_3$ |
| Stationary point: | IN2 |
| M06–2X/6–31+G(d) energy (in a.u.): | -3356.16611197 |
| Thermal correction to Gibbs Free Energy (in a.u.): | 0.215236 |
| Number of imaginary frequencies: | 0 |

**CARTESIAN COORDINATES**

| Center Number | Atomic Number | Atomic Type | Coordinates (Angstroms) X | Y | Z |
|---|---|---|---|---|---|
| 1 | 6 | 0 | 0.530138 | 0.667923 | -0.003987 |
| 2 | 6 | 0 | -0.368080 | -0.340222 | -0.070551 |
| 3 | 6 | 0 | 2.004170 | 0.537499 | 0.088523 |
| 4 | 6 | 0 | 2.824575 | 1.339996 | -0.707712 |
| 5 | 6 | 0 | 2.601900 | -0.337111 | 1.003525 |
| 6 | 6 | 0 | 4.210939 | 1.244183 | -0.616958 |
| 7 | 1 | 0 | 2.372346 | 2.044468 | -1.399751 |
| 8 | 6 | 0 | 3.985262 | -0.418854 | 1.097591 |
| 9 | 1 | 0 | 1.977818 | -0.944752 | 1.651901 |
| 10 | 6 | 0 | 4.813575 | 0.363060 | 0.283373 |
| 11 | 1 | 0 | 4.833562 | 1.869771 | -1.251672 |
| 12 | 1 | 0 | 4.433297 | -1.096881 | 1.820447 |
| 13 | 7 | 0 | 0.135989 | 2.040483 | 0.010110 |
| 14 | 7 | 0 | -0.885712 | 2.355817 | -0.619356 |
| 15 | 7 | 0 | -1.783526 | 2.776592 | -1.154940 |
| 16 | 6 | 0 | -1.830994 | -0.190731 | 0.085454 |
| 17 | 6 | 0 | -2.731915 | -0.713242 | -0.851509 |
| 18 | 6 | 0 | -2.339714 | 0.485241 | 1.199900 |
| 19 | 6 | 0 | -4.099654 | -0.539435 | -0.685360 |
| 20 | 1 | 0 | -2.352111 | -1.255139 | -1.713263 |
| 21 | 6 | 0 | -3.713566 | 0.651665 | 1.360206 |
| 22 | 1 | 0 | -1.651796 | 0.881043 | 1.943092 |
| 23 | 6 | 0 | -4.615102 | 0.146918 | 0.421520 |
| 24 | 1 | 0 | -4.783961 | -0.944507 | -1.427459 |
| 25 | 1 | 0 | -4.088673 | 1.178643 | 2.233959 |
| 26 | 6 | 0 | -6.102326 | 0.330784 | 0.582104 |
| 27 | 1 | 0 | -6.507564 | 0.952706 | -0.223505 |
| 28 | 1 | 0 | -6.624085 | -0.631241 | 0.548235 |
| 29 | 1 | 0 | -6.342209 | 0.813456 | 1.533154 |
| 30 | 6 | 0 | 6.313164 | 0.248066 | 0.383360 |
| 31 | 1 | 0 | 6.655177 | -0.725370 | 0.014709 |
| 32 | 1 | 0 | 6.810951 | 1.023571 | -0.204880 |
| 33 | 1 | 0 | 6.647381 | 0.341224 | 1.421725 |
| 34 | 35 | 0 | 0.213096 | -2.130619 | -0.400408 |

| System: | **2** |
|---|---|
| Oxidant: | NBS |
| Nitrogen-donor: | $TMSN_3$ |
| Stationary point: | TS3 |
| M06–2X/6–31+G(d) energy (in a.u.): | -3356.11784251 |
| Thermal correction to Gibbs Free Energy (in a.u.): | 0.210041 |
| Number of imaginary frequencies: | 1 (–582 $cm^{-1}$) |

**CARTESIAN COORDINATES**

| Center Number | Atomic Number | Atomic Type | Coordinates (Angstroms) X | Y | Z |
|---|---|---|---|---|---|
| 1 | 6 | 0 | 0.637534 | -1.129507 | 0.233425 |
| 2 | 6 | 0 | -0.730742 | -1.077052 | -0.008178 |
| 3 | 6 | 0 | 1.538365 | 0.035813 | 0.028302 |
| 4 | 6 | 0 | 2.601425 | 0.271584 | 0.903607 |
| 5 | 6 | 0 | 1.371722 | 0.876038 | -1.078970 |
| 6 | 6 | 0 | 3.471248 | 1.337582 | 0.683154 |
| 7 | 1 | 0 | 2.726591 | -0.371362 | 1.771360 |
| 8 | 6 | 0 | 2.244837 | 1.934268 | -1.293958 |
| 9 | 1 | 0 | 0.551894 | 0.692471 | -1.769057 |
| 10 | 6 | 0 | 3.308576 | 2.183732 | -0.416563 |
| 11 | 1 | 0 | 4.286737 | 1.517175 | 1.379576 |
| 12 | 1 | 0 | 2.104348 | 2.579412 | -2.158276 |
| 13 | 6 | 0 | -1.533845 | 0.146341 | 0.129182 |
| 14 | 6 | 0 | -2.642540 | 0.393913 | -0.697493 |
| 15 | 6 | 0 | -1.188273 | 1.115152 | 1.082710 |
| 16 | 6 | 0 | -3.346125 | 1.586040 | -0.596768 |
| 17 | 1 | 0 | -2.940625 | -0.349694 | -1.430068 |
| 18 | 6 | 0 | -1.907600 | 2.301761 | 1.182731 |
| 19 | 1 | 0 | -0.356184 | 0.931434 | 1.755756 |
| 20 | 6 | 0 | -2.993846 | 2.562026 | 0.344398 |
| 21 | 1 | 0 | -4.191243 | 1.763284 | -1.258255 |
| 22 | 1 | 0 | -1.622557 | 3.034633 | 1.933442 |
| 23 | 7 | 0 | 1.023228 | -2.253341 | 0.835456 |
| 24 | 7 | 0 | 2.578939 | -2.707419 | 0.249619 |
| 25 | 7 | 0 | 3.489758 | -3.289928 | 0.483875 |
| 26 | 6 | 0 | -3.786463 | 3.838429 | 0.458119 |
| 27 | 1 | 0 | -4.794039 | 3.640084 | 0.840302 |
| 28 | 1 | 0 | -3.303389 | 4.546419 | 1.136752 |
| 29 | 1 | 0 | -3.896050 | 4.322546 | -0.517756 |
| 30 | 6 | 0 | 4.259239 | 3.325180 | -0.672095 |
| 31 | 1 | 0 | 3.716715 | 4.244679 | -0.913200 |
| 32 | 1 | 0 | 4.889512 | 3.519631 | 0.199852 |
| 33 | 1 | 0 | 4.918298 | 3.101294 | -1.518499 |
| 34 | 35 | 0 | -1.701559 | -2.675000 | -0.346406 |

| System: | **2** |
|---|---|
| Oxidant: | NBS |
| Nitrogen-donor: | $TMSN_3$ |
| Stationary point: | IN3 |
| M06–2X/6–31+G(d) energy (in a.u.): | -3246.73710489 |
| Thermal correction to Gibbs Free Energy (in a.u.): | 0.206223 |
| Number of imaginary frequencies: | 0 |

**CARTESIAN COORDINATES**

| Center Number | Atomic Number | Atomic Type | Coordinates (Angstroms) X | Y | Z |
|---|---|---|---|---|---|
| 1 | 6 | 0 | 0.739898 | 0.424262 | 0.816523 |
| 2 | 6 | 0 | -0.587401 | 0.859707 | 0.496433 |
| 3 | 7 | 0 | 0.083241 | 0.853781 | 1.813274 |
| 4 | 6 | 0 | 2.019940 | -0.112690 | 0.414212 |
| 5 | 6 | 0 | 2.228535 | -0.467941 | -0.920006 |
| 6 | 6 | 0 | 3.045657 | -0.273843 | 1.355684 |
| 7 | 6 | 0 | 3.459906 | -0.985769 | -1.310798 |
| 8 | 1 | 0 | 1.425113 | -0.333503 | -1.639464 |
| 9 | 6 | 0 | 4.267441 | -0.790876 | 0.953079 |
| 10 | 1 | 0 | 2.870958 | 0.010170 | 2.389604 |
| 11 | 6 | 0 | 4.492549 | -1.154070 | -0.383977 |
| 12 | 1 | 0 | 3.622882 | -1.261940 | -2.348933 |
| 13 | 1 | 0 | 5.066000 | -0.916979 | 1.680186 |
| 14 | 6 | 0 | -1.745973 | -0.056157 | 0.250714 |
| 15 | 6 | 0 | -2.422806 | -0.095089 | -0.972007 |
| 16 | 6 | 0 | -2.137311 | -0.929923 | 1.266251 |
| 17 | 6 | 0 | -3.463911 | -0.995021 | -1.168846 |
| 18 | 1 | 0 | -2.140354 | 0.588197 | -1.767158 |
| 19 | 6 | 0 | -3.188413 | -1.822545 | 1.062359 |
| 20 | 1 | 0 | -1.619712 | -0.905962 | 2.221031 |
| 21 | 6 | 0 | -3.867918 | -1.872114 | -0.155551 |
| 22 | 1 | 0 | -3.978619 | -1.013274 | -2.127046 |
| 23 | 1 | 0 | -3.484500 | -2.489868 | 1.868143 |
| 24 | 6 | 0 | 5.829549 | -1.710353 | -0.799373 |
| 25 | 1 | 0 | 6.627854 | -0.984184 | -0.612990 |
| 26 | 1 | 0 | 6.069595 | -2.614476 | -0.230156 |
| 27 | 1 | 0 | 5.842869 | -1.963977 | -1.862231 |
| 28 | 6 | 0 | -5.013754 | -2.826297 | -0.378909 |
| 29 | 1 | 0 | -5.118527 | -3.522494 | 0.457612 |
| 30 | 1 | 0 | -5.959777 | -2.283978 | -0.484941 |
| 31 | 1 | 0 | -4.867693 | -3.412389 | -1.292117 |
| 32 | 35 | 0 | -0.767849 | 2.628015 | -0.345691 |

| System: | **2** |
|---|---|
| Oxidant: | NBS |
| Nitrogen-donor: | $TMSN_3$ |
| Stationary point: | TS4 |
| M06–2X/6–31+G(d) energy (in a.u.): | -3820.03100923 |
| Thermal correction to Gibbs Free Energy (in a.u.): | 0.320247 |
| Number of imaginary frequencies: | 1 (–233 $cm^{-1}$) |

**CARTESIAN COORDINATES**

| Center Number | Atomic Number | Atomic Type | Coordinates (Angstroms) X | Y | Z |
|---|---|---|---|---|---|
| 1 | 6 | 0 | -0.570157 | -0.564872 | -1.135025 |
| 2 | 6 | 0 | 0.802759 | -0.749234 | -1.185277 |
| 3 | 7 | 0 | 0.155741 | -0.447843 | -2.288833 |
| 4 | 6 | 0 | -1.859727 | -1.105978 | -0.780321 |
| 5 | 6 | 0 | -2.099915 | -1.578852 | 0.514409 |
| 6 | 6 | 0 | -2.868593 | -1.135293 | -1.756837 |
| 7 | 6 | 0 | -3.350664 | -2.114082 | 0.812696 |
| 8 | 1 | 0 | -1.329273 | -1.474704 | 1.276448 |
| 9 | 6 | 0 | -4.105305 | -1.668987 | -1.437827 |
| 10 | 1 | 0 | -2.665117 | -0.747093 | -2.751323 |
| 11 | 6 | 0 | -4.362423 | -2.167491 | -0.148589 |
| 12 | 1 | 0 | -3.545434 | -2.476609 | 1.817908 |
| 13 | 1 | 0 | -4.890050 | -1.702960 | -2.189367 |
| 14 | 6 | 0 | 2.123774 | -1.104558 | -0.770966 |
| 15 | 6 | 0 | 2.401316 | -1.447880 | 0.558040 |
| 16 | 6 | 0 | 3.139795 | -1.107941 | -1.745288 |
| 17 | 6 | 0 | 3.704286 | -1.792430 | 0.902497 |
| 18 | 1 | 0 | 1.615900 | -1.382929 | 1.307528 |
| 19 | 6 | 0 | 4.427001 | -1.455386 | -1.381359 |
| 20 | 1 | 0 | 2.901740 | -0.837787 | -2.770917 |
| 21 | 6 | 0 | 4.726797 | -1.800289 | -0.050260 |
| 22 | 1 | 0 | 3.929775 | -2.044412 | 1.934617 |
| 23 | 1 | 0 | 5.219358 | -1.460237 | -2.125417 |
| 24 | 7 | 0 | -0.994293 | 1.483072 | -0.645939 |
| 25 | 7 | 0 | -1.987868 | 1.529078 | 0.085931 |
| 26 | 7 | 0 | -2.918903 | 1.475936 | 0.715617 |
| 27 | 14 | 0 | 0.171639 | 2.886007 | -0.577952 |
| 28 | 6 | 0 | -0.093720 | 3.769095 | -2.212485 |
| 29 | 1 | 0 | -1.115714 | 4.153009 | -2.300018 |
| 30 | 1 | 0 | 0.595619 | 4.615269 | -2.315415 |
| 31 | 1 | 0 | 0.082956 | 3.085924 | -3.051059 |
| 32 | 6 | 0 | 1.916838 | 2.236757 | -0.481319 |
| 33 | 1 | 0 | 2.586918 | 3.095534 | -0.347366 |
| 34 | 1 | 0 | 2.033511 | 1.571821 | 0.380697 |
| 35 | 1 | 0 | 2.226939 | 1.723053 | -1.397797 |
| 36 | 6 | 0 | -0.319890 | 3.934656 | 0.884064 |
| 37 | 1 | 0 | -0.249473 | 3.342326 | 1.803388 |
| 38 | 1 | 0 | 0.355714 | 4.793926 | 0.967394 |
| 39 | 1 | 0 | -1.340112 | 4.322993 | 0.786184 |
| 40 | 6 | 0 | -5.720203 | -2.728079 | 0.181478 |
| 41 | 1 | 0 | -6.002341 | -3.512802 | -0.527992 |
| 42 | 1 | 0 | -5.742152 | -3.150952 | 1.188510 |
| 43 | 1 | 0 | -6.483676 | -1.944543 | 0.125329 |
| 44 | 6 | 0 | 6.137815 | -2.162413 | 0.327456 |
| 45 | 1 | 0 | 6.811906 | -1.316562 | 0.154442 |
| 46 | 1 | 0 | 6.208899 | -2.444997 | 1.380147 |
| 47 | 1 | 0 | 6.500075 | -2.998930 | -0.279377 |
| 48 | 35 | 0 | 0.169418 | 0.473297 | 2.382481 |

---------------------------------------------------------------------

| System: | **2** |
|---|---|
| Oxidant: | NBS |
| Nitrogen-donor: | $TMSN_3$ |
| Stationary point: | IN4 |
| M06–2X/6–31+G(d) energy (in a.u.): | -1245.66316522 |
| Thermal correction to Gibbs Free Energy (in a.u.): | 0.323932 |
| Number of imaginary frequencies: | 0 |

**CARTESIAN COORDINATES**

---------------------------------------------------------------------

| Center Number | Atomic Number | Atomic Type | Coordinates (Angstroms) X | Y | Z |
|---|---|---|---|---|---|
| 1 | 6 | 0 | 0.384923 | 0.156415 | 0.269807 |
| 2 | 6 | 0 | -1.003736 | 0.238686 | 0.662063 |
| 3 | 7 | 0 | -0.183199 | 0.602627 | 1.558750 |
| 4 | 6 | 0 | 1.195298 | -1.093320 | 0.162518 |
| 5 | 6 | 0 | 0.775731 | -2.219888 | 0.881573 |
| 6 | 6 | 0 | 2.347533 | -1.183192 | -0.619507 |
| 7 | 6 | 0 | 1.501455 | -3.401208 | 0.816270 |
| 8 | 1 | 0 | -0.116286 | -2.170504 | 1.500672 |
| 9 | 6 | 0 | 3.067320 | -2.375992 | -0.680429 |
| 10 | 1 | 0 | 2.713000 | -0.327771 | -1.183596 |
| 11 | 6 | 0 | 2.659871 | -3.503123 | 0.034102 |
| 12 | 1 | 0 | 1.163788 | -4.264576 | 1.384179 |
| 13 | 1 | 0 | 3.962811 | -2.425527 | -1.293985 |
| 14 | 6 | 0 | -2.392621 | 0.010653 | 0.362937 |
| 15 | 6 | 0 | -2.751876 | -0.579022 | -0.854605 |
| 16 | 6 | 0 | -3.378624 | 0.371206 | 1.295593 |
| 17 | 6 | 0 | -4.092746 | -0.806017 | -1.138454 |
| 18 | 1 | 0 | -1.980141 | -0.872444 | -1.562840 |
| 19 | 6 | 0 | -4.710791 | 0.141145 | 0.996677 |
| 20 | 1 | 0 | -3.088467 | 0.824279 | 2.239497 |
| 21 | 6 | 0 | -5.088803 | -0.449187 | -0.221546 |
| 22 | 1 | 0 | -4.373672 | -1.268473 | -2.080147 |
| 23 | 1 | 0 | -5.479206 | 0.418766 | 1.713403 |
| 24 | 7 | 0 | 0.948942 | 1.314775 | -0.500655 |
| 25 | 7 | 0 | 0.726540 | 1.300406 | -1.742597 |
| 26 | 7 | 0 | 0.537662 | 1.292012 | -2.843281 |
| 27 | 14 | 0 | 2.035779 | 2.655570 | 0.308213 |
| 28 | 6 | 0 | 3.073584 | 1.661290 | 1.479698 |
| 29 | 1 | 0 | 3.748065 | 0.976581 | 0.957282 |
| 30 | 1 | 0 | 3.681969 | 2.340834 | 2.087999 |
| 31 | 1 | 0 | 2.444076 | 1.079503 | 2.161178 |
| 32 | 6 | 0 | 0.834488 | 3.824777 | 1.105237 |
| 33 | 1 | 0 | 1.376343 | 4.675534 | 1.534759 |
| 34 | 1 | 0 | 0.113495 | 4.221611 | 0.383185 |
| 35 | 1 | 0 | 0.287396 | 3.329412 | 1.912706 |
| 36 | 6 | 0 | 2.917354 | 3.342992 | -1.182224 |
| 37 | 1 | 0 | 2.240211 | 3.847343 | -1.880549 |
| 38 | 1 | 0 | 3.644610 | 4.093982 | -0.852534 |
| 39 | 1 | 0 | 3.478235 | 2.573540 | -1.724760 |
| 40 | 6 | 0 | 3.430793 | -4.795234 | -0.025832 |
| 41 | 1 | 0 | 4.309340 | -4.705923 | -0.668901 |
| 42 | 1 | 0 | 3.768118 | -5.093685 | 0.971817 |
| 43 | 1 | 0 | 2.804755 | -5.603986 | -0.416184 |
| 44 | 6 | 0 | -6.542665 | -0.695015 | -0.518037 |
| 45 | 1 | 0 | -6.970107 | -1.392866 | 0.209571 |
| 46 | 1 | 0 | -7.114170 | 0.236233 | -0.451941 |
| 47 | 1 | 0 | -6.683619 | -1.114374 | -1.516367 |

----------------------------------------------------------------------

| System: | **2** |
|---|---|
| Oxidant: | NBS |
| Nitrogen-donor: | $TMSN_3$ |
| Stationary point: | TS5 |
| M06–2X/6–31+G(d) energy (in a.u.): | -1605.79574355 |
| Thermal correction to Gibbs Free Energy (in a.u.): | 0.399005 |
| Number of imaginary frequencies: | 1 (–58 $cm^{-1}$) |

**CARTESIAN COORDINATES**

| Center Number | Atomic Number | Atomic Type | Coordinates (Angstroms) X | Y | Z |
|---|---|---|---|---|---|
| 1 | 6 | 0 | -0.888639 | -0.535968 | 0.394353 |
| 2 | 6 | 0 | -0.803976 | 0.855049 | 0.792733 |
| 3 | 7 | 0 | -0.690106 | 0.033195 | 1.747856 |
| 4 | 6 | 0 | -2.171132 | -1.227277 | 0.054915 |
| 5 | 6 | 0 | -2.209843 | -2.443246 | -0.628153 |
| 6 | 6 | 0 | -3.380830 | -0.632760 | 0.436096 |
| 7 | 6 | 0 | -3.430358 | -3.051340 | -0.920853 |
| 8 | 1 | 0 | -1.288803 | -2.938983 | -0.923273 |
| 9 | 6 | 0 | -4.590996 | -1.246865 | 0.142962 |
| 10 | 1 | 0 | -3.373999 | 0.314831 | 0.968860 |
| 11 | 6 | 0 | -4.638760 | -2.467346 | -0.542149 |
| 12 | 1 | 0 | -3.436900 | -4.001231 | -1.449587 |
| 13 | 1 | 0 | -5.519232 | -0.770033 | 0.449950 |
| 14 | 6 | 0 | -0.994472 | 2.262638 | 0.518427 |
| 15 | 6 | 0 | -1.081584 | 2.725558 | -0.797140 |
| 16 | 6 | 0 | -1.126155 | 3.151365 | 1.589535 |
| 17 | 6 | 0 | -1.305253 | 4.074103 | -1.034167 |
| 18 | 1 | 0 | -0.945291 | 2.028148 | -1.618086 |
| 19 | 6 | 0 | -1.346782 | 4.500309 | 1.338433 |
| 20 | 1 | 0 | -1.059459 | 2.777334 | 2.607713 |
| 21 | 6 | 0 | -1.438041 | 4.979770 | 0.026707 |
| 22 | 1 | 0 | -1.368449 | 4.437716 | -2.056727 |
| 23 | 1 | 0 | -1.451316 | 5.193042 | 2.169462 |
| 24 | 7 | 0 | 0.320860 | -1.198868 | -0.138981 |
| 25 | 7 | 0 | 0.510816 | -1.026166 | -1.371759 |
| 26 | 7 | 0 | 0.671494 | -0.942281 | -2.471714 |
| 27 | 14 | 0 | 1.647923 | -2.099992 | 0.916230 |
| 28 | 6 | 0 | 2.430734 | -3.350002 | -0.234101 |
| 29 | 1 | 0 | 2.736016 | -2.920539 | -1.190437 |
| 30 | 1 | 0 | 3.348934 | -3.724930 | 0.230329 |
| 31 | 1 | 0 | 1.745392 | -4.190380 | -0.393527 |
| 32 | 6 | 0 | 0.358842 | -3.091069 | 1.895806 |
| 33 | 1 | 0 | 0.901693 | -3.782033 | 2.555501 |
| 34 | 1 | 0 | -0.264210 | -2.454898 | 2.533760 |
| 35 | 1 | 0 | -0.300007 | -3.694926 | 1.262085 |
| 36 | 6 | 0 | 2.512062 | -1.082055 | 2.228525 |
| 37 | 1 | 0 | 2.198083 | -0.036362 | 2.153649 |
| 38 | 1 | 0 | 2.244528 | -1.453710 | 3.223308 |
| 39 | 1 | 0 | 3.595987 | -1.129167 | 2.092127 |
| 40 | 7 | 0 | 2.975648 | -0.437330 | -0.437159 |
| 41 | 6 | 0 | 4.340569 | -0.603959 | -0.421911 |
| 42 | 6 | 0 | 5.065210 | 0.692480 | -0.807443 |
| 43 | 6 | 0 | 3.932174 | 1.703575 | -0.943820 |
| 44 | 6 | 0 | 2.677628 | 0.861733 | -0.694772 |
| 45 | 8 | 0 | 1.535353 | 1.335015 | -0.733159 |
| 46 | 1 | 0 | 3.966792 | 2.508671 | -0.203527 |
| 47 | 1 | 0 | 5.796021 | 0.935783 | -0.031639 |
| 48 | 8 | 0 | 4.916984 | -1.638427 | -0.118349 |

| | | | | | |
|---|---|---|---|---|---|
| 49 | 1 | 0 | 3.858143 | 2.170766 | -1.929938 |
| 50 | 1 | 0 | 5.618134 | 0.523064 | -1.736152 |
| 51 | 6 | 0 | -5.961389 | -3.119268 | -0.854633 |
| 52 | 1 | 0 | -6.525273 | -3.321017 | 0.062136 |
| 53 | 1 | 0 | -6.579557 | -2.470752 | -1.484487 |
| 54 | 1 | 0 | -5.821644 | -4.067173 | -1.380804 |
| 55 | 6 | 0 | -1.648444 | 6.446125 | -0.248108 |
| 56 | 1 | 0 | -2.368558 | 6.597065 | -1.057858 |
| 57 | 1 | 0 | -2.013684 | 6.969654 | 0.639214 |
| 58 | 1 | 0 | -0.707314 | 6.918075 | -0.552335 |

---------------------------------------------------------------------

| System: | **2** |
|---|---|
| Oxidant: | NBS |
| Nitrogen-donor: | $TMSN_3$ |
| Stationary point: | IN5 |
| M06–2X/6–31+G(d) energy (in a.u.): | -836.704529787 |
| Thermal correction to Gibbs Free Energy (in a.u.): | 0.220562 |
| Number of imaginary frequencies: | 0 |

**CARTESIAN COORDINATES**

---------------------------------------------------------------------

| Center Number | Atomic Number | Atomic Type | Coordinates (Angstroms) X | Y | Z |
|---|---|---|---|---|---|
| 1 | 6 | 0 | 0.591456 | 0.739856 | 0.615241 |
| 2 | 6 | 0 | -0.720805 | 1.223178 | 0.248608 |
| 3 | 7 | 0 | -0.013062 | 1.319482 | 1.566590 |
| 4 | 6 | 0 | 1.821256 | 0.040525 | 0.297489 |
| 5 | 6 | 0 | 1.992909 | -0.511254 | -0.973713 |
| 6 | 6 | 0 | 2.828439 | -0.090073 | 1.262959 |
| 7 | 6 | 0 | 3.167832 | -1.192367 | -1.277649 |
| 8 | 1 | 0 | 1.203167 | -0.403986 | -1.712917 |
| 9 | 6 | 0 | 3.994575 | -0.770803 | 0.947147 |
| 10 | 1 | 0 | 2.683680 | 0.346619 | 2.247518 |
| 11 | 6 | 0 | 4.181211 | -1.332498 | -0.325393 |
| 12 | 1 | 0 | 3.300976 | -1.621193 | -2.267410 |
| 13 | 1 | 0 | 4.778555 | -0.873087 | 1.693893 |
| 14 | 6 | 0 | -1.903571 | 0.318578 | 0.131195 |
| 15 | 6 | 0 | -2.956828 | 0.594438 | -0.745440 |
| 16 | 6 | 0 | -1.961176 | -0.840504 | 0.908687 |
| 17 | 6 | 0 | -4.036063 | -0.278715 | -0.840827 |
| 18 | 1 | 0 | -2.928948 | 1.495440 | -1.348840 |
| 19 | 6 | 0 | -3.047339 | -1.705621 | 0.808325 |
| 20 | 1 | 0 | -1.157894 | -1.066780 | 1.604863 |
| 21 | 6 | 0 | -4.101557 | -1.442674 | -0.068820 |
| 22 | 1 | 0 | -4.847700 | -0.048264 | -1.528171 |
| 23 | 1 | 0 | -3.074004 | -2.600831 | 1.425177 |
| 24 | 7 | 0 | -0.900576 | 2.446902 | -0.526061 |
| 25 | 7 | 0 | 0.011092 | 3.268045 | -0.414913 |
| 26 | 7 | 0 | 0.804825 | 4.070745 | -0.368979 |
| 27 | 6 | 0 | 5.449470 | -2.081433 | -0.642659 |
| 28 | 1 | 0 | 6.331240 | -1.494016 | -0.368085 |
| 29 | 1 | 0 | 5.493714 | -3.021873 | -0.082215 |
| 30 | 1 | 0 | 5.515237 | -2.320199 | -1.707171 |
| 31 | 6 | 0 | -5.276335 | -2.380162 | -0.190988 |
| 32 | 1 | 0 | -6.219329 | -1.857678 | 0.001846 |
| 33 | 1 | 0 | -5.337567 | -2.805163 | -1.198900 |
| 34 | 1 | 0 | -5.197935 | -3.208033 | 0.518980 |

---------------------------------------------------------------------

| System: | **2** |
|---|---|
| Oxidant: | NBS |
| Nitrogen-donor: | $TMSN_3$ |
| Stationary point: | TS6 |
| M06–2X/6–31+G(d) energy (in a.u.): | -836.643853191 |
| Thermal correction to Gibbs Free Energy (in a.u.): | 0.214732 |
| Number of imaginary frequencies: | 1 (–625 $cm^{-1}$) |

**CARTESIAN COORDINATES**

---------------------------------------------------------------------

| Center Number | Atomic Number | Atomic Type | Coordinates (Angstroms) X | Y | Z |
|---|---|---|---|---|---|
| 1 | 6 | 0 | 0.560162 | 0.660352 | 1.236226 |
| 2 | 6 | 0 | -0.696997 | 1.318698 | 0.759147 |
| 3 | 7 | 0 | -0.053445 | 1.067169 | 2.226088 |
| 4 | 6 | 0 | 1.707788 | -0.018999 | 0.667126 |
| 5 | 6 | 0 | 1.670596 | -0.402820 | -0.675818 |
| 6 | 6 | 0 | 2.842085 | -0.276472 | 1.445279 |
| 7 | 6 | 0 | 2.767403 | -1.048747 | -1.235628 |
| 8 | 1 | 0 | 0.777231 | -0.204086 | -1.263658 |
| 9 | 6 | 0 | 3.931117 | -0.917070 | 0.870520 |
| 10 | 1 | 0 | 2.859111 | 0.028534 | 2.487749 |
| 11 | 6 | 0 | 3.911899 | -1.309567 | -0.475123 |
| 12 | 1 | 0 | 2.736335 | -1.355273 | -2.278029 |
| 13 | 1 | 0 | 4.813936 | -1.120317 | 1.471801 |
| 14 | 6 | 0 | -1.843983 | 0.416227 | 0.408215 |
| 15 | 6 | 0 | -2.667374 | 0.755247 | -0.670637 |
| 16 | 6 | 0 | -2.109344 | -0.765141 | 1.106530 |
| 17 | 6 | 0 | -3.725182 | -0.069003 | -1.039521 |
| 18 | 1 | 0 | -2.473525 | 1.679567 | -1.206726 |
| 19 | 6 | 0 | -3.177020 | -1.580140 | 0.734529 |
| 20 | 1 | 0 | -1.490579 | -1.047024 | 1.953758 |
| 21 | 6 | 0 | -4.002480 | -1.249257 | -0.341985 |
| 22 | 1 | 0 | -4.352371 | 0.212783 | -1.883031 |
| 23 | 1 | 0 | -3.370428 | -2.492475 | 1.294705 |
| 24 | 7 | 0 | -0.635544 | 2.597154 | 0.321652 |
| 25 | 7 | 0 | 0.518805 | 2.758178 | -0.946534 |
| 26 | 7 | 0 | 1.028195 | 3.466430 | -1.626367 |
| 27 | 6 | 0 | 5.114472 | -1.974531 | -1.092516 |
| 28 | 1 | 0 | 4.836304 | -2.563124 | -1.970662 |
| 29 | 1 | 0 | 5.846080 | -1.223438 | -1.412101 |
| 30 | 1 | 0 | 5.611677 | -2.636384 | -0.377601 |
| 31 | 6 | 0 | -5.174288 | -2.117957 | -0.724795 |
| 32 | 1 | 0 | -6.112321 | -1.699293 | -0.342303 |
| 33 | 1 | 0 | -5.270340 | -2.200145 | -1.812034 |
| 34 | 1 | 0 | -5.070320 | -3.126751 | -0.315025 |

---------------------------------------------------------------------

| System: | **2** |
|---|---|
| Oxidant: | NIS |
| Nitrogen-donor: | $NaN_3$ |
| Stationary point: | R |
| M06–2X/6–31+G(d) energy (in a.u.): | -8224.16073717 |
| Thermal correction to Gibbs Free Energy (in a.u.): | 0.272738 |
| Number of imaginary frequencies: | 0 |

**CARTESIAN COORDINATES**

| Center Number | Atomic Number | Atomic Type | Coordinates (Angstroms) X | Y | Z |
|---|---|---|---|---|---|
| 1 | 6 | 0 | -1.585790 | -0.501778 | -0.356598 |
| 2 | 6 | 0 | -0.953952 | -1.539548 | -0.307968 |
| 3 | 6 | 0 | -2.336690 | 0.717901 | -0.400829 |
| 4 | 6 | 0 | -3.021852 | 1.096662 | -1.567920 |
| 5 | 6 | 0 | -2.422868 | 1.538095 | 0.739134 |
| 6 | 6 | 0 | -3.779663 | 2.268070 | -1.586431 |
| 7 | 1 | 0 | -2.970814 | 0.464331 | -2.449256 |
| 8 | 6 | 0 | -3.182380 | 2.705667 | 0.706534 |
| 9 | 1 | 0 | -1.904552 | 1.246827 | 1.648263 |
| 10 | 6 | 0 | -3.869575 | 3.095198 | -0.455731 |
| 11 | 1 | 0 | -4.311750 | 2.543997 | -2.493284 |
| 12 | 1 | 0 | -3.245917 | 3.325766 | 1.597157 |
| 13 | 7 | 0 | -4.625416 | -1.425800 | 0.918541 |
| 14 | 7 | 0 | -4.020716 | -2.420166 | 1.119803 |
| 15 | 7 | 0 | -3.425597 | -3.402716 | 1.319639 |
| 16 | 6 | 0 | -0.190773 | -2.751098 | -0.253541 |
| 17 | 6 | 0 | 0.106363 | -3.459119 | -1.426565 |
| 18 | 6 | 0 | 0.273837 | -3.237750 | 0.980755 |
| 19 | 6 | 0 | 0.852585 | -4.633262 | -1.360979 |
| 20 | 1 | 0 | -0.248441 | -3.086403 | -2.383234 |
| 21 | 6 | 0 | 1.017850 | -4.409237 | 1.029596 |
| 22 | 1 | 0 | 0.044001 | -2.691763 | 1.891703 |
| 23 | 6 | 0 | 1.318778 | -5.127008 | -0.137771 |
| 24 | 1 | 0 | 1.077554 | -5.174920 | -2.276447 |
| 25 | 1 | 0 | 1.372934 | -4.778533 | 1.989124 |
| 26 | 53 | 0 | 1.426741 | 0.700843 | -0.089253 |
| 27 | 6 | 0 | 3.199184 | 3.059831 | 0.688590 |
| 28 | 6 | 0 | 4.407973 | 1.322223 | -0.288492 |
| 29 | 6 | 0 | 4.637243 | 3.537047 | 0.663651 |
| 30 | 6 | 0 | 5.433771 | 2.387875 | 0.040369 |
| 31 | 1 | 0 | 4.681690 | 4.458724 | 0.077190 |
| 32 | 1 | 0 | 4.942138 | 3.770711 | 1.686922 |
| 33 | 1 | 0 | 5.950308 | 2.667777 | -0.881408 |
| 34 | 1 | 0 | 6.169379 | 1.954533 | 0.723441 |
| 35 | 7 | 0 | 3.159878 | 1.789472 | 0.118250 |
| 36 | 8 | 0 | 4.599166 | 0.244820 | -0.807937 |
| 37 | 8 | 0 | 2.230603 | 3.647981 | 1.116913 |
| 38 | 6 | 0 | 2.120753 | -6.398926 | -0.062539 |
| 39 | 1 | 0 | 3.080028 | -6.226861 | 0.437635 |
| 40 | 1 | 0 | 1.585557 | -7.161735 | 0.514319 |
| 41 | 1 | 0 | 2.318925 | -6.801161 | -1.059712 |
| 42 | 6 | 0 | -4.660532 | 4.374433 | -0.490851 |
| 43 | 1 | 0 | -4.002238 | 5.217653 | -0.731662 |
| 44 | 1 | 0 | -5.443517 | 4.333395 | -1.252796 |
| 45 | 1 | 0 | -5.120736 | 4.580935 | 0.479609 |
| 46 | 11 | 0 | -5.093870 | 0.637497 | 0.317539 |

| System: | **2** |
| --- | --- |
| Oxidant: | NIS |
| Nitrogen-donor: | $NaN_3$ |
| Stationary point: | TS1 |
| M06–2X/6–31+G(d) energy (in a.u.): | -8224.06859497 |
| Thermal correction to Gibbs Free Energy (in a.u.): | 0.275725 |
| Number of imaginary frequencies: | 1 (–334 $cm^{-1}$) |

**CARTESIAN COORDINATES**

| Center Number | Atomic Number | Atomic Type | Coordinates (Angstroms) X | Y | Z |
| --- | --- | --- | --- | --- | --- |
| 1 | 6 | 0 | 1.177554 | 1.224681 | 0.199207 |
| 2 | 6 | 0 | 1.401695 | -0.017390 | 0.144040 |
| 3 | 6 | 0 | 0.164652 | 2.268837 | 0.175797 |
| 4 | 6 | 0 | 0.018634 | 3.162363 | 1.248711 |
| 5 | 6 | 0 | -0.611794 | 2.465434 | -0.976705 |
| 6 | 6 | 0 | -0.872501 | 4.230036 | 1.161059 |
| 7 | 1 | 0 | 0.624312 | 3.016031 | 2.138273 |
| 8 | 6 | 0 | -1.501561 | 3.537999 | -1.054560 |
| 9 | 1 | 0 | -0.525526 | 1.760027 | -1.798288 |
| 10 | 6 | 0 | -1.647331 | 4.437023 | 0.009152 |
| 11 | 1 | 0 | -0.981126 | 4.907139 | 2.005099 |
| 12 | 1 | 0 | -2.109948 | 3.660787 | -1.947113 |
| 13 | 7 | 0 | 2.782980 | 2.520036 | 0.078123 |
| 14 | 7 | 0 | 3.711610 | 2.058915 | 0.705546 |
| 15 | 7 | 0 | 4.568313 | 1.596752 | 1.303943 |
| 16 | 6 | 0 | 2.476462 | -0.979007 | -0.042380 |
| 17 | 6 | 0 | 2.463266 | -2.223148 | 0.596520 |
| 18 | 6 | 0 | 3.542738 | -0.672503 | -0.903538 |
| 19 | 6 | 0 | 3.508020 | -3.123316 | 0.403244 |
| 20 | 1 | 0 | 1.632944 | -2.483402 | 1.246301 |
| 21 | 6 | 0 | 4.570141 | -1.584499 | -1.100703 |
| 22 | 1 | 0 | 3.554373 | 0.290258 | -1.408141 |
| 23 | 6 | 0 | 4.574369 | -2.822837 | -0.446498 |
| 24 | 1 | 0 | 3.485842 | -4.081776 | 0.915784 |
| 25 | 1 | 0 | 5.391164 | -1.330382 | -1.767821 |
| 26 | 53 | 0 | -0.966975 | -1.011311 | 0.138300 |
| 27 | 6 | 0 | -4.006883 | -1.245159 | -0.599451 |
| 28 | 6 | 0 | -3.284715 | -3.137024 | 0.508165 |
| 29 | 6 | 0 | -5.241529 | -2.143757 | -0.518747 |
| 30 | 6 | 0 | -4.762614 | -3.398301 | 0.213632 |
| 31 | 1 | 0 | -6.030561 | -1.600422 | 0.008118 |
| 32 | 1 | 0 | -5.596935 | -2.335015 | -1.534793 |
| 33 | 1 | 0 | -5.276994 | -3.579159 | 1.161089 |
| 34 | 1 | 0 | -4.839484 | -4.312078 | -0.381455 |
| 35 | 7 | 0 | -2.948501 | -1.890808 | 0.007496 |
| 36 | 8 | 0 | -2.533214 | -3.891653 | 1.083473 |
| 37 | 8 | 0 | -3.966315 | -0.145565 | -1.112226 |
| 38 | 6 | 0 | 5.706507 | -3.795115 | -0.660014 |
| 39 | 1 | 0 | 5.829539 | -4.030827 | -1.722520 |
| 40 | 1 | 0 | 6.654376 | -3.375980 | -0.304807 |
| 41 | 1 | 0 | 5.530625 | -4.731770 | -0.124168 |
| 42 | 6 | 0 | -2.652434 | 5.557526 | -0.056927 |
| 43 | 1 | 0 | -3.629104 | 5.207395 | 0.295341 |
| 44 | 1 | 0 | -2.356015 | 6.400509 | 0.573536 |
| 45 | 1 | 0 | -2.783379 | 5.918271 | -1.080897 |
| 46 | 11 | 0 | 1.612265 | 4.195664 | -0.712653 |

| System: | **2** |
|---|---|
| Oxidant: | NIS |
| Nitrogen-donor: | $NaN_3$ |
| Stationary point: | IN2 |
| M06–2X/6–31+G(d) energy (in a.u.): | -7701.91227979 |
| Thermal correction to Gibbs Free Energy (in a.u.): | 0.213655 |
| Number of imaginary frequencies: | 0 |

**CARTESIAN COORDINATES**

| Center Number | Atomic Number | Atomic Type | Coordinates (Angstroms) X | Y | Z |
|---|---|---|---|---|---|
| 1 | 6 | 0 | -0.471283 | 0.929932 | 0.012851 |
| 2 | 6 | 0 | 0.403493 | -0.100727 | 0.016084 |
| 3 | 6 | 0 | -1.949338 | 0.839579 | -0.089053 |
| 4 | 6 | 0 | -2.753056 | 1.518459 | 0.831979 |
| 5 | 6 | 0 | -2.562229 | 0.126384 | -1.121376 |
| 6 | 6 | 0 | -4.138579 | 1.452306 | 0.741644 |
| 7 | 1 | 0 | -2.284655 | 2.095570 | 1.624511 |
| 8 | 6 | 0 | -3.949978 | 0.074825 | -1.214055 |
| 9 | 1 | 0 | -1.950233 | -0.385542 | -1.858785 |
| 10 | 6 | 0 | -4.759861 | 0.730361 | -0.283368 |
| 11 | 1 | 0 | -4.749667 | 1.972931 | 1.475527 |
| 12 | 1 | 0 | -4.411522 | -0.481948 | -2.026010 |
| 13 | 7 | 0 | -0.059922 | 2.295484 | 0.085638 |
| 14 | 7 | 0 | 0.982498 | 2.563276 | 0.703727 |
| 15 | 7 | 0 | 1.894583 | 2.948366 | 1.241913 |
| 16 | 6 | 0 | 1.870220 | 0.042599 | -0.122337 |
| 17 | 6 | 0 | 2.763589 | -0.491719 | 0.815573 |
| 18 | 6 | 0 | 2.389922 | 0.737340 | -1.220021 |
| 19 | 6 | 0 | 4.132481 | -0.311460 | 0.667101 |
| 20 | 1 | 0 | 2.378478 | -1.047628 | 1.666759 |
| 21 | 6 | 0 | 3.764915 | 0.909906 | -1.363435 |
| 22 | 1 | 0 | 1.707747 | 1.144342 | -1.962609 |
| 23 | 6 | 0 | 4.658047 | 0.393217 | -0.423025 |
| 24 | 1 | 0 | 4.810140 | -0.726422 | 1.409926 |
| 25 | 1 | 0 | 4.147564 | 1.451119 | -2.225471 |
| 26 | 53 | 0 | -0.277203 | -2.102885 | 0.244715 |
| 27 | 6 | 0 | 6.145670 | 0.589201 | -0.563255 |
| 28 | 1 | 0 | 6.522439 | 1.273439 | 0.205185 |
| 29 | 1 | 0 | 6.681135 | -0.358989 | -0.450638 |
| 30 | 1 | 0 | 6.399962 | 1.009492 | -1.540116 |
| 31 | 6 | 0 | -6.262850 | 0.692128 | -0.392533 |
| 32 | 1 | 0 | -6.727055 | 0.565306 | 0.590287 |
| 33 | 1 | 0 | -6.645917 | 1.626122 | -0.819379 |
| 34 | 1 | 0 | -6.593748 | -0.128728 | -1.034665 |

| System: | **2** |
|---|---|
| Oxidant: | NIS |
| Nitrogen-donor: | $NaN_3$ |
| Stationary point: | TS3 |
| M06–2X/6–31+G(d) energy (in a.u.): | -7701.86496209 |
| Thermal correction to Gibbs Free Energy (in a.u.): | 0.210699 |
| Number of imaginary frequencies: | 1 (–567 $cm^{-1}$) |

**CARTESIAN COORDINATES**

| Center Number | Atomic Number | Atomic Type | Coordinates (Angstroms) X | Y | Z |
|---|---|---|---|---|---|
| 1 | 6 | 0 | 0.167006 | -1.165495 | 0.259616 |
| 2 | 6 | 0 | -0.898575 | -0.302766 | 0.036020 |
| 3 | 6 | 0 | 1.581579 | -0.768342 | 0.024592 |
| 4 | 6 | 0 | 2.591971 | -1.186153 | 0.896137 |
| 5 | 6 | 0 | 1.925243 | -0.011201 | -1.099633 |
| 6 | 6 | 0 | 3.917045 | -0.836051 | 0.654357 |
| 7 | 1 | 0 | 2.326194 | -1.763063 | 1.778629 |
| 8 | 6 | 0 | 3.251897 | 0.329772 | -1.337328 |
| 9 | 1 | 0 | 1.145116 | 0.312218 | -1.784438 |
| 10 | 6 | 0 | 4.268682 | -0.074523 | -0.465332 |
| 11 | 1 | 0 | 4.691575 | -1.153962 | 1.348826 |
| 12 | 1 | 0 | 3.505121 | 0.920733 | -2.214260 |
| 13 | 6 | 0 | -0.777065 | 1.158285 | 0.164488 |
| 14 | 6 | 0 | -1.450607 | 2.029571 | -0.706221 |
| 15 | 6 | 0 | 0.054412 | 1.714585 | 1.146891 |
| 16 | 6 | 0 | -1.262382 | 3.401871 | -0.617853 |
| 17 | 1 | 0 | -2.112069 | 1.621958 | -1.465419 |
| 18 | 6 | 0 | 0.225051 | 3.092459 | 1.236762 |
| 19 | 1 | 0 | 0.564399 | 1.060059 | 1.848086 |
| 20 | 6 | 0 | -0.425403 | 3.959871 | 0.356801 |
| 21 | 1 | 0 | -1.780762 | 4.056986 | -1.314674 |
| 22 | 1 | 0 | 0.872912 | 3.499345 | 2.009489 |
| 23 | 7 | 0 | -0.182271 | -2.291807 | 0.878645 |
| 24 | 7 | 0 | 0.812644 | -3.583065 | 0.299035 |
| 25 | 53 | 0 | -2.854957 | -1.063779 | -0.243282 |
| 26 | 7 | 0 | 1.207610 | -4.589563 | 0.530637 |
| 27 | 6 | 0 | -0.266133 | 5.454077 | 0.466796 |
| 28 | 1 | 0 | -0.147579 | 5.912563 | -0.519949 |
| 29 | 1 | 0 | -1.148306 | 5.905991 | 0.934849 |
| 30 | 1 | 0 | 0.604942 | 5.717635 | 1.072773 |
| 31 | 6 | 0 | 5.708662 | 0.278205 | -0.737296 |
| 32 | 1 | 0 | 5.786327 | 1.179948 | -1.351280 |
| 33 | 1 | 0 | 6.257142 | 0.449651 | 0.193676 |
| 34 | 1 | 0 | 6.213521 | -0.533721 | -1.273345 |

| | |
|---|---|
| System: | **2** |
| Oxidant: | NIS |
| Nitrogen-donor: | $NaN_3$ |
| Stationary point: | IN3 |
| M06–2X/6–31+G(d) energy (in a.u.): | -7592.48200169 |
| Thermal correction to Gibbs Free Energy (in a.u.): | 0.206497 |
| Number of imaginary frequencies: | 0 |

**CARTESIAN COORDINATES**

| Center Number | Atomic Number | Atomic Type | Coordinates (Angstroms) X | Y | Z |
|---|---|---|---|---|---|
| 1 | 6 | 0 | 0.823446 | 0.049971 | 0.895099 |
| 2 | 6 | 0 | -0.520675 | 0.448978 | 0.595613 |
| 3 | 7 | 0 | 0.149227 | 0.387348 | 1.914797 |
| 4 | 6 | 0 | 2.126623 | -0.395038 | 0.460141 |
| 5 | 6 | 0 | 2.352352 | -0.639513 | -0.896664 |
| 6 | 6 | 0 | 3.158392 | -0.576203 | 1.390561 |
| 7 | 6 | 0 | 3.607347 | -1.065207 | -1.319930 |
| 8 | 1 | 0 | 1.543194 | -0.492602 | -1.607433 |
| 9 | 6 | 0 | 4.404650 | -1.001315 | 0.955153 |
| 10 | 1 | 0 | 2.969009 | -0.381191 | 2.442465 |
| 11 | 6 | 0 | 4.647871 | -1.249449 | -0.404315 |
| 12 | 1 | 0 | 3.783834 | -1.256947 | -2.374962 |
| 13 | 1 | 0 | 5.206787 | -1.144704 | 1.674917 |
| 14 | 6 | 0 | -1.640039 | -0.497068 | 0.292348 |
| 15 | 6 | 0 | -2.178135 | -0.624414 | -0.992031 |
| 16 | 6 | 0 | -2.137096 | -1.302695 | 1.316398 |
| 17 | 6 | 0 | -3.187615 | -1.546091 | -1.240496 |
| 18 | 1 | 0 | -1.813204 | 0.009752 | -1.795911 |
| 19 | 6 | 0 | -3.156665 | -2.218921 | 1.059713 |
| 20 | 1 | 0 | -1.722383 | -1.208989 | 2.316283 |
| 21 | 6 | 0 | -3.697457 | -2.357063 | -0.218911 |
| 22 | 1 | 0 | -3.594890 | -1.635292 | -2.245359 |
| 23 | 1 | 0 | -3.537211 | -2.834310 | 1.871537 |
| 24 | 53 | 0 | -0.803884 | 2.459915 | -0.229305 |
| 25 | 6 | 0 | 6.015619 | -1.683338 | -0.863068 |
| 26 | 1 | 0 | 6.703978 | -0.830576 | -0.878290 |
| 27 | 1 | 0 | 6.437761 | -2.434771 | -0.189144 |
| 28 | 1 | 0 | 5.982467 | -2.104843 | -1.871065 |
| 29 | 6 | 0 | -4.804308 | -3.340327 | -0.504033 |
| 30 | 1 | 0 | -5.050436 | -3.929208 | 0.383689 |
| 31 | 1 | 0 | -5.714231 | -2.823394 | -0.828057 |
| 32 | 1 | 0 | -4.519281 | -4.033696 | -1.302531 |

| System: | **2** |
|---|---|
| Oxidant: | NIS |
| Nitrogen-donor: | $NaN_3$ |
| Stationary point: | TS4 |
| M06–2X/6–31+G(d) energy (in a.u.): | -7918.91240763 |
| Thermal correction to Gibbs Free Energy (in a.u.): | 0.211547 |
| Number of imaginary frequencies: | 1 (–250 $cm^{-1}$) |

**CARTESIAN COORDINATES**

| Center Number | Atomic Number | Atomic Type | Coordinates (Angstroms) X | Y | Z |
|---|---|---|---|---|---|
| 1 | 6 | 0 | -0.573800 | -0.569193 | -0.699681 |
| 2 | 6 | 0 | 0.491359 | 0.399717 | -0.663182 |
| 3 | 7 | 0 | -0.054662 | -0.103516 | -1.841439 |
| 4 | 6 | 0 | -1.980120 | -0.597299 | -0.244931 |
| 5 | 6 | 0 | -2.329208 | -0.886772 | 1.074929 |
| 6 | 6 | 0 | -2.978573 | -0.276104 | -1.166098 |
| 7 | 6 | 0 | -3.663013 | -0.850281 | 1.467492 |
| 8 | 1 | 0 | -1.550486 | -1.143017 | 1.789058 |
| 9 | 6 | 0 | -4.310892 | -0.247157 | -0.766397 |
| 10 | 1 | 0 | -2.691440 | -0.034533 | -2.185592 |
| 11 | 6 | 0 | -4.673207 | -0.530805 | 0.554158 |
| 12 | 1 | 0 | -3.926773 | -1.073229 | 2.498473 |
| 13 | 1 | 0 | -5.083735 | 0.003638 | -1.489608 |
| 14 | 6 | 0 | 1.909485 | 0.170960 | -0.264764 |
| 15 | 6 | 0 | 2.344307 | 0.258065 | 1.065499 |
| 16 | 6 | 0 | 2.812621 | -0.263397 | -1.241523 |
| 17 | 6 | 0 | 3.642623 | -0.118062 | 1.410623 |
| 18 | 1 | 0 | 1.652767 | 0.614554 | 1.824862 |
| 19 | 6 | 0 | 4.119733 | -0.620105 | -0.892714 |
| 20 | 1 | 0 | 2.460482 | -0.344323 | -2.266494 |
| 21 | 6 | 0 | 4.556756 | -0.560170 | 0.437831 |
| 22 | 1 | 0 | 3.961350 | -0.052748 | 2.448700 |
| 23 | 1 | 0 | 4.811898 | -0.944513 | -1.666766 |
| 24 | 53 | 0 | -0.075597 | 2.534123 | -0.013295 |
| 25 | 6 | 0 | -6.119098 | -0.475422 | 0.978270 |
| 26 | 1 | 0 | -6.253110 | -0.876055 | 1.986820 |
| 27 | 1 | 0 | -6.487182 | 0.556620 | 0.975045 |
| 28 | 1 | 0 | -6.752231 | -1.052043 | 0.296273 |
| 29 | 6 | 0 | 5.979217 | -0.896489 | 0.809079 |
| 30 | 1 | 0 | 6.423418 | -1.600084 | 0.099445 |
| 31 | 1 | 0 | 6.596234 | 0.008862 | 0.805320 |
| 32 | 1 | 0 | 6.041610 | -1.330271 | 1.811441 |
| 33 | 7 | 0 | 0.099998 | -2.223602 | -0.156960 |
| 34 | 7 | 0 | -0.729873 | -3.086116 | -0.396093 |
| 35 | 7 | 0 | -1.537002 | -3.857815 | -0.619887 |
| 36 | 11 | 0 | 2.271405 | -2.342630 | 0.338799 |

| System: | **2** |
|---|---|
| Oxidant: | NIS |
| Nitrogen-donor: | $NaN_3$ |
| Stationary point: | IN5 |
| M06–2X/6–31+G(d) energy (in a.u.): | -836.704529787 |
| Thermal correction to Gibbs Free Energy (in a.u.): | 0.220562 |
| Number of imaginary frequencies: | 0 |

**CARTESIAN COORDINATES**

| Center Number | Atomic Number | Atomic Type | X | Y | Z |
|---|---|---|---|---|---|
| 1 | 6 | 0 | 0.591456 | 0.739856 | 0.615241 |
| 2 | 6 | 0 | -0.720805 | 1.223178 | 0.248608 |
| 3 | 7 | 0 | -0.013062 | 1.319482 | 1.566590 |
| 4 | 6 | 0 | 1.821256 | 0.040525 | 0.297489 |
| 5 | 6 | 0 | 1.992909 | -0.511254 | -0.973713 |
| 6 | 6 | 0 | 2.828439 | -0.090073 | 1.262959 |
| 7 | 6 | 0 | 3.167832 | -1.192367 | -1.277649 |
| 8 | 1 | 0 | 1.203167 | -0.403986 | -1.712917 |
| 9 | 6 | 0 | 3.994575 | -0.770803 | 0.947147 |
| 10 | 1 | 0 | 2.683680 | 0.346619 | 2.247518 |
| 11 | 6 | 0 | 4.181211 | -1.332498 | -0.325393 |
| 12 | 1 | 0 | 3.300976 | -1.621193 | -2.267410 |
| 13 | 1 | 0 | 4.778555 | -0.873087 | 1.693893 |
| 14 | 6 | 0 | -1.903571 | 0.318578 | 0.131195 |
| 15 | 6 | 0 | -2.956828 | 0.594438 | -0.745440 |
| 16 | 6 | 0 | -1.961176 | -0.840504 | 0.908687 |
| 17 | 6 | 0 | -4.036063 | -0.278715 | -0.840827 |
| 18 | 1 | 0 | -2.928948 | 1.495440 | -1.348840 |
| 19 | 6 | 0 | -3.047339 | -1.705621 | 0.808325 |
| 20 | 1 | 0 | -1.157894 | -1.066780 | 1.604863 |
| 21 | 6 | 0 | -4.101557 | -1.442674 | -0.068820 |
| 22 | 1 | 0 | -4.847700 | -0.048264 | -1.528171 |
| 23 | 1 | 0 | -3.074004 | -2.600831 | 1.425177 |
| 24 | 7 | 0 | -0.900576 | 2.446902 | -0.526061 |
| 25 | 7 | 0 | 0.011092 | 3.268045 | -0.414913 |
| 26 | 7 | 0 | 0.804825 | 4.070745 | -0.368979 |
| 27 | 6 | 0 | 5.449470 | -2.081433 | -0.642659 |
| 28 | 1 | 0 | 6.331240 | -1.494016 | -0.368085 |
| 29 | 1 | 0 | 5.493714 | -3.021873 | -0.082215 |
| 30 | 1 | 0 | 5.515237 | -2.320199 | -1.707171 |
| 31 | 6 | 0 | -5.276335 | -2.380162 | -0.190988 |
| 32 | 1 | 0 | -6.219329 | -1.857678 | 0.001846 |
| 33 | 1 | 0 | -5.337567 | -2.805163 | -1.198900 |
| 34 | 1 | 0 | -5.197935 | -3.208033 | 0.518980 |

| System: | **2** |
|---|---|
| Oxidant: | NIS |
| Nitrogen-donor: | $NaN_3$ |
| Stationary point: | TS6 |
| M06–2X/6–31+G(d) energy (in a.u.): | -836.643853191 |
| Thermal correction to Gibbs Free Energy (in a.u.): | 0.214732 |
| Number of imaginary frequencies: | 1 (–625 $cm^{-1}$) |

**CARTESIAN COORDINATES**

| Center Number | Atomic Number | Atomic Type | Coordinates (Angstroms) X | Y | Z |
|---|---|---|---|---|---|
| 1 | 6 | 0 | 0.560162 | 0.660352 | 1.236226 |
| 2 | 6 | 0 | -0.696997 | 1.318698 | 0.759147 |
| 3 | 7 | 0 | -0.053445 | 1.067169 | 2.226088 |
| 4 | 6 | 0 | 1.707788 | -0.018999 | 0.667126 |
| 5 | 6 | 0 | 1.670596 | -0.402820 | -0.675818 |
| 6 | 6 | 0 | 2.842085 | -0.276472 | 1.445279 |
| 7 | 6 | 0 | 2.767403 | -1.048747 | -1.235628 |
| 8 | 1 | 0 | 0.777231 | -0.204086 | -1.263658 |
| 9 | 6 | 0 | 3.931117 | -0.917070 | 0.870520 |
| 10 | 1 | 0 | 2.859111 | 0.028534 | 2.487749 |
| 11 | 6 | 0 | 3.911899 | -1.309567 | -0.475123 |
| 12 | 1 | 0 | 2.736335 | -1.355273 | -2.278029 |
| 13 | 1 | 0 | 4.813936 | -1.120317 | 1.471801 |
| 14 | 6 | 0 | -1.843983 | 0.416227 | 0.408215 |
| 15 | 6 | 0 | -2.667374 | 0.755247 | -0.670637 |
| 16 | 6 | 0 | -2.109344 | -0.765141 | 1.106530 |
| 17 | 6 | 0 | -3.725182 | -0.069003 | -1.039521 |
| 18 | 1 | 0 | -2.473525 | 1.679567 | -1.206726 |
| 19 | 6 | 0 | -3.177020 | -1.580140 | 0.734529 |
| 20 | 1 | 0 | -1.490579 | -1.047024 | 1.953758 |
| 21 | 6 | 0 | -4.002480 | -1.249257 | -0.341985 |
| 22 | 1 | 0 | -4.352371 | 0.212783 | -1.883031 |
| 23 | 1 | 0 | -3.370428 | -2.492475 | 1.294705 |
| 24 | 7 | 0 | -0.635544 | 2.597154 | 0.321652 |
| 25 | 7 | 0 | 0.518805 | 2.758178 | -0.946534 |
| 26 | 7 | 0 | 1.028195 | 3.466430 | -1.626367 |
| 27 | 6 | 0 | 5.114472 | -1.974531 | -1.092516 |
| 28 | 1 | 0 | 4.836304 | -2.563124 | -1.970662 |
| 29 | 1 | 0 | 5.846080 | -1.223438 | -1.412101 |
| 30 | 1 | 0 | 5.611677 | -2.636384 | -0.377601 |
| 31 | 6 | 0 | -5.174288 | -2.117957 | -0.724795 |
| 32 | 1 | 0 | -6.112321 | -1.699293 | -0.342303 |
| 33 | 1 | 0 | -5.270340 | -2.200145 | -1.812034 |
| 34 | 1 | 0 | -5.070320 | -3.126751 | -0.315025 |
